# Dual Copper- and Aldehyde-Catalyzed Transient C–H Sulfonylation of Benzylamines

**DOI:** 10.1021/acs.orglett.3c01783

**Published:** 2023-07-13

**Authors:** Joe I. Higham, Tsz-Kan Ma, James A. Bull

**Affiliations:** Department of Chemistry, Imperial College London, Molecular Sciences Research Hub, White City Campus, Wood Lane, London W12 0BZ, United Kingdom

## Abstract

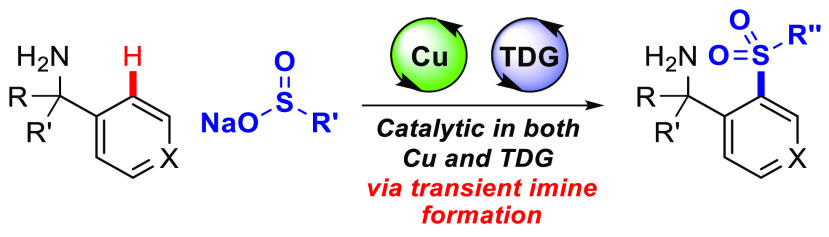

This study reports the first example of using a dual catalytic system with copper(II) acetate and 2-hydroxynicotinaldehyde to achieve transient C(sp^2^)–H sulfonylation of benzylamines with sulfinate salts via a dynamically formed imine directing group. Manganese(IV) oxide was identified as an effective oxidant and base. Computational density functional theory investigations suggest that the transient directing group lowers the energy barrier for an acetate-mediated, turnover-limiting C–H activation step and subsequent combination of the cupracycle with a RSO_2_ radical.

Developments in the functionalization of C–H bonds continue to streamline synthetic routes to medicinal compounds and materials.^1^ Transient C–H functionalization, involving an *in situ* formed transient directing group (TDG) from common functionality, presents additional opportunities for efficient synthesis by avoiding steps for directing group installation and removal (Figure 1a).^2^ Pioneering work by Jun et al.^3^ and Yu and co-workers^4^ established the potential for aldehydic C–H and benzylic C–H functionalization, respectively, with imine directing groups. Subsequent developments have enabled palladium-catalyzed C–H functionalization of benzaldehydes and aliphatic aldehydes,^5^ with fewer examples on amines.^6−15^ These approaches directly reveal useful functionality for further derivatization. To date, palladium and other precious metal catalysts have been employed almost exclusively. Given the increasing price and undesirable toxicity profile of Pd, the development of new methods relying on cheap and readily available base metals is crucial to sustainable synthesis. We recently reported the first example of copper-mediated transient C–H functionalization in the sulfonylation of benzaldehydes with sulfinate salts. β-Alanine was used as a catalytic TDG (Figure 1b).^16^

**Figure 1:**
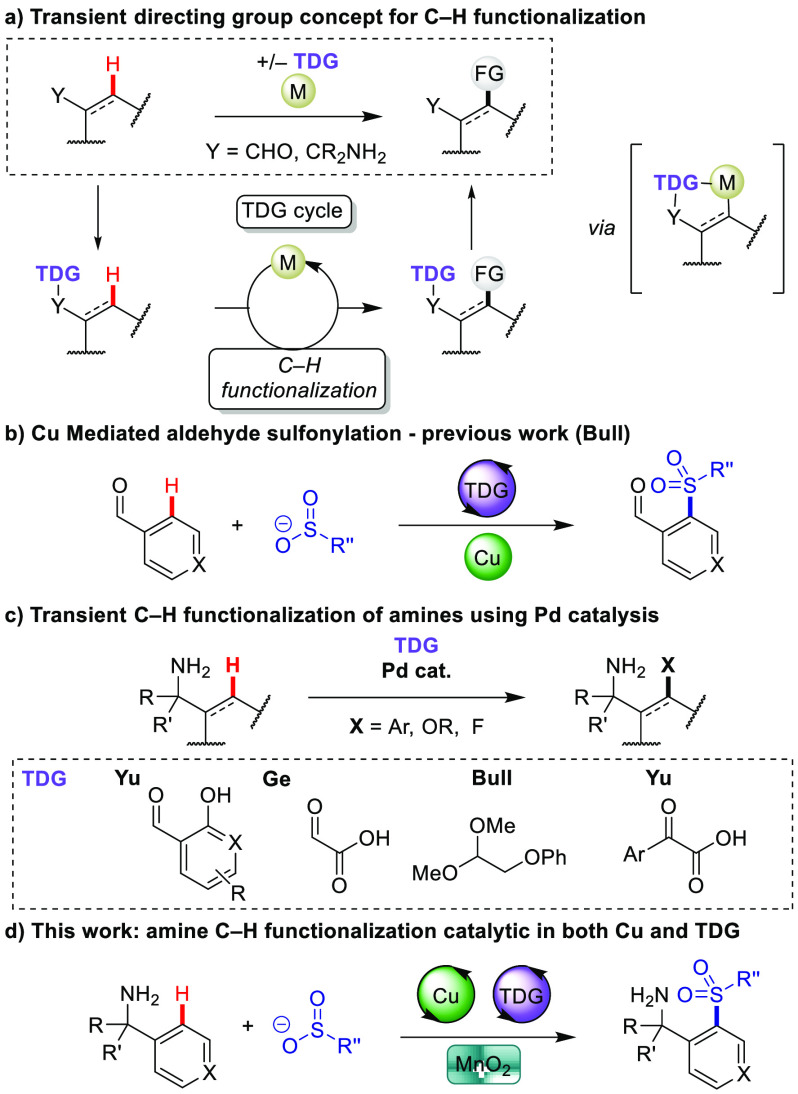
Concept of C–H functionalization using transient directing groups and copper-catalyzed transient C–H functionalization.

Amine functionalities feature in countless pharmaceutically active compounds and fine chemicals^17^ and present additional challenges for C–H functionalization as a result of coordinative poisoning of metal catalysts. There are reports of free amine-directed C(sp^2^)–H functionalization;^18^ however, robust amide and sulfonamide directing groups have been used most commonly.^19,20^ Karmakar and Samanta reported the palladium-catalyzed C–H sulfonylation of benzylamines with sulfinate salts using picolinamide as a directing group.^21^

All prior reports using TDGs with amine substrates have involved palladium catalysis (Figure 1c).^2^ Notably, Yu and co-workers developed 2-hydroxynicotinaldehyde as a powerful TDG for Pd-catalyzed C(sp^3^)–H arylation,^6^ oxygenation,^7^ and fluorination^8^ of amines. The use of this TDG for Pd-catalyzed C–H arylation of amines was also described by Kameneka and co-workers for alkyl and benzylamines.^9^ Other catalytic TDGs for amine functionalization include glyoxylic acid developed by Liu and Ge,^10^ aryl keto acids for δ-arylation,^11^ and acetal ethers.^12^ There are no examples of C–S bond formation on amine substrates using TDGs. Given the value of sulfones in medicinal chemistry,^22^ we envisaged the direct C–H sulfonylation of amine precursors to form valuable amino sulfone building blocks. Here, we report a dual copper/TDG-catalyzed C(sp^2^)–H sulfonylation of benzylamines using MnO_2_ as the terminal oxidant. This represents the first C–S-bond-forming transient C–H functionalization methodology for amines and the first example of sub-stoichiometric copper salt being used with a TDG. Computational studies reveal the mechanistic features of the reaction.

We first examined different aldehydes to function as the TDG (Scheme 1). A catalytic TDG (25 mol %) along with a stoichiometric quantity of copper fluoride was employed. Hexafluoro-2-propanol (HFIP) was used as the solvent at 100 °C in the presence of K_2_CO_3_, with 1 equiv of *p*-tolylSO_2_Na. No reaction was observed in the absence of a TDG nor using glyoxalic acid **TDG1** or 2-phenoxyacetaldehyde dimethyl acetal **TDG2**. Pleasingly, salicaldehyde (**TDG3**) afforded compound **3aa** in a 44% yield. 5-Substituted salicaldehydes (**TDG4**–**TDG6**) gave similar improved yields (54–56%), whereas 6-substituted derivatives were less effective (**TDG7** and **TDG8**). 2-Hydroxynicotinaldehyde (**TDG9**) was the most effective TDG, affording compound **3aa** in a 60% yield.

**Scheme 1:**
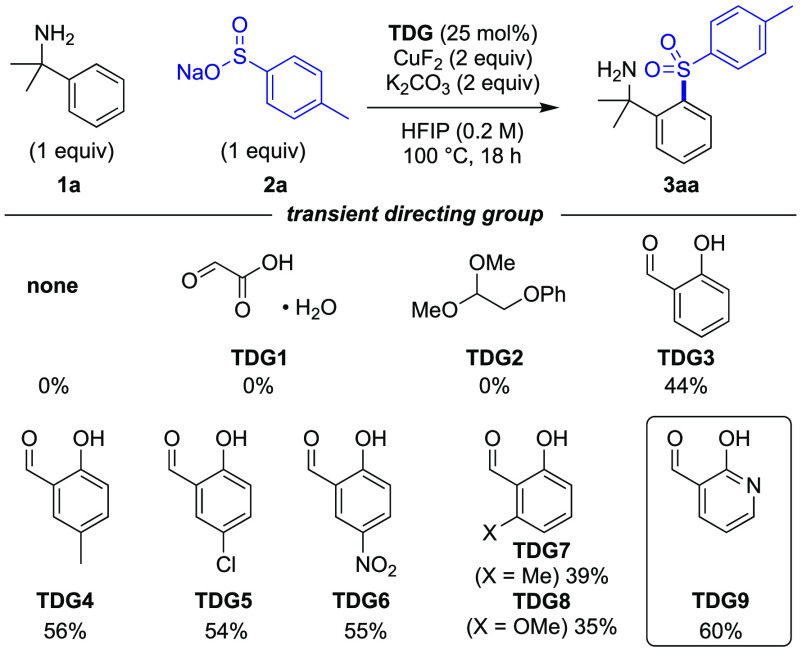
Optimization of the TDG Reactions were performed on a 0.2 mmol scale. Yields were determined by ^1^H nuclear magnetic resonance (NMR) using 1,3,5-trimethoxybenzene as an internal standard.

Encouraged by these results, different copper sources, co-oxidants, TDG loading, concentration, and base were investigated to develop a catalytic process.^23^ Inexpensive and readily available copper(II) acetate in combination with MnO_2_ was identified as an effective catalyst system. Furthermore, MnO_2_ acts as both a base and oxidant, avoiding the need for an additional base. Under these optimized conditions, compound **3aa** was isolated in 68% yield (entry 1 in Table 1).^24^ Cu(OAc)_2_ was critical for the coupling, with one turnover observed in the absence of an oxidant (entries 2 and 3).^24^ In the absence of **TDG9**, a low but non-zero yield was obtained (entry 4). Testing other TDGs under the catalytic conditions showed the same trend as that using stoichiometric copper (entries 5–7). Changing the oxidant to K_2_S_2_O_8_ was detrimental (entry 8). The addition of K_2_CO_3_ gave no change in the isolated yield (entry 9). The addition of (2,2,6,6-tetramethylpiperidin-1-yl)oxyl (TEMPO) as a radical trap fully suppressed the reaction, suggesting a radical reaction pathway in operation (entry 10). Notably, the formation of sulfonamide was never observed, despite the potential for direct coupling with the amine moiety.^25^

**Table 1:**
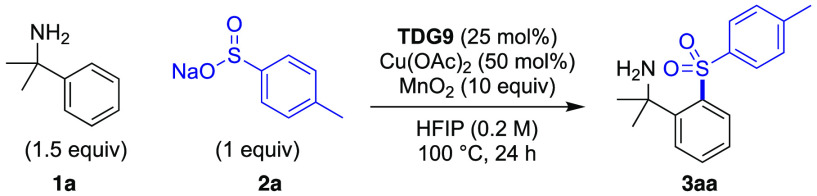
Control Reactions Describing Deviation from Standard Conditions Using Catalytic Copper and TDG^a^

entry	deviation from standard conditions	yield of compound **3aa** (%)^b^
1	none	67 (68)
2	no [Cu]	0
3	no MnO_2_	17
4	no TDG	15
5	using **TDG1**	5
6	using **TDG2**	0
7	using **TDG3**	43
8	K_2_S_2_O_8_ as an oxidant (2–10 equiv)	11–21
9	+ K_2_CO_3_ (2 equiv)	70 (68)
10	+ TEMPO (1 equiv)	0

a Reactions were performed on a 0.2 mmol scale with respect to the sulfinate salt.

b Yields were determined by ^1^H NMR using 1,3,5-trimethoxybenzene as an internal standard. Isolated yields are in parentheses. The starting material is volatile; therefore, recovered starting material was not reliably determined.

The reaction scope varying the sulfinate salt was then investigated (Scheme 2). Compound **3aa** was obtained in a 68% yield, which was readily scaled affording the product in a 61% yield (1.76 g). Aryl sulfinates bearing electron-neutral (H), electron-rich (OMe and *t*Bu), or electron-poor (CF_3_ and halogens) substituents all gave good yields with a slight preference for the electron-poor sulfinate salts (**3ab**–**3ah**). More sterically hindered naphthyl-substituted example **3ai** was less effective. Methyl and cyclopropyl sulfinate salts were both highly effective, affording compounds **3aj** and **3ak** in 88 and 70% yields, respectively. The reaction with the bicyclo[1.1.1]pentane (BCP) sulfinate salt was also successful in generating sulfone **3al**.

**Scheme 2:**
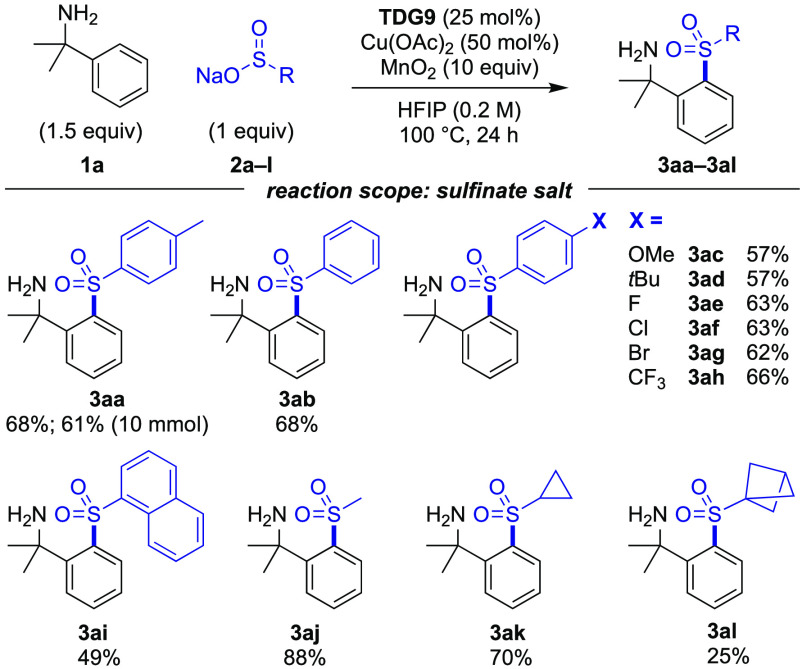
Reaction Scope Varying the Sulfinate Salt Reactions were performed on a 0.2 mmol scale, with isolated yields reported.

Next, the benzylamine component was investigated (Scheme 3). Initially, we varied the substituent in the 3 position (*para* to the C–H bond being functionalized), whereby substrates with electron-rich and electron-poor substituents reacted effectively (**3ba**–**3ea**), with slightly improved yields for the 3-OMe (**3ba**) and 3-F-phenyl (**3ca**) derivatives. Changing the bromo substituent from the 3 to 4 position gave sulfonyl amine **3fa** in a 71% yield. The pentafluorosulfanyl (SF_5_) group is increasingly of interest in medicinal chemistry, and SF_5_-substituted benzylamine was effectively sulfonylated to give amine **3ga** in a 58% yield. The functionalization of the more challenging pyridyl-containing substrate **3ha** was also realized, despite the presence of the additional coordinating moiety.

**Scheme 3:**
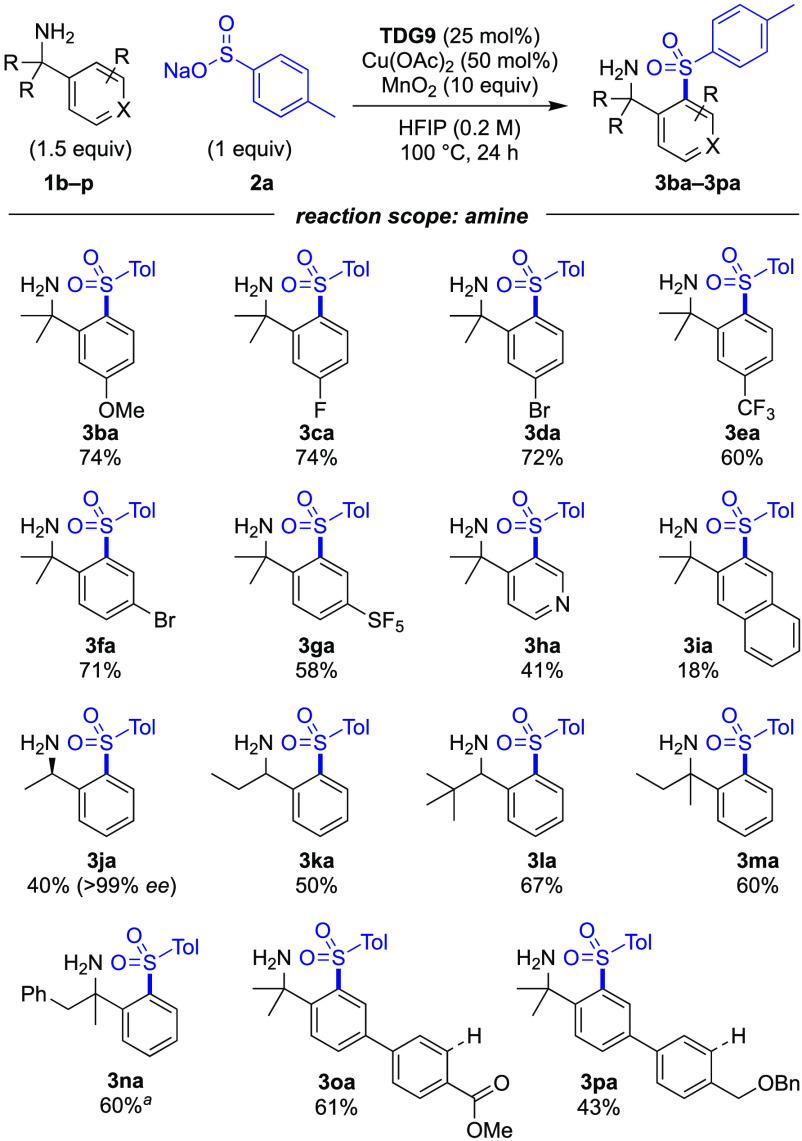
Reaction Scope Varying the Amine Contains 6% inseparable starting material. Reactions were performed on a 0.2 mmol scale, with isolated yields reported.

2-Naphthyl derivative **3ia** was formed with a selective reaction at the 3 position. A range of α-alkylbenzylamines were also converted to the sulfonylated products **3ja**, **3ka**, and **3la** in good yields. Sulfonylation of enantioenriched amine retained the chirality of the starting amine [>99% enantiomeric excess (ee)]. However, unsubstituted benzylamines were unsuccessful. Ethyl- and benzyl-substituted amines were sulfonylated exclusively at the *ortho* position to give sulfones **3ma** and **3na** in a 60% yield. In the benzyl-substituted example, no sulfonylation of the more distal aryl group was observed. Biaryl substrates **3oa** and **3pa** possessing functional groups capable of directing *ortho*-metalation were sulfonylated exclusively at the *ortho* position to the amine without any sulfonylation adjacent to either the ester or the ether functionality. Furthermore, both the methyl ester and benzyl groups remained intact under these conditions. In all cases, only monosulfonylation was observed.

The amine products generated in this transient process were directly available for further derivatization (Scheme 4). To illustrate this, sulfone **3aa** was readily acetylated with acetyl chloride to form amide **4** and was converted to aminooxetane **5** using an oxetane sulfonyl fluoride reagent in a defluorosulfonylative process.^26^ Reductive alkylation and nucleophilic aromatic substitution (S_N_Ar) reactions, as commonly employed in medicinal chemistry programs, were also readily demonstrated to provide alkyl amine **6** and aryl amine **7**.

**Scheme 4:**
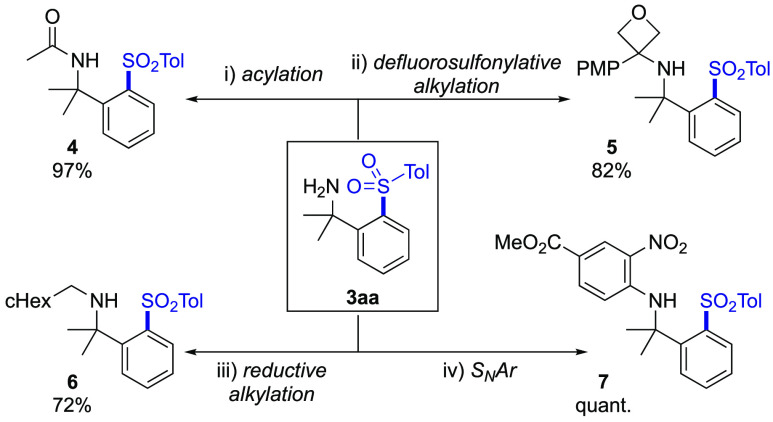
Derivatization of the Amine Functionality Reactions were performed on a 0.20 mmol scale, with isolated yields reported. Reaction conditions: (i) AcCl, NEt_3_, CH_2_Cl_2_, room temperature, 18 h; (ii) 3-(4-methoxyphenyl)-3-oxetanesulfonyl fluoride, K_2_CO_3_, MeCN, 80 °C, 1 h; (iii) cyclohexanecarboxaldehyde, NaBH(OAc)_3_, DCE, room temperature, 24 h; and (iv) methyl 4-fluoro-3-nitrobenzoate, *i*PrOH, 100 °C, 4 h.

To provide insight into the reaction mechanism, a competition kinetic isotope effect (KIE) experiment gave a preferential reaction of the protic substrate [H/D of 3.88]. Similarly, H/D exchange was not observed in the product or recovered starting materials when running the reaction with either a deuterated substrate or the protic substrate in *d*_2_-HFIP.^27^ These results were suggestive of an irreversible C–H functionalization process under the reaction conditions, that is, the turnover-limiting step.

We then investigated elementary steps through density functional theory (DFT) calculations (Figure S1 of the Supporting Information).^28−30,23^ The C–H activation to form a cupracycle was calculated to proceed via a 5-coordinate inner sphere transition state (**TS-3/4**), in which an axial acetate ligand mediates C–H activation, with a barrier of 24.1 kcal mol^–1^. Natural bond orbital (NBO) analysis of the transition state revealed a charge distribution and geometry similar to those of an arenium ion, in addition to a significant stabilizing effect from donation from the C–Cπ system into empty orbitals on Cu. This is indictive of a Wheland-like transition state for C–H activation, consistent with our previous mechanistic studies on the copper-mediated transient C–H functionalization of aldehydes.^31^ Noticeably the energy barrier of the concerted metalation–deprotonation (CMD) step is significantly lowered in the presence of the TDG when compared to the free amine-directed reaction (TDG, +24.1 kcal mol^–1^; free amine, +32.2 kcal mol^–1^; see the Supporting Information). The influence of the TDG is therefore in the provision of improved ligation properties to promote the CMD.

Association of the sulfinyl radical to the copper center occurs in a barrierless process (**Int-5**–**Int-7**; Figure S1 of the Supporting Information).^32^ Oxidation of the sulfinate salt to the radical was calculated to occur readily by a single-electron transfer (SET) process, mediated by copper acetate.^33^ Cyclic voltammetry (CV) studies indicate that the sulfinate salt can be oxidized in the redox window of the reaction (CVs in HFIP versus Fc/Fc^+^: MeSO_2_Na, *E*_pa_ = +1.02 V; TolSO_2_Na, *E*_pa_ = +1.06 V). Reductive elimination forms the C–S bond and a Cu^I^ species, with a barrier of +21.2 kcal mol^–1^.

In summary, C–H sulfonylation of benzylamines has been achieved using both catalytic copper acetate and a catalytic aldehyde TDG to form γ-sulfonyl amines. Earth abundant and cheap manganese dioxide was used as a stoichiometric oxidant and base. Selective reactivity was maintained in the presence of other coordinating functionalities, and the sulfonyl amine products were readily diversified. A significant role of the TDG is to lower the barrier for C–H activation and formation of the cupracycle through an inner sphere CMD step, involving a Wheland-type intermediate.

## Data Availability

Raw and processed characterization data for all novel compounds and Cartesian coordinates from computed structures can be found at the Imperial College London Research Data Repository: 10.14469/hpc/12033. A version of this manuscript was deposited on the preprint repository ChemRxiv.^34^ The data underlying this study are available in the published article and its Supporting Information.

## Supporting Information Available

Optimization reactions, deuteration experiments, KIE experiments, unsuccessful substrates, details of computational studies, and experimental procedures and characterization data (PDF)
