# Supplementary material for: Dual Copper- and Aldehyde-Catalyzed Transient C–H Sulfonylation of Benzylamines
Source: Org Lett. 2023 Jul 13;25(28):5285–90. doi: 10.1021/acs.orglett.3c01783 (PMC10367073; doi:10.1021/acs.orglett.3c01783)
Supplement: Supplementary file 1 — ol3c01783_si_001.pdf [file ol3c01783_si_001.pdf]

SUPPORTING INFORMATION

Dual Copper and Aldehyde Catalyzed Transient C–H Sulfonylation of Benzylamines

Joe I. Higham, Tsz-Kan Ma and James A. Bull*
Department of Chemistry, Imperial College London, Molecular Sciences Research Hub, White City Campus, Wood Lane, London, W12 0BZ, UK

*E-mail: j.bull@imperial.ac.uk

Table of Contents

Contents

Dual Copper and Aldehyde Catalyzed Transient C–H Sulfonylation of Benzylamines........... 1
General Experimental 3
Optimisation of Reaction Conditions for Copper Mediated C–H Sulfonylation 5
Stoichiometric copper conditions 5
Transient directing group 5
Copper source 6
Solvent... 7
Further optimisation 8
Copper catalysed conditions.. 9
Oxidant 9
Copper loading 10
Additional variation to reaction conditions 11
Control reactions of base mediated protocol 12
K.I.E. and deuteration studies 13
Cyclic Voltammetry 16
Summary of computation calculations.. 17
Unsuccessful Substrates.. 26
Copper Catalysed C(sp^2)–H Sulfonylation... 27
General Procedure A: If amine is either solid or a liquid of known density 27
General Procedure B: If amine is a liquid of unknown density 27
Reaction Scope Varying the Sulfinate Salt 28
Reaction Scope Varying the Amine 32
Synthesis of deuterated material 47
Product Derivatization Reactions.. 49
^{1}H and ^{13}C Spectra of Selected Compounds 51
References 129

General Experimental

All reactions were run under an inert atmosphere (argon) with flame-dried glassware using standard techniques unless otherwise stated. Anhydrous solvents were obtained by filtration through drying columns (THF, diethyl ether, CH_2Cl_2, DMF). $Cu(OAc)_2$ (98%, product code: B23615) and anhydrous CuF_2 (99.5%, product code: 11489) were obtained from Alfa Aesar and used as provided. 2-Oxo-1,2-dihydro-3-pyridinecarbaldehyde (95%, product code: 044084) was obtained from Fluorochem and used as provided. Potassium carbonate (99.5%, product code: 024862) was obtained from Fluorochem and used as provided. Liquid commercial aldehydes were distilled prior to use. Solid aldehydes with boiling points >300 °C were dissolved in CH_2Cl_2, washed with 1 M NaOH, dried over Na_2SO_4, filtered, then concentrated *in vacuo*. All other commercial reagents were used as supplied or purified by standard techniques where necessary. All C–H activation reactions were performed in microwave vials sealed with Fisherbrand™ 20 mm aluminium, plain, centre hole, molded septa butyl, dark grey, 55° shore A, 3.0 mm caps if using high boiling solvents (>100 °C). Fisherbrand™ 20mm Crimp Seal, Gold, Magnetic Cap, 8mm Center hole, assembled septum, molded septa butyl, dark grey, 55° shore A, 3.0 mm caps were used with lower boiling solvents (<100 °C) if heating significantly above their boiling point.

Flash column chromatography was performed using 230-400 mesh silica with the indicated solvent system according to standard techniques. Analytical thin-layer chromatography (TLC) was performed on precoated, glass-backed silica gel plates. Visualisation of the developed chromatogram was performed by UV absorbance (254 nm), aqueous potassium permanganate, *p*-anisaldehyde, phosphomolybdic acid or vanillin stains. Infrared spectra (ν_{max}, FTIR ATR) were recorded in reciprocal centimeters (cm^{-1}). Nuclear magnetic resonance (NMR) spectra were recorded on 400 MHz spectrometers. Chemical shifts for ^1H NMR spectra are recorded in parts per million from tetramethylsilane with the solvent resonance as the internal standard (chloroform δ = 7.27 ppm). Data is reported as follows: chemical shift [multiplicity (s = singlet, d = doublet, t = triplet, q = quartet, pent = pentet, m = multiplet and b = broad), coupling constant in Hz, integration, assignment]. ^{13}C NMR spectra were recorded with complete proton decoupling. Chemical shifts are reported in parts per million from tetramethylsilane with the solvent resonance as the internal standard (chloroform: δ = 77.00 ppm). *J* values are reported in Hz. Assignments of $^1H/^{13}C$ spectra were made by the analysis of δ/J values, and COSY, HSQC, and HMBC experiments as appropriate. ^{19}F NMR spectra were recorded without complete proton decoupling unless otherwise stated. ^{19}F NMR spectra are indirectly referenced to $CFCl_3$ automatically via direct measurement of the absolute frequency of the deuterium lock signal by the spectrometer hardware. Melting points are uncorrected.

The high-resolution mass spectrometry (HRMS) analyses were performed using electrospray ion source (ESI) or pneumatically assisted atmospheric pressure chemical ionization (APCI) using an atmospheric solids analysis probe (ASAP). ESI was performed using a Waters LCT Premier equipped with an ESI source operated in positive or negative ion mode. The software used was MassLynx 4.1. This software does not account for the electron and all the calibrations/references are calculated accordingly, i.e. $[M+H]^+$ is detected and the mass is calibrated to output [M+H]. APCI was performed using an Orbitrap XL or Xevo G2S using an ASAP to insert samples into the APCI source. The sample was introduced at ambient temperature and the temperature increased until the sample vaporised.

Computational methods

DFT calculations were run using Gaussian 16 (Revision C.01).[1] The reaction free energies were calculated using the hybrid exchange-correlation ωB97X-D functional, which includes D2 dispersion corrections described by Grimme.[2,3] A functional screening was undertaken on key transition states for all pathways, with the surveyed functionals including B3LYP[4,5] with Grimme's D3 dispersion correction including Becke-Johnson damping (GD3BJ),[6,7] M06[8] with Grimme's D3 dispersion correction (GD3)[9] and PBE0[10,11] with GD3BJ.[6,7] NBO analysis was performed using NBO 6.0.[12]

The split valence 6-31+g(d,p) basis sets were used for carbon and hydrogen. This lower basis set was chosen as these elements do not bind directly to either catalytic metal center, but extra diffuse functions were added to capture more mid- and long-range interactions. The triple-ζ 6-311+g(d) basis set was used for all heteroatoms. 6-31++G(d,3pd) was used to describe the H atom being deprotonated in the C–H activation step and the H atom bound to the TDG. Copper centers were described with the Stuttgart SDD pseudopotential.

Geometry optimisation calculations were performed without symmetry constraints and using an "ultrafine" grid for numerical integration. All structures are optimized using the self-consistent reaction field (SCRF) approach with conductor-like polarisable continuum model (CPCM). As the required solvent information for hexafluoroisopropanol is not available on gaussian 16, it was modelled implicitly using a CPCM model using the built-in parameters of Trifluoroethanol, modified with the polar parameter of hexafluoroisopropanol (ε=16.7) ([SCRF=(cpcm,solvent=2,2,2-trifluoroethanol),read] and [eps=16.7].

All intermediates and transition states were characterised by normal coordinate analysis revealing either precisely zero or one imaginary frequency, respectively. In the case of transition states, the imaginary frequency corresponds to the mode of the intended reaction step. The connection between each transition state and intermediates either side was verified with forwards and backwards intrinsic reaction coordinate (IRC) calculations. Full coordinates for all the calculated stationary points are included as part of the supplementary information (.xyz).

Full coordinates for all structures, together with computed energies and vibrational frequency data, are available via the corresponding Gaussian 16 output files and calculation spreadsheet, stored in the open-access digital repository detailed below.

All Data for this manuscript can be found at the Imperial College London Research Data Repository: DOI: 10.14469/hpc/12033

Optimisation of Reaction Conditions for Copper Mediated C–H Sulfonylation

Stoichiometric copper conditions

Initial optimisation of the reaction conditions used conditions stoichiometric in copper adapted from our previous work functionalising benzaldehydes.[13]

Transient directing group

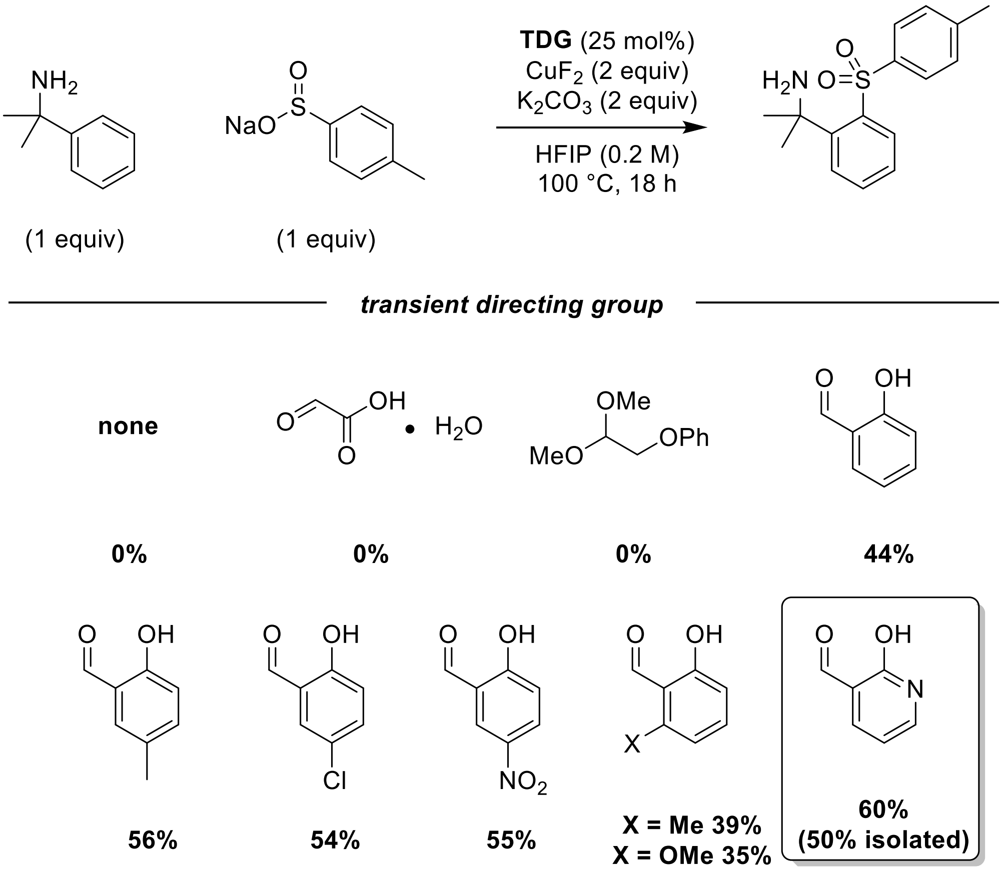

Scheme S1 – Optimisation of the transient directing group

Copper source

Under stoichiometric conditions, CuF_2 was confirmed as the most effective copper source (Table S1).

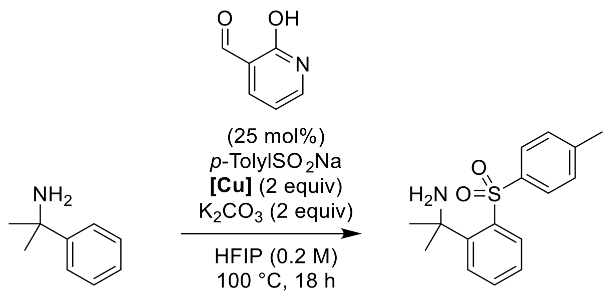

Entry	[Cu]	Yield 3aa (%)[a]
**1**	**CuF_2**	**60(50)**
2	$Cu(OAc)_2$	56
3	$CuSO_4$ $5H_2O$	0
4	$CuCl_2$	43
6	CuO	0
7	$Cu(OTf)_2$	45

Table S1 – Optimisation varying the copper source. Reactions performed on 0.2 mmol scale with respect to the sulfinate salt. [a]Yield determined by ^1H NMR using 1,3,5-trimethoxybenzene as an internal standard.

Solvent

All solvents aside from HFIP were ineffective (Table S2).

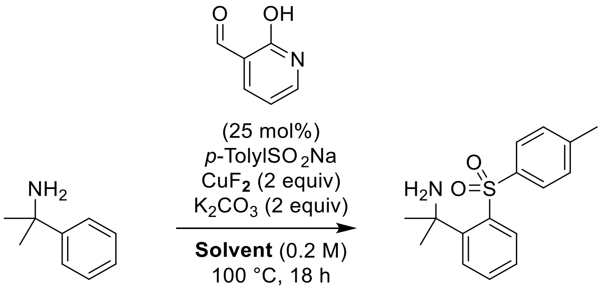

Entry	Solvent	Yield 3aa (%)
**1**	**HFIP**	**60(50)**
2	AcOH	0
3	MeOH	0
4	MeCN	0
6	TFE	0
7	DMSO	0

Table S2 – Optimisation varying the Solvent. Reactions performed on 0.2 mmol scale with respect to the sulfinate salt. [a]Yield determined by ^{1}H NMR using 1,3,5-trimethoxybenzene as an internal standard.

Further optimisation

Further optimisation was carried out varying several parameters (Table S3). Increased loading of sulfinate was detrimental (Entry 2) and under stoichiometric conditions increased amount of amine has a negligible effect (Entry 3). Raising or lowering the loading of the TDG led to decreased yields (Entries 4–6) as did changing the concentration of the reaction (Entries 7 and 8). A comparable yield of sulfonyl amine was observed when using the potassium sufinate in place of the sodium sulfinate (Entry 9).

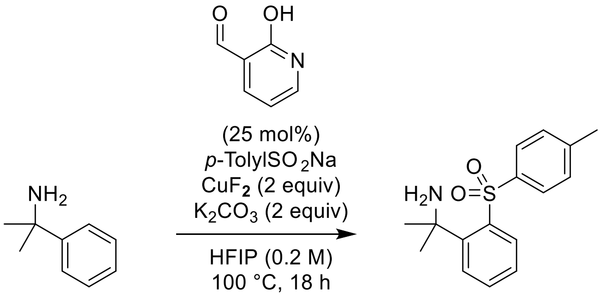

Entry	Change to above conditions	Yield 3aa (%)
**1**	**none**	**60(50)**
2	1.5 equiv sulfinate salt	10
3	2 equiv amine	62
4	50 mol% TDG	48
5	10 mol% TDG	53
6	5 mol% TDG	21
7	0.1 M	33
8	0.2 M	43
9	KSO_2*p*-tolyl instead of $NaSO_2$*p*-tolyl	59

Table S3 – Further optimization of Cu mediated conditions. Reactions performed on 0.2 mmol scale with respect to the sulfinate salt. [a]Yield determined by ^{1}H NMR using 1,3,5-trimethoxybenzene as an internal standard.

Copper catalysed conditions

Oxidant

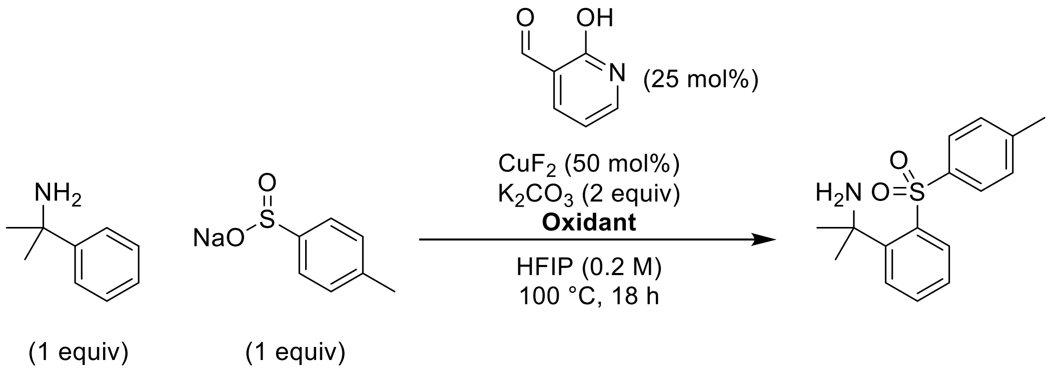

Entry	Oxidant	**3aa** (%)[a]
1	None	8
2	$K_2S_2O_8$ (2 equiv)	9
3	$K_2S_2O_8$ (3 equiv)	10
4	MnO_2 (2 or 5 equiv)	22
**5**	**MnO_2 (10 equiv)**	**59**

Table S4 – Optimisation of oxidant for copper catalysed conditions. Reactions performed on 0.2 mmol scale with respect to the sulfinate salt. [a]Yield determined by ^{1}H NMR using 1,3,5-trimethoxybenzene as an internal standard.

Copper loading

Reduced loading of copper had a detrimental effect on the yield (Table S5).

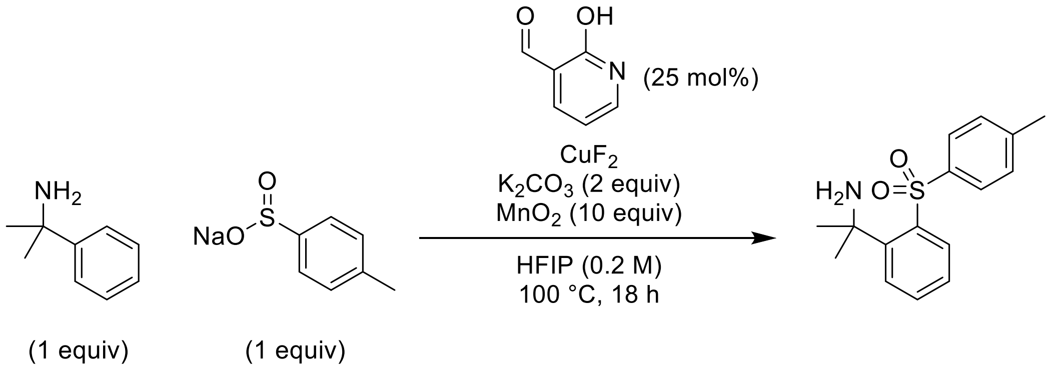

Entry	CuF_2 Loading	**3aa** (%)[a]
**1**	**50 mol%**	**59**
2	25 mol%	33
3	10 mol%	Trace
4	No Cu	NR

Table S5 – Optimisation varying copper loading. Reactions performed on 0.2 mmol scale with respect to the sulfinate salt. [a]Yield determined by ^{1}H NMR using 1,3,5-trimethoxybenzene as an internal standard.

Copper source

Re-evaluation of the copper source highlighted copper acetate as more effective (Table S6, Entry 3).

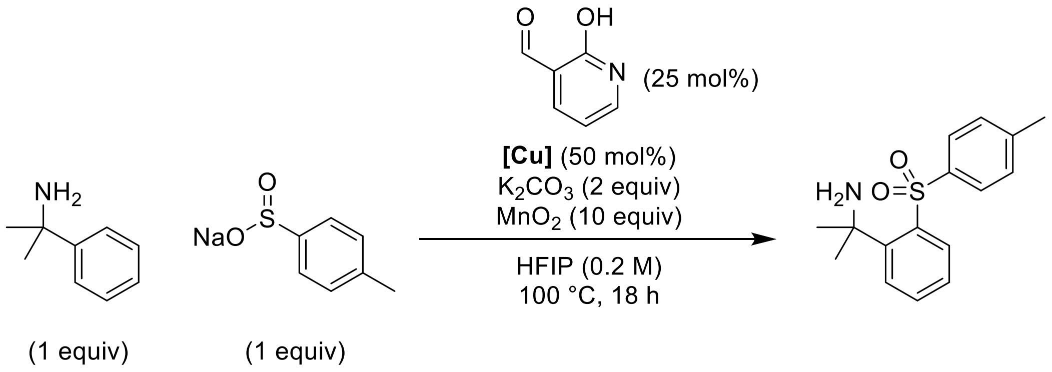

Entry	[Cu]	**3aa** (%)[a]
1	CuF_2	59
2	CuF_2 dihydrate	56
**3**	**$Cu(OAc)_2$**	**61**
4	$CuCl_2$	47

Table S6 – Optimization varying copper salt. Reactions performed on 0.2 mmol scale with respect to the sulfinate salt. [a]Yield determined by ^{1}H NMR using 1,3,5-trimethoxybenzene as an internal standard.

Additional variation to reaction conditions

Further alteration of reaction conditions found further increasing the amount of manganese(IV) oxide was not beneficial (Table S7, Entry 2), and increasing the reaction time was of no benefit (Entry 3). Increasing the loading of [Cu] back to stoichiometric quantities gave the exact same yield as when 50 mol% was used (Entry 4). Increasing the amine loading under catalytic conditions yielded a positive effect when >2 equiv amine was used at 18 h, with 70% yield observed when 2 equiv of amine was used (Entries 5–7). By using 1.5 equiv amine and increasing the time to 24 h led to a comparable yield as 2 equiv, 18 h (Entry 8).

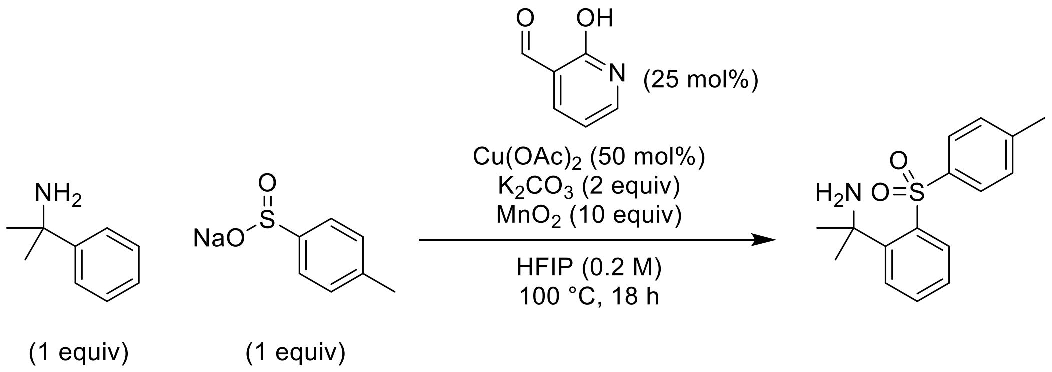

Entry	Change to above conditions	Yield 3aa (%)
1	none	60
2	20 equiv MnO_2	56
3	72 h	61
4	1 equiv $Cu(OAc)_2$	60
5	1.5 equiv amine	58
6	2 equiv amine	70
7	3 equiv amine	65
**8**	**1.5 equiv amine, 24 h**	**70 (68)**

Table S7 – Further optimization of reaction conditions. Reactions performed on 0.2 mmol scale with respect to the sulfinate salt. [a]Yield determined by ^1H NMR using 1,3,5-trimethoxybenzene as an internal standard.

Control reactions of base mediated protocol

Finally, control reaction were carried out (Table S8). No reaction was observed in the absence of Copper (Entry 2), and only trace amounts of product were present in the absence of the TDG (Entry 3). Unexpectedly the reaction progressed as effectively without any additional base (Entry 4), thus the base was omitted in the optimised conditions. Without MnO_2 the reaction still progressed to just under 1 turnover of the copper catalyst (Entry 5).

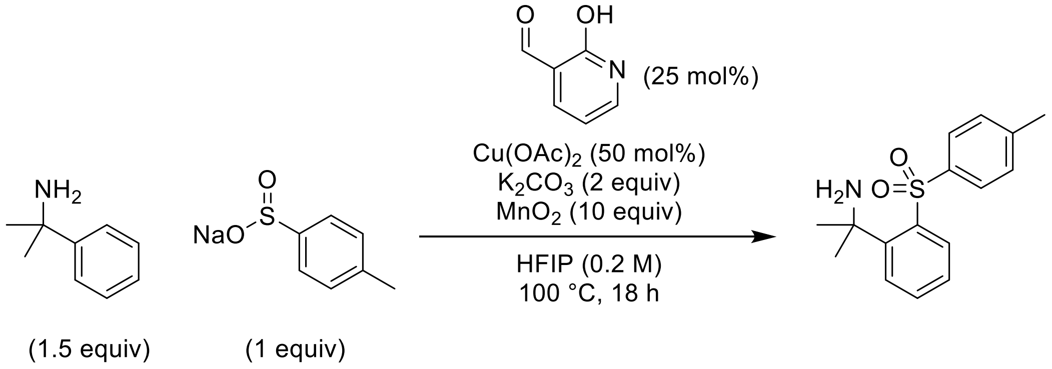

Entry	Change to Conditions	Yield 3aa (%)
1	None	70 (68)
2	No Cu	0
3	No TDG	trace
**4**	**No K_2CO_3**	**67(68)**
5	No MnO_2	19

Table S8 – Control reactions of K_2CO_3 mediated protocol. Reactions performed on 0.2 mmol scale with respect to the sulfinate salt. [a]Yield determined by ^{1}H NMR using 1,3,5-trimethoxybenzene as an internal standard.

K.I.E. and deuteration studies

To identify if the CMD step is turnover limiting, and the degree of reversibility of this system, experiments with **cumylamine-d₈** were carried out. A competition K.I.E. experiment was carried out, revealing a K.I.E. of 3.88 when comparing the relative amounts of the proteo and deutro product by using the benzylic methyl signal. This primary K.I.E. is consistent with our prior work on the analogous aldehyde system, where a large K.I.E. was observed, implying a turnover limiting C–H activation step.

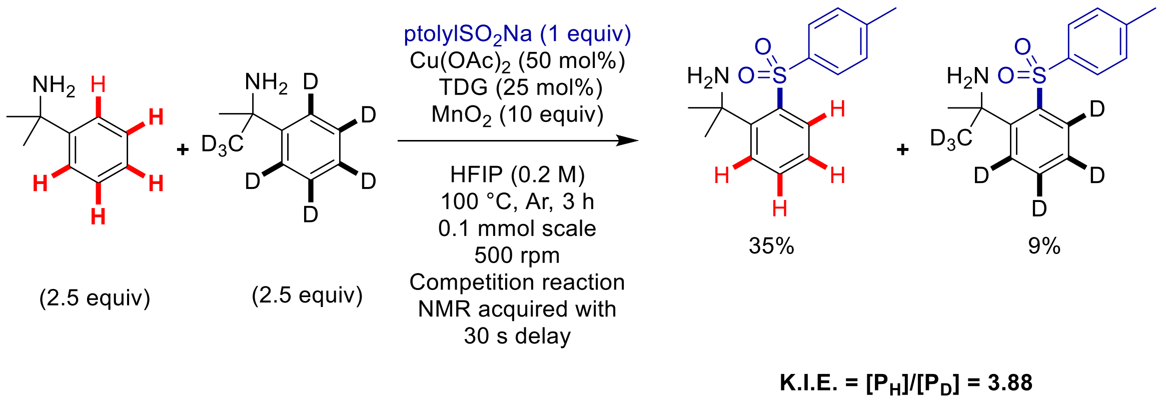

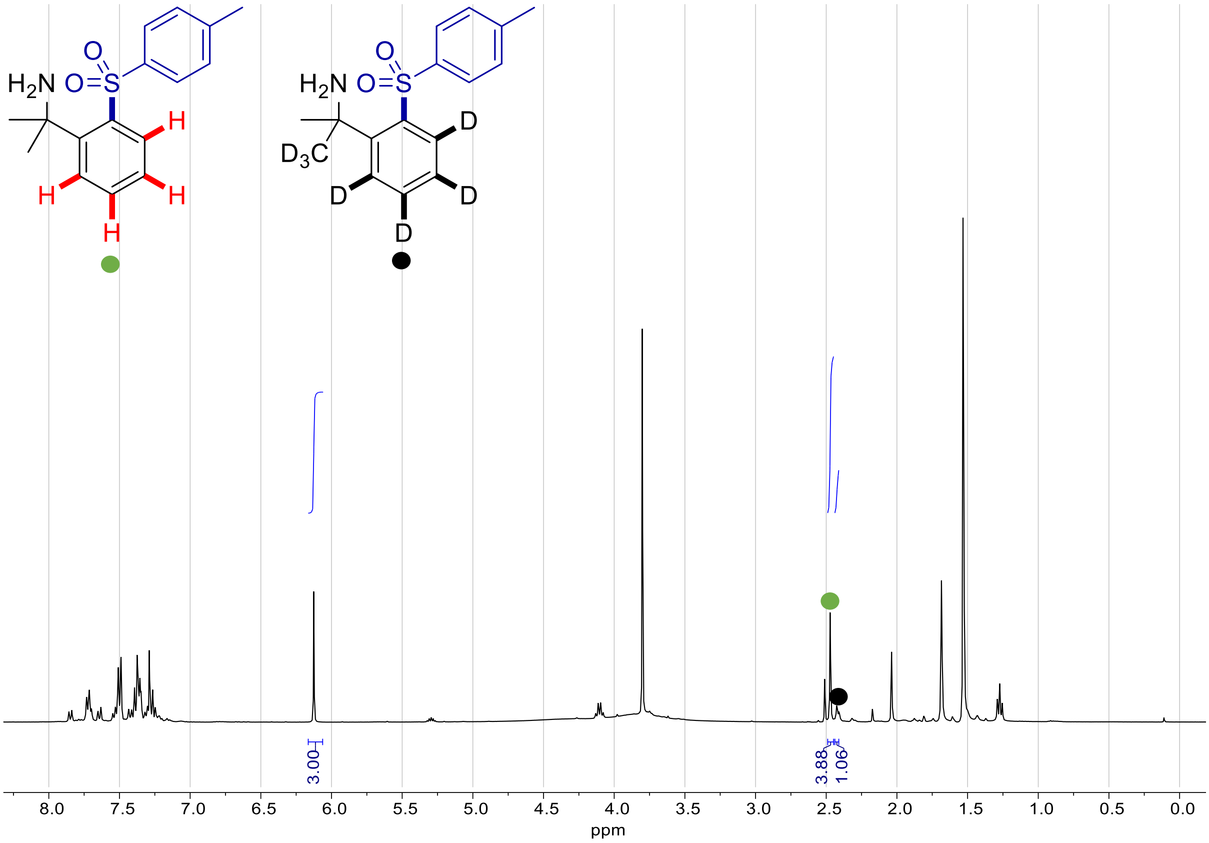

Scheme S2 – Competition K.I.E. experiment

In addition to K.I.E. experiments, further studies into the reversibility of the C–H activation was carried out. Initial experiments focused on determining if there was protonation of cumylamine-d₈ in the reaction conditions in the absence of the sulfinate salt (Scheme S3). Evidence of H/D exchange was observed (appearance of ^{1}H signal at δ 7.46 ppm) when the TDG was present, supporting a reversible C–H activation mechanism. Protonation of Cumylamine-d₈ was not observed without the TDG present.

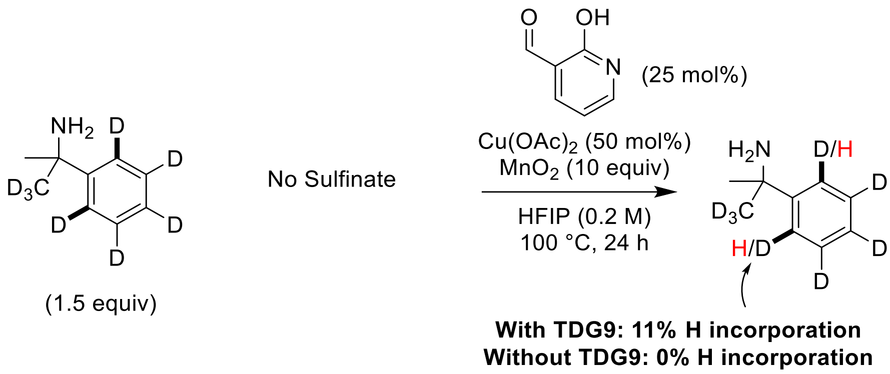

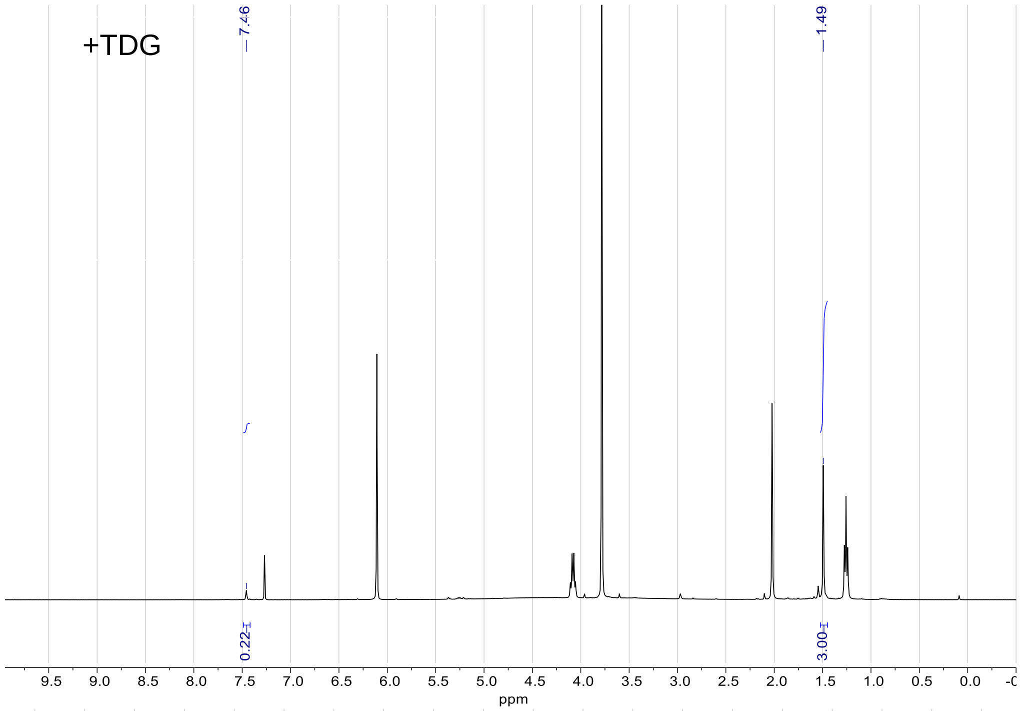

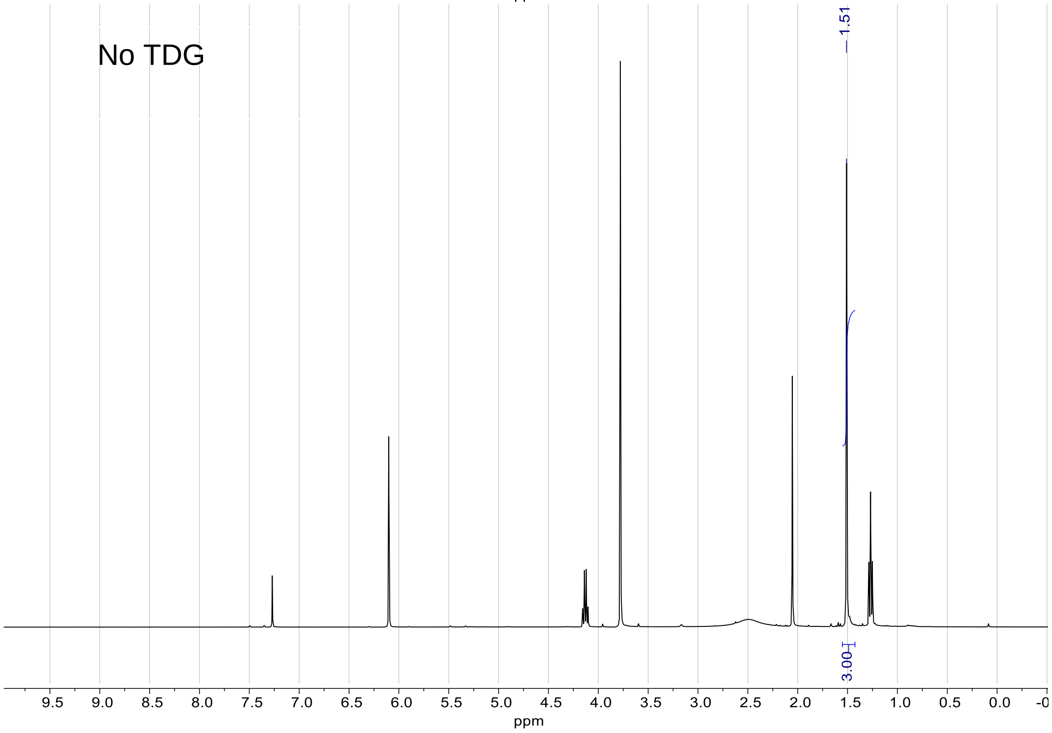

Scheme S3 – H,D exchange with and without TDG

The deuteration of amine **1f** in HFIP-d_2 was also carried out in the presence and absence of the TDG. This signal corresponding to the Hs *ortho* to the benzylamine moiety show reduced signal integration in both cases, corresponding to approximately 16% D incorporation with the TDG present, consistent with the earlier protonation experiment, and around 8% D incorporation in the absence of the TDG. Both NMRs were acquired with a 30 s delay to ensure any disparity in signal integration was a result of deuteration and not relaxation time.

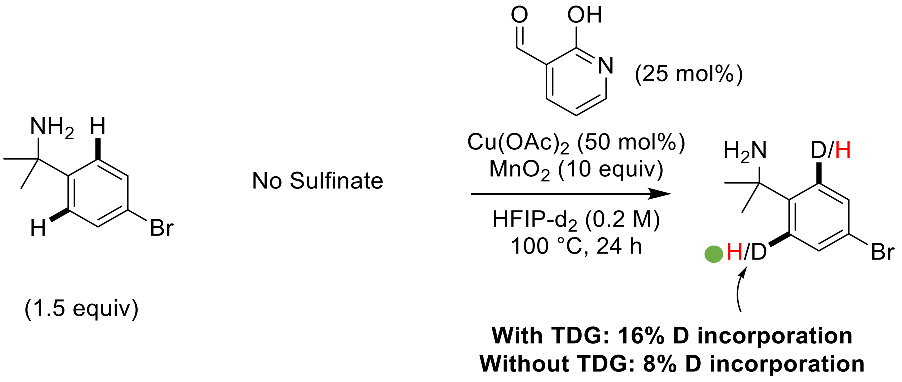

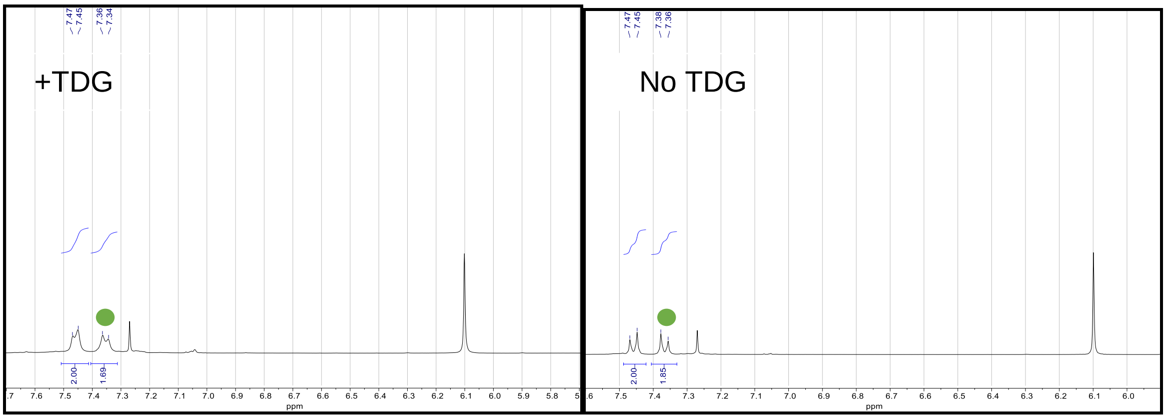

Scheme S4 – Deuteration of amine in HFIP-d_2

Cyclic Voltammetry

General Procedure. The cyclic voltammogram (IUPAC convention) of the sulfinate salts were recorded at room temperature with a Metrohm Autolab PGSTAT204 workstation. Analysis was performed with the Nova 2.0 software. A three electrodes undivided electrochemical cell was used. A glassy carbon disc-electrode (3 mm-diameter) was used as the working electrode, with Pt sheet as the counter electrode and Ag rod as pseudo-reference electrode. The glassy carbon electrode was polished on a polishing pad in a water-alumina slurry with figure-eight motions and rinsed with deionised water and acetone before each measurement. The solution of interest was sparged with Ar for 2 min before data collection. Scan direction: positive direction from 0 V. Ferrocene was used as an internal standard after each measurement and all potentials were referred against the ferrocenium/ferrocene redox couple. Current was reported in μA. Applied voltage was corrected with positive feedback iR compensation.

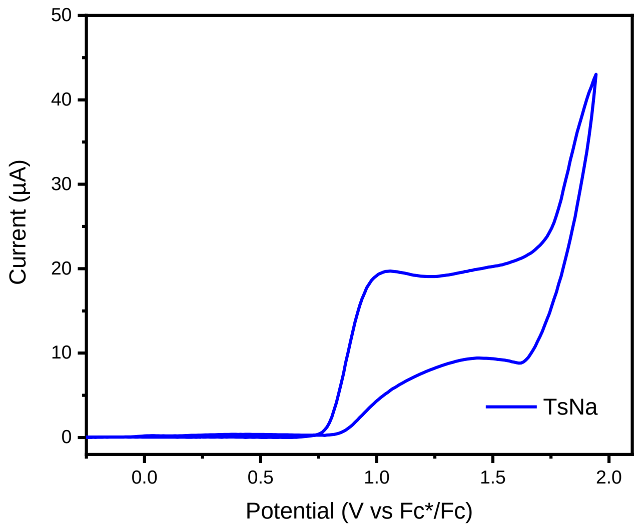

Figure S1 - Cyclic voltammogram of sodium p-toluenesulfinate (10 mM). Conditions: *n*-Bu_4NPF_6 (0.10 M in HFIP). Purged with Ar. Scan rate: 100 mVs^{-1}. Scan direction: Positive direction from 0 V.

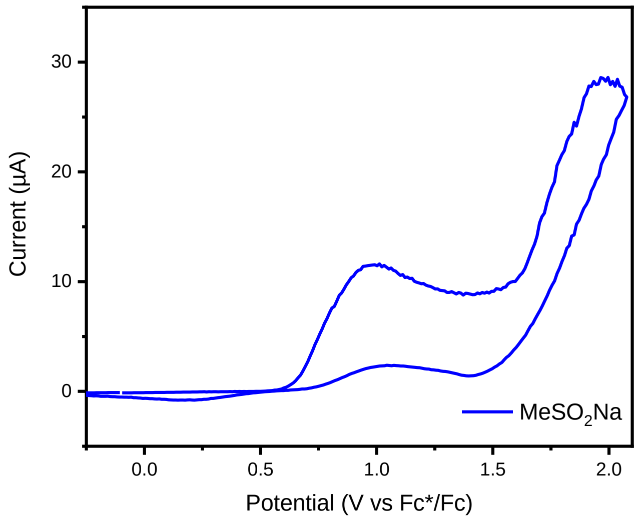

Figure S2 - Cyclic voltammogram of sodium methanesulfinate (10 mM). Conditions: *n*-Bu_4NPF_6 (0.10 M in HFIP). Purged with Ar. Scan rate: 100 mVs^{-1}. Scan direction: Positive direction from 0 V.

Summary of computation calculations

DFT calculations were performed using gaussian 16 to better understand the mechanism of this process. A single turnover of the copper catalyst was considered, as the exact Mn(IV) species responsible for reoxidation was unclear and the exact mechanism of oxidation by manganese is poorly understood.

<u>C–H activation</u>: Given the competitive K.I.E. experiment was indicative of a turnover limiting C–H activation step, the possible pathways of this were first evaluated (Scheme S5). Imine formation is predicated to be feasible, and reversible, with only a slight change in free energy (+0.5 kcal/mol) on imine condensation. Subsequent coordination to copper is highly exergonic leading to a shared intermediate **Int-3** which is lowest energy point for both CMD pathways. At this point, in the pathway proceeding through a more common 4 coordinate CMD transition state (Scheme S5a), loss of acetic acid and leads to **TS-S1/5** resulting in an energy barrier of +27.2 kcal/mol. Subsequently cupracycle **Int-5** is formed, in which the overall C–H activation is slightly endergonic (+10 kcal/mol). An alternative pathway involving a 5 coordinate CMD transition state was found to have a lower energy barrier (Scheme S5b). The same intermediary copper bound imine **Int-3** is formed, however instead of loss of acetic acid, a CMD process directly from this intermediate through **TS-3/4** is indicated to be energetically feasible and possesses a lower energy barrier for this process (+24.1 kcal/mol). Subsequently, there is formation of cupracycle **Int-4** with an extra acetic acid ligand bound, which after loss of HOAc leads to the shared cupracyclic intermediate **Int-5**. The slightly endergonic C–H activation process implies a reversible CH activation step, and deuteration studies show H/D exchange in the reaction at the *ortho* position, supporting the predictions of these calculations.

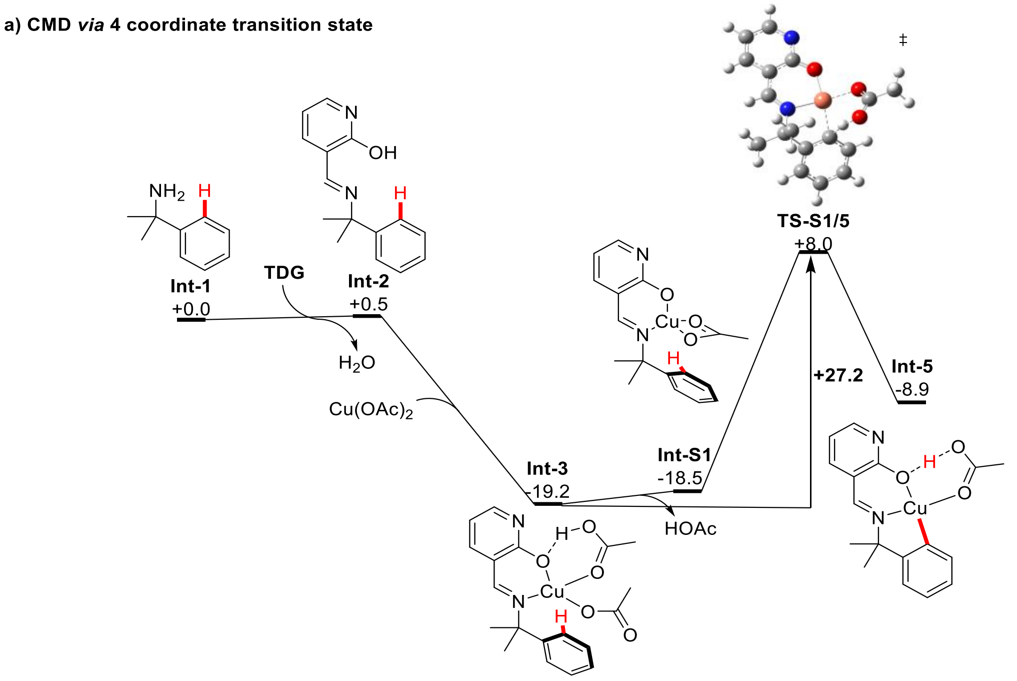

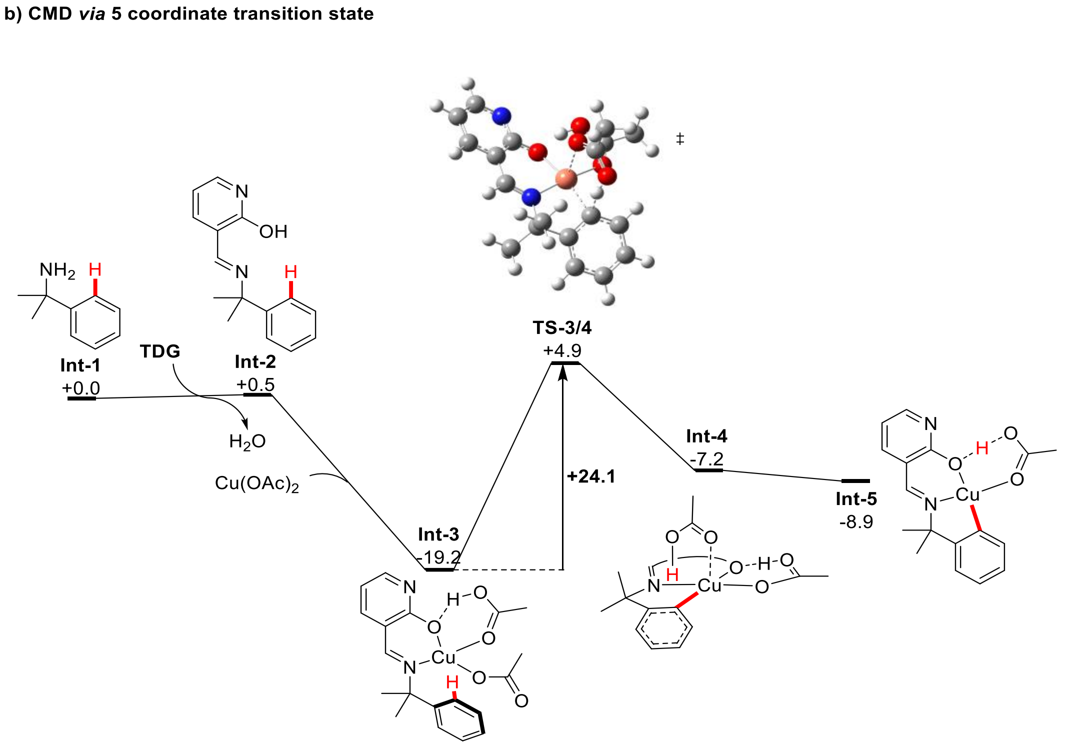

Scheme S5 – Calculated pathways for the TDG mediated C–H activation of cumylamine

Sulfinate activation: The next calculations focused on the subsequent steps to install the C–S bond (Scheme S6). First the pathway to access **Int-7** was calculated, either by a sulfinyl radical (Green) or ionic (Blue) pathway. CV studies indicate the sulfinate salt can be oxidized in the redox window of the reaction (CVs in HFIP vs Fc/Fc*: $MeSO_2Na$, E_{pa} = +1.02V; $TolSO_2Na$ E_{pa} = +1.06V). The blue sulfinate coordination pathway involves first coordination of the sulfinate to the copper centre, followed by oxidation by another equivalent of copper(II) acetate. An alternate pathway is the direct oxidation of the sulfinate salt to the sulfonyl radical, followed by a radical mediated oxidation event where the radical couples to the copper center to give **Int-7**. Both pathways are feasible, and the energy difference between them is negligible indicating either pathway could be in operation, or potentially both. Regardless, both lead to high valent copper intermediate **Int-7** which is a key intermediate for the reductive elimination to the sulfonylated product.

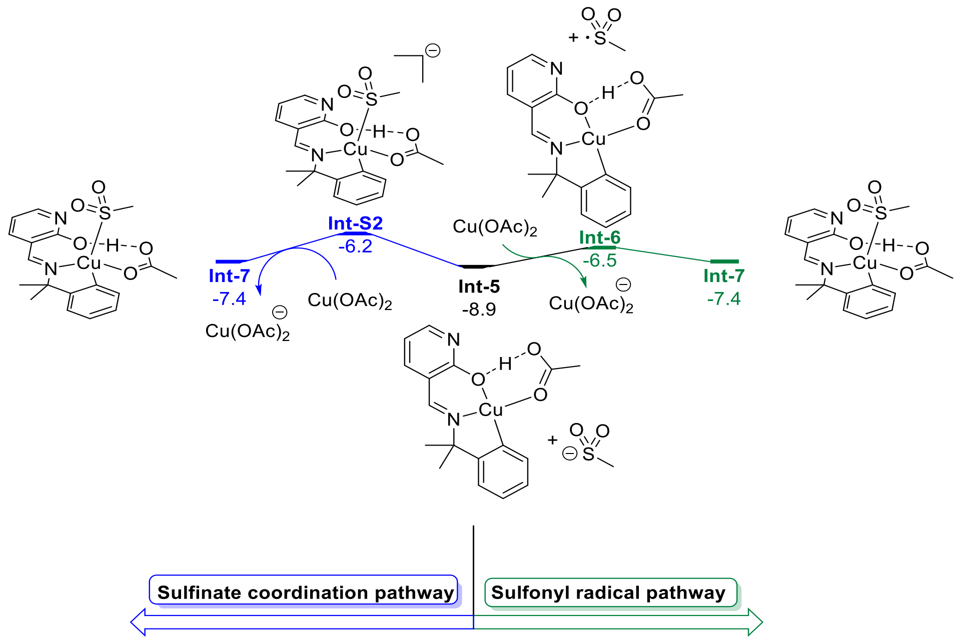

Scheme S6 – Potential oxidation pathways.

Reductive elimination and hydrolysis: this oxidation step, **Int-7** undergoes reductive elimination *via* **TS-7/8**, in process predicted to be exergonic (ΔG_{RE} = -20.3) and irreversible. Once the C–S bond is formed, the ligated copper(I) species can be decoordinated by another equivalent of SM amine coordinating, leading to formation of a copper(I) amine complex in a slightly endergonic step. Additionally, the product imine **Int-9** can be hydrolysed to release the product amine and regenerate the TDG.

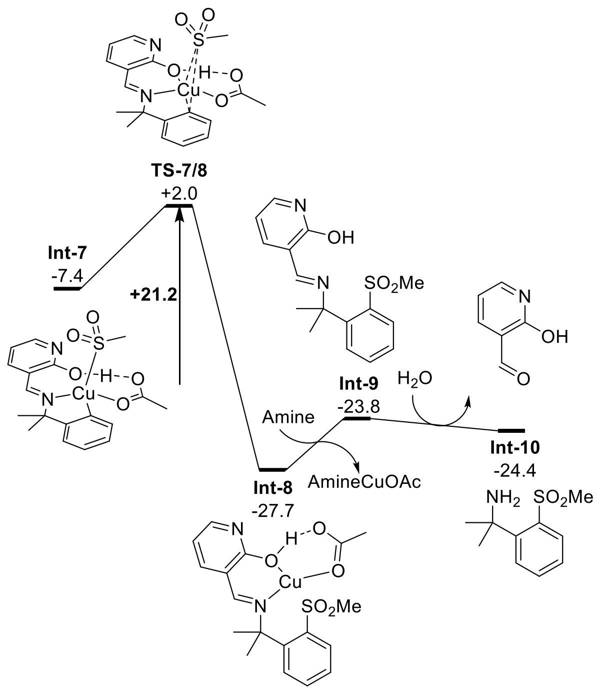

Scheme S7 – Pathway for reductive elimination followed by copper decoordination and imine hydrolysis

NBO analysis of CMD Transition state

In previous work investigating the analogous C–H sulfonylation reaction of aldehyde,[13] the CMD process found to proceed *via* a transition state reminiscent of a Wheland intermediate, with a build up of positive charge on the aromatic ring. We propose the 5 coordinate CMD transition state for the amine functionalisation to also share similarities with the Wheland-like intermediate proposed in our previous work. To this end, the 5 coordinate CMD transition state was explored by NBO analysis and its structure compared to benzene and the benzenium ion as a model for formal Wheland intermediate. First the change in charge was investigated from the pre-C–H activation intermediate **Int-4** to cupracycle **Int-5** *via* **TS-4/5**. NBO analysis indicated a subtle build up of positive charge at C(1), C(3) and C(5) (+0.027, +0.052 and +0.031 respectively), with a significant build up of negative charge at C(2) (-0.296) and to a lesser extent on the copper center itself (-0.061). This is similar to the extreme case of the benzenium ion formation by protonation of benzene, whereby positive charge build-up is observed at C(1), C(3) and C(5) (albeit more significant due to the structure changing from neutral to +1. Additionally, there is a build up of negative charge at C(2), consistent with charge indicated by NBO analysis of the CMD transition state **TS-4/5.**

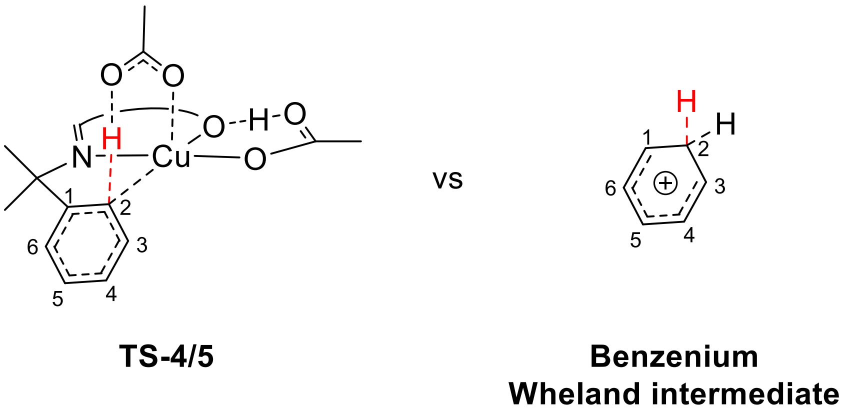

Atom	Change in charge	
	**TS-4/5**	**Benzenium**
C(1)	+0.027	+0.271
C(2)	-0.296	-0.374
C(3)	+0.052	+0.271
C(4)	-0.01	-0.046
C(5)	+0.031	+0.266
C(6)	-0.011	-0.046
Cu	-0.061	-

Table S9 – Change in relative charge for the formation of transition state **TS-4/5** compared to the formation of benzenium ion.

In addition to investigating electronic changes, the fundamental geometrical similarities of **TS-4/5** to benzenium ion were also investigated. In the extreme case for the benzenium Wheland intermediate, a H–C–X bond angle of 100.6° and a H–C–C–H dihedral angle of 54.7° were calculated. **TS-4/5** was calculated to possess a H–C–X bond angle of 72.3° and a dihedral angle of 38.7°. Clearly **TS-4/5,** while not identical, does possess geometric similarities to benzenium.

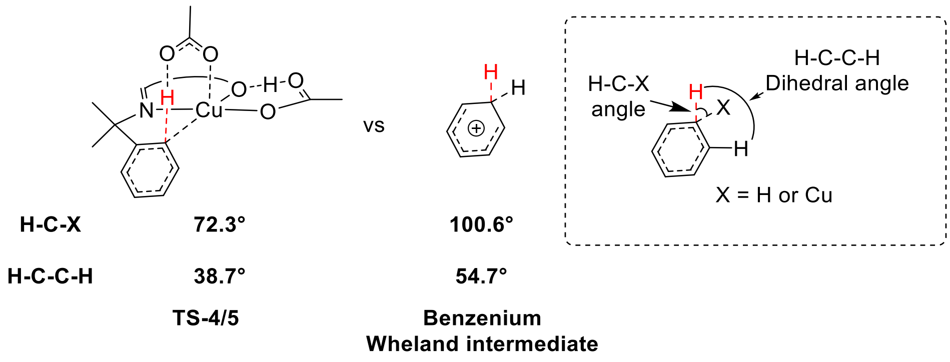

Scheme S8 – Structural comparison of **TS-4/5** and benzenium.

During the transition state **TS-4/5** there is significant C–Cu bond order present, with more than 50% of the C–Cu bond formed at this stage. There is also significant cleavage of the C–H bond, with approximately 50% of the C–H bond cleave in the transition state **TS-4/5**.

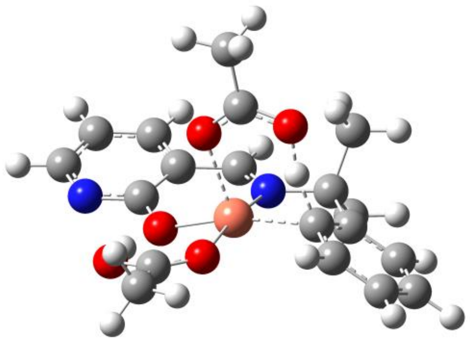

Bond	Covalent Bond Order		
	preTS	TS	postTS
**C–Cu**	-	0.257	0.478
**C–H**	0.907	0.464	-

Scheme S9 – Bond order pre, during and post **TS-4/5**

The C–Cu interaction was also characterised by NBO analysis to determine the specific interactions contributing to the C(2)-Cu bond in this transition state. The donation of the C–Hσ bond into the copper was a clear and significant interaction indicated by the NBO analysis, with this interaction stabilising the transition state by 64.5 kcal/mol. A secondary stabilisation effect was found from the C–Cπ system donating to unoccupied orbitals on copper which provides a significant contribution to the C–Cu interaction (18.9 kcal/mol).

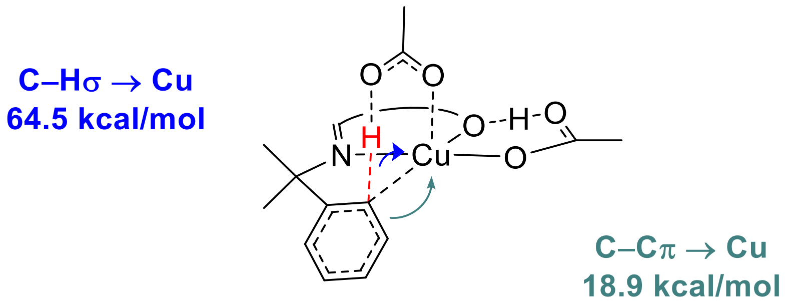

Scheme S10 – Summary of stabilising interaction between C(2) and Cu.

Overall the changes in electronics, similar geometry and participation of the aromatic in **TS-4/5** support the proposed Wheland-like transition state. The aromatic character of the aryl ring remains intact however, reflecting the transition state's partial Wheland-like character.

Summary of Computational Pathway

A full summary of calculations of the most likely pathway are summarised below (Figure S1). Imine formation between **Int-1** and 2-hydroxynicotinaldehyde was slightly endergonic, followed by an exergonic complexation with copper acetate to give **Int-3** as the lowest energy intermediate prior to the turnover limiting C–H activation step. The C–H activation is calculated to proceed *via* a 5-coordinate inner sphere transition state (**TS-3/4**) in which an axial acetate ligand mediates C–H activation, with a barrier of 24.1 $kcalmol^{-1}$. This CMD process leads to the formation of cupracycle **Int-4** which is converted to **Int-5** by release of acetic acid.

Oxidation of the sulfinate salt is calculated to occur readily by an SET process, mediated by copper acetate. CV studies indicate the sulfinate salt can be oxidized in the redox window of the reaction (CVs in HFIP vs Fc/Fc*: $MeSO_2Na$, E_{pa} = +1.02V; $TolSO_2Na$ E_{pa} = +1.06V). Association of the sulfinyl radical to the copper center occurs a barrierless process converting **Int-5** to **Int-7**.[30] Reductive elimination *via* **TS-7/8** forms the C–S bond and a Cu^{I} species, with a barrier of +21.2 kcal mol^{-1}. The de-coordination of copper from **Int-8** is slightly uphill in energy, releasing the product imine which can then be hydrolyzed to produce the product sulfonyl amine **Int-10**.

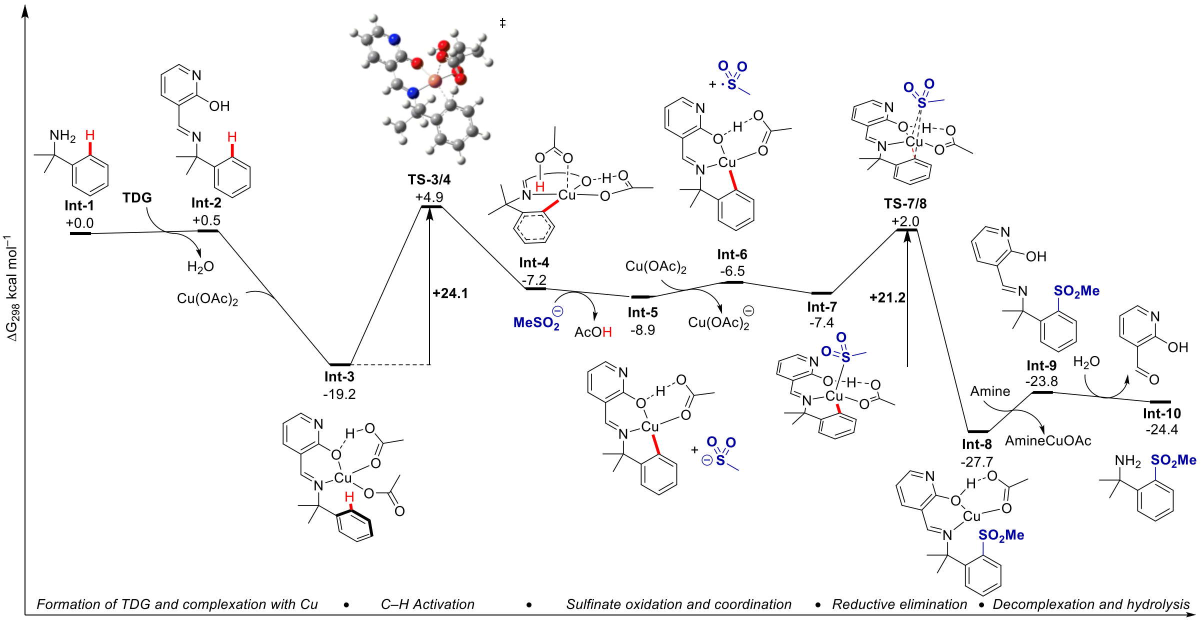

Figure S1 – Calculated free energy profile for imine directed C–H sulfonylation of cumylamine via CMD. Dotted lines included to show bonds involved in the transition states.

Free amine directed C–H sulfonylation

In addition to calculating the reaction pathway with the TDG, the pathway for the free amine directed process was also calculated to gain an idea of which step(s) energy barriers are changed by the presence of the TDG to gain insight into its in the reaction. The lowest energy point in this pathway is **Int-S3** in which two amine SM molecules are bound, and one must decoordinate to allow access to **Int-S4**. A CMD *via* **TS-S4/S5** was found to possess a higher energy barrier than in the TDG pathway, and due to the lack of a bidentate directing group it was not possible to find a transition state *via* a 5 coordinate pathway. In addition to the C–H activation step possessing a higher energy barrier, the energy barrier of the reductive elimination is higher in this free amine directed process (free amine: +27.2 kcal/mol, TDG directed: +21.2 kcal/mol). Overall, the energies of the intermediates and transition states are higher without the assistance of the TDG. The key effect of the TDG is to allow access to a 5 coordinate CMD transition state which leads to the lower energy barrier of C–H activation.

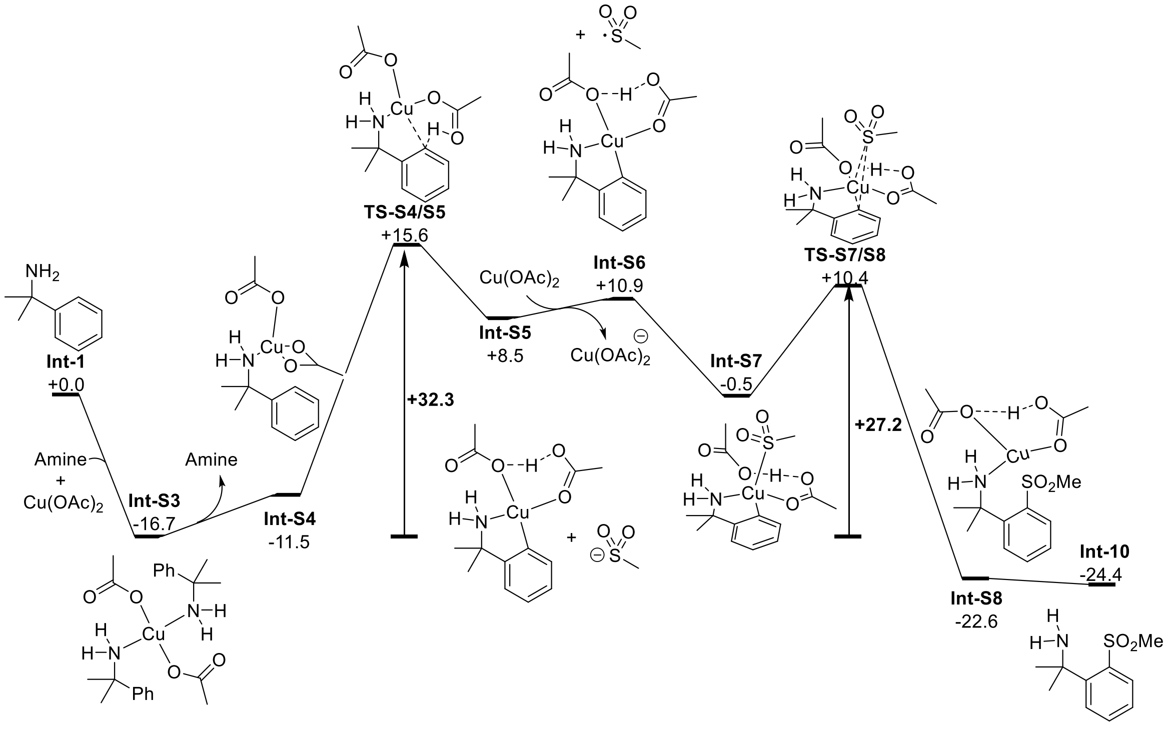

Scheme S11 – Calculated pathway for the free amine directed C–H sulfonylation

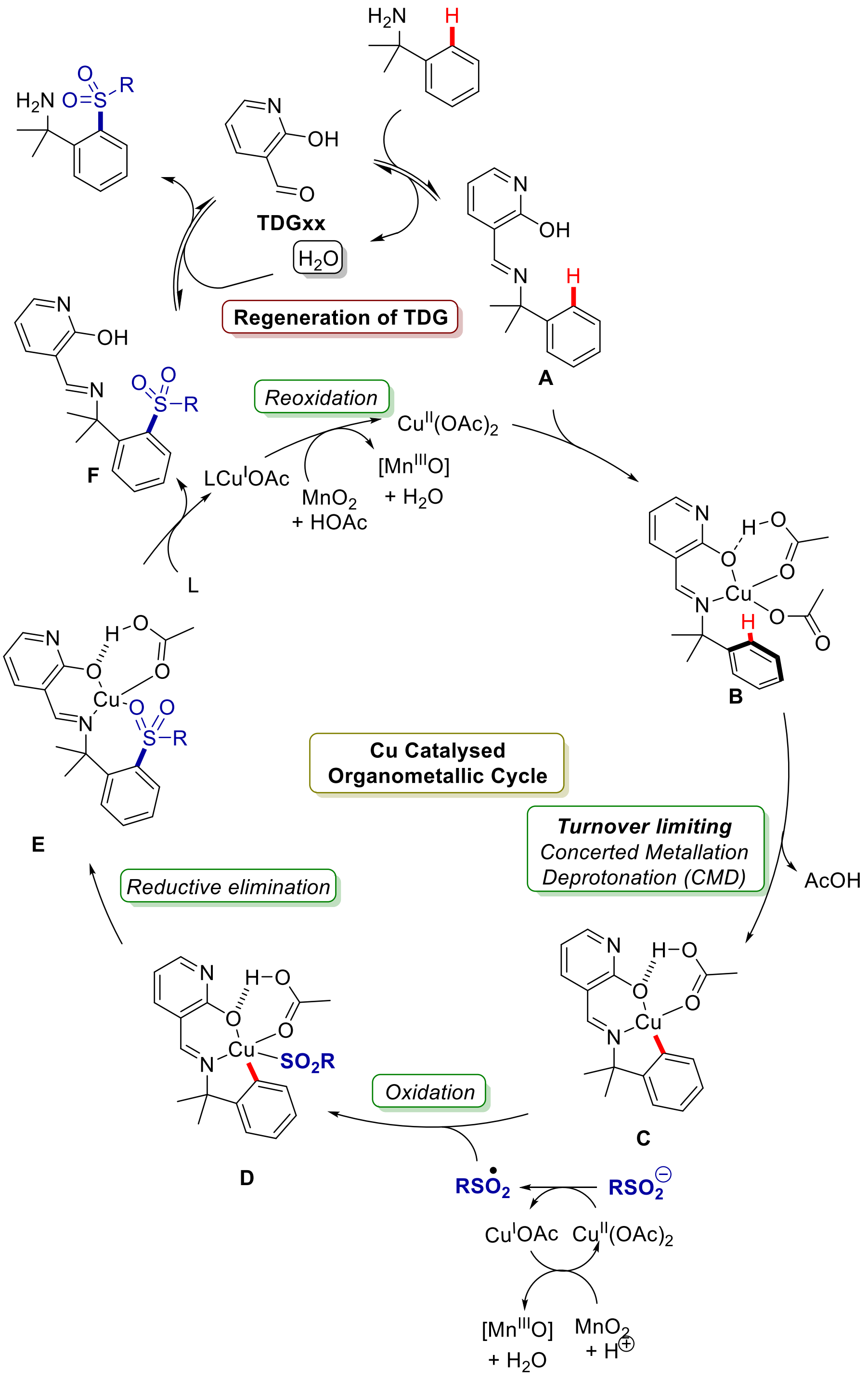

Scheme S12 – Proposed mechanism

Unsuccessful Substrates

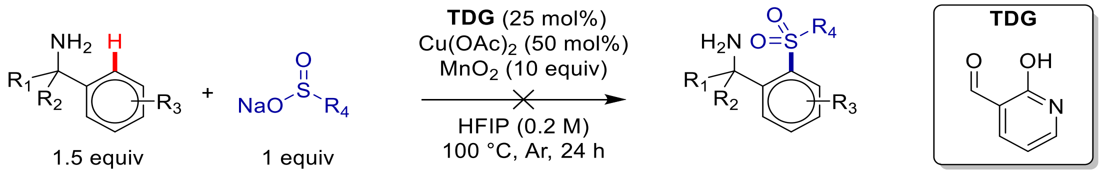

Oxidatively sensitive substrates

C(sp^3)–H sulfonylation

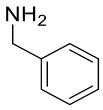

Complex Mixture

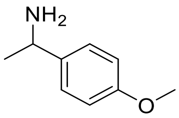

Complex Mixture

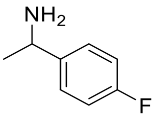

Complex mixture

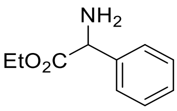

Complex Mixture

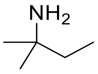

C(sp^3)–H bond unreactive

Copper Catalysed C(sp^2)–H Sulfonylation

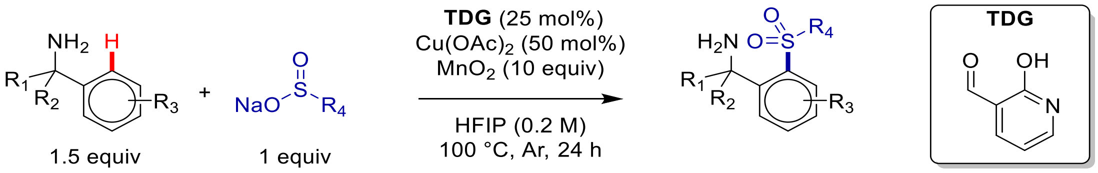

General Procedure A: If amine is either solid or a liquid of known density

Copper(II) acetate (18.1 mg, 0.1 mmol) were added to a microwave vial which was then flame dried under argon (ca. 2–5 s). The microwave vial was allowed to cool to room temperature and 2-hydroxynicotinaldehyde (6.2 mg, 0.05 mmol), sulfinic acid sodium salt (0.2 mmol), manganese(IV) oxide (174 mg, 2 mmol) and the amine (0.3 mmol) were added to a microwave vial sequentially under argon, sealed and HFIP (1 mL, 0.2 M) was added and the vial was submerged in a preheated oil bath to 100 °C for 24 h [Stirring rate set to 500 rpm]. The reaction was allowed to cool to room temperature, diluted with aqueous 1 M HCl (2 mL) and transferred to a separating funnel, washing out the microwave vial with EtOAc (3 × 5 mL). the aqueous 1M NaOH (5 mL) was added [note pH should be between 10 and 14] and the product was extracted from the aqueous phase with EtOAc (3 × 10 mL).[Note: the excess MnO_2 causes the aqueous phase to be extremely dark, and can obscure the phase boundary, brine can be added to aid phase separation.] The combined organic extracts were dried over Na_2SO_4, filtered and concentrated *in vacuo*.

General Procedure B: If amine is a liquid of unknown density

Copper(II) acetate (18.1 mg, 0.1 mmol) were added to a microwave vial which was then flame dried under argon. The microwave vial was allowed to cool to room temperature and 2-hydroxynicotinaldehyde (6.2 mg, 0.05 mmol), sulfinic acid sodium salt (0.2 mmol), manganese(IV) oxide (174 mg, 2 mmol) were added to a microwave vial sequentially under argon, sealed and solution of amine in HFIP (0.3 mmol in 1 mL HFIP) was added and the vial was submerged in a preheated oil bath to 100 °C for 24 h [Stirring rate set to 500 rpm]. The reaction was allowed to cool to room temperature, diluted with aqueous 1 M HCl (2 mL) and transferred to a separating funnel, washing out the microwave vial with EtOAc (3 × 5 mL). the aqueous 1M NaOH (5 mL) was added [note pH should be between 10 and 14] and the product was extracted from the aqueous phase with EtOAc (3 × 10 mL). [Note: the excess MnO_2 causes the aqueous phase to be extremely dark, and can obscure the phase boundary, brine can be added to aid phase separation.] The combined organic extracts were dried over Na_2SO_4, filtered and concentrated *in vacuo*.

Reaction Scope Varying the Sulfinate Salt

2-(2-(4-Methylbenzenesulfonyl)phenyl)propan-2-amine (3aa)

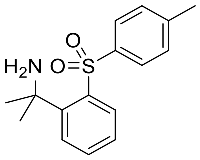

Prepared according to general procedure **A** using cumylamine (43.2 μL, 0.3 mmol) and 4-methylbenzene sulfinic acid sodium salt (35.6 mg, 0.2 mmol). Sulfonyl amine **3aa** was isolated by flash column chromatography (100% EtOAc to 10% MeOH:EtOAc) as a yellow oil (39.5 mg, 68%). R_f 0.17 (10% MeOH:EtOAc). IR (film)/cm^{-1} 3392 (br, weak, N–H), 3056, 2967, 1595, 1461, 1290, 1150, 910, 813. ^{1}H NMR (400 MHz, $CDCl_3$) δ 7.94 (dd, *J* = 8.1, 1.5 Hz, 1H, Ar–CH), 7.72 (d, *J* = 8.4 Hz, 2H, 2 × Ar–CH), 7.63 (dd, *J* = 8.1, 1.5 Hz, 1H, Ar–CH), 7.53 – 7.46 (m, 1H, Ar–CH), 7.35 – 7.28 (m, 3H, 3 × Ar–CH), 2.46 (brs, 2H, NH_2), 2.42 (s, 3H, $ArCH_3$), 1.67 (s, 6H, $C(CH_3)_2$). ^{1}H NMR (400 MHz, DMSO-d_6) δ 7.98 (dd, *J* = 8.1, 1.5 Hz, 1H, Ar–CH), 7.75 (dd, *J* = 8.0, 1.3 Hz, 1H, Ar–CH), 7.67 (d, *J* = 8.2 Hz, 2H, 2 × Ar–CH), 7.61 (td, *J* = 7.6, 1.5 Hz, 1H, Ar–CH), 7.48 – 7.40 (m, 1H, Ar–CH), 7.38 (d, *J* = 8.2 Hz, 2H, 2 × Ar–CH), 2.37 (s, 3H, $ArCH_3$), 1.55 (s, 6H, $C(CH_3)_2$). ^{13}C NMR (101 MHz, DMSO-d_6) δ 143.1 (Ar–C_q), 140.8 (Ar–C_q), 139.2 (Ar–C_q), 133.2 (Ar–CH), 132.6 (Ar–CH), 129.6 (3 × Ar–CH), 128.8 (Ar–C_q), 126.9 (Ar–CH), 126.5 (2 × Ar–CH), 53.9 (C_qNH_2), 33.1* ($C(CH_3)_2$), 21.0 (CH_3). HRMS (TOF–ESI^+) m/z calcd. For $C_{16}H_{20}NO_2S$ [M+H]: 290.1215; found: 290.1212. *Assigned by HSQC and HMBC analysis.

Procedure for Multi Gram Synthesis of Sulfonyl Amine 3aa

$Cu(OAc)_2$ (908 mg, 5 mmol) was added to a Schlenk flask and then flame-dried under Ar (ca. 5 seconds) and was allowed to cool to room temperature. 2-Hydroxynicotinaldehyde (308 mg, 2.5 mmol), 4-methylbenzene sulfinic acid sodium salt (1.78 g, 10 mmol), MnO_2 (8.69 g, 100 mmol), cumylamine (2.03 g, 15 mmol) and HFIP (50 mL) were added sequentially. The resulting mixture was purged with Ar (ca 1 min) before being stirred at 100 °C in a preheated oil bath for 24 h [Stirring rate set to 800 rpm]. The reaction mixture was allowed to cool and filtered through celite and rinsed with EtOAc (200 mL). The filtrate was concentrated to give a green oil. The reaction mixture was diluted with EtOAc (100 mL) and aqueous HCl (50 mL; 1 M) and then basified with NaOH (100 mL; 1 M) before the phases were separated. The aqueous layer was extracted with EtOAc (2 x 100 mL) and the combined organic layers were dried over Na_2SO_4, filtered, and concentrated to give the crude reaction mixture as a brown oil. The crude mixture was purified by column chromatography (EtOAc:MeOH 0% to 10%) to give the product amine as a yellow oil (1.76 g, 6.08 mmol, 61%).

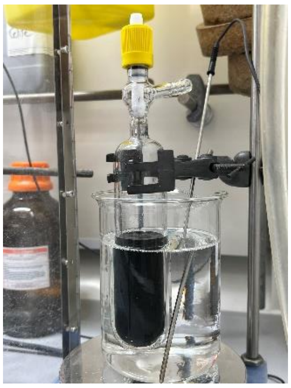

Reaction setup

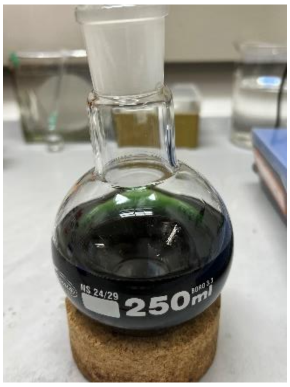

After celite filtration

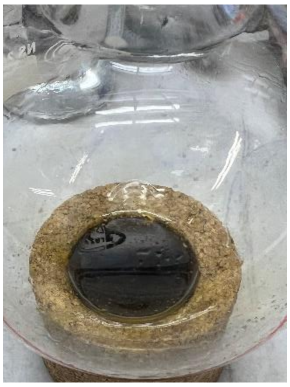

Crude product

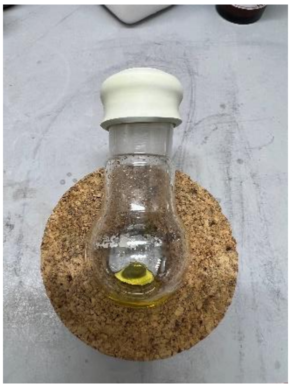

Isolated product

2-(2-(Benzenesulfonyl)phenyl)propan-2-amine (3ab)

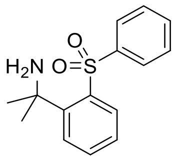

Prepared according to general procedure **A** using cumylamine (43.2 μL, 0.3 mmol) and benzene sulfinic acid sodium salt (32.8 mg, 0.2 mmol). Sulfonyl amine **3ab** was isolated by flash column chromatography (100% EtOAc to 10% MeOH:EtOAc) as a yellow oil (37.7 mg, 68%). R_f 0.22 (10% MeOH:EtOAc). IR (film)/cm^{-1} 3393 (br, weak, N–H), 3060, 2967, 1654, 1470, 1445, 1292, 1146, 1087, 746, 712, 688, 589. ^{1}H NMR (400 MHz, $CDCl_3$) δ 8.04 (dd, *J* = 8.1, 1.5 Hz, 1H, Ar–CH), 7.84 – 7.79 (m, 2H, 2 × Ar–CH), 7.63 (dd, *J* = 8.1, 1.3 Hz, 1H, Ar–CH), 7.58 – 7.45 (m, 4H, 4 × Ar–CH), 7.34 (ddd, *J* = 8.4, 7.2, 1.4 Hz, 1H, Ar–CH), 2.35 (brs, 2H, NH_2), 1.66 (s, 6H, $C(CH_3)_2$). ^{13}C NMR (101 MHz, $CDCl_3$) δ 151.7 (Ar–C_q), 143.9 (Ar–C_q), 139.1 (Ar–C_q), 133.04 (Ar–CH), 133.00 (Ar–CH), 132.4 (Ar–CH), 128.9 (2 × Ar–CH), 128.7 (Ar–CH), 126.61 (Ar–CH), 126.6 (2 × Ar–CH), 54.2 (C_qNH_2), 33.5 ($C(CH_3)_2$). HRMS (ESI$^+$) m/z calcd. For $C_{15}H_{18}NO_2S$ [M+H]: 276.1053; found: 276.1066.

2-(2-(4-Methoxybenzenesulfonyl)phenyl)propan-2-amine (3ac)

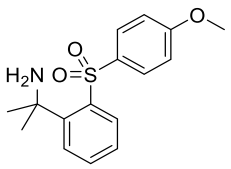

Prepared according to general procedure **A** using cumylamine (43.2 μL, 0.3 mmol) and 4-methoxybenzene sulfinic acid sodium salt (38.7 mg, 0.2 mmol). Sulfonyl amine **3ac** was isolated by flash column chromatography (100% EtOAc) as a yellow oil (34.6 mg, 57%). R_f 0.17 (5% MeOH:EtOAc). IR (film)/cm^{-1} 3400 (br, weak, N–H), 2964, 2841, 1592, 1496, 1462, 1438, 1289, 1257, 1110, 1087, 1023, 830, 800, 762, 672, 552, 443, 413. ^{1}H NMR (400 MHz, $CDCl_3$) δ 7.86 (d, *J* = 8.2 Hz, 1H. Ar–CH), 7.63 (d, *J* = 8.2 Hz, 1H, Ar–CH), 7.47 (dd, *J* = 7.6, 7.6 Hz, 1H, Ar–CH), 7.32–7.24 (m, 2H, 2 × Ar–CH), 6.98 (d, *J* = 8.2 Hz, 2H, 2 × Ar–CH), 3.87 (s, 3H, CH_3), 2.35 (s, 2H, NH_2), 1.68 (s, 6H, $C(CH_3)_2$). ^{13}C NMR (101 MHz, $CDCl_3$) δ 162.9 (Ar–C_q), 151.3 (Ar–C_q), 140.2 (Ar–C_q), 134.6 (Ar–C_q), 132.6 (Ar–CH), 132.4 (Ar–CH), 129.4 (2 × Ar–CH), 128.6 (Ar–CH), 126.5 (Ar–CH), 114.2 (2 × Ar–CH), 55.6 (OCH_3), 54.2 (C_q), 33.4 ($C(CH_3)_2$). HRMS (FTMS+pAPCI) m/z calcd. For $C_{16}H_{20}NO_3S$ [M+H]: 306.1158; found: 306.1160.

2-(2-(4-*tert*-Butylbenzenesulfonyl)phenyl)propan-2-amine (3ad)

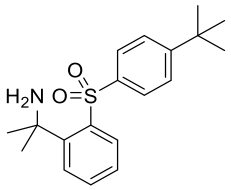

Prepared according to general procedure **A** using cumylamine (43.2 μL, 0.3 mmol) and 4-*tert*butylbenzene sulfinic acid sodium salt (44.1 mg, 0.2 mmol). Sulfonyl amine **3ad** was isolated by flash column chromatography (100% EtOAc) as a yellow oil (37.7 mg, 57%). R_f 0.15 (10% MeOH:EtOAc). IR (film)/cm^{-1} 3400 (br, weak, N–H), 2962, 2869, 1592, 1464, 1364, 1290, 1231, 1197, 1149, 1106, 908, 835, 757, 665, 619, 589, 548. ^{1}H NMR (400 MHz, $CDCl_3$) δ 8.00 (d, *J* = 7.8 Hz, 1H, Ar–CH), 7.77 (d, *J* = 8.7 Hz, 2H, 2 × Ar–CH), 7.66 (d, *J* = 7.8 Hz, 1H, Ar–CH), 7.54–7.50 (m, 3H, 3 × Ar–CH), 7.35 (dd, *J* = 7.8, 7.8 Hz, 1H, Ar–CH), 2.57 (s, 2H, NH_2), 1.70 (s, 6H, $C(CH_3)_2$), 1.35 (s, 9H, $C(CH_3)_3$). ^{13}C NMR (101 MHz, $CDCl_3$) δ 156.5 (Ar–C_q), 151.3 (Ar–C_q), 140.3 (Ar–C_q), 139.6 (Ar–C_q), 132.9 (2 × Ar–CH), 128.6 (Ar–CH), 126.74 (2 × Ar–CH), 126.69 (Ar–CH), 126.0 (2 × Ar–CH), 54.2 ($H_2NC(CH_3)_2$), 35.2 ($C(CH_3)_3$), 33.3 ($H_2NC(CH_3)_2$), 31.0 ($C(CH_3)_3$).

2-(2-(4-Fluorobenzenesulfonyl)phenyl)propan-2-amine (3ae)

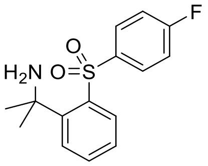

Prepared according to general procedure **A** using cumylamine (43.2 μL, 0.3 mmol) and 4-fluorobenzene sulfinic acid sodium salt (36.6 mg, 0.2 mmol). Sulfonyl amine **3ae** was isolated by flash column chromatography (100% EtOAc) as a colourless oil (37.1 mg, 63%). R_f 0.10 (EtOAc). IR (film)/cm^{-1} 3397 (br, weak, N–H), 3103, 3074, 2970, 1589, 1493, 1468, 1288, 1233, 1147, 1087, 835, 743, 676, 586, 550. ^{1}H NMR (400 MHz, $CDCl_3$) δ 8.03 (dd, *J* = 8.1, 1.5 Hz, 1H, Ar–CH), 7.88–7.80 (m, 2H, 2 × Ar–CH), 7.63 (dd, *J* = 8.1, 1.5 Hz, 1H, Ar–CH), 7.56–7.48 (m, 1H, Ar–CH), 7.35 (ddd, *J* = 8.5, 7.3, 1.5 Hz, 1H, Ar–CH), 7.16 (dd, *J* = 8.5, 8.5 Hz, 2H, 2 × Ar–CH), 2.10 (s, 2H, NH_2), 1.66 (s, 6H, $C(CH_3)_2$). ^{13}C NMR (101 MHz, $CDCl_3$) δ 164.8 (d, *J* = 254.9 Hz, Ar–C_q), 151.8 (Ar–C_q), 140.0 (d, *J* = 3.5 Hz, Ar–C_q), 139.2 (Ar–C_q), 133.1 (Ar–CH), 132.9 (Ar–CH), 129.4 (d, *J* = 9.5 Hz, 2 × Ar–CH), 128.7 (Ar–CH), 126.7 (Ar–CH), 116.1 (d, *J* = 22.5 Hz, 2 × Ar–CH), 54.1 (NH_2*C*$(CH_3)_2$), 33.7 ($NH_2C($*C*$H_3)_2$). ^{19}F NMR (377 MHz, $CDCl_3$) δ -105.48. HRMS (FTMS+pAPCI) m/z calcd. For $C_{15}H_{17}FNO_2S$ [M+H]: 294.0959; found: 294.0958.

2-(2-(4-Chlorobenzenesulfonyl)phenyl)propan-2-amine (3af)

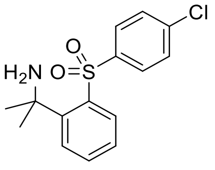

Prepared according to general procedure **A** using cumylamine (43.2 μL, 0.3 mmol) and 4-chlorobenzene sulfinic acid sodium salt (39.8 mg, 0.2 mmol). Sulfonyl amine **3af** was isolated by flash column chromatography (100% EtOAc) as a brown oil (38.9 mg, 63%). R_f 0.25 (10% MeOH:EtOAc). IR (film)/cm^{-1} 3394 (br, weak, N–H), 3086, 2968, 1580, 1475, 1389, 1300, 1149, 1088, 1011, 824, 734, 666, 639, 620, 759, 613. ^{1}H NMR (400 MHz, $CDCl_3$) δ 8.07 (dd, *J* = 8.1, 1.5 Hz, 1H, Ar–CH), 7.78 – 7.71 (m, 2H, 2 × Ar–CH), 7.62 (dd, *J* = 8.1, 1.5 Hz, 1H, Ar–CH), 7.54 (ddd, *J* = 8.1, 7.2, 1.5 Hz, 1H), Ar–CH, 7.47 – 7.43 (m, 2H, 2 × Ar–CH), 7.37 (ddd, *J* = 8.6, 7.2, 1.5 Hz, 1H, Ar–CH), 2.29 (s, 2H, NH_2), 1.65 (s, 6H, $C(CH_3)_2$). ^{13}C NMR (101 MHz, $CDCl_3$) δ 151.7 (Ar–C_q), 142.8 (Ar–C_q), 138.8 (Ar–C_q), 138.7 (Ar–C_q), 133.3 (Ar–CH), 133.0 (Ar–CH), 129.1 (2 × Ar–CH), 128.7 (Ar–CH), 128.0 (2 × Ar–CH), 126.8 (Ar–CH), 54.1 (NH_2*C*$(CH_3)_2$), 33.7 ($NH_2C($*C*$H_3)_2$). HRMS (FTMS+pAPCI) m/z calcd. For $C_{15}H_{17}NO_2SCl$ [M+H]: 310.0663; found: 310.0663.

2-(2-(4-Bromobenzenesulfonyl)phenyl)propan-2-amine (3ag)

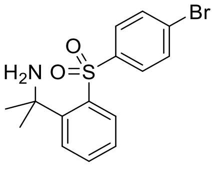

Prepared according to general procedure **A** using cumylamine (43.2 μL, 0.3 mmol) and 4-Bromobenzene sulfinic acid sodium salt (48.4 mg, 0.2 mmol). Sulfonyl amine **3ag** was isolated by flash column chromatography (100% EtOAc) as a brown oil (44.0 mg, 62%). R_f 0.33 (10% MeOH:EtOAc). IR (film)/cm^{-1} 3395 (br, weak, N–H), 3083, 2965, 1570, 1469, 1385, 1296, 1230, 1145, 1107, 1085, 1065, 1006, 816, 749, 664, 577, 558. ^{1}H NMR (400 MHz, $CDCl_3$) δ 8.11 (d, *J* = 8.1 Hz, 1H, Ar–CH), 7.66 (d, *J* = 8.1 Hz, 2H, 2 × Ar–CH), 7.63 – 7.58 (m, 3H, 3 × Ar–CH), 7.54 (dd, *J* = 7.6, 7.6 Hz 1H, Ar–CH), 7.37 (dd, *J* = 7.6, 7.6 Hz, 1H, Ar–CH), 2.03 (s, 2H, NH_2), 1.64 (s, 6H, $C(CH_3)_2$). ^{13}C NMR (101 MHz, $CDCl_3$) δ 151.9 (Ar–C_q), 143.5 (Ar–C_q), 138.7 (Ar–C_q), 133.3 (Ar–CH), 133.0 (Ar–CH), 132.0 (2 × Ar–CH), 128.7 (Ar–CH), 128.0 (2 × Ar–CH), 127.1 (Ar–C_q), 126.7 (Ar–CH), 54.1 (NH_2*C*$(CH_3)_2$), 33.8 ($NH_2C($*C*$H_3)_2$). HRMS (FTMS+pAPCI) m/z calcd. For $C_{15}H_{17}NO_2SBr$ [M+H]: 354.0158; found: 354.0160.

2-(2-(4-Trifluoromethylbenzenesulfonyl)phenyl)propan-2-amine (3ah)

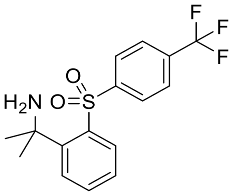

Prepared according to general procedure **A** using cumylamine (43.2 μL, 0.3 mmol) and 4-trifluoromethylbenzene sulfinic acid sodium salt (46.4 mg, 0.2 mmol). Sulfonyl amine **3ah** was isolated by flash column chromatography (100% EtOAc) as an off white solid (45.1 mg, 66%). m.p. = 105–108 °C. R_f 0.3 (EtOAc). IR (film)/cm^{-1} 3400 (br, weak, N–H), 2969, 1607, 1566, 1402, 1363, 1321, 1298, 1170, 1147, 1130, 1061, 1014, 837, 744, 603, 588, 425. ^{1}H NMR (400 MHz, $CDCl_3$) δ 8.28 (d, *J* = 8.2 Hz, 1H, Ar–CH), 7.87 (d, *J* = 8.2 Hz, 2H, 2 × Ar–CH), 7.71 (d, *J* = 8.2 Hz, 2H, 2 × Ar–CH), 7.61–7.56 (d, *J* = 6.1 Hz, 2H, 2 × Ar–CH), 7.42 (dd, *J* = 7.3, 7.3 Hz, 1H, Ar–CH), 1.85 (s, 2H, NH_2), 1.62 (s, 6H, $C(CH_3)_2$). ^{13}C NMR (101 MHz, $CDCl_3$) δ 152.3 (Ar–C_q), 149.0 (Ar–C_q), 137.9 (Ar–C_q), 133.6 (Ar–CH), 133.4 (Ar–CH), 133.4 (q, *J* = 32.9 Hz, Ar–C_q), 128.7 (Ar–CH), 126.8 (Ar–CH), 126.2 (2 × Ar–CH), 125.8 (q, *J* = 4.0 Hz, 2 × Ar–CH), 123.3 (q, *J* = 272.8 Hz, CF_3), 54.0 ($H_2N\mathit{C}(CH_3)_2$), 34.1 ($H_2NC(\mathit{C}H_3)_2$). ^{19}F NMR (377 MHz, $CDCl_3$) δ -62.97. HRMS (ESI$^+$) m/z calcd. For $C_{16}H_{17}F_3NO_2S$ [M+H]: 344.0927; found: 344.0935.

2-(2-(Naphthalen-1-ylsulfonyl)phenyl)propan-2-amine (3ai)

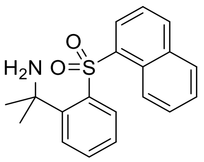

Prepared according to general procedure **A** using cumylamine (43.2 μL, 0.3 mmol) and naphthene-1-sulfinic acid sodium salt (42.8 mg, 0.2 mmol). Sulfonyl amine **3ai** was isolated by flash column chromatography (100% EtOAc) as an yellow solid (32.2 mg, 49%). m.p.= 148–153 °C. R_f 0.06 (EtOAc). IR (film)/cm^{-1} 3390 (N–H), 3057, 2966, 2926, 1592, 1505, 1462, 1412, 1364, 1291, 1196, 1152, 1115, 908, 825, 800, 765, 729, 677, 587, 513, 483, 461. ^{1}H NMR (400 MHz, $CDCl_3$) δ 8.29 (dd, *J* = 8.1, 8.1 Hz, 2H,), 8.13 (d, *J* = 8.3 Hz, 1H, Ar–CH), 7.98–7.92 (m, 1H, Ar–CH), 7.74 (d, *J* = 8.0 Hz, 1H, Ar–CH), 7.61 (dd, *J* = 7.8, 7.8 Hz, 1H, Ar–CH), 7.58–7.43 (m, 4H, 4 × Ar–CH), 7.12–7.05 (m, 1H, Ar–CH), 2.47 (s, 2H, NH_2), 1.80 (s, 6H, $C(CH_3)_2$). ^{13}C NMR (101 MHz, $CDCl_3$) δ 151.0 (Ar–C_q), 140.3 (Ar–C_q), 137.4 (Ar–C_q), 134.6 (Ar–CH), 134.3 (Ar–C_q), 132.6 (Ar–CH), 130.4 (Ar–CH), 129.0 (Ar–CH), 128.9 (Ar–CH), 128.8 (Ar–CH), 128.6 (Ar–C_q), 128.2 (Ar–CH), 126.9 (Ar–CH), 126.6 (Ar–CH), 124.9 (Ar–CH), 124.1 (Ar–CH), 54.4 ($NH_2\mathit{C}(CH_3)_2$), 33.3 ($NH_2C(\mathit{C}H_3)_2$). HRMS (FTMS+pAPCI) m/z calcd. For $C_{19}H_{20}NO_2S$ [M+H]: 326.1209; found: 326.1210.

2-(2-(Methylsulfonyl)phenyl)propan-2-amine (3aj)

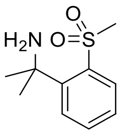

Prepared according to general procedure **A** using cumylamine (43.2 μL, 0.3 mmol) and Methyl sulfinic acid sodium salt (20.0 mg, 0.2 mmol). Sulfonyl amine **3aj** was isolated by flash column chromatography (100% EtOAc) as an brown oil (37.0 mg, 88%). R_f 0.3 (10% MeOH:EtOAc). IR (film)/cm^{-1} 3390 (N–H), 3028, 2967, 2928, 1657, 1596, 1470, 1430, 1363, 1291, 1230, 1142, 1049, 961, 761, 535. ^{1}H NMR (400 MHz, $CDCl_3$) δ 8.25 (d, *J* = 7.9 Hz, 1H, Ar–CH), 7.54 (d, *J* = 6.2 Hz, 2H, 2 × Ar–CH), 7.40 (dd, *J* = 7.2, 7.2 Hz, 1H, Ar–CH), 3.51 (s, 3H, SO_2CH_3), 2.16 (s, 2H, NH_2), 1.69 (s, 6H, $C(CH_3)_2$). ^{13}C NMR (101 MHz, $CDCl_3$) δ 150.9 (Ar–C_q), 139.8 (Ar–C_q), 133.2 (Ar–CH), 132.2 (Ar–CH), 128.2 (Ar–CH), 126.9 (Ar–CH), 54.2 ($NH_2\mathit{C}(CH_3)_2$), 46.9 (SO_2CH_3), 34.8 ($NH_2C(\mathit{C}H_3)_2$). HRMS (FTMS+pAPCI) m/z calcd. For $C_{10}H_{16}NO_2S$ [M+H]: 214.0896; found: 214.0894.

2-(2-(Cyclopropylsulfonyl)phenyl)propan-2-amine (3ak)

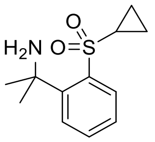

Prepared according to general procedure **A** using cumylamine (43.2 μL, 0.3 mmol) and cyclopropane sulfinic acid sodium salt (25.6 mg, 0.2 mmol). Sulfonyl amine **3ak** was isolated by flash column chromatography (100% EtOAc) as an brown oil (33.7 mg, 70%). R_f 0.09 (100% EtOAc). IR (film)/cm^{-1} 3389 (N–H), 3063, 2963, 1593, 1468. 1427, 1303, 1279, 1228, 1136, 1037, 881, 829, 766, 746, 691, 591, 540, 507. ^{1}H NMR (400 MHz, $CDCl_3$) δ 8.05 (d, *J* = 8.1 Hz, 1H, Ar–CH), 7.59 (d, *J* = 8.1 Hz, 1H, Ar–CH), 7.51 (dd, *J* = 7.6, 7.6 Hz, 1H, Ar–CH), 7.36 (dd, *J* = 7.6, 7.6 Hz, 1H, Ar–CH), 3.79–3.73 (m, 1H, SO_2CH), 2.32 (s, 2H, NH_2), 1.71 (s, 6H, $C(CH_3)_2$), 1.42–1.28 (m, 2H, C*H*HC*H*H), 1.03–0.88 (m, 2H, CH*H*CH*H*). ^{13}C NMR (101 MHz, $CDCl_3$) δ 150.9 (Ar–C_q), 139.6 (Ar–C_q), 132.7 (Ar–CH) , 132.0 (Ar–CH), 128.4 (Ar–CH), 126.6 (Ar–CH), 54.2 (NH_2*C*$(CH_3)_2$), 34.7 (NH_2C(*C*$H_3)_2$), 34.3 (SO_2CH), 6.3 (*C*H_2*C*H_2). HRMS (FTMS+pAPCI) m/z calcd. For $C_{12}H_{18}NO_2S$ [M+H]: 240.1053; found: 240.1055.

2-(2-(Bicyclo[1.1.1]pentan-1-ylsulfonyl)phenyl)propan-2-amine (3al)

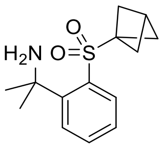

Prepared according to general procedure **A** using cumylamine (43.2 μL, 0.3 mmol) and bicyclo[1.1.1]pentan-1-ylsulfinic acid sodium salt (30.7 mg, 0.2 mmol). Sulfonyl amine **3al** was isolated by flash column chromatography (100% EtOAc) as an off white solid (13.3 mg, 25%). m.p. = 68–70 °C. R_f 0.12 (100% EtOAc). IR (film)/cm^{-1} 3396 (N–H), 2973, 2919, 2883, 1467, 1292, 1205, 1169, 1110, 876, 766, 665, 614, 562. ^{1}H NMR (400 MHz, $CDCl_3$) δ 8.02 (d, *J* = 8.0 Hz, 1H, Ar–CH), 7.66 (d, *J* = 8.0 Hz, 1H, Ar–CH), 7.53 (dd, *J* = 7.6, 7.6 Hz, 1H, Ar–CH), 7.40 (t, *J* = 7.6, 7.6 Hz, 1H, Ar–CH), 2.73 (s, 1H, $C(CH_2)_3C$*H*), 2.26 (s, 2H, NH_2), 2.14 (s, 6H, C(C*H*$_2)_3$CH), 1.68 (s, 6H, $C(CH_3)_2$). ^{13}C NMR (101 MHz, $CDCl_3$) δ 152.1 (Ar–C_q), 136.5 (Ar–C_q), 134.2 (Ar–CH), 133.2 (Ar–CH), 127.7 (Ar–CH), 126.7 (Ar–CH), 57.1 (SO_2C_q), 53.4 (NH_2*C*$(CH_3)_2$), 51.3 (SO_2C(*C*$H_2)_3$CH), 33.4 (NH_2C(*C*$H_3)_2$), 26.1 ($SO_2C(CH_2)_3$*C*H). HRMS (FTMS+pAPCI) m/z calcd. For $C_{14}H_{20}NO_2S$ [M+H]: 266.1209; found: 266.1212.

Reaction Scope Varying the Amine

2-(3-Methoxyphenyl)propan-2-amine (1b)

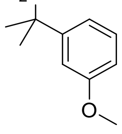

Methylmagnesium bromide (2.34 mL, 7.5 mmol, 3.2 M in 2-MeTHF) was added to a stirring solution of 3-methoxyacetophenone (686 μL, 5 mmol) in diethyl ether (6.65 mL) at 0 °C and was allowed to warm to rt overnight. The reaction was quenched by the addition of a saturated aqueous solution of ammonium chloride (10 mL) and the product extracted from the aqueous layer with diethyl ether (3 × 20 mL), the combined organic extracts were dried over sodium sulfate, filtered then concentrated *in vacuo* to afford the alcohol intermediate. The alcohol intermediate was placed under argon, then dissolved in MeCN (25 mL), trimethylsilylazide (795 μL, 6 mmol) was added and the reaction cooled to 0 °C and $FeCl_3$ (16 mg, 10 mol%) was added and the reaction allowed to warm to rt over 4 h. The reaction was quenched by addition of H_2O and the azide was extracted with EtOAc (3 × 20 mL), dried over sodium sulfate, filtered then concentrated *in vacuo*. The residue was dissolved in diethyl ether (2 mL) and added dropwise to a stirring

solution of $LiAlH_4$ (181 mg, 5 mmol) in Et_2O (2 mL) at 0 °C and the reaction stirred for 4 h. The reaction was cooled to 0 °C then diluted with diethyl ether (10 mL). Water (181 μL) was added slowly followed by 10% aqueous NaOH solution (181 μL) then more water was added (600 μL). The reaction was further diluted by water (20 mL) and transferred to a separating funnel and acidified by the addition of 1 M HCl (20 mL). The organic phase was then discarded. The aqueous phase was basified by the addition of 1 M NaOH (50 mL) and the amine extracted from the aqueous phase with diethyl ether (3 × 20 mL). The combined organic phases were dried over sodium sulfate, filtered, then concentrated *in vacuo* to afford amine **1b** as a yellow oil (363 mg, 44%). IR (film)/cm^{-1} 3400 (br, weak, N–H), 2961, 2833, 1601, 1580, 1484, 1458, 1426, 1286, 1245, 1210, 1175, 1044, 869, 853, 779, 698, 569, 474. ^{1}H NMR (400 MHz, $CDCl_3$) δ 7.31–7.22 (m, 1H, Ar–CH), 7.14–7.04 (m, 2H, 2 × Ar–CH), 6.83–6.70 (m, 1H, Ar–CH), 3.83 (s, 3H, OCH_3), 1.60 (s, 2H, NH_2), 1.49 (s, 6H, $C(CH_3)_2$). ^{13}C NMR (101 MHz, $CDCl_3$) δ 159.4 (Ar–C_q), 152.1 (Ar–C_q), 129.1 (Ar–CH), 117.1 (Ar–CH), 111.0 (Ar–CH), 110.9 (Ar–CH), 55.1 (NH_2*C*$(CH_3)_2$), 52.4 ($NH_2C($*C*$H_3)_2$), 32.7 (OCH_3). Analytical data (IR, ^{1}H, ^{13}C) is consistent with the reported literature.[14]

2-(3-Methoxy-2-(4-methylbenzenesulfonyl)phenyl)propan-2-amine (3ba)

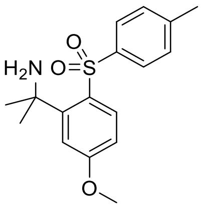

Prepared according to general procedure **B** using 2-(3-methoxyphenyl)propan-2-amine (49.6 mg, 0.3 mmol) and 4-methylbenzene sulfinic acid sodium salt (35.7 mg, 0.2 mmol). Sulfonyl amine **3ba** was isolated by flash column chromatography (100% EtOAc) as yellow oil (47.7 mg, 74%). R_f 0.08 (100% EtOAc). IR (film)/cm^{-1} 3400 (br, weak, N–H), 2967, 1592, 1564, 1463, 1402, 1284, 1240, 1140, 1041, 908, 727, 674, 545. ^{1}H NMR (400 MHz, $CDCl_3$) δ 8.11 (d, *J* = 8.9 Hz, 1H, Ar–CH), 7.67 (d, *J* = 8.4 Hz, 2H, 2 × Ar–CH), 7.28 (d, *J* = 8.4, 2H, 2 × Ar–CH), 7.15 (d, *J* = 2.7 Hz, 1H, Ar–CH), 6.84 (dd, *J* = 8.9, 2.7 Hz, 1H, Ar–CH), 3.88 (s, 3H, OCH_3), 2.42 (s, 3H, $ArCH_3$), 2.36 (s, 2H, NH_2), 1.63 (s, 6H, $C(CH_3)_2$). ^{13}C NMR (101 MHz, $CDCl_3$) δ 162.6 (Ar–C_q), 153.9 (Ar–C_q), 143.0 (Ar–C_q), 141.5 (Ar–C_q), 136.0 (Ar–CH), 130.7 (Ar–C_q), 129.5 (2 × Ar–CH), 126.3 (2 × Ar–CH), 115.7 (Ar–CH), 109.7 (Ar–CH), 55.5 (OCH_3), 54.1 (NH_2*C*$(CH_3)_2$), 33.2 ($NH_2C($*C*$H_3)_2$), 21.5 ($ArCH_3$). HRMS (FTMS+pAPCI) m/z calcd. For $C_{17}H_{22}NO_3S$ [M+H]: 320.1315; found: 320.1312.

2-(3-Fluorophenyl)propan-2-amine (1c)

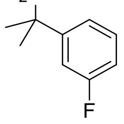

Methylmagnesium bromide (2.34 mL, 7.5 mmol, 3.2 M in 2-MeTHF) was added to a stirring solution of 3-fluoroacetophenone (613 μL, 5 mmol) in diethyl ether (6.65 mL) at 0 °C and was allowed to warm to rt overnight. The reaction was quenched by the addition of a saturated aqueous solution of ammonium chloride (10 mL) and the product was extracted from the aqueous layer with diethyl ether (3 × 20 mL), the combined organic extracts were dried over sodium sulfate, filtered then concentrated *in vacuo* to afford the alcohol intermediate. The alcohol intermediate was placed under argon, then dissolved in MeCN (25 mL), trimethylsilylazide (795 μL, 6 mmol) was added and the reaction cooled to 0 °C. $FeCl_3$ (16 mg, 0.5 mmol) was added reaction allowed to warm to rt over 4 h. The reaction was quenched by addition of H_2O and the azide was extracted with EtOAc (3 × 20 mL). The organic phases were dried over sodium sulfate, filtered then concentrated *in vacuo*. The residue was dissolved in diethyl ether (2 mL) and added dropwise to a stirring solution of $LiAlH_4$ (181 mg, 5 mmol) in Et_2O (3 mL) at 0 °C and the reaction stirred for 4 h. The reaction was cooled to 0 °C then diluted with diethyl ether (10 mL). Water (181 μL) was added slowly followed by 10% aqueous NaOH solution (181 μL) then more water was

added (600 μL). The reaction was further diluted by water (20 mL) and transferred to a separating funnel and acidified by the addition of 1 M HCl (20 mL) and the organic phase was discarded. The aqueous phase was basified by the addition of 1 M NaOH (50 mL) and the amine extracted from the aqueous phase with diethyl ether (3 × 20 mL). The combined organic phases were dried over sodium sulfate, filtered, then concentrated *in vacuo* to afford amine **1c** as a yellow oil (144.8 mg, 19%).IR (film)/cm^{-1} 3400 (br, weak, N–H), 2966, 2928, 1613, 1584, 1482, 1430, 1238, 1194, 910, 867, 782, 696, 470. ^{1}H NMR (400 MHz, $CDCl_3$) δ 7.34–7.19 (m, 3H, 3 × Ar–CH), 6.95–6.86 (m, 1H 1 × Ar–CH), 1.58 (s, 2H, NH_2), 1.49 (s, 6H, C(C*H*$_3$)$_2$). ^{13}C NMR (101 MHz, $CDCl_3$) δ 162.9 (d, *J* = 244.9 Hz, Ar–C_q), 153.2 (d, *J* = 6.1 Hz, Ar–C_q), 129.6 (d, *J* = 8.2 Hz, Ar–CH), 120.3 (d, *J* = 3.0 Hz, Ar–CH), 112.9 (d, *J* = 21.2 Hz, Ar–CH), 112.0 (d, *J* = 22.1 Hz, Ar–CH), 52.4 (NH_2*C*(CH_3)$_2$), 32.8 (NH_2C(*C*H$_3$)$_2$). ^{19}F NMR (377 MHz, $CDCl_3$) δ -113.19. Analytical data (IR, ^{1}H, ^{13}C) is consistent with the reported literature.[14]

2-(3-Fluoro-2-(4-methylbenzenesulfonyl)phenyl)propan-2-amine (3ca)

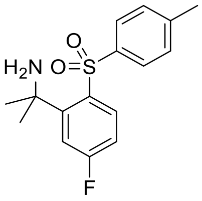

Prepared according to general procedure **B** using 2-(3-fluorophenyl)propan-2-amine (46.0 mg, 0.3 mmol) and 4-methylbenzene sulfinic acid sodium salt (35.4 mg, 0.2 mmol). Sulfonyl amine **3ca** was isolated by flash column chromatography (100% EtOAc) as a yellow oil (45.5 mg, 74%). R_f 0.14 (100% EtOAc). IR (film)/cm^{-1} 3400 (br, weak, N–H), 2968, 2924, 1598, 1577, 1463, 1392, 1294, 1224, 1146, 1091, 928, 874, 811, 677, 549. ^{1}H NMR (400 MHz, $CDCl_3$) δ 8.08 (dd, *J* = 9.0, 6.0 Hz, 1H, Ar–CH), 7.68 (d, *J* = 8.2 Hz, 2H, 2 × Ar–CH), 7.33 (dd, *J* = 11.4, 2.7 Hz, 1H, Ar–CH), 7.29 (d, *J* = 8.2, Hz, 2H, 2 × Ar–CH), 7.01 (ddd, *J* = 9.3, 6.8, 2.7 Hz, 1H, Ar–CH), 2.42 (s, 3H, CH_3), 2.16 (s, 2H, NH_2), 1.64 (s, 6H, C(CH_3)$_2$). ^{13}C NMR (101 MHz, $CDCl_3$) δ 164.6 (d, *J* = 255.3 Hz, Ar–C_q), 155.5 (d, *J* = 7.4 Hz, Ar–C_q), 143.5 (Ar–C_q), 140.7 (Ar–C_q), 135.9 (d, *J* = 9.5 Hz, Ar–CH), 135.5 (d, *J* = 3.5 Hz, Ar–C_q), 129.6 (2 × Ar–CH), 126.7 (2 × Ar–CH), 116.1 (d, *J* = 23.4 Hz, Ar–CH), 113.2 (d, *J* = 21.2 Hz, Ar–CH), 54.3 (NH_2*C*(CH_3)$_2$), 33.3 (NH_2C(*C*H$_3$)$_2$), 21.5 (ArCH_3). ^{19}F NMR (377 MHz, $CDCl_3$) δ -104.79. HRMS (TOF-ESI$^+$) m/z calcd. For $C_{16}H_{19}NO_2SF$ [M+H]: 308.1121; found: 308.1114.

2-(3-Bromophenyl)propan-2-amine (1d)

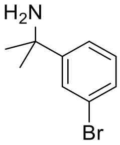

Methylmagnesium bromide (4.68 mL, 15 mmol, 3.2 M in 2-MeTHF) was added to a stirring solution of methyl 3-bromobenzoate (800 μL, 5 mmol) in diethyl ether (6.65 mL) at 0 °C and was allowed to warm to rt overnight. The reaction was quenched by the addition of a saturated aqueous solution of ammonium chloride (10 mL) and the product extracted from the aqueous layer with diethyl ether (3 × 20 mL). The combined organic extracts were dried over sodium sulfate, filtered then concentrated *in vacuo* to afford the alcohol intermediate. The alcohol intermediate was evacuated and backfilled with argon, then dissolved in CH_2Cl_2 (4 mL), trimethylsilylazide (795 μL, 6 mmol) was added and the reaction cooled to 0 °C and BF_3OEt_2 (741 mL, 6 mmol) was added dropwise and the reaction was allowed to warm to rt overnight. The reaction was quenched by addition of saturated aqueous $NaHCO_3$ and the azide was extracted with CH_2Cl_2 (3 × 20 mL), dried over sodium sulfate, filtered then concentrated *in vacuo*. The residue was evacuated and backfilled with argon then dissolved in diethyl ether (3.2 mL). $LiAlH_4$ (181 mg, 5 mmol) was added at 0 °C and the reaction stirred for 4 h. The reaction was quenched by slow addition of water (181 μL) then 10% aqueous NaOH (181 μL) then water (543 μL) was added. The residue was diluted with diethyl ether (10 mL) and filtered through celite and Na_2SO_4 and washed with diethyl ether (3 × 20 mL) then concentrated *in vacuo*. The amine

was extracted into the aqueous phase with 1 M HCl (20 mL) and the organic phase discarded. The aqueous phase was basified by the addition of 1 M NaOH (50 mL) and the amine extracted from the aqueous phase with diethyl ether (3 × 20 mL) dried over sodium sulfate, filtered then concentrated *in vacuo* to afford amine **1d** as a yellow oil (408.6 mg, 38%). IR (film)/cm^{-1} 3400 (br, weak, N–H), 3063, 2962, 2924, 2865, 1590, 1562, 1468, 1410, 1361, 1221, 994, 879, 657, 781, 747, 694, 551, 443. ^{1}H NMR (400 MHz, $CDCl_3$) δ 7.68 (t, *J* = 1.9 Hz, 1H, Ar–CH), 7.44 (ddd, *J* = 7.8, 1.8, 1.0 Hz, 1H, Ar–CH), 7.36 (ddd, *J* = 7.9, 2.0, 1.1 Hz, 1H, Ar–CH), 7.20 (t, *J* = 7.9 Hz, 1H, Ar–CH), 1.54 (s, 2H, NH_2), 1.48 (s, 6H, $C(CH_3)_2$). ^{13}C NMR (101 MHz, $CDCl_3$) δ 152.8 (Ar–C_q), 129.8 (Ar–CH), 129.2 (Ar–CH), 128.1 (Ar–CH), 123.5 (Ar–CH), 122.5 (Ar–C_q), 52.4 (C_q), 32.8 ($C(CH_3)_2$). Analytical data (IR, ^{1}H, ^{13}C) is consistent with the reported literature.[14]

2-(3-Bromo-2-(4-methylbenzenesulfonyl)phenyl)propan-2-amine (3da)

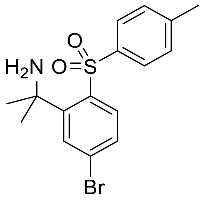

Prepared according to general procedure **B** using 2-(3-bromophenyl)propan-2-amine (64.2 mg, 0.3 mmol) and 4-methylbenzene sulfinic acid sodium salt (35.8 mg, 0.2 mmol). Sulfonyl amine **3da** was isolated by flash column chromatography (100% EtOAc) as a colourless oil (53.6 mg, 72%). R_f 0.3 (100% EtOAc). IR (film)/cm^{-1} 3400 (N–H), 2969, 2924, 1596, 1570, 1545, 1449, 1293, 1148, 1086, 909, 811, 772, 730, 678, 654, 548. ^{1}H NMR (400 MHz, $CDCl_3$) δ 7.86 (d, *J* = 8.6 Hz, 1H, Ar–CH), 7.76 (d, *J* = 2.1 Hz, 1H, Ar–CH), 7.68 (d, *J* = 8.4 Hz, 2H, 2 × Ar–CH), 7.46 (dd, *J* = 8.6, 2.1 Hz, 1H, Ar–CH), 7.30 (d, *J* = 8.4 Hz, 2H, 2 × Ar–CH), 2.42 (s, 3H, CH_3), 2.16 (s, 2H, NH_2), 1.65 (s, 6H, $C(CH_3)_2$). ^{13}C NMR (101 MHz, $CDCl_3$) δ 153.7 (Ar–C_q), 143.8 (Ar–C_q), 140.4 (Ar–C_q), 138.8 (Ar–C_q), 134.4 (Ar–CH), 131.9 (Ar–CH), 129.71 (2 × Ar–CH), 129.67 (Ar–CH), 128.1 (Ar–C_q), 126.9 (2 × Ar–CH), 54.3 ($NH_2\mathit{C}(CH_3)_2$), 33.4 ($NH_2C(\mathit{C}H_3)_2$), 21.6 (CH_3). HRMS (TOF-ESI$^+$) m/z calcd. For $C_{16}H_{19}NO_2SBr$ [M+H]: 368.0320; found: 368.0327.

2-(3-Trifluorophenyl)propan-2-amine (1e)

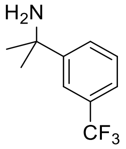

Methylmagnesium bromide (2.81 mL, 9 mmol, 3.2 M in 2-MeTHF) was added dropwise to 3-trifluoromethylbenzonitrile (401 μL mg, 3 mmol) in tetrahydrofuran (5 mL) at rt. After 30 min, titanium isopropoxide (874 μL, 3 mmol) was added and the reaction was heated to 50 °C overnight. The reaction was quenched by the addition of 10% aqueous NaOH (10 mL). The reaction was acidified by the addition of 1 M HCl (20 mL) and the aqueous layer was washed with Et_2O and the organic layer was discarded. The aqueous layer was basified by the addition of 1 M aqueous NaOH (50 mL) and the amine was extracted with Et_2O (3 × 20 mL) and the combined organic phases were dried over sodium sulfate, filtered then concentrated *in vacuo* to afford amine **1e** as a yellow oil (149.2 mg, 24%). IR (film)/cm^{-1} 3300 (br, weak, N–H), 2968, 1613, 1491, 1462, 1328, 1306, 1162, 1116, 1071, 698, 845, 800, 701, 654. ^{1}H NMR (400 MHz, $CDCl_3$) δ 7.81–7.77 (m, 1H, Ar–CH), 7.73–7.69 (m, 1H, Ar–CH), 7.51–7.41 (m, 2H, 2 × Ar–CH), 1.68 (s, 2H, NH_2), 1.52 (s, 6H, $C(CH_3)_2$). ^{13}C NMR* (101 MHz, $CDCl_3$) δ 151.2 (Ar–C_q), 130.4 (q, *J* = 31.9 Hz, Ar–C_q), 128.6 (Ar–CH), 128.3 (Ar–CH), 123.1 (q, *J* = 3.9 Hz, Ar–CH), 121.8–121.1 (m, Ar–CH), 52.5 ($NH_2\mathit{C}(CH_3)_2$), 32.8 ($NH_2C(\mathit{C}H_3)_2$). ^{19}F NMR (377 MHz, $CDCl_3$) δ -62.42. ***CF_3 quaternary ^{13}C signal was not possible to observe due to C–F coupling.

2-(3-Trifluoromethyl-2-(4-methylbenzenesulfonyl)phenyl)propan-2-amine (3ea)

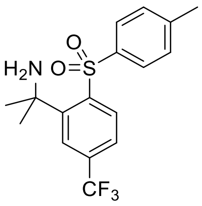

Prepared according to general procedure **B** using 2-(3-Trifluoromethylphenyl)propan-2-amine (61.0 mg, 0.3 mmol) and 4-methylbenzene sulfinic acid sodium salt (35.4 mg, 0.2 mmol). Sulfonyl amine **3ea** was isolated by flash column chromatography (100% EtOAc) as a yellow oil (42.8 mg, 60%). R_f 0.17 (50% EtOAc:hexane). IR (film)/cm^{-1} 3400 (br, weak, N–H), 2971, 1596, 1397, 1329, 1301, 1175, 1130, 1081, 896, 812, 722, 682, 599, 550. ^{1}H NMR (400 MHz, $CDCl_3$) δ 8.05 (d, *J* = 8.4 Hz, 1H, Ar–CH), 7.87 (s, 1H, Ar–CH), 7.73 (d, *J* = 8.3 Hz, 2H, 2 × Ar–CH), 7.55 (d, *J* = 8.4 Hz, 1H, Ar–CH), 7.32 (d, *J* = 8.3 Hz, 2H, 2 × Ar–CH), 2.44 (s, 3H, CH_3), 2.25 (s, 2H, NH_2), 1.70 (s, 6H, $C(CH_3)_2$). ^{13}C NMR (101 MHz, $CDCl_3$) δ 152.9 (Ar–C_q), 144.1 (Ar–C_q), 143.6 (Ar–C_q), 139.7 (Ar–C_q), 134.0 (q, *J* = 32.9 Hz, Ar–C_q), 133.2 (Ar–CH), 129.8 (2 × Ar–CH), 127.2 (2 × Ar–CH), 125.6 (q, *J* = 3.5 Hz, Ar–CH), 123.3 (q, *J* = 3.6 Hz, Ar–CH), 123.2 (q, *J* = 273.1 Hz, CF_3), 54.4 ($NH_2\mathit{C}(CH_3)_2$), 33.5 ($NH_2C(\mathit{C}H_3)_2$), 21.6 (CH_3). ^{19}F NMR (377 MHz, $CDCl_3$) δ -63.27. HRMS (TOF-ESI$^+$) m/z calcd. For $C_{17}H_{19}NO_2F_3S$ [M+H]: 358.1089; found: 358.1079.

2-(4-Bromophenyl)propan-2-amine (1f)

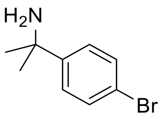

Methylmagnesium bromide (2.34 mL, 7.5 mmol, 3.2 M in 2-MeTHF) was added to a stirring solution of 4-bromoacetophenone (995 mg, 5 mmol) in diethyl ether (6.65 mL) at 0 °C and was allowed to warm to rt overnight. The reaction was quenched by the addition of a saturated aqueous solution of ammonium chloride (10 mL) and the product was extracted from the aqueous layer with diethyl ether (3 × 20 mL). The combined organic extracts were dried over sodium sulfate, filtered the concentrated *in vacuo* to afford the alcohol intermediate. The alcohol intermediate was evacuated and backfilled with argon, then dissolved in CH_2Cl_2 (4 mL), trimethylsilylazide (795 mL, 6 mmol) was added and the reaction cooled to 0 °C and BF_3OEt_2 (741 mL, 6 mmol) was added dropwise and the reaction allowed to warm to rt overnight. The reaction was quenched by addition of $NaHCO_3$ and the azide was extracted with CH_2Cl_2 (3 × 20 mL), dried over sodium sulfate, filtered then concentrated *in vacuo*. The residue was evacuated and backfilled with argon then dissolved in diethyl ether (3.2 mL) and $LiAlH_4$ (151 mg, 4 mmol) was added at 0 °C and the reaction stirred for 4 h. The reaction was quenched by slow addition of Na_2SO_4•10 H_2O until effervescence stopped and the residue was diluted with diethyl ether (10 mL) and filtered through celite and washed with diethyl ether (3 × 20 mL) then concentrated *in vacuo*. The amine was extracted into the aqueous phase by 1 M HCl (20 mL) and the organic phase discarded. The aqueous phase was basified by the addition of 1 M NaOH (50 mL) and the amine extracted from the aqueous phase with diethyl ether (3 × 20 mL) dried over sodium sulfate, filtered then concentrated *in vacuo* to afford amine **1f** as a yellow oil (408.6 mg, 38%). IR (film)/cm^{-1} 3400 (br, weak, N–H), 2962, 2925, 2855, 1587, 1483, 1393, 1360, 1223, 1099, 1006, 819, 725, 543, 416. ^{1}H NMR (400 MHz, $CDCl_3$) δ 7.48–7.42 (m, 2H, 2 × Ar–CH), 7.42–7.36 (m, 2H, 2 × Ar–CH), 1.56 (s, 2H, NH_2), 1.48 (s, 6H, $C(CH_3)_2$). ^{13}C NMR (101 MHz, $CDCl_3$) δ 149.3 (Ar–C_q), 131.1 (2 × Ar–CH), 126.7 (2 × Ar–CH), 120.0 (Ar–C_q), 52.2 (C_q), 32.9 ($C(\mathit{C}H_3)_2$). HRMS (TOF–ESI$^+$) m/z calcd. For $C_9H_{13}NBr$ [M+H]: 214.0231; found: 214.0233.

2-(4-Bromo-2-(4-methylbenzenesulfonyl)phenyl)propan-2-amine (3fa)

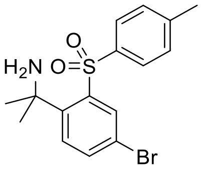

Prepared according to general procedure **B** using 2-(4-bromophenyl)propan-2-amine (64.2 mg, 0.3 mmol) and 4-methylbenzene sulfinic acid sodium salt (35.6 mg, 0.2 mmol). Sulfonyl amine **3fa** was isolated by flash column chromatography (100% EtOAc) as an off white solid (52.2 mg, 71%). m.p. = 86–89 °C. R_f 0.16 (100% EtOAc). IR (film)/cm^{-1} 3395 (N–H), 3079, 2966, 2924, 1595, 1451, 1293, 1140, 1088, 1036, 907, 809, 772, 730, 576, 547.

^{1}H NMR (400 MHz, $CDCl_3$) δ 8.18 (d, *J* = 2.4 Hz, 1H, Ar–CH), 7.69 (d, *J* = 8.4 Hz, 2H, 2 × Ar–CH), 7.60 (dd, *J* = 8.6, 2.4 Hz, 1H, Ar–CH), 7.48 (d, *J* = 8.6 Hz, 1H, Ar–CH), 7.30 (d, *J* = 8.4 Hz, 2H, 2 × Ar–CH), 2.42 (s, 3H, CH_3), 2.15 (s, 2H, NH_2), 1.61 (s, 6H, $C(CH_3)_2$). ^{13}C NMR (101 MHz, $CDCl_3$) δ *150.7 (Ar–C_q), 143.8 (Ar–C_q), 141.5 (Ar–C_q), 140.2 (Ar–C_q), 135.7 (Ar–CH), 135.2 (Ar–CH), 130.4 (Ar–CH), 129.7 (2 × Ar–CH), 126.8 (2 × Ar–CH), 120.2 (Ar–C_q), 54.1 (NH_2*C*(CH_3)$_2$), 33.7 (NH_2C(*C*H_3)$_2$), 21.6 (CH_3). HRMS (ESI$^+$) m/z calcd. For $C_{16}H_{19}NO_2SBr$ [M+H]: 368.0314; found: 368.0330. *Signal identified by HMBC correlation. [NOTE: signals at δ 150.8, 130.4 , 54.1, 33.7 ppm in ^{13}C NMR are heavily supressed by adjacent amine functional group]

2-(4-Pentafluoro-λ^6-sulfaneyl)propan-2-amine (1g)

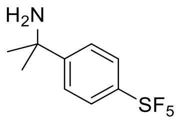

Methylmagnesium bromide (2.81 mL, 9 mmol, 3.2 M in 2-MeTHF) was added dropwise to 4-pentafluoro-λ^6-sulfaneylbenzonitrile (687.5 mg, 3 mmol) in tetrahydrofuran (5 mL) at rt. After 30 min, titanium isopropoxide (874 μL, 3 mmol) was added and the reaction was heated to 50 °C overnight. The reaction was quenched by the addition of 10% aqueous NaOH (10 mL). The reaction was acidified by the addition of 1 M HCl (20 mL) and the aqueous layer was washed with Et_2O and the organic layer was discarded. The aqueous layer was basified by the addition of 1 M aqueous NaOH (50 mL) and the amine was extracted with Et_2O (3 × 20 mL) and the combined organic phases were dried over sodium sulfate, filtered then concentrated *in vacuo* to afford amine **1g** as a yellow oil (157.4 mg, 20%). IR (film)/cm^{-1} 3300 (br, weak, N–H), 1596, 1494, 1462, 1402, 1365, 1229, 1090, 825, 663, 621, 593. ^{1}H NMR (400 MHz, $CDCl_3$) δ 7.71 (d, *J* = 8.8 Hz, 2H, 2 × Ar–CH), 7.62 (d, *J* = 8.8 Hz, 2H, 2 × Ar–CH), 1.68 (s, 2H, NH_2), 1.51 (s, 6H, $C(CH_3)_2$). ^{13}C NMR (101 MHz, $CDCl_3$) δ 154.0 (Ar–C_q), 152.3–151.6 (m, Ar–C_q), 125.9–125.5 (m, 2 × Ar–CH), 125.3 (2 × Ar–CH), 52.5 (*C*(CH_3)$_2$), 32.7 (C(*C*H_3)$_2$). ^{19}F NMR (377 MHz, $CDCl_3$) δ 85.05 (pent, *J* = 151.2, S*F*$_4$F), 63.11 (d, *J* = 151.2 Hz, SF_4*F*). HRMS (TOF–ESI$^+$) m/z calcd. For $C_9H_{13}NF_5S$ [M+H]: 262.0689; found: 262.0683.

2-(3-(Pentafluoro-λ^6-sulfaneyl)-2-(4-methylbenzenesulfonyl)phenyl)propan-2-amine (3ga)

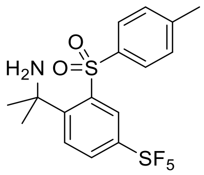

Prepared according to general procedure **B** using 2-(3-(pentafluoro-λ^6-sulfaneyl)phenyl)propan-2-amine (78.4 mg, 0.3 mmol) and 4-methylbenzene sulfinic acid sodium salt (35.5 mg, 0.2 mmol). Sulfonyl amine **3ga** was isolated by flash column chromatography (100% EtOAc) as an amorphous solid (48.2 mg, 58%). R_f 0.21 (100% EtOAc). IR (film)/cm^{-1} 3400 (br, weak, N–H), 2871, 1595, 1466, 1387, 1299, 1142, 1088, 831, 761, 734, 685, 549. ^{1}H NMR (400 MHz, $CDCl_3$) δ 8.45 (d, *J* = 2.6 Hz, 1H, Ar–CH), 7.84 (dd, *J* = 8.8, 2.4 Hz, 1H, Ar–CH), 7.76–7.65 (m, 3H, 3 × Ar–CH), 7.32 (d, *J* = 8.3 Hz, 2H, 2 × Ar–CH), 2.44 (s, 3H, CH_3), 2.07 (s, 2H, NH_2), 1.66 (s, 6H, $C(CH_3)_2$). ^{13}C NMR (101 MHz, $CDCl_3$) δ 155.7 (Ar–C_q), 151.6–150.8 (m, Ar–C_q), 144.1 (Ar–C_q), 141.2 (Ar–C_q), 139.8 (Ar–C_q), 130.3–130.0 (m, Ar–CH), 129.7 (3 × Ar–CH), 129.4 (Ar–CH), 126.9 (2 × Ar–CH), 54.4 (NH_2*C*(CH_3)$_2$), 33.5 (NH_2C(*C*H_3)$_2$), 21.6 (CH_3). ^{19}F NMR (377 MHz, $CDCl_3$) δ 82.42 (pent, *J* = 151.1 Hz, SF_4*F*), 62.74 (d, *J* = 151.1 Hz, S*F*$_4$F). HRMS (TOF-ESI$^+$) m/z calcd. For $C_{16}H_{19}NO_2S_2F_5$ [M+H]: 416.0777; found: 416.0787.

2-(3-(4-Methylbenzenesulfonyl)pyridin-4-yl)propan-2-amine (3ha)

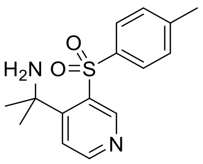

Prepared according to general procedure **A** using 2-(pyridin-4-yl)propan-2-amine (40.9 mg, 0.3 mmol) and 4-methylbenzene sulfinic acid sodium salt (35.6 mg, 0.2 mmol). Sulfonyl amine **3ha** was isolated by flash column chromatography (0–5% MeOH:EtOAc) as a yellow oil (23.6 mg, 41%). R_f 0.29 (10% MeOH:EtOAc). IR (film)/cm^{-1} 3400 (br, weak, N–H), 2971, 2926, 1578, 1525, 1463, 1401, 1297, 1149, 1080, 813, 754, 658, 590, 523. ^{1}H NMR (400 MHz, $CDCl_3$) δ 9.00 (s, 1H, Ar–CH), 8.64 (d, *J* = 5.4 Hz, 1H, Ar–CH), 7.76 (d, *J* = 8.2 Hz, 2H, 2 × Ar–CH), 7.49 (d, *J* = 5.4 Hz, 1H, Ar–CH), 7.33 (d, *J* = 8.2 Hz, 2H, 2 × Ar–CH), 2.52 (s, 2H, NH_2), 2.43 (s, 3H, CH_3), 1.67 (s, 6H, $C(CH_3)_2$). ^{13}C NMR (101 MHz, $CDCl_3$) δ 159.9 (Ar–C_q), 153.4 (Ar–CH), 152.6 (Ar–CH), 144.4 (Ar–C_q), 139.2 (Ar–C_q), 136.6 (Ar–C_q), 129.8 (2 × Ar–CH), 127.5 (2 × Ar–CH), 122.5 (Ar–CH), 54.1 ($NH_2\mathit{C}(CH_3)_2$), 32.6 ($NH_2C(\mathit{C}H_3)_2$), 21.6 (CH_3). HRMS (TOF-ESI$^+$) m/z calcd. For $C_{15}H_{19}N_2O_2S$ [M+H]: 291.1167; found: 291.1164.

2-(Naphthalen-2-yl)propan-2-amine (1i)

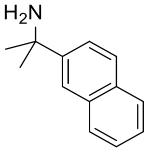

Methylmagnesium bromide (2.81 mL, 9 mmol, 3.2 M in 2-MeTHF) was added to a stirring solution of methyl 2-naphthylnitrile (459 mg, 3 mmol) in tetrahydrofuran (5 mL) at rt and was stirred for 0.5 h then Ti(O*i*Pr)$_4$ (874 µL, 3 mmol) was added and the reaction heated to 50 °C overnight. The reaction was allowed to cool to rt then 10 % aqueous NaOH (10 mL) was added and stirred for 15 min. The solution was acidified with 1 M aqueous HCl (20 mL) the washed with diethyl ether (10 mL) and the organic layer discarded. The aqueous phase was basified with 1 M aqueous NaOH (50 mL) and the product extracted with diethyl ether (3 × 50 mL), the combined organic extracts were dried over sodium sulfate, filtered, then concentrated *in vacuo* to afford amine **1i** as a brown oil (322 mg, 58%). IR (film)/cm^{-1} 3356 (N–H), 3053, 2961, 2925, 2865, 1597, 1524, 1502, 1361, 1194, 1131, 890, 854, 814, 744, 474. ^{1}H NMR (400 MHz, $CDCl_3$) δ 7.94 (d, *J* = 2.2 Hz, 1H, Ar–CH), 7.86–7.80 (m, 3H, 3 × Ar–CH), 7.66 (dd, *J* = 8.7, 2.0 Hz, 1H, Ar–CH), 7.47 (tt, *J* = 6.8, 5.1 Hz, 2H, 2 × Ar–CH), 1.71 (s, 2H, NH_2), 1.60 (s, 6H, $C(CH_3)_2$). ^{13}C NMR (101 MHz, $CDCl_3$) δ 147.6 (Ar–C_q), 133.2 (Ar–C_q), 132.0 (Ar–C_q), 128.0 (Ar–CH), 127.8 (Ar–CH), 127.4 (Ar–CH), 126.0 (Ar–CH), 125.5 (Ar–CH), 124.1 (Ar–CH), 122.4 (Ar–CH), 52.5 (C_q), 32.7 ($C(CH_3)_2$).

2-(3-(4-Methylbenzenesulfonyl)naphthalen-2-yl)propan-2-amine (3ia)

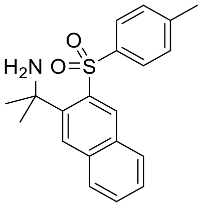

Prepared according to general procedure **B** using 2-(naphthalen-2-yl)propan-2-amine (55.6 mg, 0.3 mmol) and 4-methylbenzene sulfinic acid sodium salt (35.6 mg, 0.2 mmol). Sulfonyl amine **3ia** was isolated by flash column chromatography (100% EtOAc) as an brown oil (12.5 mg, 18%). R_f 0.07 (100% EtOAc). IR (film)/cm^{-1} 3390 (N–H), 3054, 2963, 2922, 1625, 1596, 1577, 1489, 1449, 1382, 1298, 1282, 1207, 1142, 1082, 910, 893, 810, 673, 612, 551, 474. ^{1}H NMR (400 MHz, $CDCl_3$) δ 8.76 (s, 1H, Ar–CH), 8.03 (s, 1H, Ar–CH), 7.90 (d, *J* = 8.2 Hz, 1H, Ar–CH), 7.86 (d, *J* = 8.2 Hz, 1H, Ar–CH), 7.70–7.63 (m, 3H, 3 × Ar–CH), 7.57 (dd, *J* = 7.5, 7.5 Hz, 1H, Ar–CH), 7.29 – 7.22 (m, 2H, 2 × Ar–CH), 2.41 (s, 3H, CH_3), 2.05 (s, 2H, NH_2), 1.74 (s, 6H, $C(CH_3)_2$). ^{13}C NMR (101 MHz, $CDCl_3$) δ *146.6 (Ar–C_q), 143.3 (Ar–C_q), 141.1 (Ar–C_q), 137.4 (Ar–C_q), 135.6 (Ar–CH), 134.8 (Ar–C_q), 130.4 (Ar–C_q), 129.6 (2 × Ar–CH), 129.4 (Ar–CH), 128.8 (Ar–CH), 127.6 (Ar–CH), 127.4 (Ar–CH), 127.2 (Ar–CH), 126.6 (2 × Ar–CH), 54.0 ($NH_2\mathit{C}(CH_3)_2$), 33.6 ($NH_2C(\mathit{C}H_3)_2$), 21.5 (CH_3). HRMS (TOF-ESI$^+$) m/z calcd. For $C_{20}H_{22}NO_2S$ [M+H]: 340.1371; found: 340.1374. *Signal identified by HMBC correlation.

1-(2-(4-Methylbenzenesulfonyl)phenyl)ethan-1-amine (3ja)

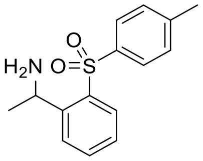

Prepared according to general procedure **A** using 1-phenylethylamine (38.3 μL, 0.3 mmol) and 4-methylbenzene sulfinic acid sodium salt (35.7 mg, 0.2 mmol). Sulfonyl amine **3ja** was isolated by flash column chromatography (100% EtOAc) as an brown oil (24.6 mg, 45%). R_f 0.05 (100% EtOAc). IR (film)/cm^{-1} 3370 (br, weak, N–H), 3062, 2974, 2926, 1594, 1443, 1372, 1297, 1150, 1126, 1090, 910, 813, 766, 728, 647, 571, 519. ^{1}H NMR (400 MHz, $CDCl_3$) δ 8.13 (dd, *J* = 8.0, 1.4 Hz, 1H, Ar–CH), 7.74 (d, *J* = 8.3 Hz, 2H, 2 × Ar–CH), 7.70 (dd, *J* = 7.9, 1.4 Hz, 1H, Ar–CH), 7.61 (td, *J* = 7.4, 1.3 Hz, 1H, Ar–CH), 7.41 (td, *J* = 8.0, 7.4, 1.4 Hz, 1H, Ar–CH), 7.31 (d, *J* = 8.3 Hz, 2H, 2 × Ar–CH), 4.89 (brs, 1H, C*H*CH_3), 2.42 (s, 3H, ArCH_3), 1.16 (d, *J* = 6.5 Hz, 3H, CHCH_3). ^{13}C NMR (101 MHz, $CDCl_3$) δ 144.1 (Ar–C_q), 139.1 (Ar–C_q), 138.1 (Ar–C_q), 134.0* (Ar–CH + Ar–C_q), 129.8 (2 × Ar–CH), 128.9 (Ar–CH), 127.7 (Ar–CH), 127.4 (2 × Ar–CH), 127.1 (Ar–CH), 45.6 (*C*HCH_3), 24.2 (CHCH_3), 21.6 (ArCH_3). HRMS (TOF–ESI$^+$) m/z calcd. For $C_{15}H_{18}NO_2S$ [M+H]: 276.1058; found: 276.1049. *identified by HMBC correlation

Also synthesised using enantioenriched (*R*)-1-phenylethylamine which afforded sulfonyl amine **3ja** as a colourless oil (22 mg, 40%, 99% *ee*): $[\alpha]_D^{20}$ +4.99(*c* 1.00, $CHCl_3$).

***tert*-Butyl (*R*)-(1-phenylethyl)carbamate (H1)**

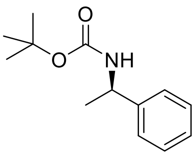

Et_3N (0.30 mL, 2.00 mmol) and Boc_2O (0.30 mL, 1.20 mmol) were added sequentially to a solution of (R)-1-Phenylethylamine (121 mg, 1.00 mmol) in CH_2Cl_2 (10 mL) at room temperature. The resulting reaction mixture was stirred at room temperature for 18 h. The reaction mixture was concentrated *in vacuo* to give the crude product as white solid, which was purified by flash column chromatography (10% EtOAc/pentane) to afford (*R*)-carbamate **H1** (99 mg, 0.45 mmol, 45%) as white solid. R_f 0.45 (10% EtOAc/pentane). m.p. = 88–89 °C. IR (film)/cm^{-1} 3382, 2982, 1685, 1519, 1248, 1175, 698. ^{1}H NMR (400 MHz, $CDCl_3$) δ 7.39 – 7.15 (m, 5H, 5 × Ar–CH), 4.79 (s, 2H, C*H*CH_3, NH), 1.45 (m, 12H, 3 × C(CH_3)$_3$, CHCH_3). ^{13}C NMR (101 MHz, $CDCl_3$) δ 155.1 (C=O), 144.0 (Ar–C_q), 128.6 (2C, 2 × Ar–CH), 127.1 (Ar–CH), 125.9 (2C, 2 × Ar–CH), 79.4 (*C*(CH_3)$_3$), 50.2 (*C*HCH_3), 28.4 (3C, C(CH_3)$_3$), 22.7 (CHCH_3). HRMS (TOF–ESI$^+$) m/z calcd. For $C_{26}H_{39}N_2O_4$ [2M + H]: 443.2910; found 443.2917. $[\alpha]_D^{22}$ +41.83 (*c* 1.00, $CHCl_3$).

The racemic carbamate (104 mg, 0.47 mmol, 47%) was also synthesised using 1-phenylethylamine (121 mg, 1.00 mmol) as the amine following the same protocol.

The enantiomeric excess of *tert*-butyl (*R*)-(1-phenylethyl)carbamate was determined to be >99% by chiral HPLC on Chiralpak ID column using hexanes/isopropyl alcohol (90:10) at a flow rate of 1.0 mL/min, while monitoring at 210 nm. The retention time (tR) of the S enantiomer = 11.4 min, and the retention time (tR) of the R enantiomer = 14.5 min.

HPLC Trace of racemic *tert*-butyl (1-phenylethyl)carbamate (**H1**)

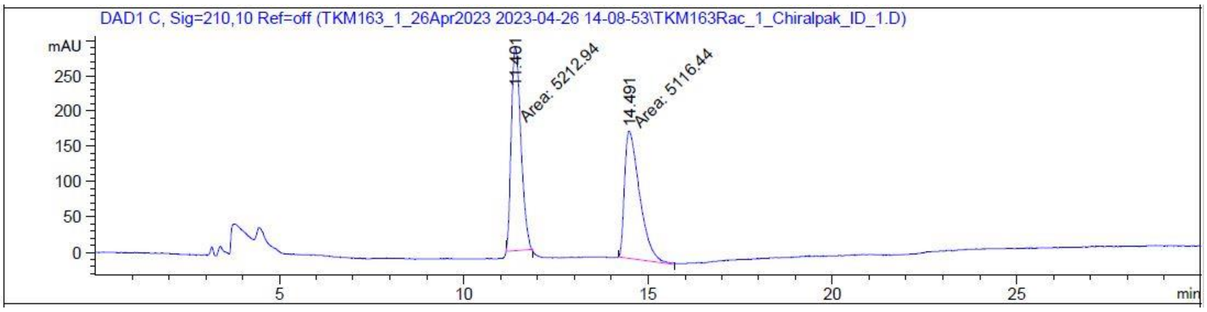

Signal 3: DAD1 C, Sig=210,10 Ref=off

Peak #	RetTime [min]	Type	Width [min]	Area [mAU*s]	Height [mAU]	Area %
1	11.401	MM	0.3020	5212.94092	287.68652	50.4671
2	14.491	MM	0.4733	5116.43994	180.17361	49.5329

Totals : 1.03294e4 467.86014

HPLC Trace of *tert*-butyl (R)-(1-phenylethyl)carbamate (**H1**)

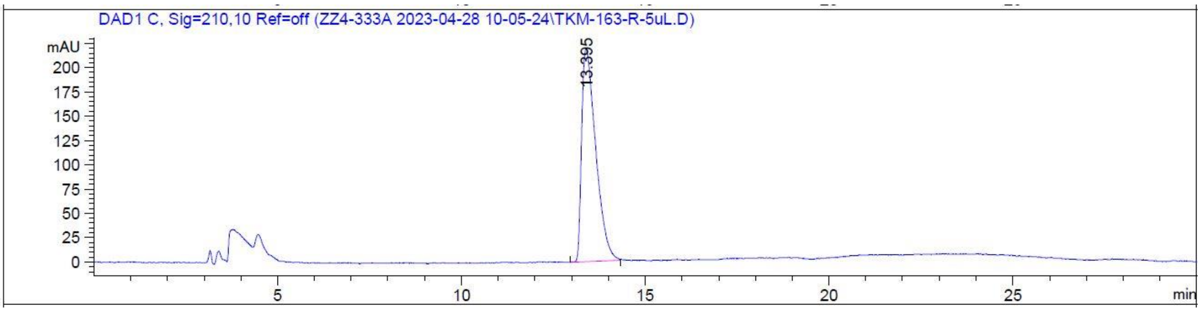

Signal 3: DAD1 C, Sig=210,10 Ref=off

Peak #	RetTime [min]	Type	Width [min]	Area [mAU*s]	Height [mAU]	Area %
1	13.395	BV	0.3817	5816.74658	219.12120	100.0000

Totals : 5816.74658 219.12120

***tert*-Butyl (*R*)-(1-(2-tosylphenyl)ethyl)carbamate (H2)**

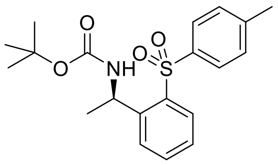

Et_3N (0.05 mL, 0.36 mmol) and Boc_2O (0.05 mL, 0.22 mmol) were added sequentially to a solution of *tert*-butyl (*R*)-(1-phenylethyl)carbamate (11 mg, 0.04 mmol) in CH_2Cl_2 (1 mL) at room temperature. The resulting reaction mixture was stirred at room temperature for 18 h. The reaction mixture was concentrated *in vacuo* to give the crude product as white solid, which was purified by flash column chromatography (10% EtOAc/pentane) to afford (*R*)-carbamate **H2** (15 mg, 0.04 mmol, quant.) as white foam. R_f 0.20 (10% EtOAc/pentane). IR (film)/cm^{-1} 3384, 2975, 2929, 1702, 1501, 1366, 1295, 1154, 1051, 712, 651, 575. ^{1}H NMR (400 MHz, $CDCl_3$) δ 8.09 (d, *J* = 7.8 Hz, 1H, Ar–CH), 7.85 (d, *J* = 7.9 Hz, 2H, 2 × Ar–CH), 7.61 – 7.48 (m, 2H, 2 × Ar–CH), 7.40 (m, 1H, Ar–CH), 7.29 (d, *J* = 8.1 Hz, 2H, 2 × Ar–CH), 5.56 (p, *J* = 6.5 Hz, 1H, C*H*CH_3), 4.85 (s, 1H, NH), 2.39 (s, 3H, Ar–CH_3), 1.34 (s, 12H, 3 × C(CH_3)$_3$, CHCH_3). ^{13}C NMR (101 MHz, $CDCl_3$) δ 154.4 (C=O), 144.8 (Ar–C_q), 143.9 (Ar–C_q), 138.9 (Ar–CH), 138.3 (Ar–CH), 133.8 (Ar–CH), 129.9 (Ar–C_q), 129.7 (2C, 2 × Ar–CH), 127.7 (2C, 2 × Ar–CH), 127.5 (Ar–CH), 127.3 (Ar–C_q), 79.3 (*C*(CH_3)$_3$), 47.1 (*C*HCH_3), 28.2 (3C, C(*C*H_3)$_3$), 23.7 (CH*C*H_3), 21.5 (ArCH_3). HRMS (TOF–ESI$^+$) m/z calcd. For $C_{20}H_{26}NO_4S$ [M + H]: 376.1583; found 376.1570. $[\alpha]_D^{25}$ +4.16 (*c* 1.00, $CHCl_3$).

The racemic carbamate (4 mg, 0.01 mmol, 25%) was also synthesised using 1 *tert*-butyl (1-phenylethyl)carbamate (11 mg, 0.04 mmol) as the amine following the same protocol.

The enantiomeric excess of *tert*-butyl (*R*)-(1-(2-tosylphenyl)ethyl)carbamate was determined to be >99% by chiral HPLC on Chiralpak ID column using hexanes/isopropyl alcohol (90:10) at a flow rate of 1.0 mL/min, while monitoring at 250 nm. The retention time (tR) of the S enantiomer = 39.7 min, and the retention time (tR) of the R enantiomer = 30.1 min.

HPLC Trace of racemic *tert*-butyl (1-(2-tosylphenyl)ethyl)carbamate (**H2)**

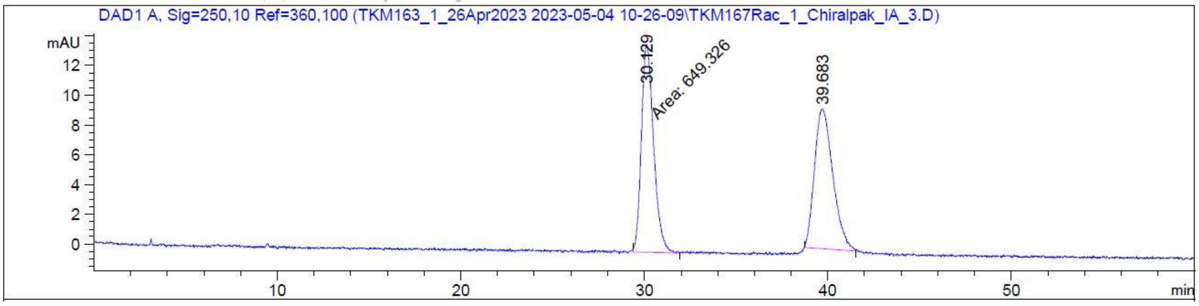

Signal 1: DAD1 A, Sig=250,10 Ref=360,100

Peak #	RetTime [min]	Type	Width [min]	Area [mAU*s]	Height [mAU]	Area %
1	30.129	MM	0.7754	649.32623	13.95722	49.8598
2	39.683	BB	0.8252	652.97778	9.39159	50.1402
Totals :				1302.30402	23.34880	

HPLC Trace of tert-butyl (R)-(1-(2-tosylphenyl)ethyl)carbamate (**H2)**

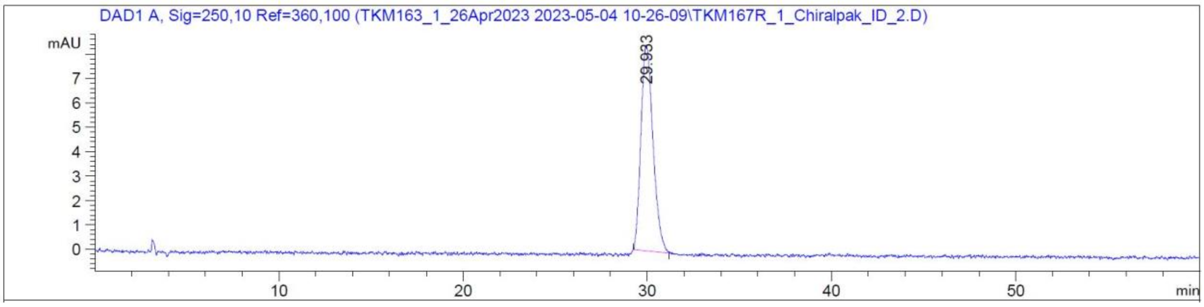

Signal 1: DAD1 A, Sig=250,10 Ref=360,100

Peak #	RetTime [min]	Type	Width [min]	Area [mAU*s]	Height [mAU]	Area %
1	29.933	BB	0.5445	383.74051	8.43863	100.0000
Totals :				383.74051	8.43863	

1-(2-Tosylphenyl)propan-1-amine (3ka)

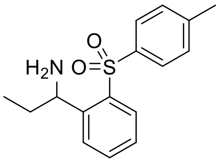

Prepared according to general procedure **A** using 1-phenylpropan-1-amine (43.3 µL, 0.3 mmol) and 4-methylbenzene sulfinic acid sodium salt (35.7 mg, 0.2 mmol). Sulfonyl amine **3ka** was isolated by flash column chromatography (100% EtOAc) as a pale yellow oil (29 mg, 50%). R_f 0.20

(100% EtOAc). IR (film)/cm[-1] 2963, 2929, 1655, 1307, 1152, 766, 652, 576. ^{1}H NMR (500 MHz, $CDCl_3$) δ 8.18 (d, *J* = 8.1 Hz, 1H, Ar–CH), 7.74 (d, *J* = 8.3 Hz, 2H, 2 × Ar–CH), 7.63 – 7.57 (m, 2H, 2 × Ar–CH), 7.46 – 7.40 (m, 1H, Ar–CH), 7.31 (d, *J* = 8.3 Hz, 2H, 2 × Ar–CH), 4.56 (t, *J* = 7.0 Hz, 1H, $CHNH_2$), 2.42 (s, 3H, Ar–CH_3), 1.67 – 1.33 (m, 4H, CH_3CH_2, NH_2), 0.62 (t, *J* = 7.4 Hz, 3H, CH_3CH_2). ^{13}C NMR (100 MHz, $CDCl_3$) δ 146.6 (Ar–C_q), 144.0 (Ar–C_q), 139.3 (Ar–C_q), 138.7 (Ar–C_q), 134.0 (Ar–CH), 129.8 (2 × Ar–CH), 129.0 (Ar–CH), 127.9 (Ar–CH), 127.4 (2 × Ar–CH), 127.0 (Ar–CH), 51.6 ($CHNH_2$), 30.8 (CH_3CH_2), 21.6 (Ar–CH_3), 10.8 (CH_3CH_2). HRMS (TOF–ESI$^+$) m/z calcd. For $C_{16}H_{20}NO_2S$ [M+H]: 290.1215; found: 290.1224.

2,2-Dimethyl-1-(2-tosylphenyl)propan-1-amine (3la)

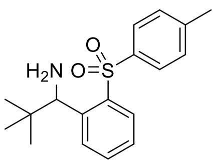

Prepared according to general procedure **B** using 2,2-dimethyl-1-phenylpropan-1-amine (49 mg, 0.3 mmol) and 4-methylbenzene sulfinic acid sodium salt (35.7 mg, 0.2 mmol). Sulfonyl amine **3la** was isolated by flash column chromatography (40% EtOAc) as a colourless oil (44 mg, 0.137 mmol, 67%). R_f 0.23 (40% EtOAc). IR (film)/cm[-1] 2952, 2869, 1307, 1151, 718, 653, 577, 552. ^{1}H NMR (500 MHz, $CDCl_3$) δ 8.19 (dd, *J* = 8.1, 1.4 Hz, 1H, Ar–CH), 7.73 (d, *J* = 8.3 Hz, 2H, 2 × Ar–CH), 7.66 (dd, *J* = 7.9, 1.3 Hz, 1H, Ar–CH), 7.56 (td, *J* = 7.6, 1.5 Hz, 1H, Ar–CH), 7.41 (ddd, *J* = 8.5, 7.3, 1.3 Hz, 1H, Ar–CH), 7.31 (d, *J* = 8.0 Hz, 2H, 2 × Ar–CH), 4.62 (s, 1H, $CHNH_2$), 2.41 (s, 3H, Ar–CH_3), 1.14 (s, 2H, NH_2), 0.87 (s, 9H, 3 × $C(CH_3)_3$). ^{13}C NMR (100 MHz, $CDCl_3$) δ 145.4 (Ar–C_q) , 144.0 (Ar–C_q) , 139.64 (Ar–C_q), 139.56 (Ar–C_q), 133.0 (Ar–CH), 129.8 (2 × Ar–CH), 129.3 (Ar–CH), 129.2 (Ar–CH), 127.5 (2 × Ar–CH), 127.0 (Ar–CH), 57.2 ($CHNH_2$), 35.7 ($C(CH_3)_3$), 27.0 ($C(CH_3)_3$), 21.6 (Ar–CH_3). HRMS (TOF–ESI$^+$) m/z calcd. For $C_{18}H_{24}NO_2S$ [M+H]: 318.1528; found: 318.1517.

2-Phenylbutan-2-amine (1m)

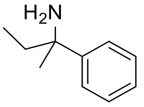

Phenyllithium (4.39 mL, 7.5 mmol, 1.71 M in Bu_2O) was added to a stirring solution of 2-butanone (448 μL, 5 mmol) in diethyl ether (6.65 mL) at 0 °C and was allowed to warm to rt overnight. The reaction was quenched by the addition of a saturated aqueous solution of ammonium chloride (10 mL) and the product extracted from the aqueous layer with diethyl ether (3 × 20 mL). The combined organic extracted were dried over sodium sulfate, filtered then concentrated *in vacuo* to afford the alcohol intermediate. The alcohol intermediate was placed under argon, then dissolved in MeCN (25 mL), trimethylsilylazide (795 μL, 6 mmol) was added and the reaction cooled to 0 °C and $FeCl_3$ (16 mg, 0.5 mmol) was added and the reaction allowed to warm to rt over 4 h. The reaction was quenched by addition of H_2O and the azide was extracted with EtOAc (3 × 20 mL), dried over sodium sulfate, filtered then concentrated *in vacuo*. The residue was dissolved in diethyl ether (2 mL) and added dropwise to a stirring solution of $LiAlH_4$ (181 mg, 5 mmol) in Et_2O (3 mL) at 0 °C and the reaction stirred for 4 h. The reaction was cooled to 0 °C then diluted with diethyl ether (10 mL). Water (181 μL) was added slowly followed by 10% aqueous NaOH solution (181 μL) then more water was added (600 μL). The reaction was further diluted by water (20 mL) and transferred to a separating funnel and acidified by the addition of 1 M HCl (20 mL) and the organic phase was discarded. The aqueous phase was basified by the addition of 1 M NaOH (50 mL) and the amine extracted from the aqueous phase with diethyl ether (3 × 20 mL). The combined organic phases were dried over sodium sulfate, filtered, then concentrated *in vacuo* to afford amine **1m** as a yellow oil (156.1 mg, 21%). IR (film)/cm[-1] 3400 (br, weak, N–H), 3057, 3025, 2963, 2928, 2876, 1737, 1675, 1600, 1493, 1445, 1374, 1207, 1028, 867, 820, 759, 698, 568. ^{1}H NMR (400 MHz, $CDCl_3$) δ 7.49–7.39 (m, 2H, 2 × Ar–CH), 7.38–7.30 (m, 2H, 2 × Ar–CH), 7.26–7.18 (m, 1H, Ar–CH), 1.85–1.71 (m, 2H, CH_2CH_3), 1.62 (s, 2H, NH_2),

1.46 (s, 3H, CH_3), 0.75 (t, *J* = 7.5 Hz, 3H, CH_2CH_3). ^{13}C NMR (101 MHz, $CDCl_3$) δ 148.6 (Ar–C_q), 128.1 (2 × Ar–CH), 126.0 (Ar–CH), 125.3 (2 × Ar–CH), 55.3 (NH_2C), 37.6 (CH_2CH_3), 30.5 (NH_2CCH_3), 8.6 (CH_2CH_3). Analytical data (^{1}H) is consistent with the reported literature.[15]

2-(2-(4-Methylbenzenesulfonyl)phenyl)butan-2-amine (3ma)

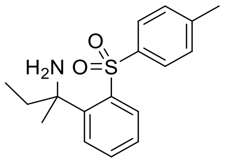

Prepared according to general procedure **B** using 2-phenylbutan-2-amine (48.1 μL mg, 0.3 mmol) and 4-methylbenzene sulfinic acid sodium salt (35.8 mg, 0.2 mmol). Sulfonyl amine **3ma** was isolated by flash column chromatography (100% EtOAc) as a yellow oil (36.6 mg, 60%). R_f 0.17 (100% EtOAc). IR (film)/cm^{-1} 3397 (N–H), 2964, 2929, 2674, 1700, 1594, 1492. 1459, 1430, 1378, 1290, 1145, 1088, 1014, 838, 812, 734, 671, 580. ^{1}H NMR (400 MHz, $CDCl_3$) δ 8.00 (dd, *J* = 8.1, 1.5 Hz, 1H, Ar–CH), 7.69 (d, *J* = 8.4 Hz, 2H, 2 × Ar–CH), 7.58 7.45 (m, 2H, 2 × Ar–CH), 7.37–7.24 (m, 3H, 3 × Ar–CH), 2.42 (s, 3H, $ArCH_3$), 2.25 (s, 2H, NH_2), 2.07–1.97 (m, 2H, CH_2CH_3), 1.54 (s, 3H, CCH_3), 0.69 (t, *J* = 7.5 Hz, 3H, CH_2CH_3). ^{13}C NMR (101 MHz, $CDCl_3$) δ 150.6 (Ar–C_q), 143.3 (Ar–C_q), 140.7 (Ar–C_q), 134.0 (Ar–C_q), 132.5 (2 × Ar–CH), 129.8 (Ar–CH), 129.5 (2 × Ar–CH), 126.8 (2 × Ar–CH), 126.3 (Ar–CH), 57.4 (NH_2C), 37.2 (CH_2CH_3), 31.3 (NH_2CCH_3), 21.5 (Ar-CH_3), 8.8 (CH_2CH_3). HRMS (TOF-ESI^+) m/z calcd. For $C_{17}H_{22}NO_2S$ [M+H]: 304.1371; found: 304.1360.

1,2-Diphenylpropan-2-amine (1n)

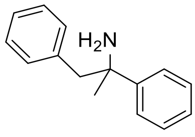

Methylmagnesium bromide (2.81 mL, 9 mmol, 3.2 M in 2-MeTHF) was added to a stirring solution of 2-phenylacetophenone (981 mg, 5 mmol) in diethyl ether (6.65 mL) at 0 °C and was allowed to warm to rt overnight. The reaction was quenched by the addition of a saturated aqueous solution of ammonium chloride (10 mL) and the product was extracted from the aqueous layer with diethyl ether (3 × 20 mL). The combined organic extracts were dried over sodium sulfate, filtered then concentrated *in vacuo* to afford the alcohol intermediate. The alcohol intermediate was placed under argon, then dissolved in MeCN (25 mL), trimethylsilylazide (795 μL, 6 mmol) was added and the reaction cooled to 0 °C. $FeCl_3$ (16 mg, 0.5 mmol) was added reaction and the allowed to warm to rt over 4 h. The reaction was quenched by addition of H_2O and the azide was extracted with EtOAc (3 × 20 mL), dried over sodium sulfate, filtered then concentrated *in vacuo*. The residue was dissolved in diethyl ether (2 mL) and added dropwise to a stirring solution of $LiAlH_4$ (181 mg, 5 mmol) in Et_2O (3 mL) at 0 °C and the reaction stirred for 4 h. The reaction was cooled to 0 °C then diluted with diethyl ether (10 mL). Water (181 μL) was added slowly followed by 10% aqueous NaOH solution (181 μL) then more water was added (600 μL). The reaction was further diluted by water (20 mL) and transferred to a separating funnel and acidified by the addition of 1 M HCl (20 mL) and the organic phase was discarded. The aqueous phase was basified by the addition of 1 M NaOH (50 mL) and the amine extracted from the aqueous phase with diethyl ether (3 × 20 mL). The combined organic phases were dried over sodium sulfate, filtered, then concentrated *in vacuo* to afford amine **1n** as a yellow oil (422.0 mg, 40%). IR (film)/cm^{-1} 3400 (br, weak, N–H), 3082, 3057, 3025, 2964, 2921, 1600, 1579, 1493, 1446, 1372, 1078, 1028, 873, 839, 765, 695, 567. ^{1}H NMR (400 MHz, $CDCl_3$) δ 7.43 (dd, *J* = 7.7, 2.1 Hz, 2H, 2 × Ar–CH), 7.38–7.30 (m, 2H, 2 × Ar–CH), 7.30–7.15 (m, 4H, 4 × Ar–CH), 6.93–6.91 (m, 2H, 2 × Ar–CH), 3.05 (d, *J* = 13.1 Hz, 1H, PhC*H*H), 2.97 (d, *J* = 13.1 Hz, 1H, PhCH*H*), 1.54–1.52 (m, 5H, NH_2 + CH_3). ^{13}C NMR (101 MHz, $CDCl_3$) δ 148.4 (Ar–C_q), 137.5 (Ar–C_q), 130.5 (2 × Ar–CH), 128.0 (2 × Ar–CH), 127.7 (2 × Ar–CH), 126.3 (Ar–CH), 126.2 (Ar–CH), 125.4 (2 × Ar–CH), 55.5 (NH_2C_q), 51.6 ($PhCH_2$) , 30.5 (CH_3). Analytical data (IR, ^{1}H, ^{13}C) is consistent with the reported literature.[15]

1-Phenyl-2-(2-(4-methylbenzenesulfonyl)phenyl)propan-2-amine (3na)

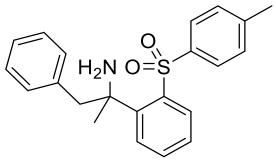

Prepared according to general procedure **B** using 1,2-diphenylpropan-2-amine (63.4 mg, 0.3 mmol) and 4-methylbenzene sulfinic acid sodium salt (35.5 mg, 0.2 mmol). Sulfonyl amine **3na** was isolated by flash column chromatography (50–100% EtOAc:hexane) as an yellow oil (43.9 mg, 60%*). R_f 0.33 (100% EtOAc). IR (film)/cm^{-1} 3395 (N–H), 3057, 3026, 2921, 1594, 1493, 1453, 1288, 1143, 1088, 909, 810, 765, 728, 703, 674, 577, 549. ^{1}H NMR (400 MHz, $CDCl_3$) δ 8.09 (d, *J* = 8.0, 1.5 Hz, 1H, Ar–CH), 7.73 (d, *J* = 8.4 Hz, 2H, 2 × Ar–CH), 7.46–7.40 (m, 2H, 2 × Ar–CH), 7.35 (ddd, *J* = 8.4, 6.1, 2.5 Hz, 1H, Ar–CH), 7.30–7.25 (m, 2H, 2 × Ar–CH), 7.21–7.18 (m, 3H, 3 × Ar–CH), 7.05–7.01 (m, 2H, 2 × Ar–CH), 3.40 (d, *J* = 13.2 Hz, 1H, PhC*H*H), 3.28 (d, *J* = 13.2 Hz, 1H, PhCH*H*), 2.41 (s, 3H, $ArCH_3$), 2.23 (s, 2H, NH_2), 1.54 (s, 3H, CCH_3). ^{13}C NMR (101 MHz, $CDCl_3$) δ 150.0 (Ar–C_q), 143.3 (Ar–C_q), 140.9 (Ar–C_q), 139.9 (Ar–C_q), 137.6 (Ar–C_q), 132.9 (Ar–CH), 132.5 (Ar–CH) , 130.8 (2 × Ar–CH), 129.8 (Ar–CH), 129.5 (2 × Ar–CH), 127.7 (2 × Ar–CH), 126.7 (3 × Ar–CH), 126.3 (Ar–CH), 57.6 (NH_2*C*CH_3), 50.1 (*C*H_2Ph), 30.9 (NH_2C*C*H_3), 21.5 (Ar-CH_3). HRMS (TOF-ESI$^+$) m/z calcd. For $C_{22}H_{24}NO_2S$ [M+H]: 366.1528; found: 366.1513.*contains 6% inseparable starting material.

***tert*-Butyl (2-(4-bromophenyl)propan-2-yl)carbamate (S2)**

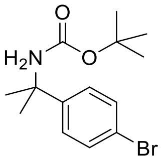

Methylmagnesium bromide (19 mL, 60 mmol, 3.2 M in 2-MeTHF) was added dropwise to 4-bromobenzonitrile (3.64 g, 20 mmol) in diethyl ether (100 mL) at rt. After 30 min, titanium isopropoxide (5.86 mL, 20 mmol) was added and the reaction was heated to 50 °C overnight. The reaction was quenched by the addition of 10% aqueous NaOH (10 mL). The reaction was acidified by the addition of 1 M HCl (20 mL) and the aqueous layer was washed with Et_2O and the organic layer was discarded. The aqueous layer was basified by the addition of 1 M aqueous NaOH (50 mL) and the amine was extracted with Et_2O (3 × 20 mL) and the combined organic phases were dried over sodium sulfate, filtered then concentrated *in vacuo* to afford the amine intermediate**.** The Amine intermediate (2.14 g, 10 mmol) was dissolved in CH_2Cl_2 (100 mL) and cooled to 0 °C. Et_3N (2.79 mL, 20 mmol) and Boc_2O (2.76 mL, 12 mmol) were added sequentially, and the resulting reaction mixture was stirred at room temperature for 18 h. The reaction mixture was concentrated *in vacuo* to give the crude product as brown solid, which was purified by flash column chromatography (10% EtOAc/pentane) to afford bromide **S2** (1.89 g, 60%) as white solid. R_f 0.33 (10% EtOAc/pentane). m.p. = 87–91 °C. IR (film)/cm^{-1} 3426, 3355, 2974, 2930, 1695 (s, C=O), 1489, 1454, 1389, 1363, 1251, 1161, 1073, 1008, 823. ^{1}H NMR (400 MHz, $CDCl_3$) δ 7.44 (d, *J* = 8.5 Hz, 2H, 2 × Ar–CH), 7.28 (d, *J* = 6.1 Hz, 2H, 2 × Ar–CH), 4.94 (s, 1H, NH), 1.60 (s, 6H, $C(CH_3)_2$), 1.40 (s, 9H, $C(CH_3)_3$). ^{13}C NMR (101 MHz, $CDCl_3$) δ 154.0 (C=O), 146.5 (Ar–C_q), 131.3 (2 × Ar–CH), 126.7 (2 × Ar–CH), 120.2 (Ar–C_q), 79.3 (*C*$(CH_3)_3$), 54.6 (*C*$(CH_3)_2$), 29.6 (C(*C*$H_3)_2$), 28.3 (C(*C*$H_3)_3$). HRMS (TOF–ESI$^+$) m/z calcd. for $C_{14}H_{21}NO_2Br$ [M + H]: 314.0756; found 314.0747.

Methyl 4'-(2-((tert-butoxycarbonyl)amino)propan-2-yl)-[1,1'-biphenyl]-4-carboxylate (S3)

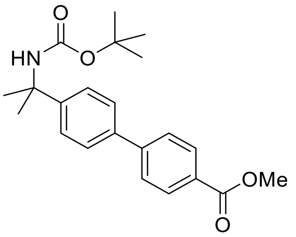

Bromide **S2** (314 mg, 1 mmol), (4-(methoxycarbonyl)phenyl)boronic acid (216 mg, 1.2 mmol), Pd(dppf)Cl_2·CH_2Cl_2 (82 mg, 0.1 mmol) and Cs_2CO_3 (977 mg, 3 mmol) were dissolved in dioxane (9 mL) and H_2O (0.9 mL) and the resulting reaction mixture was degassed with Ar for 15 min. The reaction mixture was heated with stirring at 90 °C for 24 h. The reaction mixture was allowed to cool down to room temperature and filtered through a pad of celite and diluted with CH_2Cl_2 and H_2O. The phases were separated, and the aqueous layer was extracted with CH_2Cl_2 (3 × 20 mL). The combined organic layers were dried over $MgSO_4$, filtered and concentrated *in vacuo* to give the crude product as a black solid, which was purified by column chromatography (10% to 20% EtOAc:pentane) to give the desired carbamate **S3** (315 mg, 85%) as white solid. R_f 0.19 (10% EtOAc/pentane). m.p. = 159–161 °C. IR (film)/cm^{-1} 3372, 2974, 1716 (C=O), 1608, 1496, 1436, 1389, 1364, 1277, 1161, 1108, 1073, 830, 774, 735. ^{1}H NMR (400 MHz, $CDCl_3$) δ 8.10 (d, *J* = 8.4 Hz, 2H, 2 × Ar–CH), 7.66 (d, *J* = 8.3 Hz, 2H, 2 × Ar–CH), 7.59 (d, *J* = 8.5 Hz, 2H, 2 × Ar–CH), 7.50 (d, *J* = 8.5 Hz, 2H, 2 × Ar–CH), 5.00 (s, 1H, NH), 3.95 (s, 3H, OCH_3), 1.67 (s, 6H, $C(CH_3)_2$), 1.42 (s, 9H, $C(CH_3)_3$). ^{13}C NMR (101 MHz, $CDCl_3$) δ 167.0 (C=O), 154.2 (C=O), 147.6 (Ar–C_q), 145.4 (Ar–C_q), 138.0 (Ar–C_q), 130.1 (2 × Ar–CH), 128.7 (Ar–C_q), 127.1 (2 × Ar–CH), 126.9 (2 × Ar–CH) , 125.4 (2 × Ar–CH), 77.2 (*C*$(CH_3)_3$), 54.8 (*C*$(CH_3)_2$), 52.1 (OCH_3), 29.7 (C(*C*$H_3)_2$), 28.4 (C(*C*$H_3)_3$). HRMS (TOF–ESI$^+$) m/z calcd. for $C_{22}H_{28}NO_4$ [M + H]: 370.2018; found 370.2018.

Methyl 4'-(2-aminopropan-2-yl)-[1,1'-biphenyl]-4-carboxylate (S4)

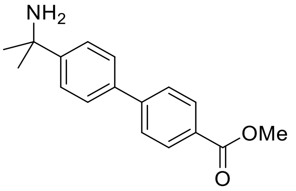

Carbamate **S3** (122 mg, 0.33 mmol) and H_3PO_4 (85%wt, 0.51 mL, 7.5 mmol) were dissolved in THF (1 mL) and the resulting reaction mixture was heated and stirred at 50 °C for 48 h. The reaction mixture was allowed to cool down to room temperature and diluted with H_2O and EtOAc. NaOH (1 M) was added until pH ~ 8. Phases were separated and the aqueous layer was extracted with EtOAc (3 × 20 mL). The combined organic layers were dried over $MgSO_4$, filtered and concentrated *in vacuo* to afford amine **S4** (55 mg, 62%) as a white solid. R_f 0.10 (10% MeOH/EtOAc). m.p. = 123 – 124 °C. IR v_{max} (film)/cm^{-1} 3400 (NH), 2962, 1716 (C=O), 1607, 1437, 1279, 1108, 830, 775, 735. ^{1}H NMR (400 MHz, $CDCl_3$) δ 8.13 – 8.09 (m, 2H, 2 × Ar–CH), 7.69 – 7.65 (m, 2H, 2 × Ar–CH), 7.62 (s, 4H, 4 × Ar–CH), 3.95 (s, 3H, OCH_3), 1.63 (s, 2H, NH), 1.55 (s, 6H, $C(CH_3)_2$). ^{13}C NMR (101 MHz, $CDCl_3$) δ 167.0 (C=O), 150.4 (Ar–C_q), 145.3 (Ar–C_q), 137.8 (Ar–C_q), 130.1 (2 × Ar–CH), 128.7 (Ar–C_q), 127.1 (2 × Ar–CH), 126.9 (2 × Ar–CH), 125.4 (2 × Ar–CH), 52.3 (*C*$(CH_3)_2$), 52.1 (OCH_3), 32.9 (C(*C*$H_3)_2$). HRMS (FTMS) m/z calcd. for $C_{17}H_{20}NO_2$ [M + H]: 270.1489; found 270.1489.

Methyl 4'-(2-aminopropan-2-yl)-3'-tosyl-[1,1'-biphenyl]-4-carboxylate (3oa)

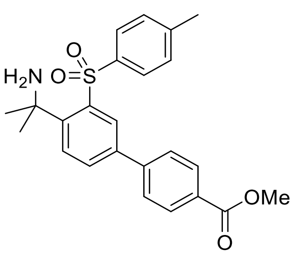

Prepared according to general procedure **A** using methyl 4'-(2-aminopropan-2-yl)-[1,1'-biphenyl]-4-carboxylate **S4** (81 mg, 0.3 mmol) and 4-methylbenzene sulfinic acid sodium salt (35.6 mg, 0.2 mmol). Sulfonyl amine **3oa** (52 mg, 61%) was isolated by flash column chromatography (10% MeOH/EtOAc) as a white solid. R_f 0.30 (10% MeOH/EtOAc). m.p. = 161 – 164 °C. IR v_{max} (film)/cm^{-1} 3400 (N–H), 2952, 1716 (C=O), 1607, 1435, 1273, 1140, 1110, 835,

706, 581. ^{1}H NMR (400 MHz, $CDCl_3$) δ 8.31 (s, 1H), 8.10 (d, J = 7.7 Hz, 2H, 2 × Ar–CH), 7.74 (d, J = 6.9 Hz, 4H, 4 × Ar–CH), 7.59 (d, J = 8.1 Hz, 2H, 2 × Ar–CH), 7.30 (d, J = 8.0 Hz, 2H, 2 × Ar–CH), 3.95 (s, 3H, OCH_3), 2.42 (s, 3H, Ar–CH_3), 2.32 (s, 2H, NH_2), 1.69 (s, 6H, $C(CH_3)_2$). ^{13}C NMR (101 MHz, $CDCl_3$) δ 166.7 (C=O), 151.2 (Ar–C_q), 143.6 (Ar–C_q), 143.0 (Ar–C_q), 140.6 (Ar–C_q), 140.3 (Ar–C_q), 138.2 (Ar–C_q), 131.5 (Ar–CH), 131.1 (Ar–CH), 130.3 (2 × Ar–CH), 129.6 (2 × Ar–CH), 129.5 (Ar–CH, Ar–C_q), 126.79 (2 × Ar–CH), 126.75 (2 × Ar–CH), 54.2 (*C*$(CH_3)_2$), 52.2 (OCH_3), 33.5 (C(*C*$H_3)_2$), 21.5 (Ar–CH_3). HRMS (ESI) m/z calcd. for $C_{24}H_{26}NO_4S$ [M + H]: 424.1583; found 424.1582.

tert-Butyl (2-(4'-(hydroxymethyl)-[1,1'-biphenyl]-4-yl)propan-2-yl)carbamate (S5)

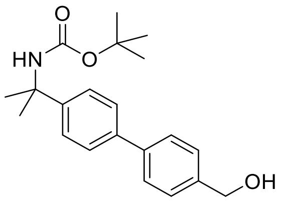

Bromide **S2** (314 mg, 1 mmol), (4-(hydroxymethyl)phenyl)boronic acid (182 mg, 1.2 mmol), Pd(dppf)Cl_2·CH_2Cl_2 (82 mg, 0.1 mmol) and Cs_2CO_3 (977 mg, 3 mmol) were dissolved in dioxane (9 mL) and H_2O (0.9 mL) and the resulting reaction mixture was degassed with Ar for 15 min. The reaction mixture was heated with stirring at 90 °C for 24 h. The reaction mixture was allowed to cool down to room temperature and filtered through a pad of celite and diluted with CH_2Cl_2 & H_2O. Phases were separated and the aqueous layer was extracted with CH_2Cl_2. The combined organic layers were dried over $MgSO_4$, filtered and concentrated *in vacuo* to give the crude product as a black solid, which was purified by column chromatography pentane:EtOAc (9:1 to 1:1) to afford carbamate **S5** (558 mg, 1.63 mmol, 82%) as white foam. R_f 0.61 (50% EtOAc/pentane). IR (film)/cm^{-1} 3405 (OH), 2974, 1692 (C=O), 1498, 1391, 1365, 1163, 1075, 811. ^{1}H NMR (400 MHz, $CDCl_3$) δ 7.62 – 7.53 (m, 4H, 4 × Ar–CH), 7.51 – 7.41 (m, 4H, 4 × Ar–CH), 4.75 (d, *J* = 5.8 Hz, 2H, CH_2OH), 1.67 (s, 6H, $C(CH_3)_2$), 1.42 (s, 9H, $C(CH_3)_3$). ^{13}C NMR (101 MHz, $CDCl_3$) δ 154.2 (C=O), 146.6 (Ar-C_q), 140.4 (Ar-C_q), 139.7 (Ar-C_q), 138.8 (Ar-C_q), 127.4 (2 × Ar–CH), 127.2 (2 × Ar–CH), 126.9 (2 × Ar–CH), 125.3 (2 × Ar–CH), 77.2 (*C*$(CH_3)_3$), 65.2 (*C*H_2OH), 54.8 (*C*$(CH_3)_2$), 29.7 (2 × C(*C*$H_3)_2$), 28.4 (3 × C(*C*$H_3)_3$). HRMS (TOF–ESI$^+$) m/z calcd. for $C_{21}H_{28}NO_3$ [M + H]: 342.2069; found 342.2060.

tert-Butyl (2-(4'-((benzyloxy)methyl)-[1,1'-biphenyl]-4-yl)propan-2-yl)carbamate (S6)

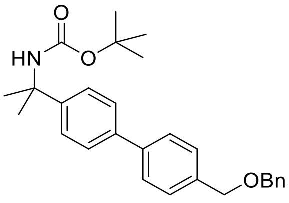

Alcohol **S5** (200 mg, 0.586 mmol) was dissolved in THF (1 mL) and NaH (60%, 24 mg, 0.592 mmol) was added. The resulting mixture was stirred for 10 min. Benzyl bromide (104 µL, 0.879 mmol) was added and the reaction mixture was stirred for 2 hr at room temperature. The reaction mixture was filtered through a pad of celite and washed with CH_2Cl_2 (10 mL). The filtrate was concentrated under reduced pressure to give the crude material as a brown oil, which was purified by column chromatography pentane:EtOAc (9:1) to afford benzyl ether **S6** (158 mg, 0.336 mmol, 62%) as a white solid. R_f 0.50 (10% EtOAc/pentane). m.p. = 106.0 – 106.4 °C. IR (film)/cm^{-1} 3416, 2974, 1720, 1696 (C=O), 1496, 1388, 1362, 1250, 1162, 1072, 813, 739. ^{1}H NMR (400 MHz, $CDCl_3$) δ 7.62 – 7.54 (m, 4H, 4 × Ar–CH), 7.51 – 7.29 (m, 9H, 4 × Ar–CH), 4.98 (s, 1H, NH), 4.61 (s, 2H, CH_2O), 4.61 (s, 2H, CH_2O), 1.68 (s, 6H, $C(CH_3)_2$), 1.42 (s, 9H, $C(CH_3)_3$). ^{13}C NMR (101 MHz, $CDCl_3$) δ 154.1 (C=O), 146.7 (Ar–C_q), 140.3 (Ar–C_q), 138.9 (Ar–C_q), 138.3 (Ar–C_q), 137.1 (Ar–C_q), 128.4 (2 × Ar–CH), 128.2 (2 × Ar–CH), 127.8 (2 × Ar–CH), 127.6 (2 × Ar–CH), 127.1 (1 × Ar–CH), 125.2 (2 × Ar–CH), 77.2 (*C*$(CH_3)_3$), 72.1 (*C*H_2O), 71.9 (*C*H_2O), 54.8 (*C*$(CH_3)_2$), 29.7 (2 × C(*C*$H_3)_2$), 28.3 (3 × C(*C*$H_3)_3$).

2-(4'-((Benzyloxy)methyl)-[1,1'-biphenyl]-4-yl)propan-2-amine (S7)

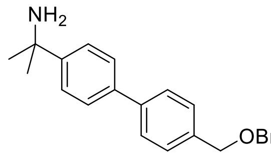

HCl in dioxane (4 M, 0.78 mL, 3.11 mmol) was added to benzyl ether **S6** (134 mg, 0.310 mmol) and the resulting mixture was stirred at room temperature for 3 hr. Et_2O (2 mL) was added and the solid was filtered and washed with Et_2O (1 mL). Aqueous NaOH (2 M, 3 mL) and CH_2Cl_2 (5 mL) were added to dissolve the solid and phases were separated. The aqueous layer was extracted with CH_2Cl_2. The combined organic layers were dried over $MgSO_4$, filtered, and concentrated *in vacuo* to give the amine (83 mg, 0.250 mmol, 92%) as a white solid. R_f 0.03 (10% EtOAc/pentane). m.p. = 59.2 – 61.2 °C. IR (film)/cm^{-1} 3400, 2962, 1497, 1360, 1094, 1073, 813, 740. ^{1}H NMR (400 MHz, $CDCl_3$) δ 7.62 – 7.57 (m, 6H, 6 × Ar–CH), 7.45 (m, 2H, 2 × Ar–CH), 7.42 – 7.36 (m, 4H, 4 × Ar–CH), 7.34 – 7.31 (m, 1H, Ar–CH), 4.612 (s, 2H, CH_2O), 4.609 (s, 2H, CH_2O), 1.69 (s, 6H, $C(CH_3)_2$). ^{13}C NMR (126 MHz, $CDCl_3$) δ 149.3 (Ar–C_q), 140.2 (Ar–C_q), 138.8 (Ar–C_q), 138.3 (Ar–C_q), 137.2 (Ar–C_q), 128.4 (2 × Ar–CH), 128.2 (2 × Ar–CH), 127.8 (2 × Ar–CH), 127.7 (Ar–CH), 127.1 (2 × Ar–CH), 126.9 (2 × Ar–CH), 125.2 (2 × Ar–CH), 72.2 (*C*H$_2$O), 71.8 (*C*H$_2$O), 52.4 (*C*(CH$_3$)$_2$), 32.7 (2 × C(*C*H$_3$)$_2$). HRMS (TOF–ESI$^+$) m/z calcd. for $C_{23}H_{23}O+$ [M – NH$_2$]: 315.1749; found 315.1751.

2-(4'-((Benzyloxy)methyl)-3-tosyl-[1,1'-biphenyl]-4-yl)propan-2-amine (3pa)

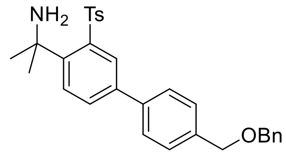

Prepared according to general procedure **A** using 2-(4'-((benzyloxy)methyl)-[1,1'-biphenyl]-4-yl)propan-2-amine **S7** (99 mg, 0.3 mmol) and 4-methylbenzene sulfinic acid sodium salt (35.6 mg, 0.2 mmol). Sulfonyl amine **3pa** (42 mg, 0.0865 mmol, 43%) was isolated by flash column chromatography (10% MeOH/EtOAc) as a yellow oil. R_f 0.23 (10% MeOH/EtOAc). IR (film)/cm^{-1} 3400 (NH), 2967, 2858, 1293, 1142, 1088, 814, 739, 692, 582. ^{1}H NMR (400 MHz, $CDCl_3$) δ 8.27 – 8.23 (m, 1H, Ar–CH), 7.78 – 7.69 (m, 4H, 4 × Ar–CH), 7.54 – 7.28 (m, 11H, 11 × Ar–CH), 4.61 (s, 4H, CH_2O), 2.42 (s, 3H, Ar–CH_3), 2.37 (s, 2H, NH_2), 1.70 (s, 6H, $C(CH_3)_2$). ^{13}C NMR (101 MHz, $CDCl_3$) δ 150.3 (Ar–C_q), 143.5 (Ar–C_q), 140.6 (Ar–C_q), 140.0 (Ar–C_q), 139.2 (Ar–C_q), 138.3 (Ar–C_q), 138.1 (Ar–C_q), 138.0 (Ar–C_q), 131.3 (Ar–CH), 130.9 (Ar–CH), 129.6 (2 × Ar–CH), 129.3 (Ar–CH), 128.42 (2 × Ar–CH), 128.37 (2 × Ar–CH), 127.8 (2 × Ar–CH), 127.7 (Ar–CH), 126.9 (2 × Ar–CH), 126.8 (2 × Ar–CH), 72.3 (*C*H$_2$O), 71.6 (*C*H$_2$O), 54.1 (*C*(CH$_3$)$_2$), 33.5 (2 × C(*C*H$_3$)$_2$), 21.5 (Ar–CH_3). HRMS (TOF–ESI$^+$) m/z calcd. for $C_{20}H_{32}NO_3S$ [M + H]: 486.2103; found 486.2105.

Synthesis of deuterated material

Cumylamine-d$_8$ (1a-d$_8$)

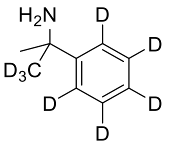

Methylmagnesium bromide (3.65 mL, 11.7 mmol, 3.2 M in 2-MeTHF) was added to a stirring solution of acetophenone-d$_8$ (1 g, 7.8 mmol) in diethyl ether (10.4 mL) at 0 °C and was allowed to warm to rt overnight. The reaction was quenched by the addition of a saturated aqueous solution of ammonium chloride (15 mL) and the product was extracted from the aqueous layer with diethyl ether (3 × 20 mL). The combined organic extracts were dried over sodium sulfate, filtered then concentrated *in vacuo* to afford the alcohol intermediate. The alcohol intermediate was placed under argon, then dissolved in MeCN (39 mL), trimethylsilylazide (1.24 mL, 9.36 mmol) was added and the reaction cooled to 0 °C. $FeCl_3$ (25 mg, 0.78 mmol) was added reaction allowed to warm to rt over 4 h. The reaction was quenched by addition of H_2O and the azide was extracted with EtOAc (3 × 20 mL), dried over sodium sulfate, filtered then concentrated *in vacuo*. The residue was dissolved in diethyl ether (3 mL) and added dropwise to a stirring solution of $LiAlH_4$ (282 mg, 7.8 mmol) in Et_2O (3 mL) at 0 °C and the reaction stirred for 4 h. The reaction was cooled to 0 °C then diluted with diethyl ether (15 mL). Water (282 mL) was added slowly followed by 10% aqueous NaOH solution (282 mL) then more

water was added (1 mL). The reaction was further diluted by water (20 mL) and transferred to a separating funnel and acidified by the addition of 1 M HCl (20 mL) and the organic phase was discarded. The aqueous phase was basified by the addition of 1 M NaOH (50 mL) and the amine extracted from the aqueous phase with diethyl ether (3 × 20 mL). The combined organic phases were dried over sodium sulfate, filtered, then concentrated *in vacuo* to afford amine **1a-d₈** as a yellow oil (178 mg, 16%). IR (film)/cm^{-1} 3366 (N–H), 3275, 2964, 2927, 2274, 2222, 483. ^{1}H NMR (400 MHz, $CDCl_3$) δ 1.71 (s, 2H, NH_2), 1.51 (s, 3H, CH_3). ^{13}C{H} NMR* (101 MHz, $CDCl_3$) δ 150.1 (Ar–C_q), 128.1–127.0 (m, 2 × Ar–CD), 126.2–125.0 (m, Ar–CD), 124.7–123.5 (m, 2 × Ar–CD), 52.2 (NH_2*C*), 32.7 (CH_3), 32.4–31.1 (m, CD_3). ^{13}C{D} NMR (101 MHz, $CDCl_3$) δ 150.1 (Ar–C_q), 127.6 (2 × Ar–CD), 125.6 (Ar–CD), 124.2 (2 × Ar–CD), 32.7 (q, *J* = 126.1 Hz, CH_3). HRMS (TOF–ESI$^+$) m/z calcd. for $C_9H_6D_8N$ [M + H]: 315.1749; found 315.1751. *CD_3 signal not visible due to C–H coupling with adjacent methyl group, this signal is visible in ^{13}C{H} spectrum at δ 32.4–31.1 ppm.

2-(2-Tosylphenyl-3,4,5,6-*d*₄)propan-1,1,1-*d*₃-2-amine (3aa-d₇)

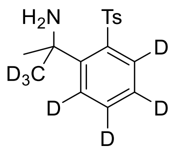

Copper(II) acetate (9 mg, 0.05 mmol) were added to a microwave vial which was then flame dried under argon (ca. 2–5 s). The microwave vial was allowed to cool to room temperature and 2-hydroxynicotinaldehyde (3 mg, 0.025 mmol), 4-methylbenzene sulfinic acid sodium salt (18 mg, 0.10 mmol), manganese(IV) oxide (87 mg, 1 mmol) and the cumylamine-*d*₈ **1a-d₈** (22 mg, 0.15 mmol) were added to a microwave vial sequentially under argon, sealed and HFIP (1 mL, 0.2 M) was added and the vial was submerged in a preheated oil bath to 100 °C for 24 h [Stirring rate set to 500 rpm]. The reaction was allowed to cool to room temperature, diluted with aqueous 1 M HCl (2 mL) and transferred to a separating funnel, washing out the microwave vial with EtOAc (3 × 5 mL). the aqueous 1M NaOH (5 mL) was added [note pH should be between 10 and 14] and the product was extracted from the aqueous phase with EtOAc (3 × 10 mL).[Note: the excess MnO_2 causes the aqueous phase to be extremely dark, and can obscure the phase boundary, brine can be added to aid phase separation.] The combined organic extracts were dried over Na_2SO_4, filtered and concentrated *in vacuo*. Sulfonyl amine **3aa-d₇** (17 mg, 57%) was isolated by flash column chromatography (100% EtOAc) as a pale yellow oil. R_f 0.33 (100% EtOAc). IR (film)/cm^{-1} 3394, 2966, 2925, 1596, 1286, 1142, 1087, 813, 713, 663, 577, 539. ^{1}H NMR (400 MHz, $CDCl_3$) δ 7.70 (d, *J* = 8.3 Hz, 2H, 2 × Ar–CH), 7.28 (d, *J* = 8.1 Hz, 2H, 2 × Ar–CH), 2.67 (s, 2H, NH_2), 2.41 (s, 3H, Ar–CH_3), 1.66 (s, 3H, $C(CH_3)_2$). ^{13}C{H} NMR (101 MHz, $CDCl_3$) δ 151.1 (Ar–C_q), 148.2 –148.0 (m, Ar–CH), 143.6 (Ar–C_q), 140.4 (Ar–C_q), 139.5 (Ar–C_q), 132.6 – 132.1 (m, Ar–CD), 129.6 (2 × Ar–CH), 128.5 – 128.0 (m, Ar–CD), 126.9 (2 × Ar–CH), 126.4 – 125.9 (m, Ar–CD), 54.0 (C_qNH_2), 33.2 (C(*C*H_3)$_2$), 32.8 – 32.2 (C(*C*D_3)$_2$), 21.5 (CH_3). ^{13}C{D} NMR (101 MHz, $CDCl_3$) δ 151.1 (Ar–C_q), 139.5 (Ar–C_q), 132.4 (Ar–CH), 130.5 – 130.3 (m, Ar–CH), 129.0 – 128.7 (m, Ar–CD), 128.2 (Ar–CD), 127.8 (d, *J* = 5.3 Hz), 126.1 (Ar–CH), 54.0 (C_qNH_2), 33.2 (d, *J* = 126.9 Hz, C(*C*D_3)$_2$), 21.5 (d, *J* = 127.1 Hz, CH_3). HRMS (ESI) m/z calcd. for $C_{16}H_{13}D_7NO_2S$ [M + H]: 297.1576; found 297.1645.

Product Derivatization Reactions

N-(2-(2-Tosylphenyl)propan-2-yl)acetamide (4)

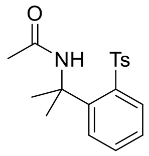

Acetyl chloride (28 µL, 0.40 mmol) was added to a solution of sulfonyl amine **3aa** (58 mg, 0.20 mmol) and NEt_3 (112 µL, 0.80 mmol) in CH_2Cl_2 (1 mL) and the resulting mixture was stirred for 18 h at room temperature. The reaction mixture was quenched with saturated aqueous NH_4Cl (1 mL). Phases were separated and the aqueous layer was extracted with CH_2Cl_2 (3 x 1 mL). The combined organic layers were dried over Na_2SO_4, filtered and concentrated *in vacuo* to give the crude product as a pale yellow oil, which was purified by column chromatography pentane:EtOAc (1:1) to give the desired amide **4** (64 mg, 0.193 mmol, 97%) as white foam. R_f 0.15 (50% EtOAc/pentane). IR (film)/cm^{-1} 3439, 2977, 2926, 1664, 1512, 1293, 1149, 732, 683, 648, 582. ^{1}H NMR (500 MHz, $CDCl_3$) δ 7.70 – 7.64 (m, 3H, 3 × Ar–CH), 7.49 (t, *J* = 7.7 Hz, 1H, Ar–CH), 7.40 (d, *J* = 8.1 Hz, 1H, Ar–CH), 7.33 (d, *J* = 8.3 Hz, 2H, 2 × Ar–CH), 7.19 (t, *J* = 7.7 Hz, 1H, Ar–CH), 6.84 (s, 1H, NH), 2.44 (s, 3H, $ArCH_3$), 1.92 (s, 6H, $C(CH_3)_2$), 1.74 (s, 3H, CH_3CO). ^{13}C NMR (101 MHz, $CDCl_3$) δ 169.9 (CH_3*C*O), 147.9 (Ar-C_q), 144.1 (Ar-C_q), 139.3 (Ar-C_q), 138.2 (Ar-C_q), 132.7 (Ar–CH), 131.9 (Ar–CH), 129.7 (2 × Ar–CH), 128.6 (Ar–CH), 127.4 (2 × Ar–CH), 126.6 (Ar–CH), 55.1 (C_qNH), 30.6 (2C, C(*C*H_3)$_2$), 24.1 (*C*H_3CO), 21.5 (Ar–CH_3). HRMS (TOF–ESI$^+$) m/z calcd. for $C_{18}H_{22}NO_3S$ [M + H]: 332.1320; found 332.1319.

3-(4-Methoxyphenyl)-*N*-(2-(2-tosylphenyl)propan-2-yl)oxetan-3-amine (5)

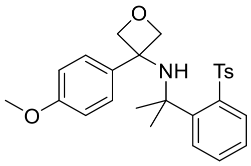

K_2CO_3 (36 mg, 0.26 mmol) and a stirrer bar was added to a reaction vial and then flame dried under Ar. The vial was allowed to cool to room temperature then sulfonyl amine **3aa** (58 mg, 0.20 mmol) and oxetane sulfonyl fluoride (59 mg, 0.24 mmol) were added. The vial was sealed and MeCN (0.7 mL) was added and the resulting reaction mixture was stirred at 80 °C for 1 h. The reaction mixture was filtered through a pad of celite and washed with EtOAc (10 mL). The filtrate was concentrated under reduced pressure to give the crude material as a pale yellow oil, which was purified by column chromatography pentane:EtOAc (4:1) to give the oxetane **5** (74 mg, 0.164 mmol, 82%) as a white foam. R_f 0.09 (20% EtOAc/pentane). IR (film)/cm^{-1} 3359, 2954, 1610, 1512, 1462, 1293, 1246, 1179, 1148, 1032, 983, 733, 573, 550. ^{1}H NMR (500 MHz, $CDCl_3$) δ 7.79 (dd, *J* = 7.4, 2.0 Hz, 1H, Ar–CH), 7.76 (d, *J* = 8.3 Hz, 2H, 2 × Ar–CH), 7.31 (d, *J* = 8.1 Hz, 2H, 2 × Ar–CH), 7.23 – 7.11 (m, 4H, 4 × Ar–CH), 7.08 – 7.02 (m, 1H, Ar–CH), 6.62 (d, *J* = 8.7 Hz, 2H, 2 × Ar–CH), 4.80 – 4.74 (m, 2H, 2 × CCH_2O), 4.72 (d, *J* = 5.8 Hz, 2H, 2 × CCH_2O), 4.50 (s, 1H, NH), 3.73 (s, 3H, OCH_3), 2.42 (s, 3H, $ArCH_3$), 1.48 (s, 6H, $C(CH_3)_2$). ^{13}C NMR (101 MHz, $CDCl_3$) δ 158.1 (Ar-C_q), 150.3 (Ar-C_q), 143.8 (Ar-C_q), 140.1 (Ar-C_q), 139.9 (Ar-C_q), 136.4 (Ar-C_q), 133.1 (Ar–CH), 132.4 (Ar–CH), 129.7 (2 × Ar–CH), 128.3 (Ar–CH), 127.9 (2 × Ar–CH), 127.2 (2 × Ar–CH), 126.3 (Ar–CH), 113.2 (2 × Ar–CH), 84.3 (2 × C*C*H_2O), 61.7 (*C*CH_2O), 57.1 (C_qNH), 55.2 (OCH_3), 31.5 (2C, C(*C*H_3)$_2$), 21.5 (Ar–CH_3). HRMS (TOF–ESI$^+$) m/z calcd. for $C_{26}H_{30}NO_4S$ [M + H]: 452.1896; found 452.1895.

N-(Cyclohexylmethyl)-2-(2-tosylphenyl)propan-2-amine (6)

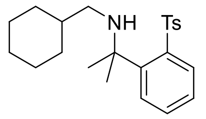

Cyclohexanecarbaldehyde (45 mg, 0.40 mmol), NaBH(OAc)$_3$ (85 mg, 0.40 mmol) and amine (58 mg, 0.20 mmol) were dissolved in dichloroethane (0.7 mL) and the resulting mixture was stirred at rt for 24 h. The reaction was quenched with saturated aqueous NaHCO$_3$ (1 mL) and phases were separated. The aqueous layer was extracted with EtOAc (3x1 mL) and the combined organic layers were dried over Na$_2$SO$_4$, filtered and concentrated to give the crude mixture as a pale yellow oil, which was purified by column chromatography (EtOAc) to give the product amine **6** (55 mg, 0.143 mmol, 72%) as white foam. R$_f$ 0.49 (EtOAc). IR (film)/cm^{-1} 3371, 2919, 2847, 1448, 1296, 1148, 1088, 737, 671, 550. ^{1}H NMR (500 MHz, CDCl$_3$) δ 7.85 (dd, *J* = 8.1, 1.5 Hz, 1H, Ar–CH), 7.69 (d, *J* = 8.3 Hz, 2H, 2 × Ar–CH), 7.63 (d, *J* = 7.5 Hz, 1H, Ar–CH), 7.48 (td, *J* = 7.6, 1.5 Hz, 1H, Ar–CH), 7.35 – 7.27 (m, 3H, 3 × Ar–CH), 3.20 (s, 1H, NH), 2.42 (s, 3H, ArCH$_3$), 2.05 (d, *J* = 6.6 Hz, 2H, CH$_2$Cy), 1.77 – 1.56 (m, 11H, C(CH$_3$)$_2$), 1.36 – 1.01 (m, 4H), 0.80 (qd, *J* = 12.2, 2.9 Hz, 2H). ^{13}C NMR (101 MHz, CDCl$_3$) δ 148.9 (Ar-C$_q$), 143.7 (Ar-C$_q$), 140.2 (2 × Ar-C$_q$), 133.2 (Ar–CH), 132.5 (Ar–CH), 129.9 (Ar–CH), 129.6 (2 × Ar–CH), 127.0 (2 × Ar–CH), 126.7 (Ar–CH), 57.5 (C$_q$NH), 50.2 (CH$_2$NH), 38.6 (*C*H(CH$_2$)$_3$), 31.6 (2 × (*C*H$_2$)$_2$CH$_2$NH), 30.1 (2C, C(*C*H$_3$)$_2$), 26.7 (2C, CH$_2$(*C*H$_2$)$_2$), 26.1 (*C*H$_2$(CH$_2$)$_2$), 21.5 (Ar–CH$_3$). HRMS (TOF–ESI$^+$) m/z calcd. for C$_{23}$H$_{32}$NO$_2$S [M + H]: 386.2154; found 386.2155.

Methyl 3-nitro-4-((2-(2-tosylphenyl)propan-2-yl)amino)benzoate (7)

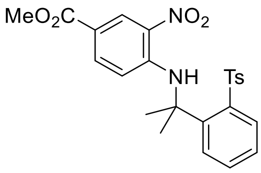

Methyl 4-fluoro-3-nitrobenzoate (20 mg, 0.10 mmol) and sulfonyl amine **3aa** (58 mg, 0.20 mmol) were dissolved in *i*PrOH (1 mL) and the resulting mixture was heated in a sealed tube for 4 h at 100 °C. The reaction was allowed to cool to room temperature and diluted with CH$_2$Cl$_2$ (10 mL) and NaOH (2 M, 10 mL). Phases were separated and the aqueous layer was extracted with CH$_2$Cl$_2$ (2x10 mL) and the combined organic layer was dried over Na$_2$SO$_4$, filtered, and concentrated. The crude mixture was a yellow solid which was purified by column chromatography (pentane:EtOAc 4:1) to give the product amine **7** (47 mg, 0.10 mmol, 100%) as yellow solid. m.p. = 190–195 °C. R$_f$ 0.14 (50% EtOAc/pentane). IR (film)/cm^{-1} 3359, 2952, 1716, 1621, 1527, 1438, 1292, 1241, 1218, 1149, 762, 684. ^{1}H NMR (500 MHz, CDCl$_3$) δ 8.96 (s, 1H, NH), 8.46 (d, *J* = 2.1 Hz, 1H, Ar–CH), 8.39 (d, *J* = 7.8 Hz, 1H, Ar–CH), 7.78 – 7.73 (m, 2H, 2 × Ar–CH), 7.62 – 7.56 (m, 1H, Ar–CH), 7.47 – 7.42 (m, 1H, Ar–CH), 7.22 (d, *J* = 8.3 Hz, 2H, 2 × Ar–CH), 6.78 (d, *J* = 8.0 Hz, 2H, 2 × Ar–CH), 6.01 (d, *J* = 9.1 Hz, 1H), 3.85 (s, 3H, CH$_3$OCO), 2.17 (s, 3H, ArCH$_3$), 2.06 (s, 6H, C(*C*H$_3$)$_2$). ^{13}C NMR (101 MHz, CDCl$_3$) δ 165.4 (CH$_3$O*C*O), 147.3 (Ar-C$_q$), 146.3 (Ar-C$_q$), 142.9 (Ar-C$_q$), 138.6 (Ar-C$_q$), 138.0 (Ar-C$_q$), 135.1 (Ar–CH), 134.4 (2 × Ar–CH), 132.3 (Ar-C$_q$), 129.2 (Ar–CH), 129.1 (2 × Ar–CH), 128.2 (Ar–CH), 128.0 (Ar–CH), 124.9 (2 × Ar–CH), 117.1 (Ar-C$_q$), 115.4 (Ar–CH), 57.4 (C$_q$NH), 52.0 (3C, *C*H$_3$OCO, 2 × C(*C*H$_3$)$_2$) , 21.2 (Ar–CH$_3$). HRMS (TOF–ESI$^+$) m/z calcd. for C$_{24}$H$_{24}$N$_2$O$_6$S [M + H]: 469.1433; found 469.1436.

^{1}H and ^{13}C Spectra of Selected Compounds

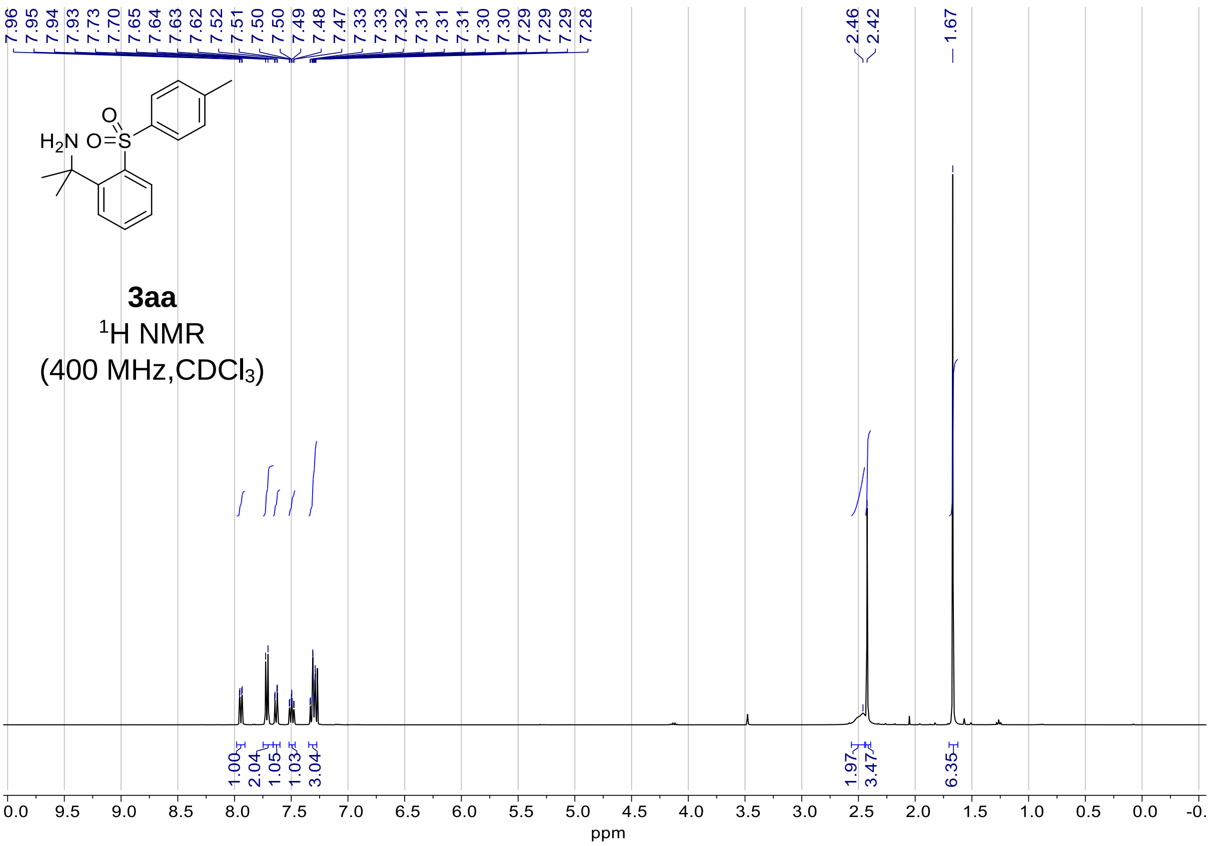
H2N O=S O
3aa
1H NMR
(400 MHz,CDCl3)
7.96 7.95 7.94 7.93 7.73 7.70 7.65 7.64 7.63 7.62 7.52 7.51 7.50 7.50 7.49 7.48 7.47 7.33 7.33 7.32 7.31 7.31 7.31 7.30 7.30 7.29 7.29 7.29 7.28
2.46 2.42
1.67
1.00 2.04 1.05 1.03 3.04 1.97 3.47 6.35
ppm

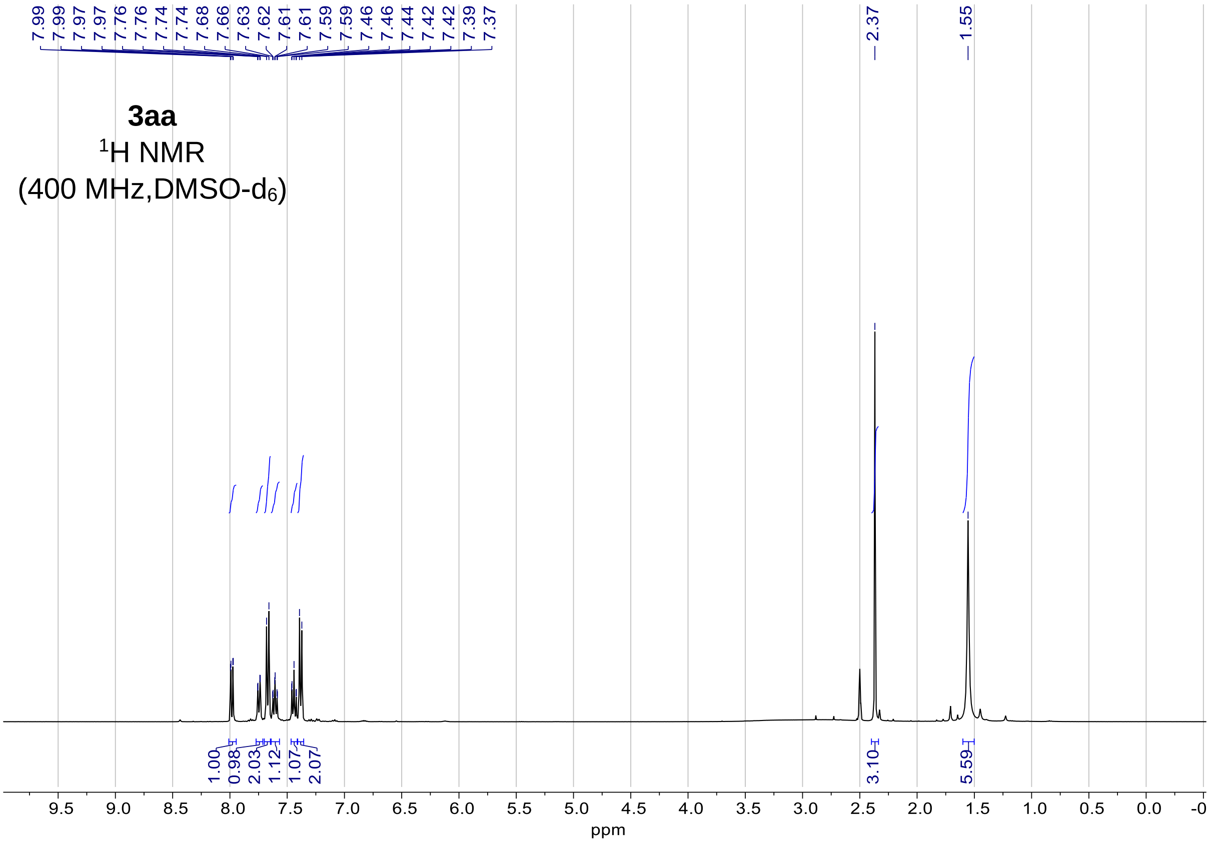
3aa
1H NMR
(400 MHz,DMSO-d6)
7.99 7.99 7.97 7.76 7.76 7.74 7.74 7.68 7.66 7.63 7.62 7.61 7.61 7.59 7.59 7.46 7.46 7.44 7.42 7.42 7.39 7.37
2.37
1.55
1.00 0.98 2.03 1.12 1.07 2.07 3.10 5.59
ppm

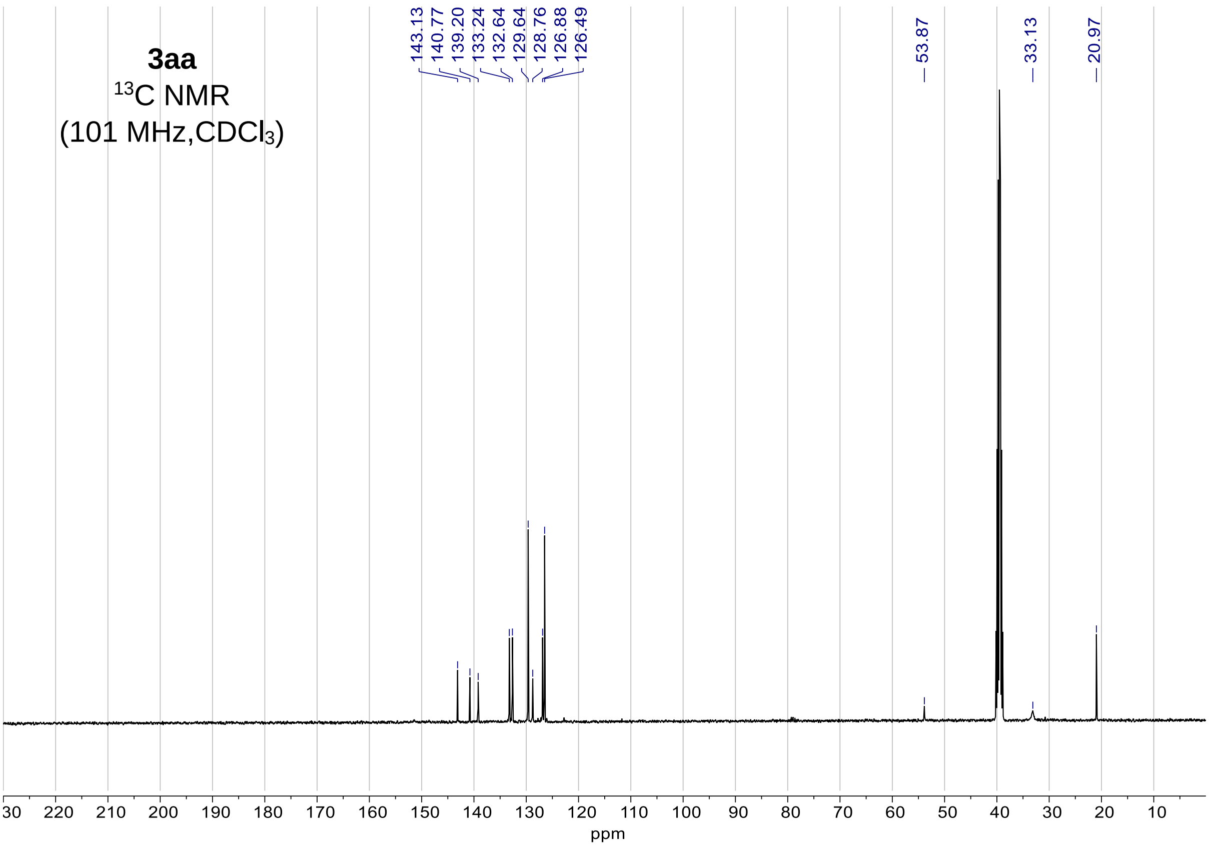
3aa
13C NMR
(101 MHz,CDCl3)
143.13
140.77
139.20
133.24
132.64
129.64
128.76
126.88
126.49
53.87
33.13
20.97
ppm

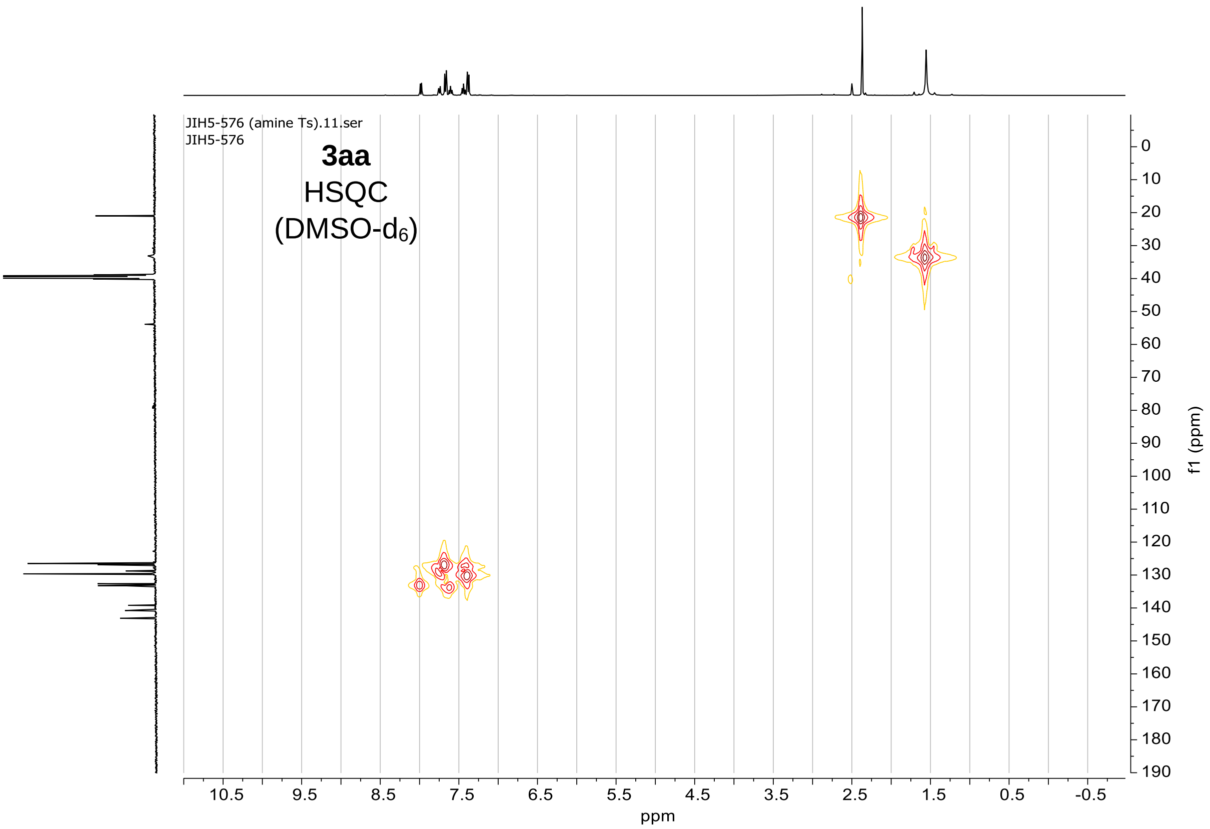
JIH5-576 (amine Ts).11.ser
JIH5-576
3aa
HSQC
(DMSO-d6)
ppm
f1 (ppm)

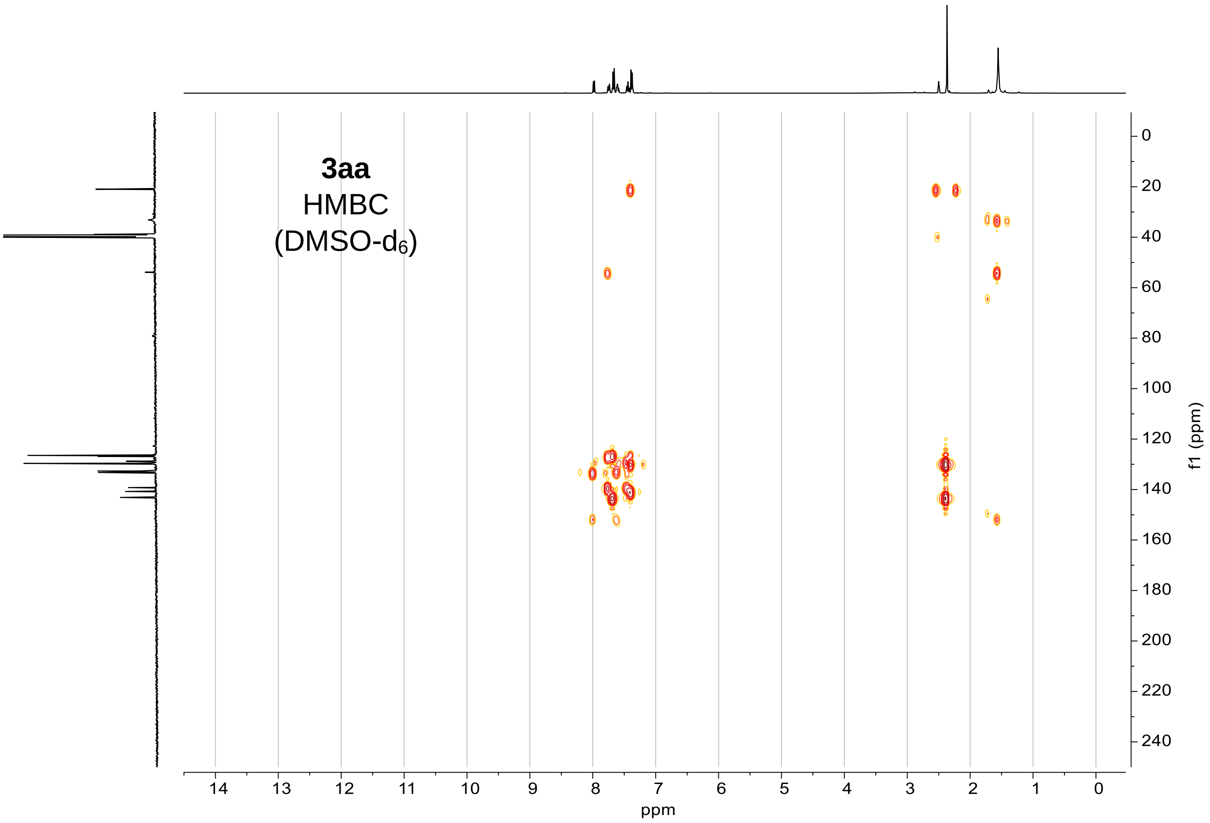
3aa
HMBC
(DMSO-d6)
ppm
f1 (ppm)

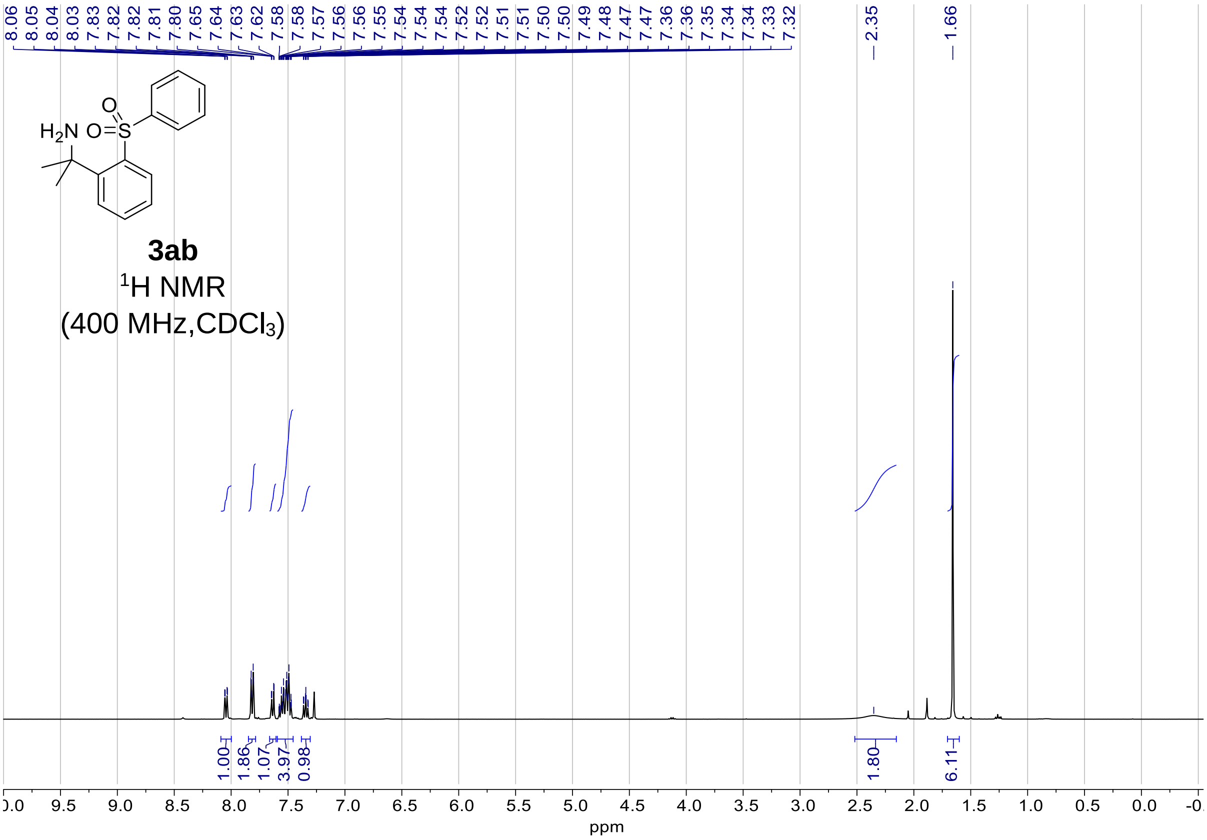
8.06
8.05
8.04
8.03
7.83
7.82
7.82
7.81
7.80
7.65
7.64
7.63
7.62
7.58
7.58
7.57
7.56
7.56
7.55
7.54
7.54
7.54
7.52
7.52
7.51
7.51
7.50
7.50
7.49
7.48
7.47
7.47
7.36
7.36
7.35
7.34
7.34
7.33
7.32
2.35
1.66
H2N O=S O
3ab
1H NMR
(400 MHz,CDCl3)
1.00
1.86
1.07
3.97
0.98
1.80
6.11
ppm

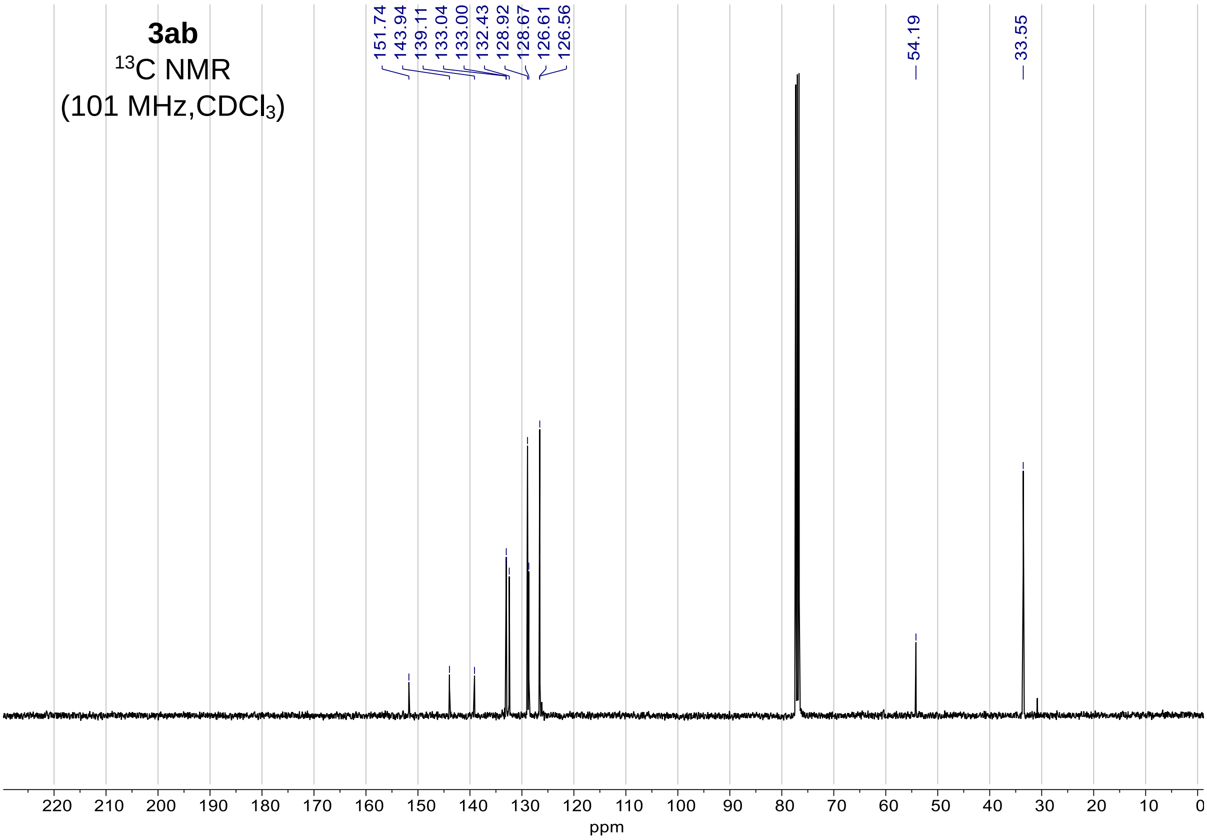
3ab
13C NMR
(101 MHz,CDCl3)
151.74
143.94
139.11
133.04
133.00
132.43
128.92
128.67
126.61
126.56
54.19
33.55
ppm

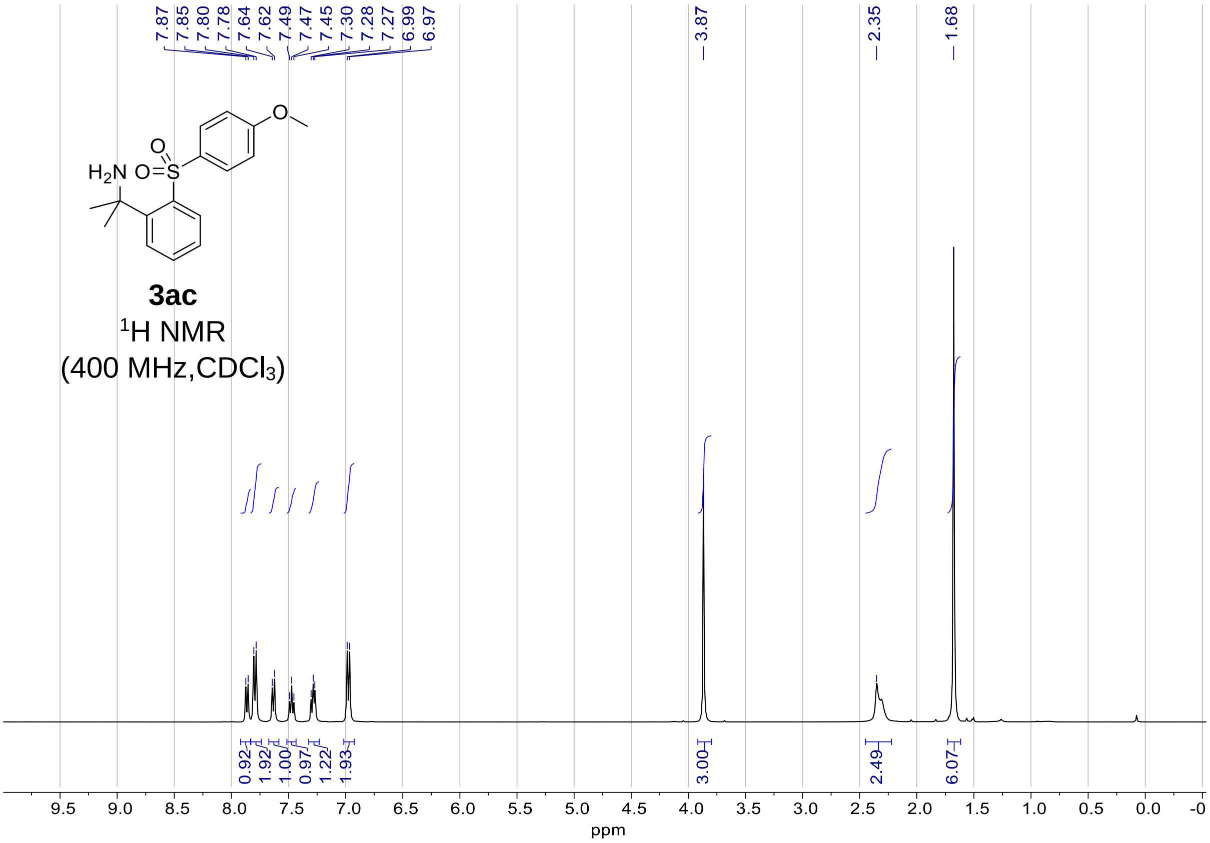
7.87 7.85 7.80 7.78 7.64 7.62 7.49 7.47 7.45 7.30 7.28 7.27 6.99 6.97
3.87
2.35
1.68
H_2N O=S O
3ac
^{1}H NMR
(400 MHz,$CDCl_3$)
0.92 1.92 1.00 0.97 1.22 1.93
3.00
2.49
6.07
9.5 9.0 8.5 8.0 7.5 7.0 6.5 6.0 5.5 5.0 4.5 4.0 3.5 3.0 2.5 2.0 1.5 1.0 0.5 0.0 -0
ppm

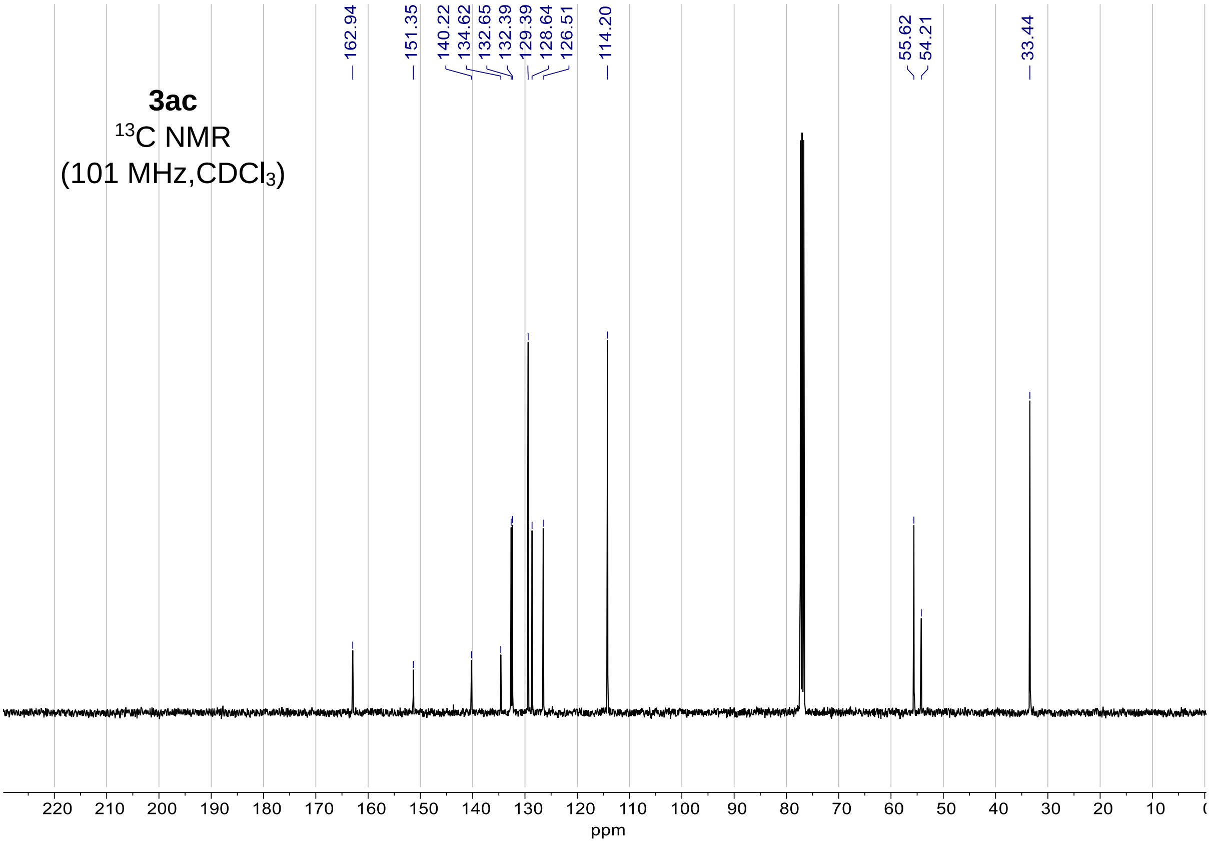
162.94
151.35
140.22 134.62 132.65 132.39 129.39 128.64 126.51
114.20
55.62 54.21
33.44
3ac
^{13}C NMR
(101 MHz,$CDCl_3$)
220 210 200 190 180 170 160 150 140 130 120 110 100 90 80 70 60 50 40 30 20 10
ppm

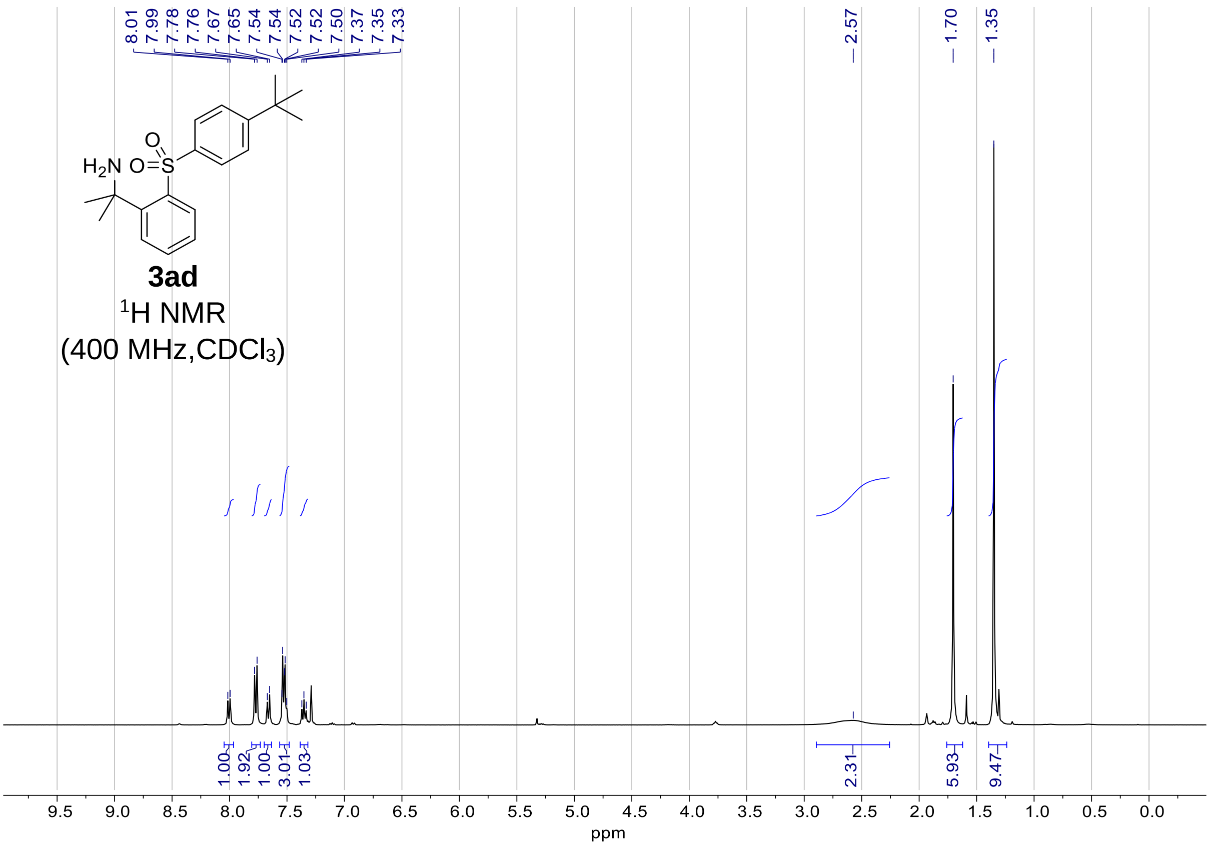
8.01 7.99 7.78 7.76 7.67 7.65 7.54 7.54 7.52 7.52 7.50 7.37 7.35 7.33
2.57
1.70
1.35
H2N O=S O
3ad
1H NMR
(400 MHz,CDCl3)
1.00 1.92 1.00 3.01 1.03
2.31
5.93
9.47
9.5 9.0 8.5 8.0 7.5 7.0 6.5 6.0 5.5 5.0 4.5 4.0 3.5 3.0 2.5 2.0 1.5 1.0 0.5 0.0
ppm

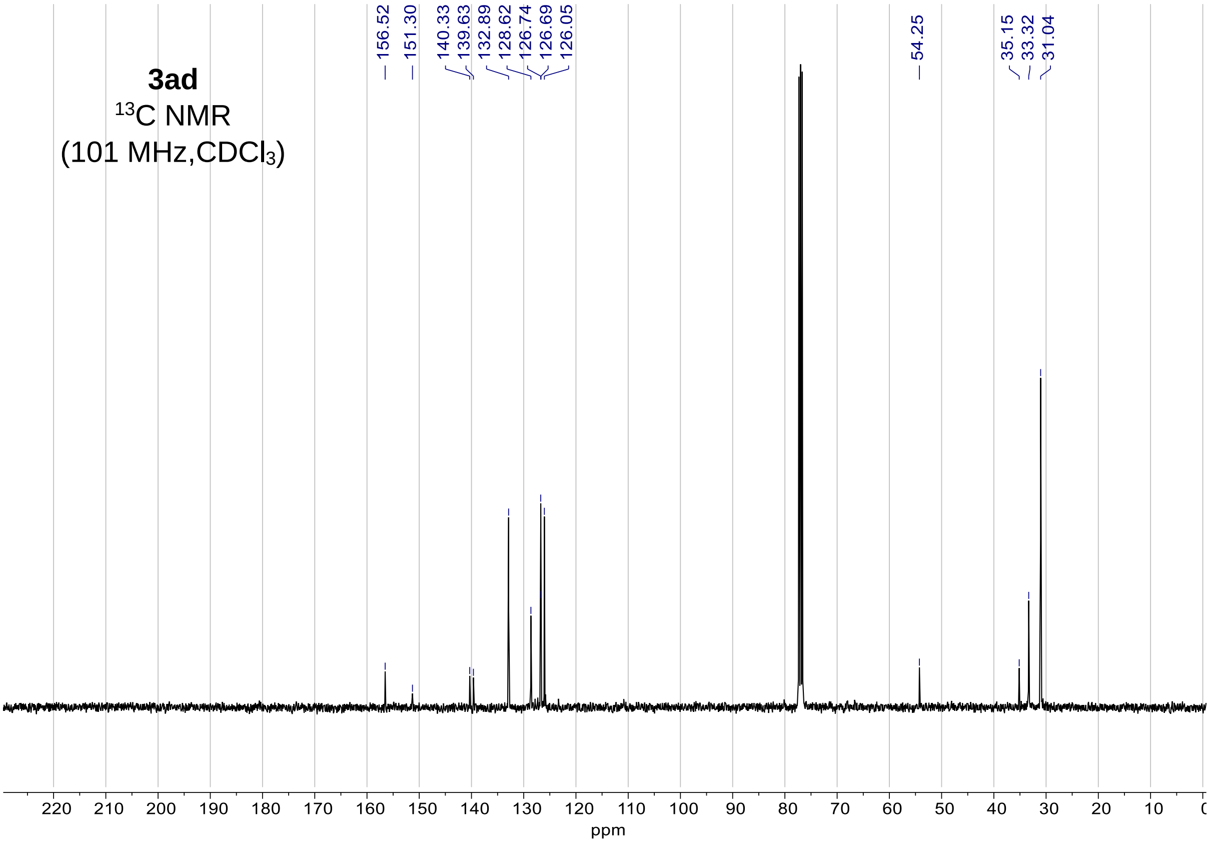
156.52
151.30
140.33 139.63 132.89 128.62 126.74 126.69 126.05
54.25
35.15 33.32 31.04
3ad
13C NMR
(101 MHz,CDCl3)
220 210 200 190 180 170 160 150 140 130 120 110 100 90 80 70 60 50 40 30 20 10 0
ppm

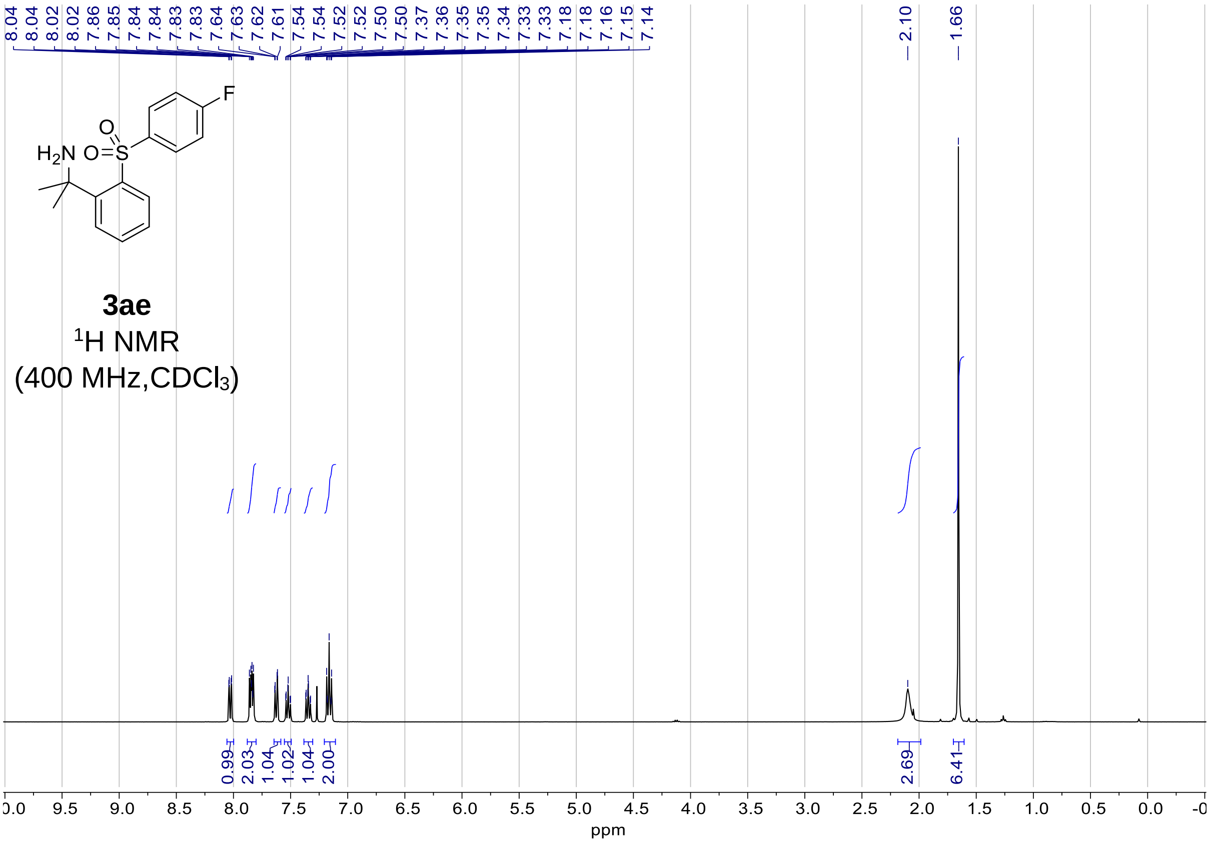
8.04 8.04 8.02 8.02 7.86 7.85 7.84 7.84 7.83 7.83 7.64 7.63 7.62 7.61 7.54 7.54 7.52 7.52 7.50 7.50 7.37 7.36 7.35 7.35 7.34 7.33 7.33 7.18 7.18 7.16 7.15 7.14
2.10
1.66
F
O
H_2N O=S
3ae
^1H NMR
(400 MHz,$CDCl_3$)
0.99 2.03 1.04 1.02 1.04 2.00
2.69
6.41
ppm

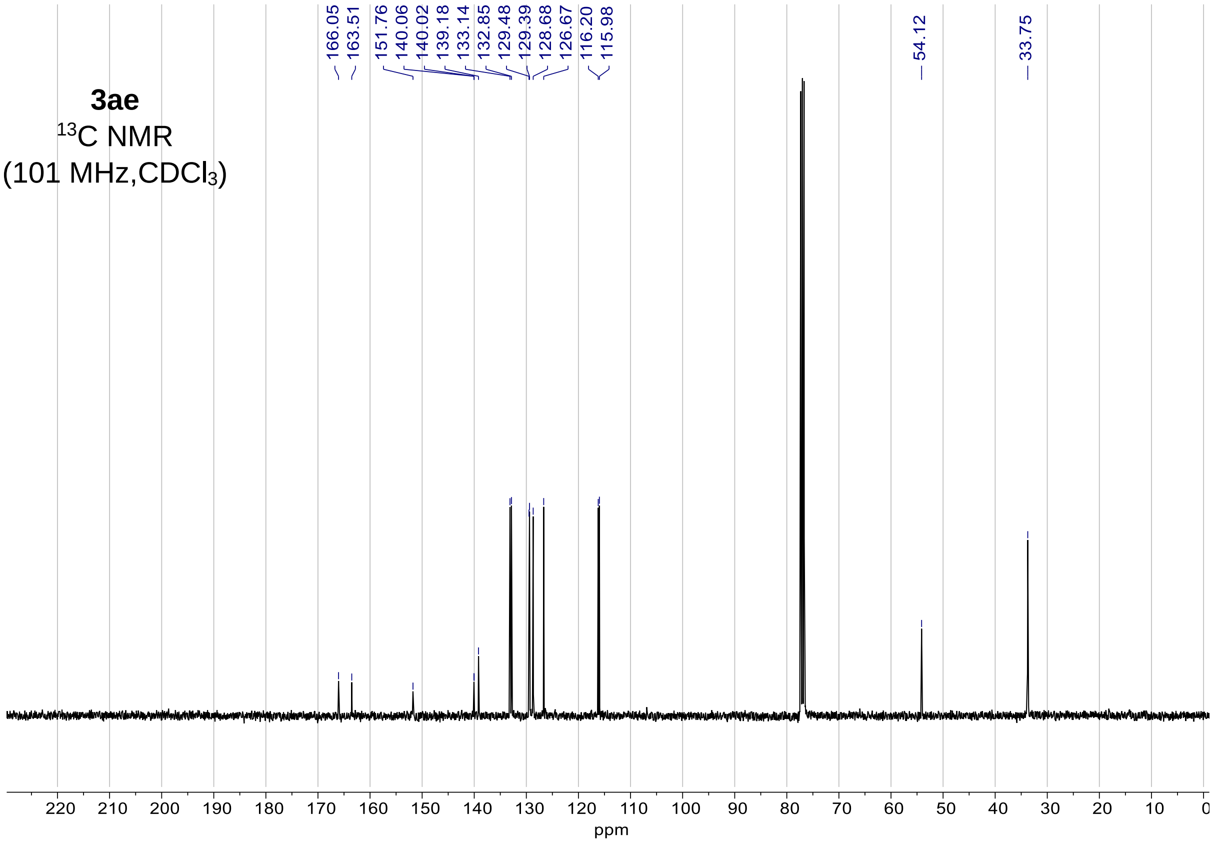
166.05 163.51
151.76 140.06 140.02 139.18 133.14 132.85 129.48 129.39 128.68 126.67 116.20 115.98
54.12
33.75
3ae
^{13}C NMR
(101 MHz,$CDCl_3$)
ppm

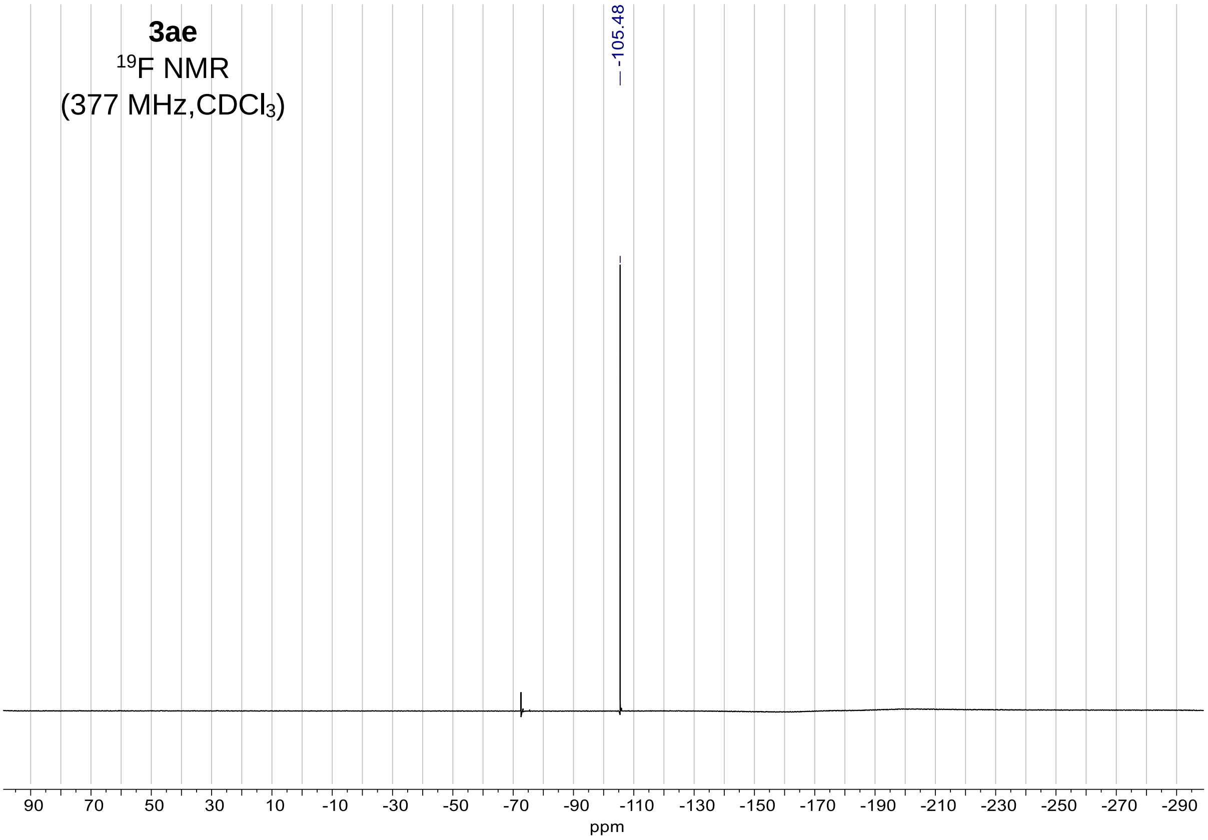
3ae
19F NMR
(377 MHz,CDCl3)
-105.48
90 70 50 30 10 -10 -30 -50 -70 -90 -110 -130 -150 -170 -190 -210 -230 -250 -270 -290
ppm

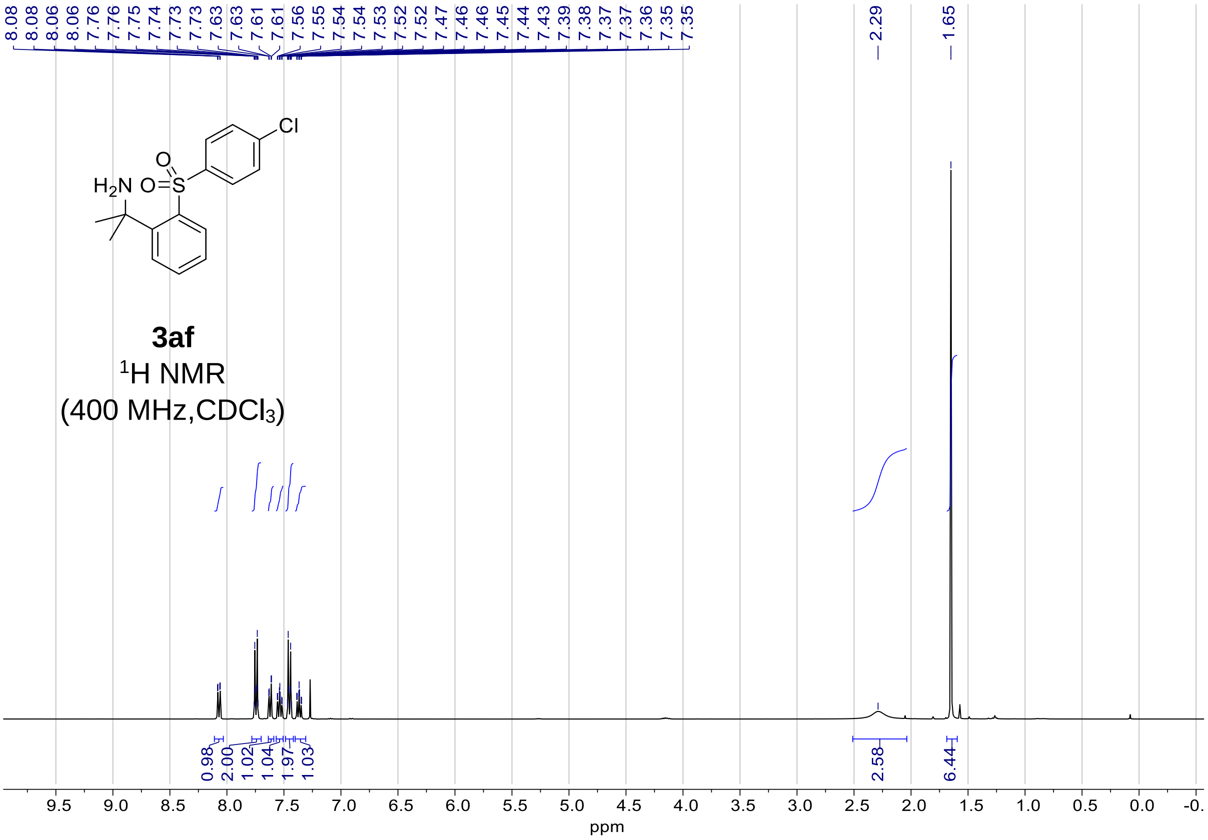

3af
^1H NMR
(400 MHz, $CDCl_3$)

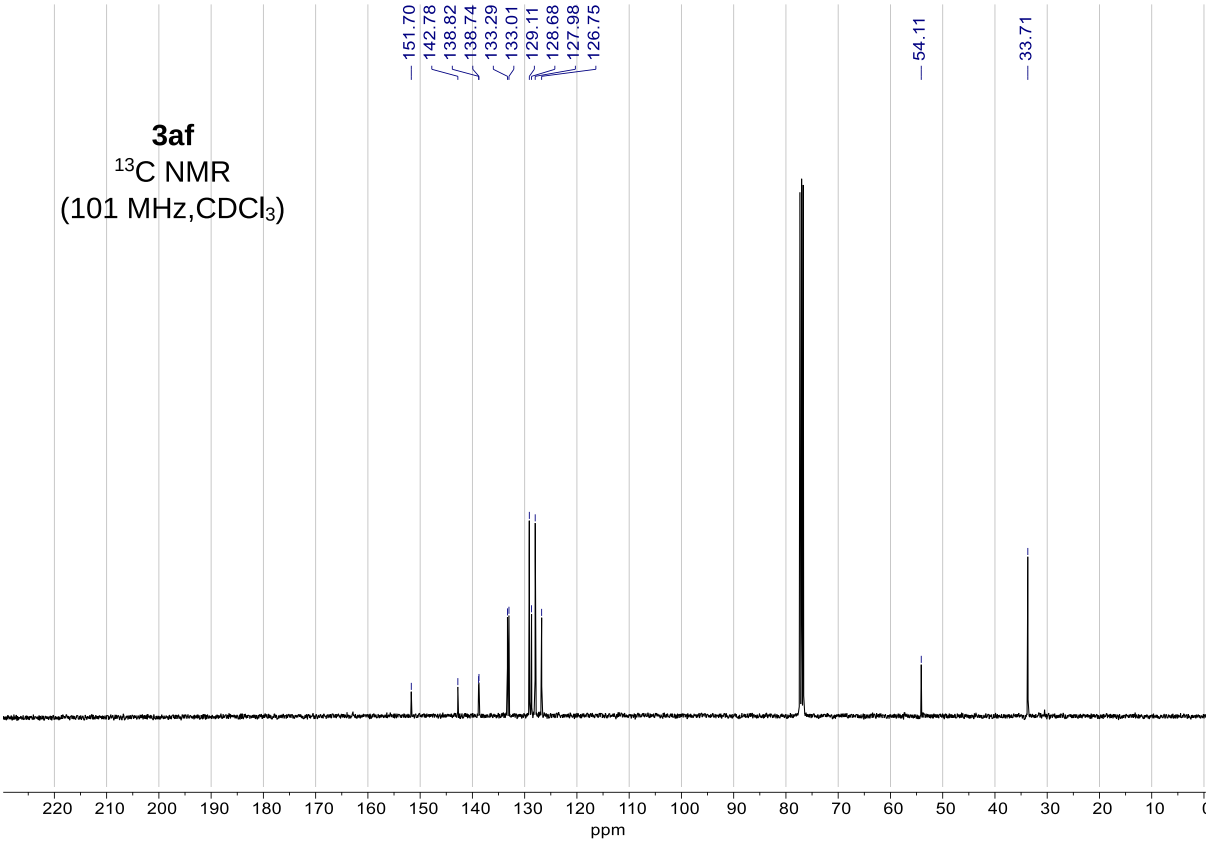

3af
^{13}C NMR
(101 MHz, $CDCl_3$)

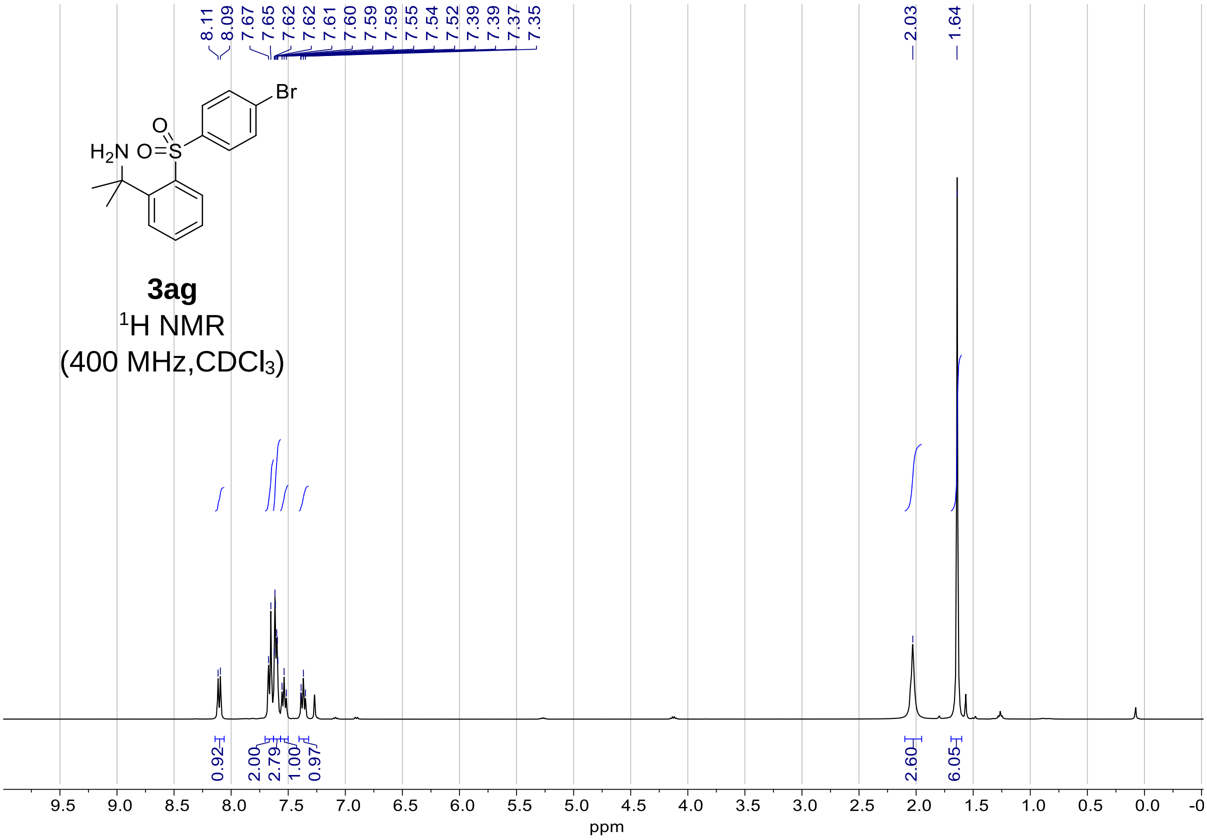

3ag
^{1}H NMR
(400 MHz, $CDCl_3$)

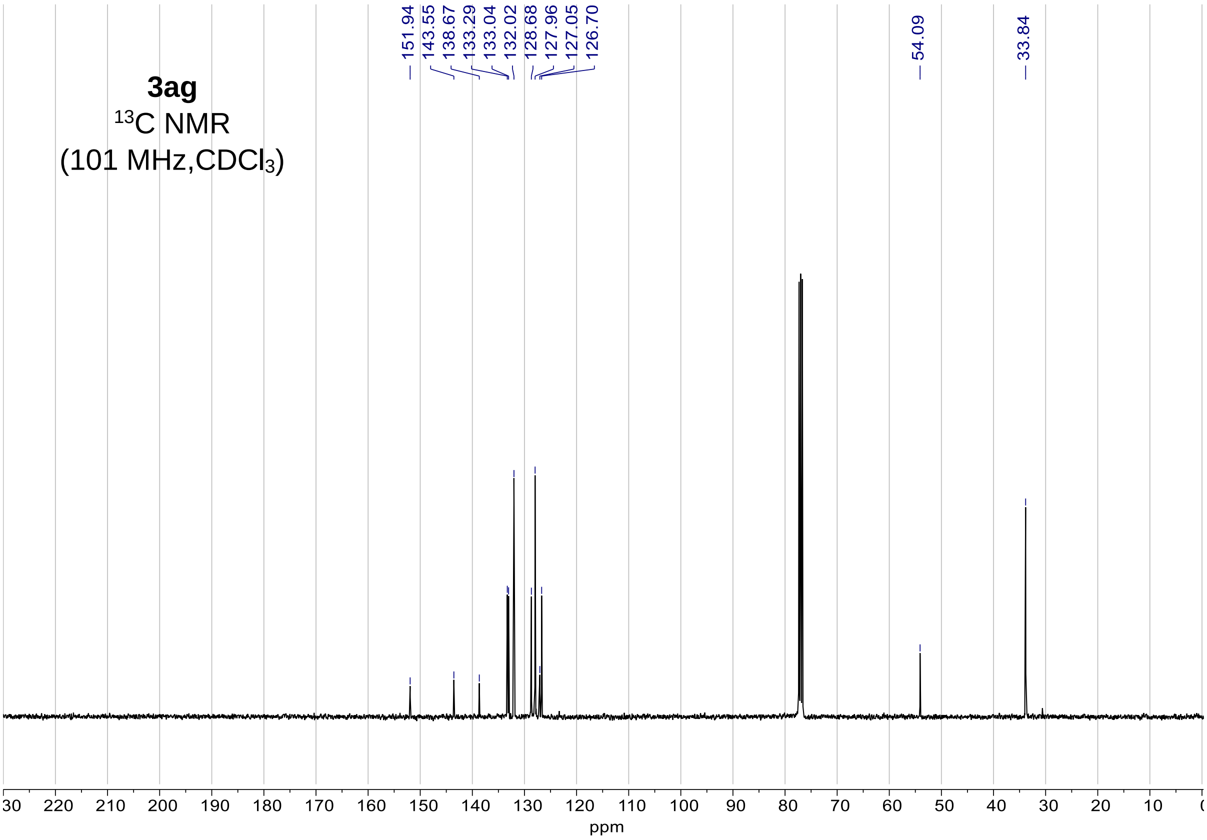

3ag
^{13}C NMR
(101 MHz, $CDCl_3$)

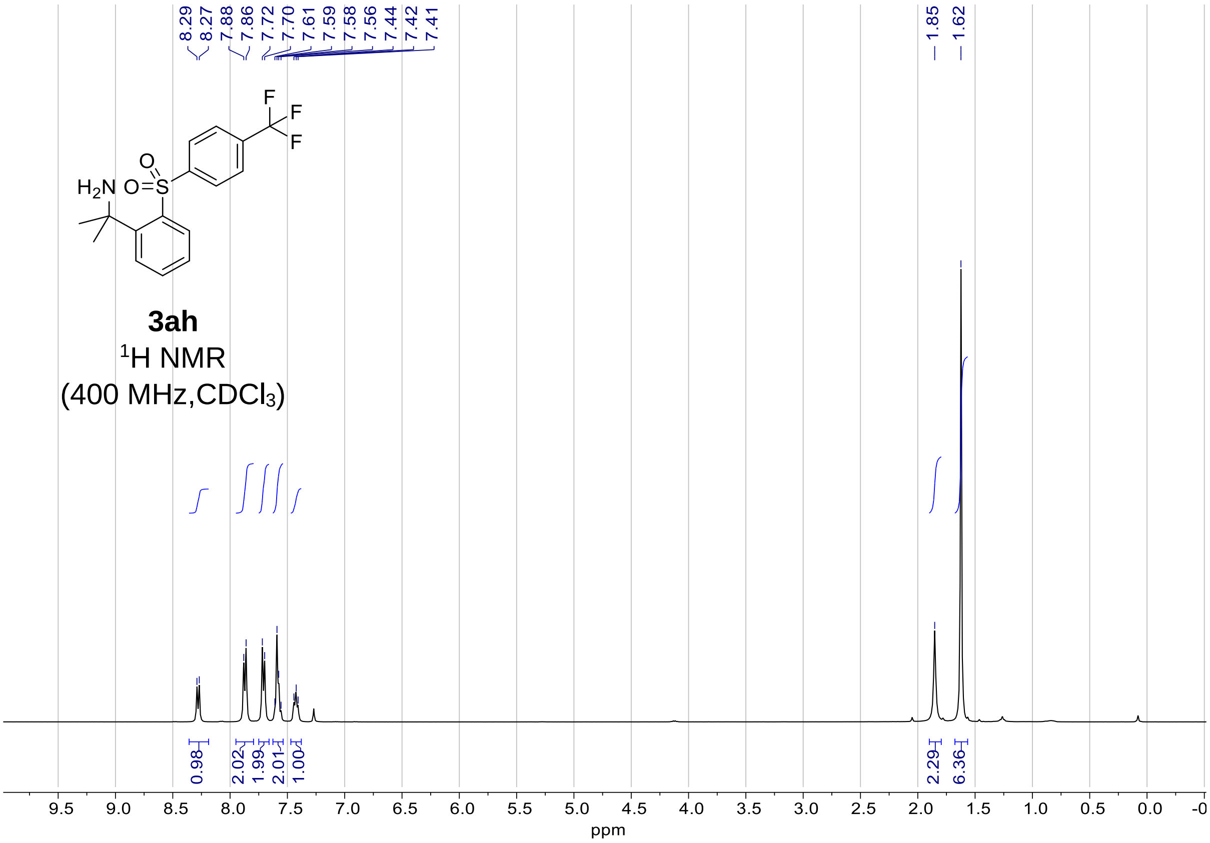

3ah
^{1}H NMR
(400 MHz, $CDCl_3$)

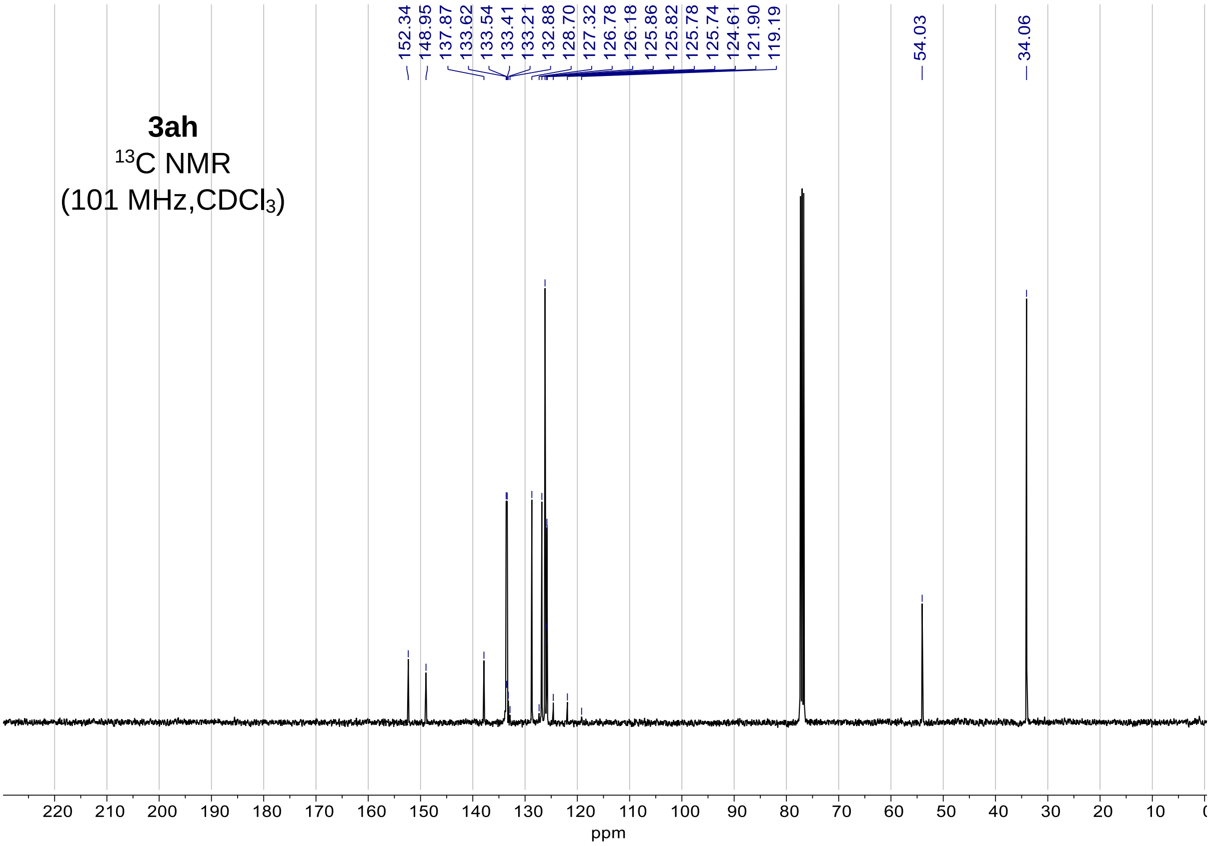

3ah
^{13}C NMR
(101 MHz, $CDCl_3$)

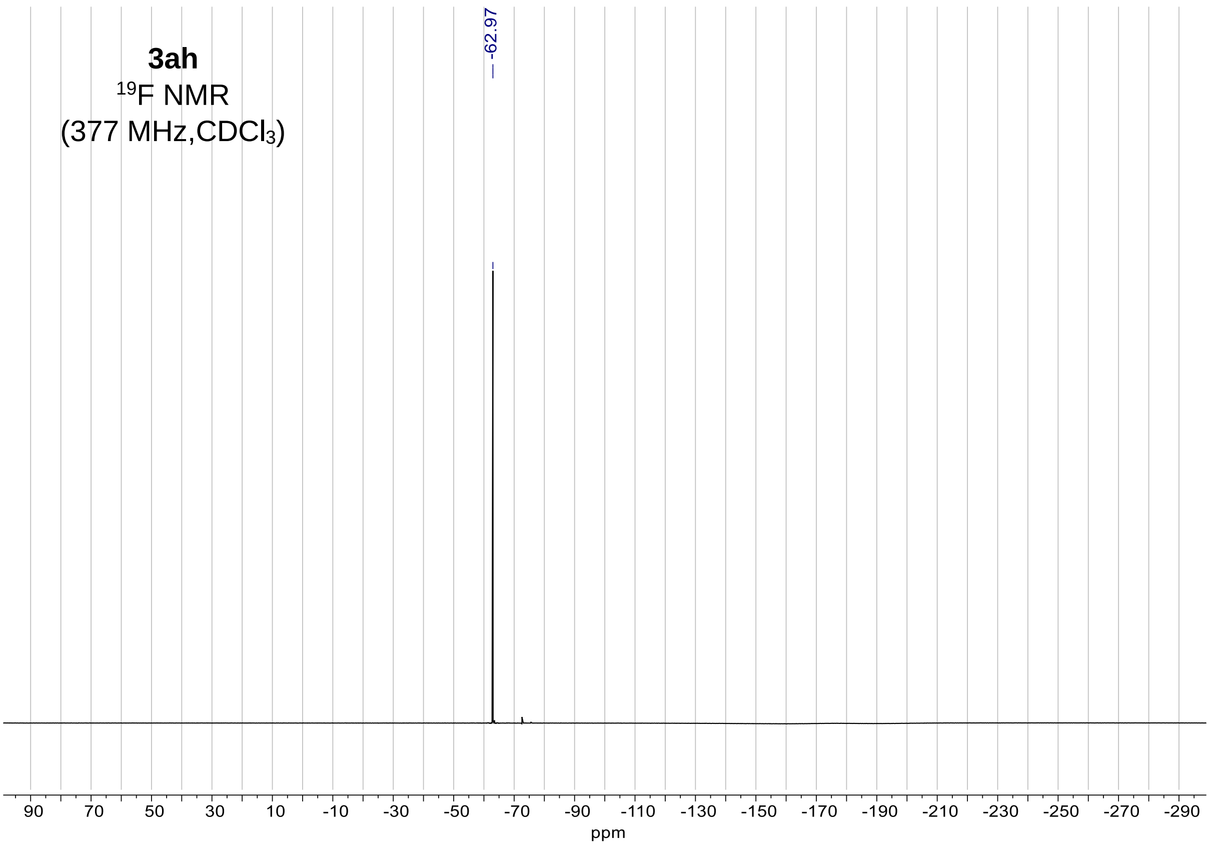
3ah
19F NMR
(377 MHz,CDCl3)
-62.97
90 70 50 30 10 -10 -30 -50 -70 -90 -110 -130 -150 -170 -190 -210 -230 -250 -270 -290
ppm

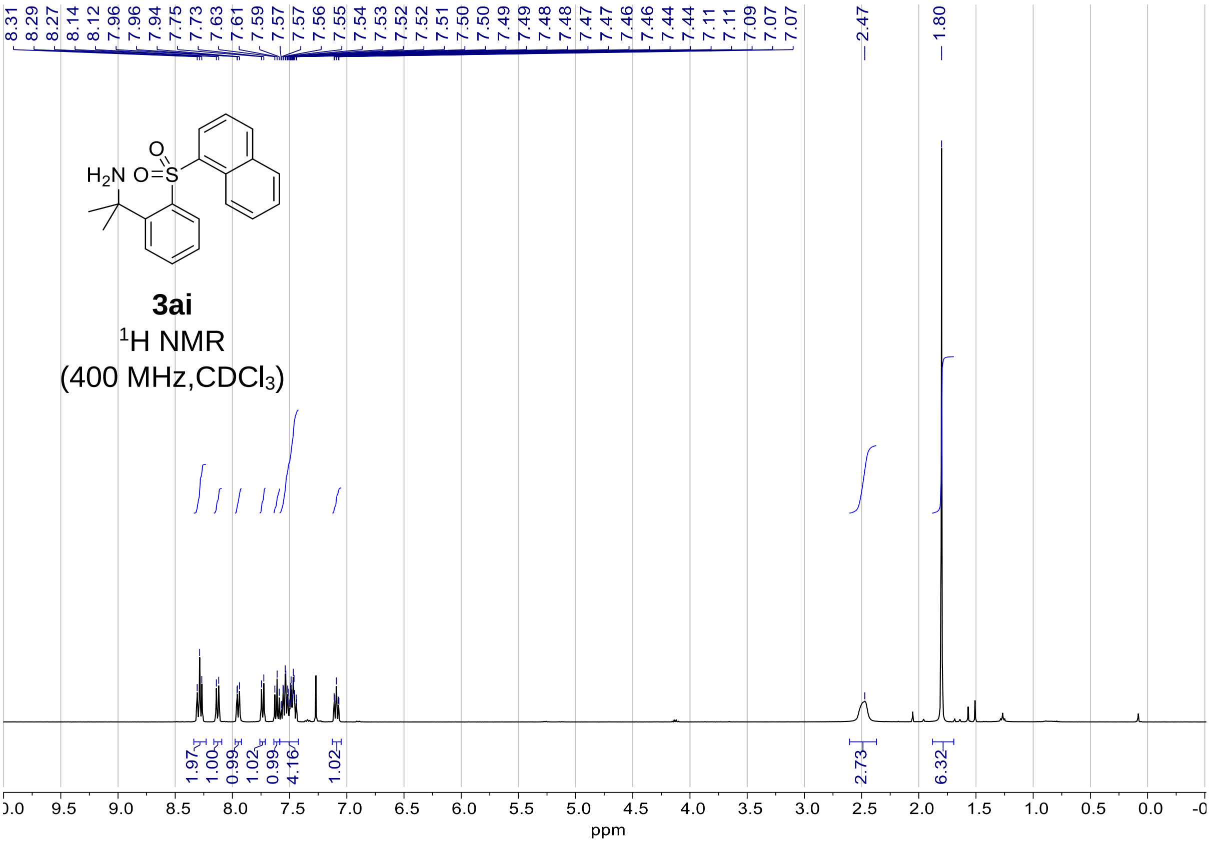
3ai
^{1}H NMR
(400 MHz,CDCl$_3$)
8.31 8.29 8.27 8.14 8.12 7.96 7.96 7.94 7.75 7.73 7.63 7.61 7.59 7.57 7.57 7.56 7.55 7.54 7.53 7.52 7.52 7.51 7.50 7.50 7.49 7.49 7.48 7.48 7.47 7.47 7.46 7.44 7.44 7.11 7.11 7.09 7.07 7.07
2.47
1.80
1.97 1.00 0.99 1.02 0.99 4.16 1.02 2.73 6.32
ppm

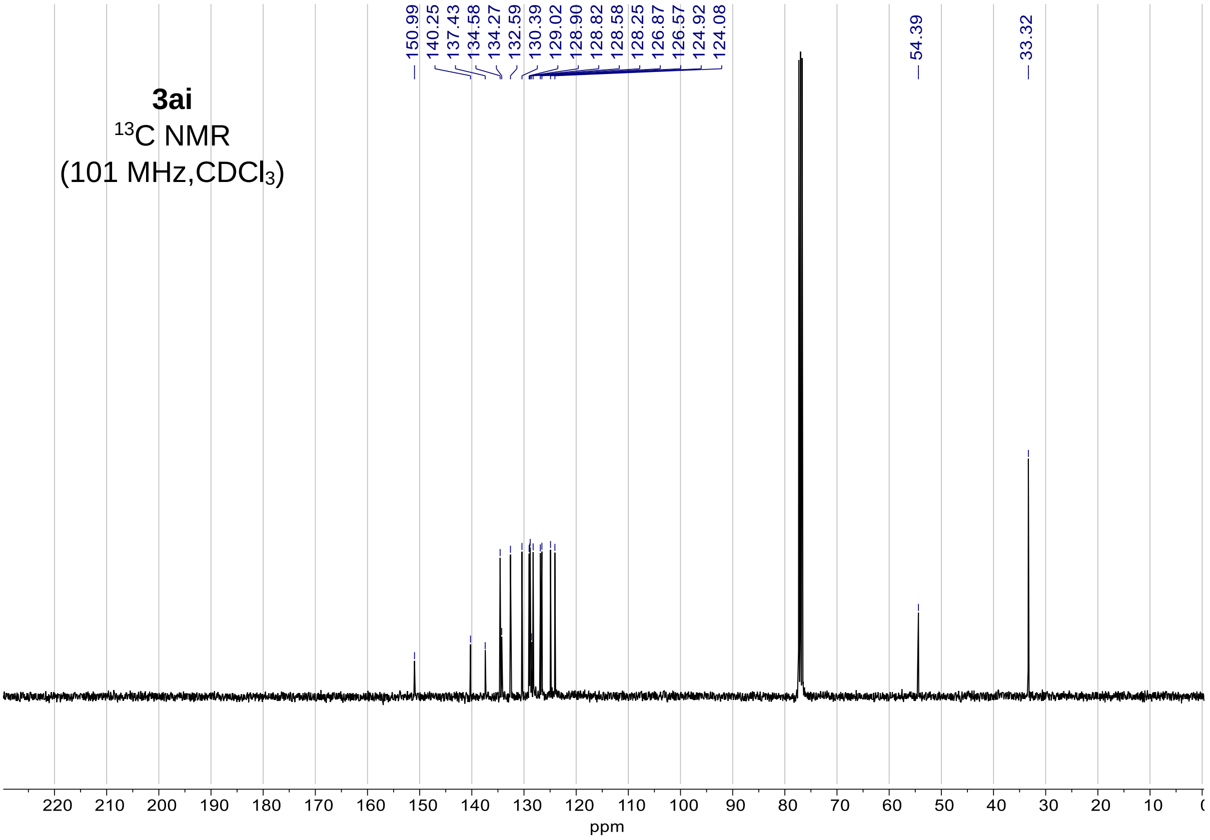
3ai
^{13}C NMR
(101 MHz,CDCl$_3$)
150.99 140.25 137.43 134.58 134.27 132.59 130.39 129.02 128.90 128.82 128.58 128.25 126.87 126.57 124.92 124.08
54.39
33.32
ppm

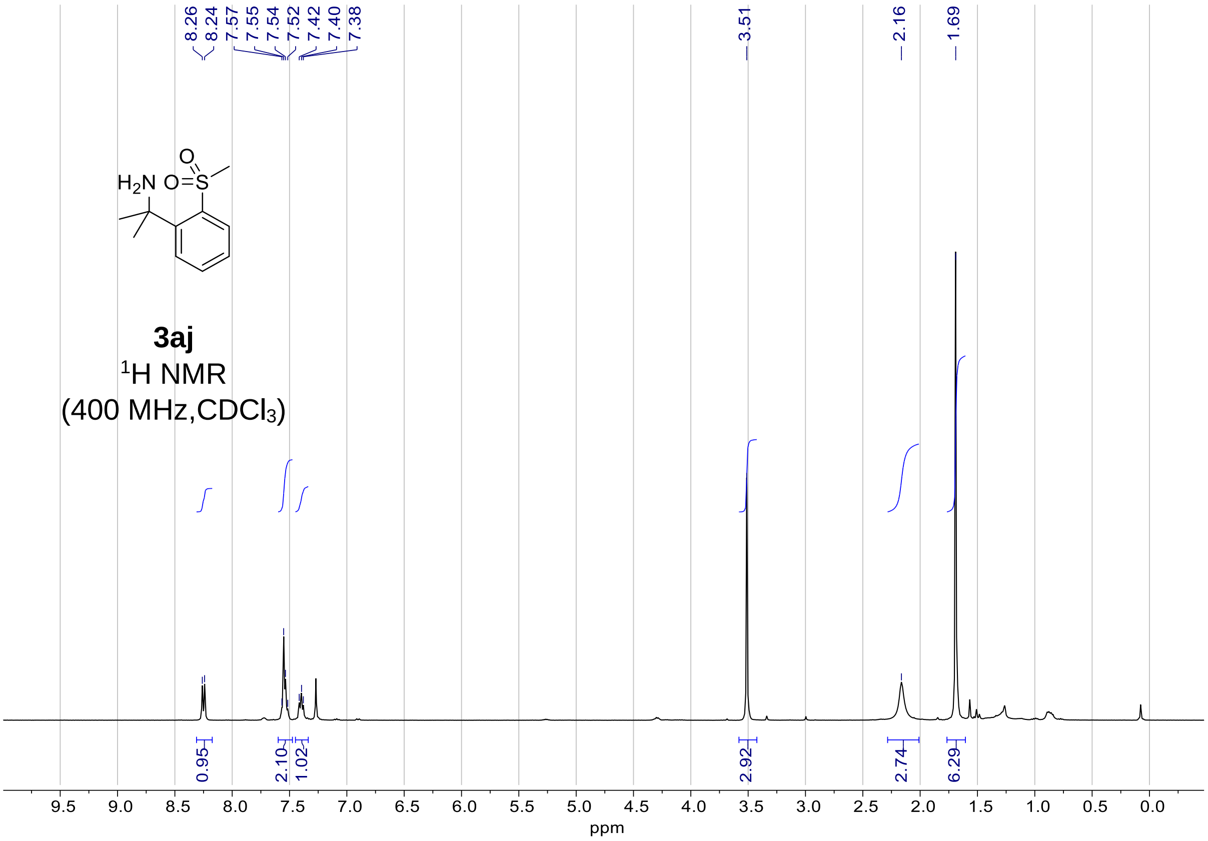
8.26
8.24
7.57
7.55
7.54
7.52
7.42
7.40
7.38
3.51
2.16
1.69
H2N O=S O
3aj
1H NMR
(400 MHz,CDCl3)
0.95
2.10
1.02
2.92
2.74
6.29
9.5 9.0 8.5 8.0 7.5 7.0 6.5 6.0 5.5 5.0 4.5 4.0 3.5 3.0 2.5 2.0 1.5 1.0 0.5 0.0
ppm

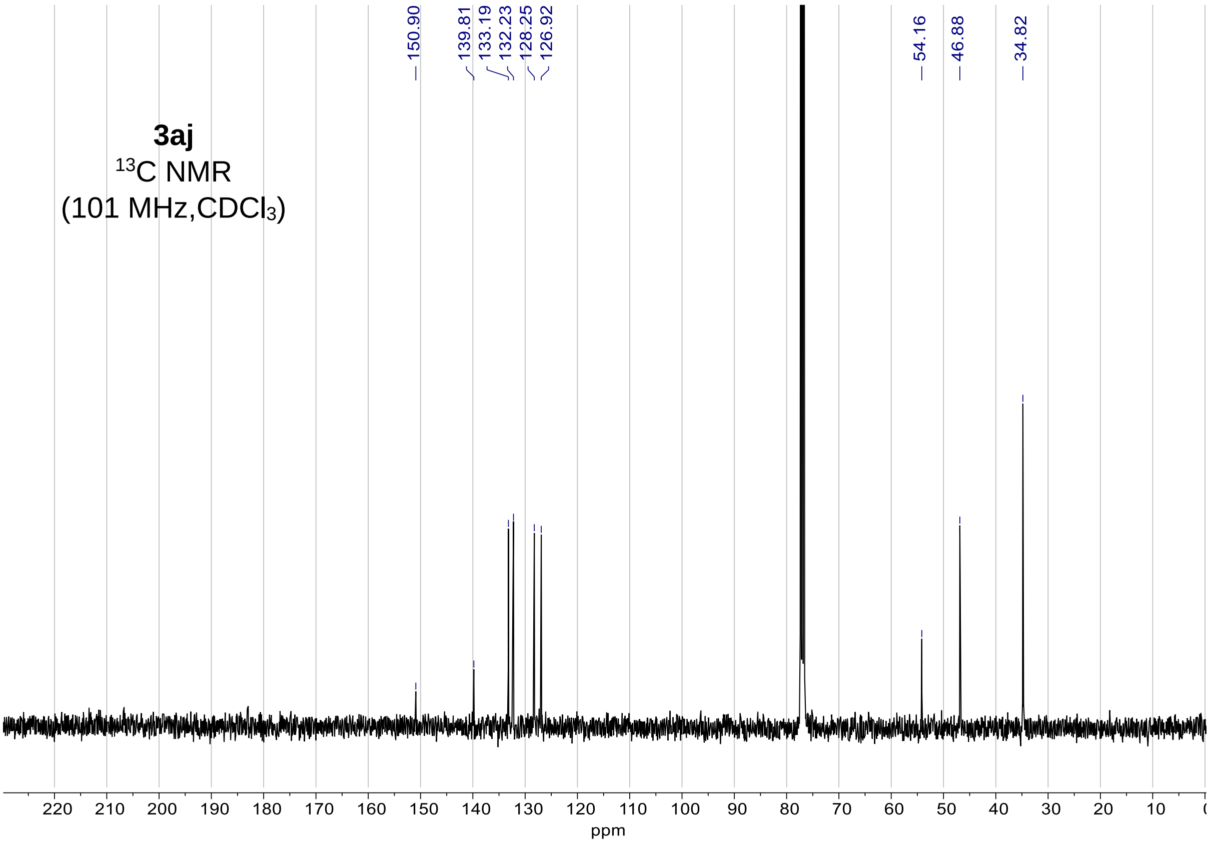
150.90
139.81
133.19
132.23
128.25
126.92
54.16
46.88
34.82
3aj
13C NMR
(101 MHz,CDCl3)
220 210 200 190 180 170 160 150 140 130 120 110 100 90 80 70 60 50 40 30 20 10
ppm

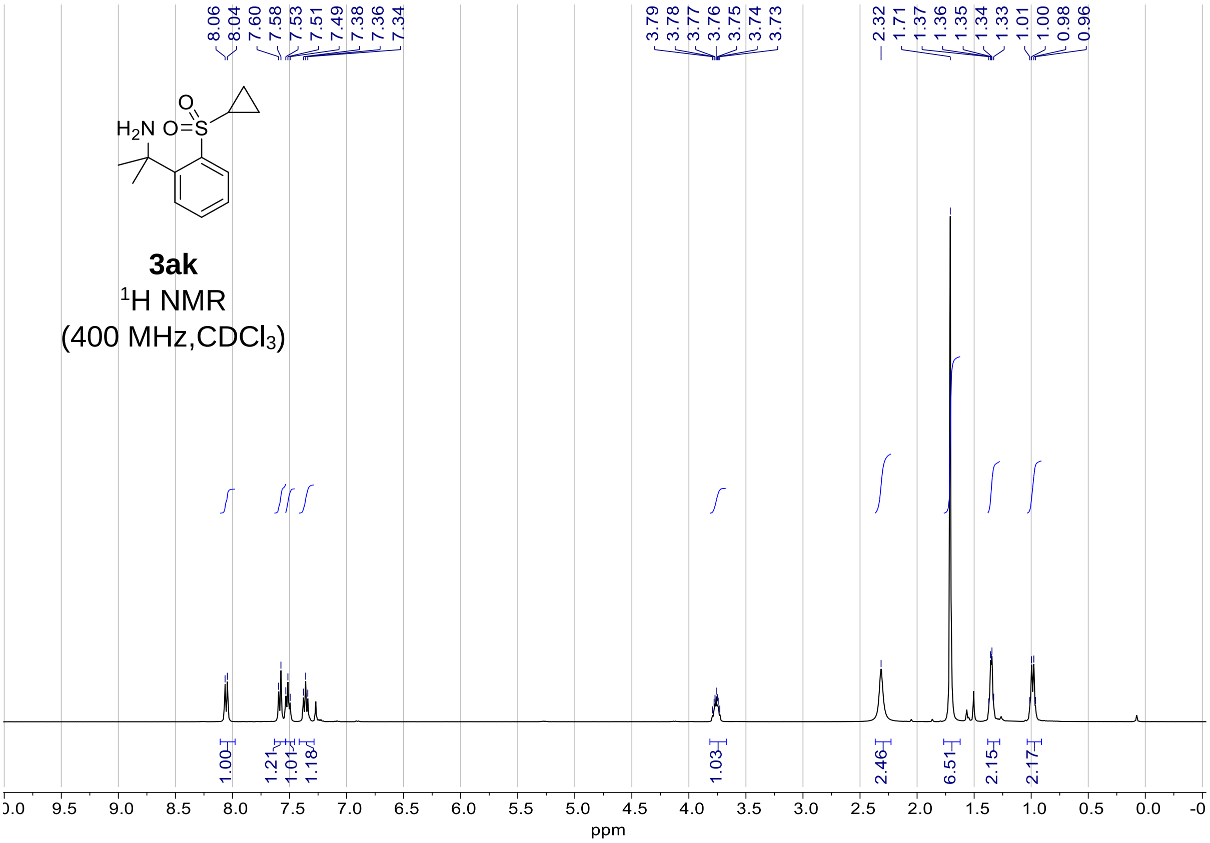
8.06
8.04
7.60
7.58
7.53
7.51
7.49
7.38
7.36
7.34
3.79
3.78
3.77
3.76
3.75
3.74
3.73
2.32
1.71
1.37
1.36
1.35
1.34
1.33
1.01
1.00
0.98
0.96
H2N O=S
O
3ak
1H NMR
(400 MHz,CDCl3)
1.00
1.21
1.01
1.18
1.03
2.46
6.51
2.15
2.17
ppm

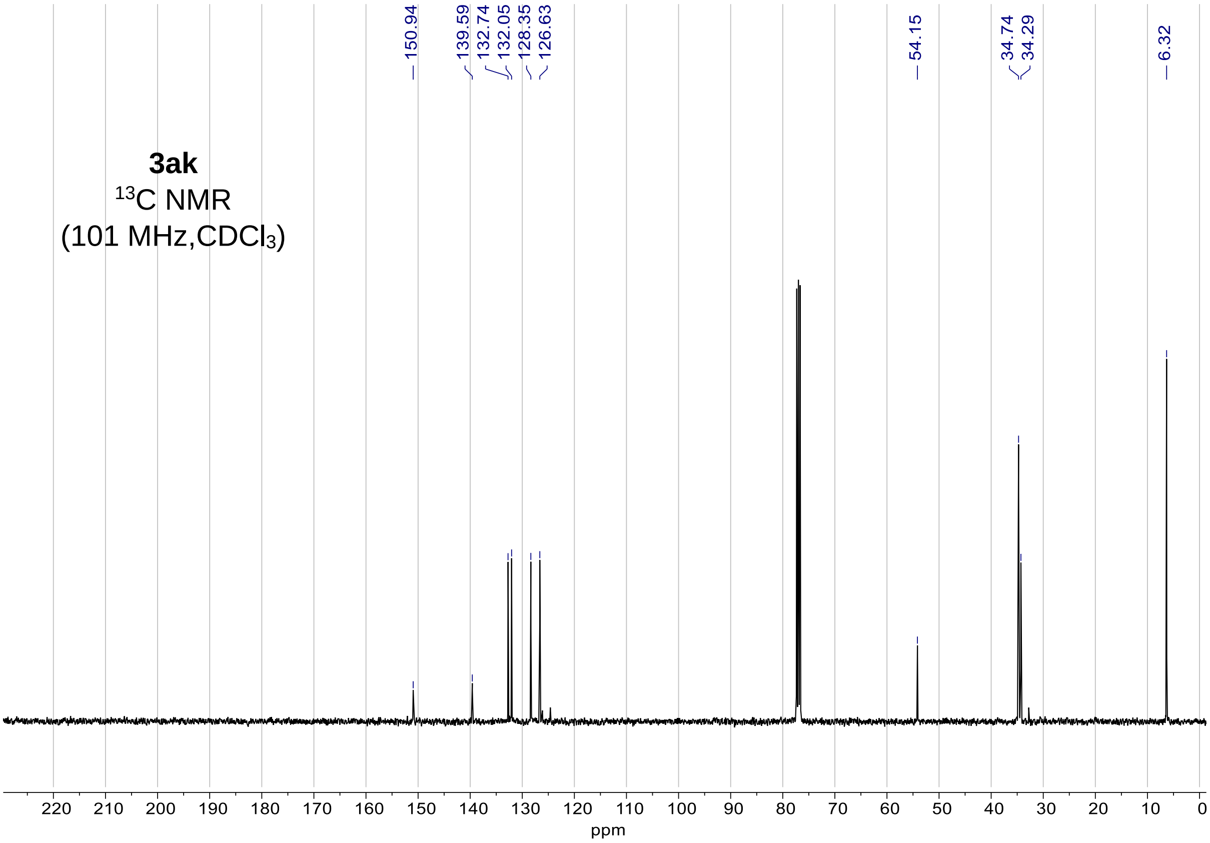
150.94
139.59
132.74
132.05
128.35
126.63
54.15
34.74
34.29
6.32
3ak
13C NMR
(101 MHz,CDCl3)
ppm

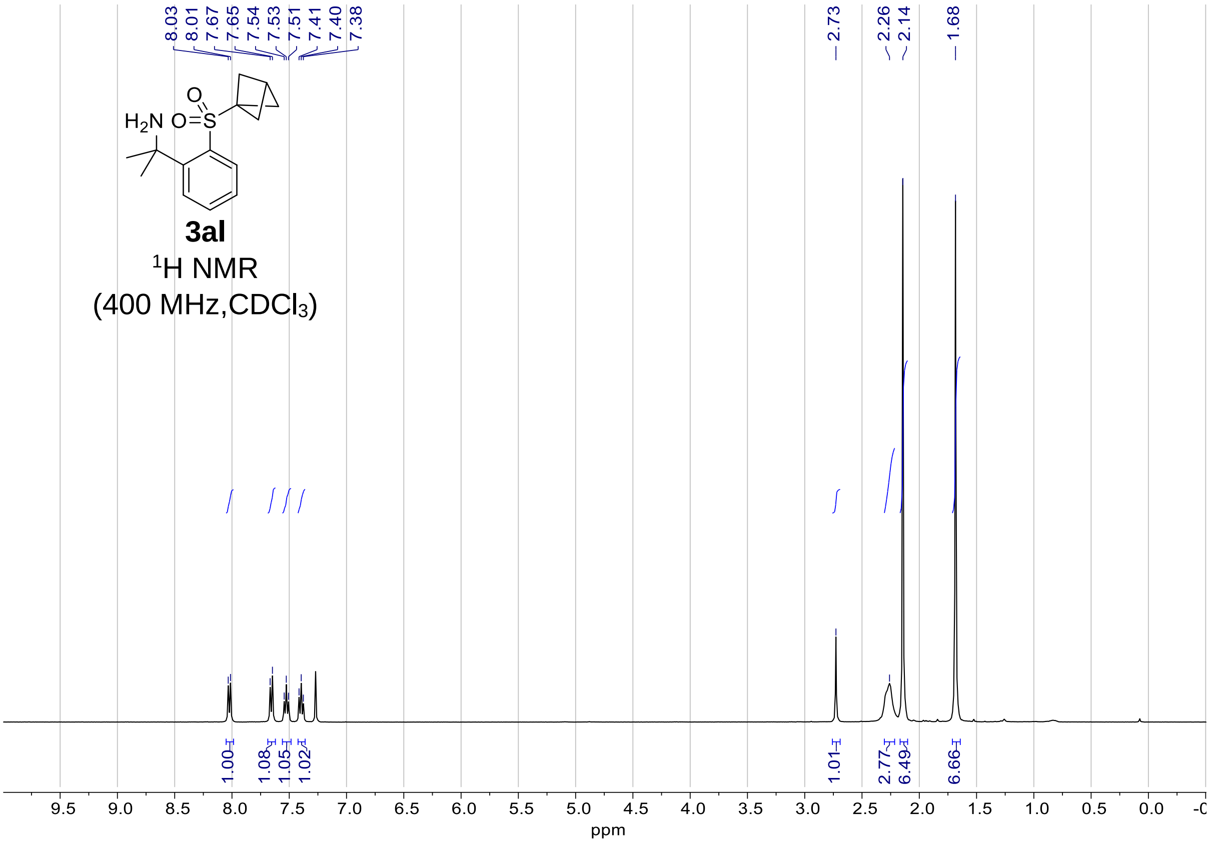
8.03 8.01 7.67 7.65 7.54 7.53 7.51 7.41 7.40 7.38
2.73
2.26 2.14
1.68
H2N
O
O=S
3al
1H NMR
(400 MHz,CDCl3)
1.00 1.08 1.05 1.02
1.01
2.77 6.49
6.66
9.5 9.0 8.5 8.0 7.5 7.0 6.5 6.0 5.5 5.0 4.5 4.0 3.5 3.0 2.5 2.0 1.5 1.0 0.5 0.0
ppm

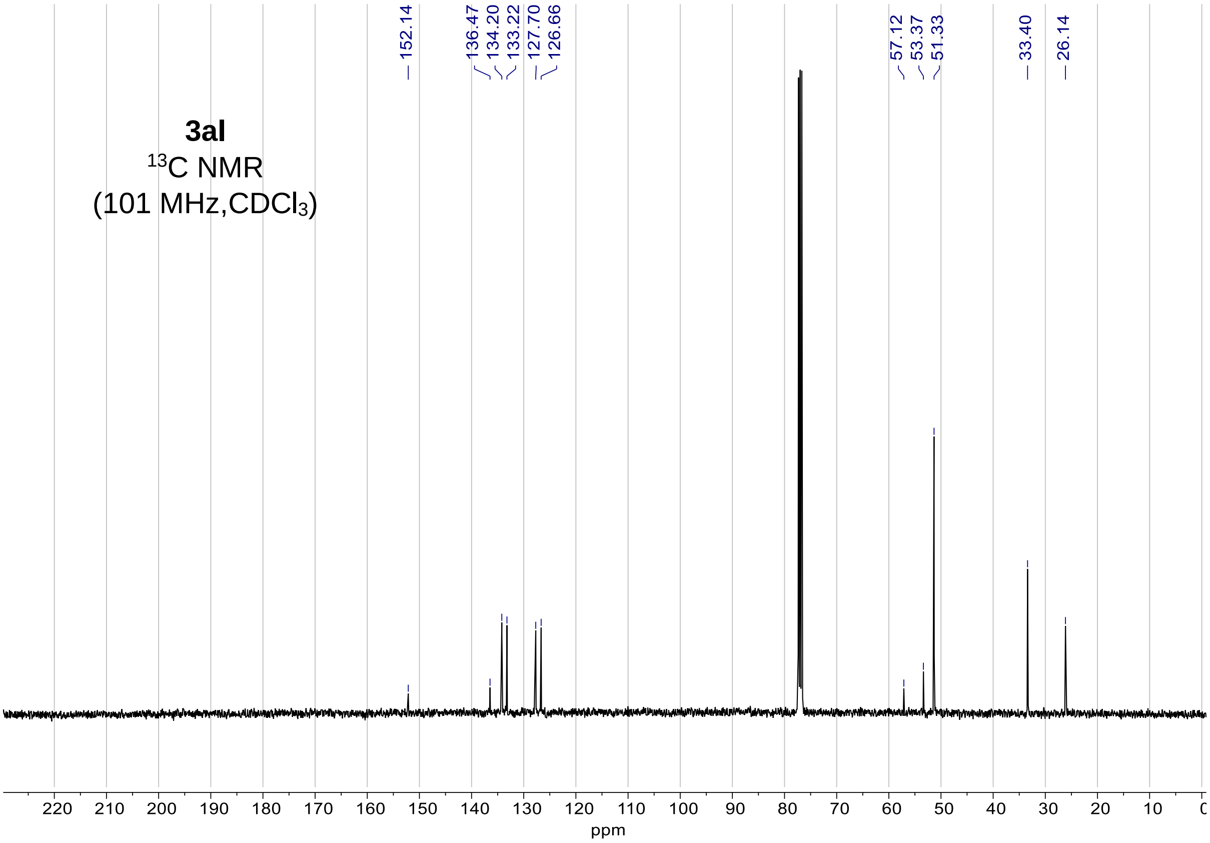
152.14
136.47 134.20 133.22 127.70 126.66
57.12 53.37 51.33
33.40
26.14
3al
13C NMR
(101 MHz,CDCl3)
220 210 200 190 180 170 160 150 140 130 120 110 100 90 80 70 60 50 40 30 20 10
ppm

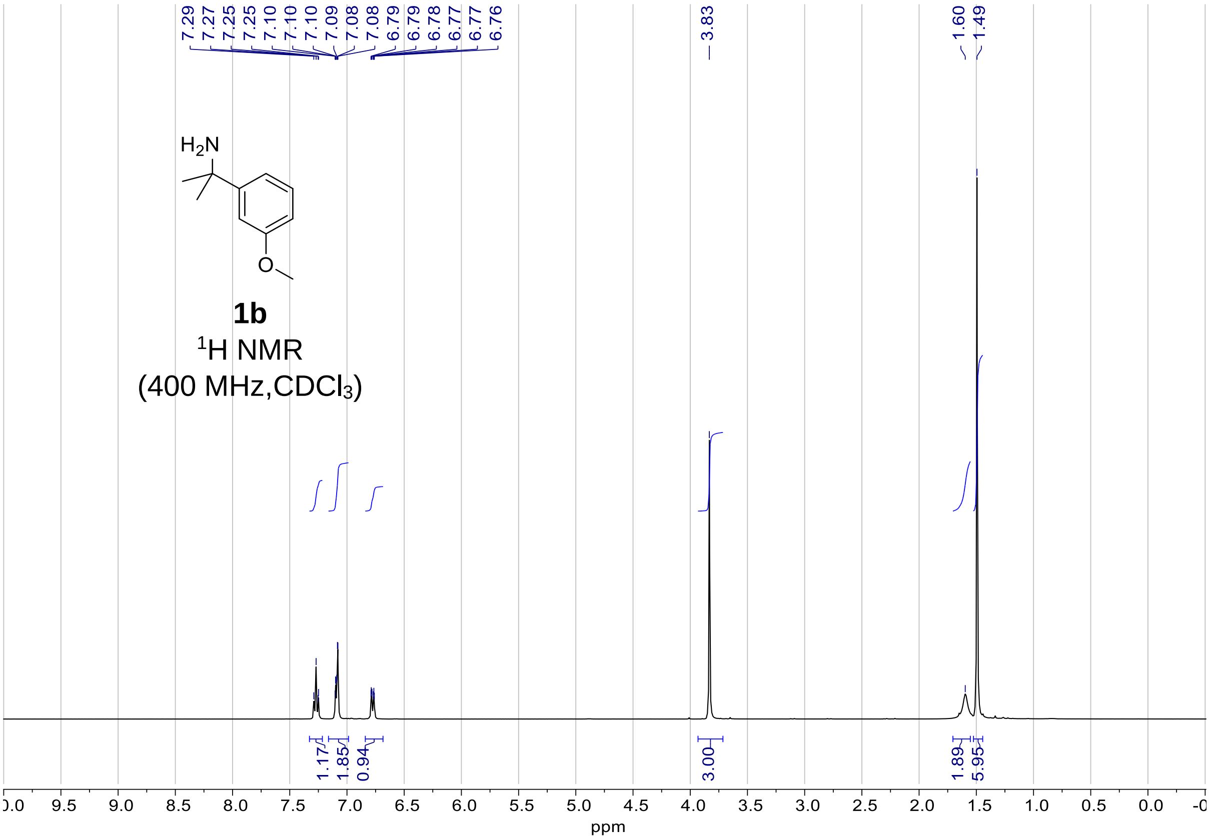
H2N
O
1b
1H NMR
(400 MHz,CDCl3)
7.29 7.27 7.25 7.25 7.10 7.10 7.10 7.09 7.08 7.08 6.79 6.79 6.78 6.77 6.77 6.76
3.83
1.60 1.49
1.17 1.85 0.94 3.00 1.89 5.95
ppm

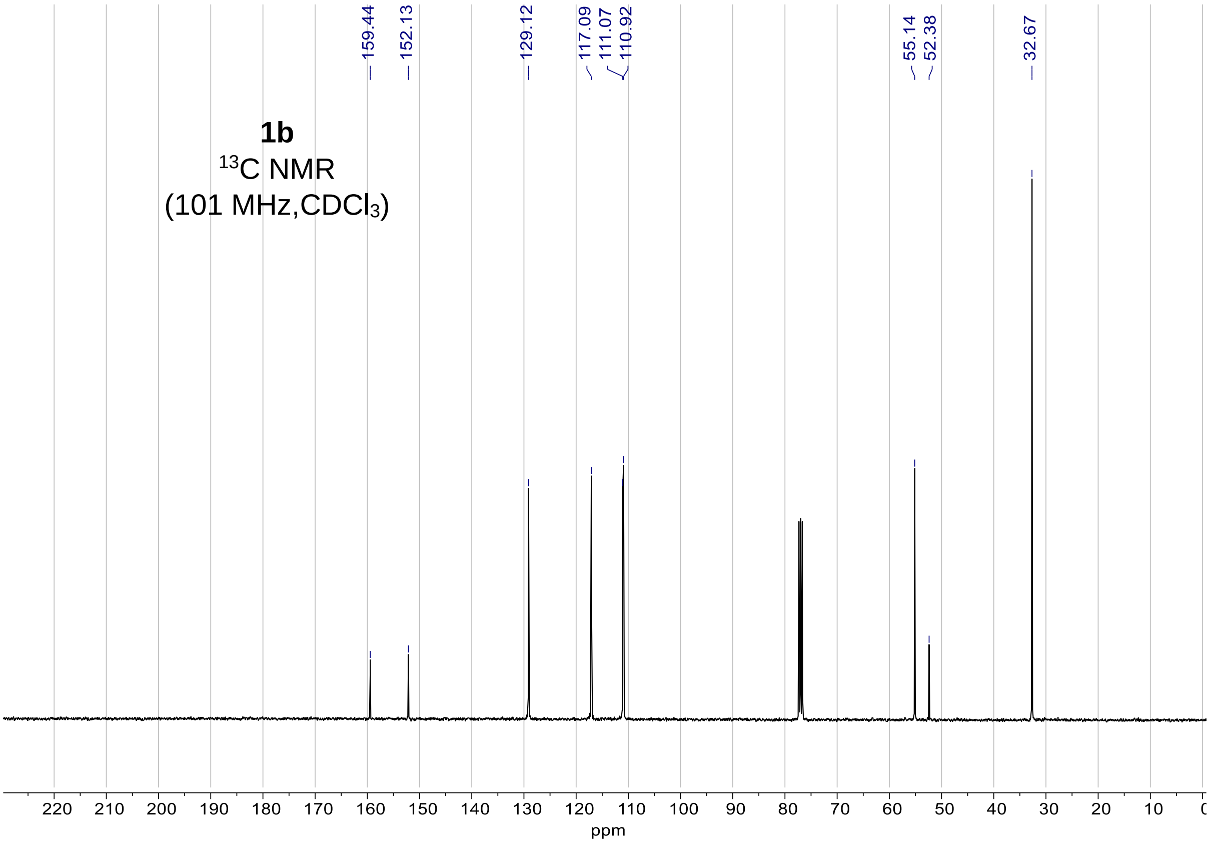
1b
13C NMR
(101 MHz,CDCl3)
159.44 152.13 129.12 117.09 111.07 110.92 55.14 52.38 32.67
ppm

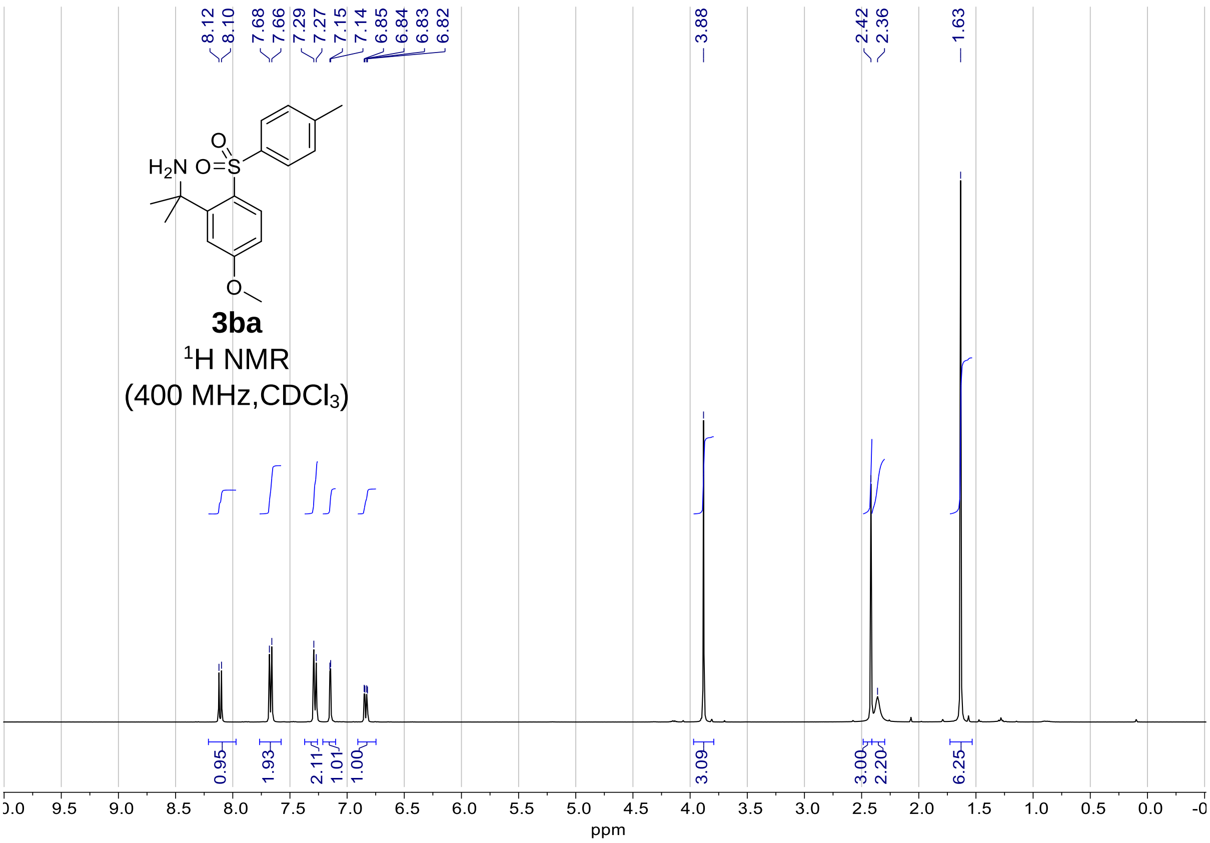
8.12
8.10
7.68
7.66
7.29
7.27
7.15
7.14
6.85
6.84
6.83
6.82
3.88
2.42
2.36
1.63
H2N O=S O
O
3ba
1H NMR
(400 MHz,CDCl3)
0.95
1.93
2.11
1.01
1.00
3.09
3.00
2.20
6.25
ppm

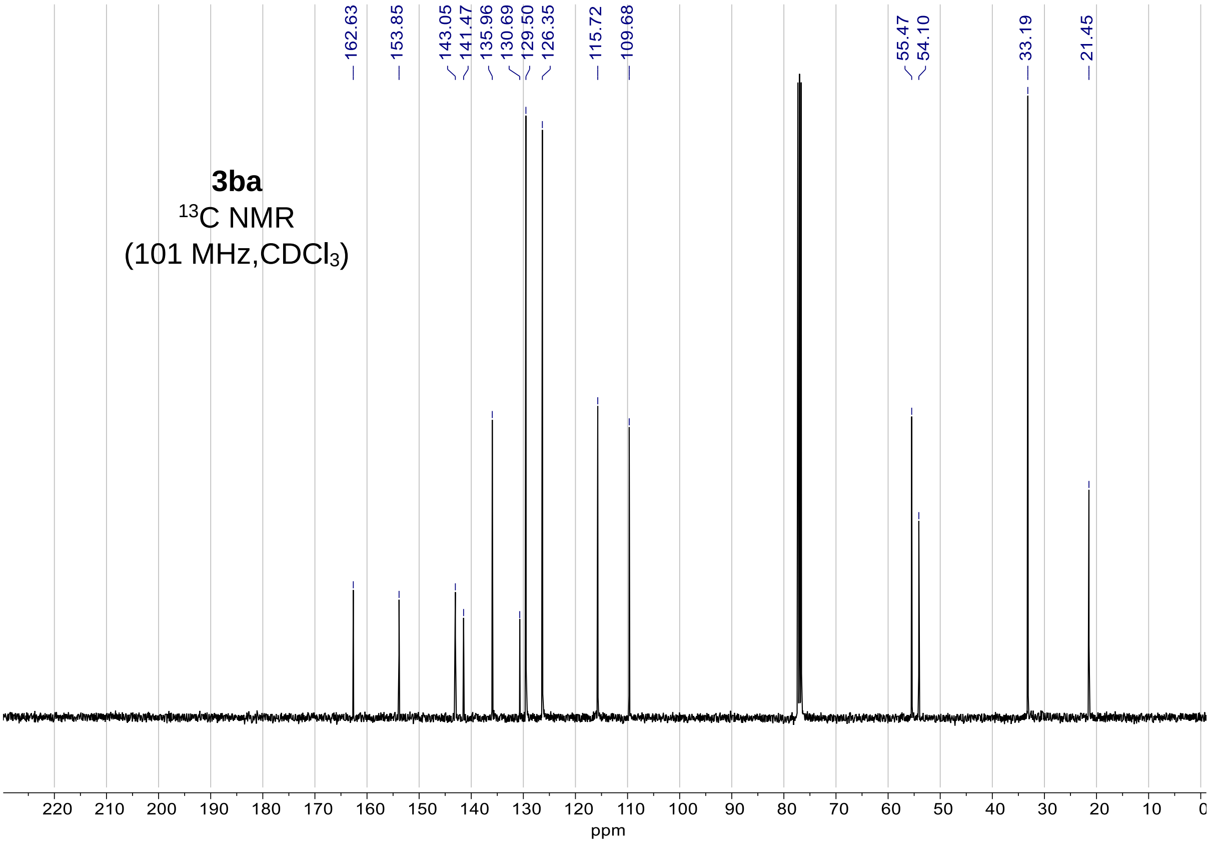
162.63
153.85
143.05
141.47
135.96
130.69
129.50
126.35
115.72
109.68
55.47
54.10
33.19
21.45
3ba
13C NMR
(101 MHz,CDCl3)
ppm

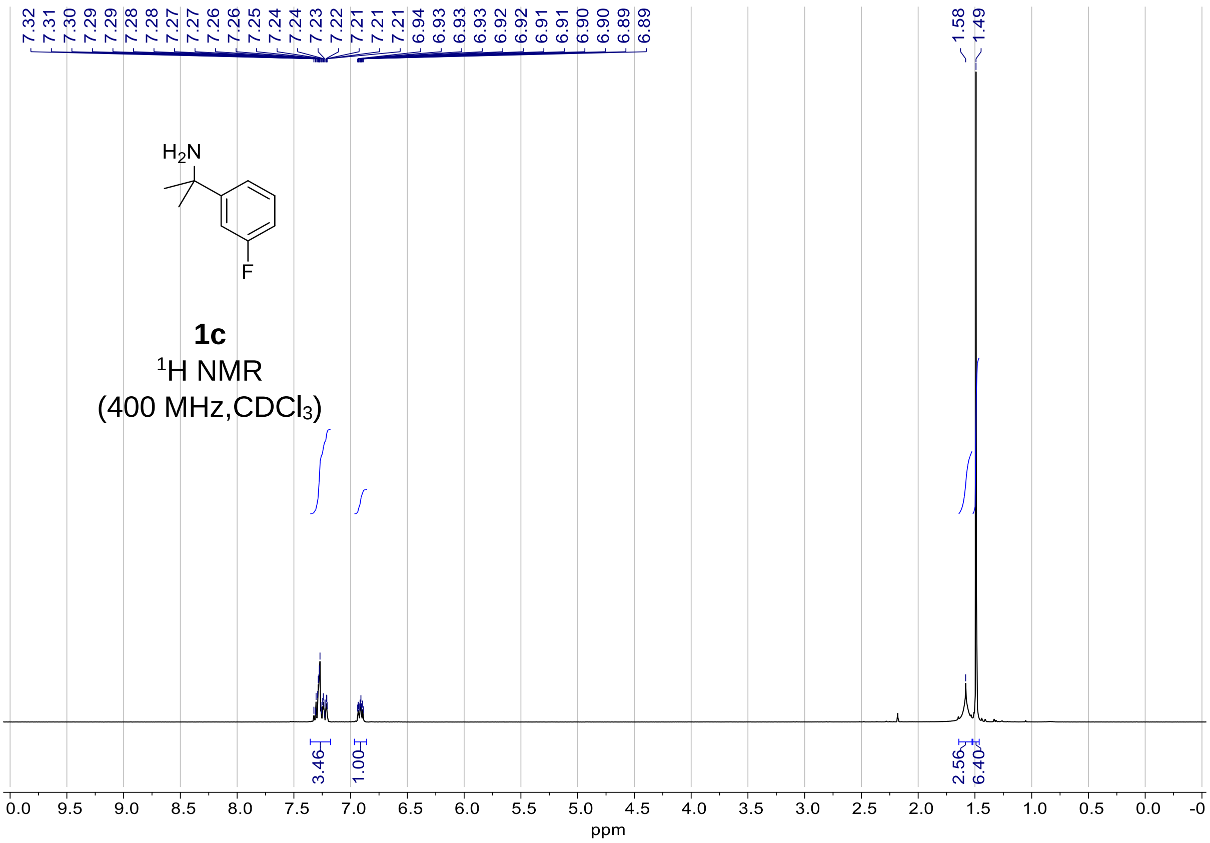
7.32 7.31 7.30 7.29 7.29 7.28 7.28 7.27 7.27 7.26 7.26 7.25 7.24 7.24 7.23 7.22 7.21 7.21 7.21 6.94 6.93 6.93 6.93 6.92 6.92 6.91 6.91 6.90 6.90 6.89 6.89
1.58 1.49
H2N
F
1c
1H NMR
(400 MHz,CDCl3)
3.46
1.00
2.56
6.40
0.0 9.5 9.0 8.5 8.0 7.5 7.0 6.5 6.0 5.5 5.0 4.5 4.0 3.5 3.0 2.5 2.0 1.5 1.0 0.5 0.0 -0
ppm

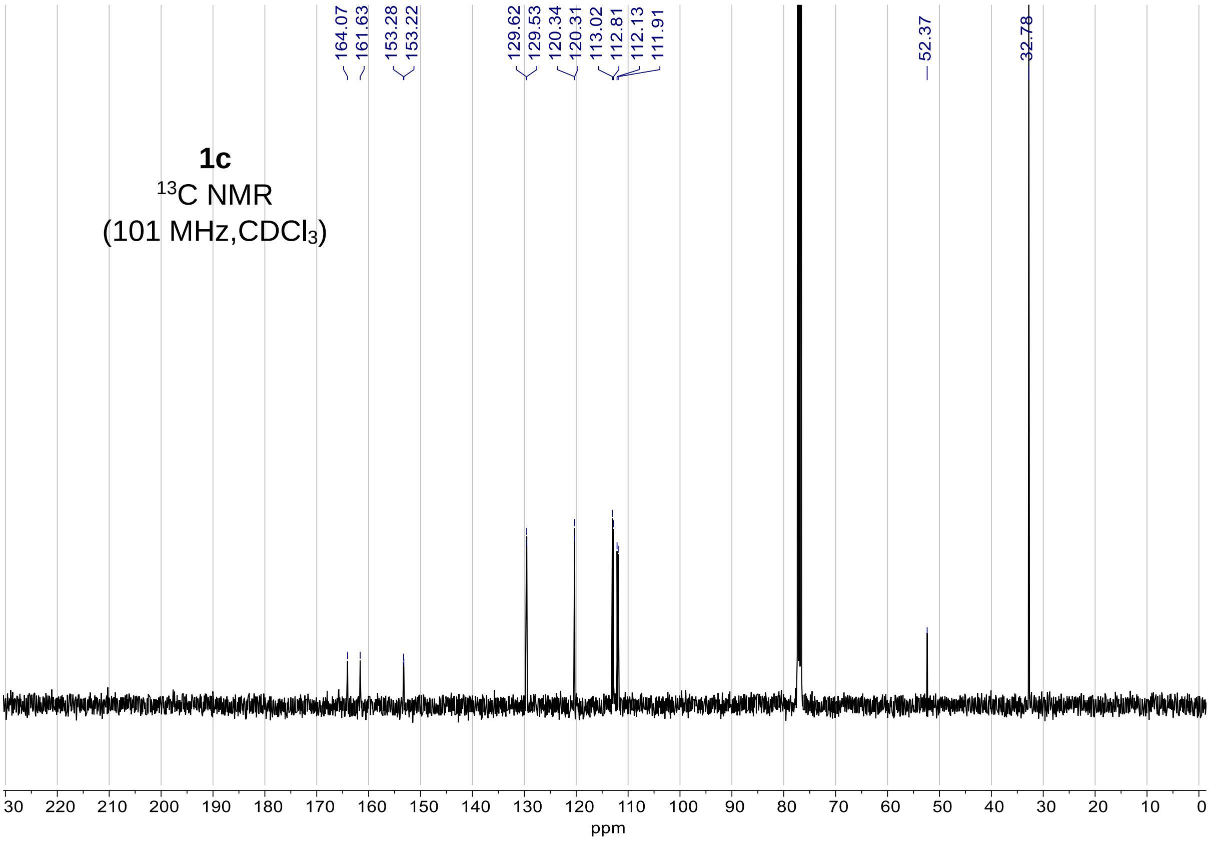
164.07 161.63 153.28 153.22
129.62 129.53 120.34 120.31 113.02 112.81 112.13 111.91
52.37
32.78
1c
13C NMR
(101 MHz,CDCl3)
30 220 210 200 190 180 170 160 150 140 130 120 110 100 90 80 70 60 50 40 30 20 10 0
ppm

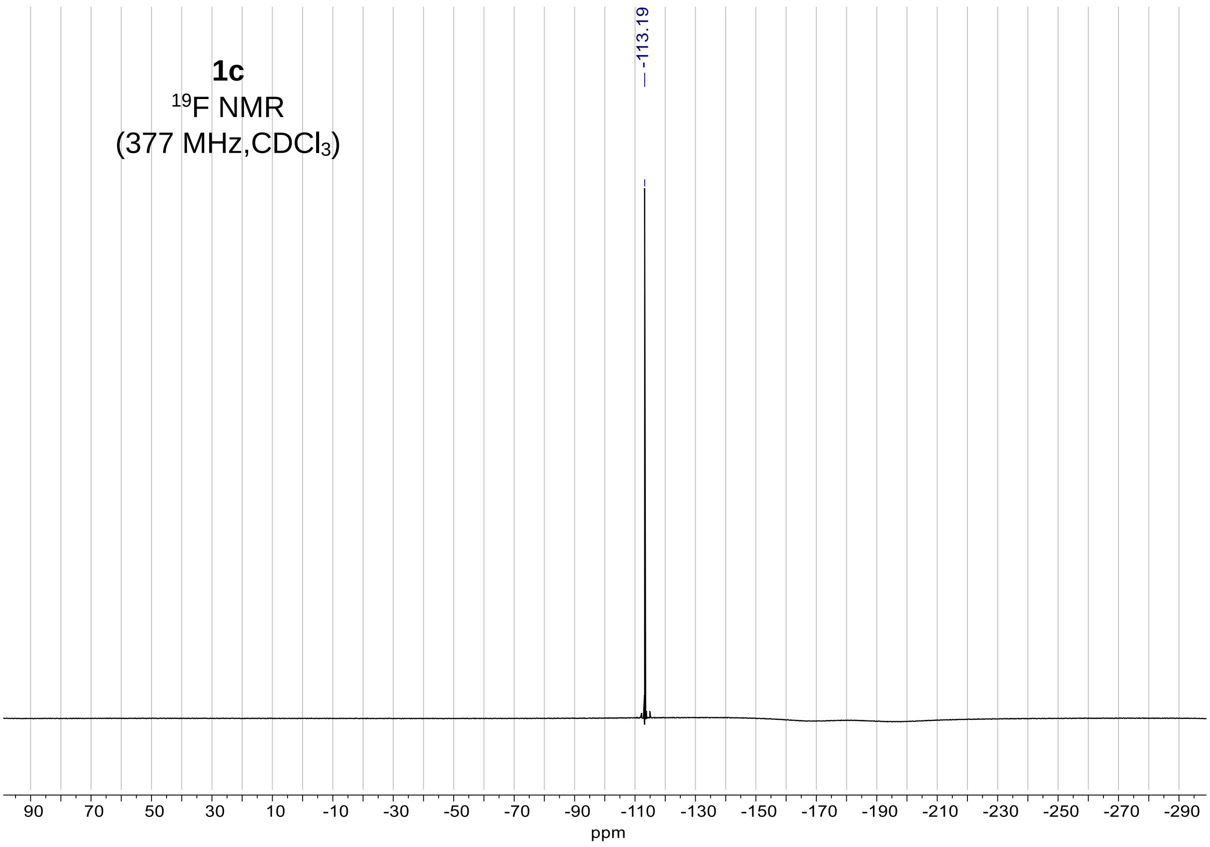
1c
19F NMR
(377 MHz,CDCl3)
-113.19
90 70 50 30 10 -10 -30 -50 -70 -90 -110 -130 -150 -170 -190 -210 -230 -250 -270 -290
ppm

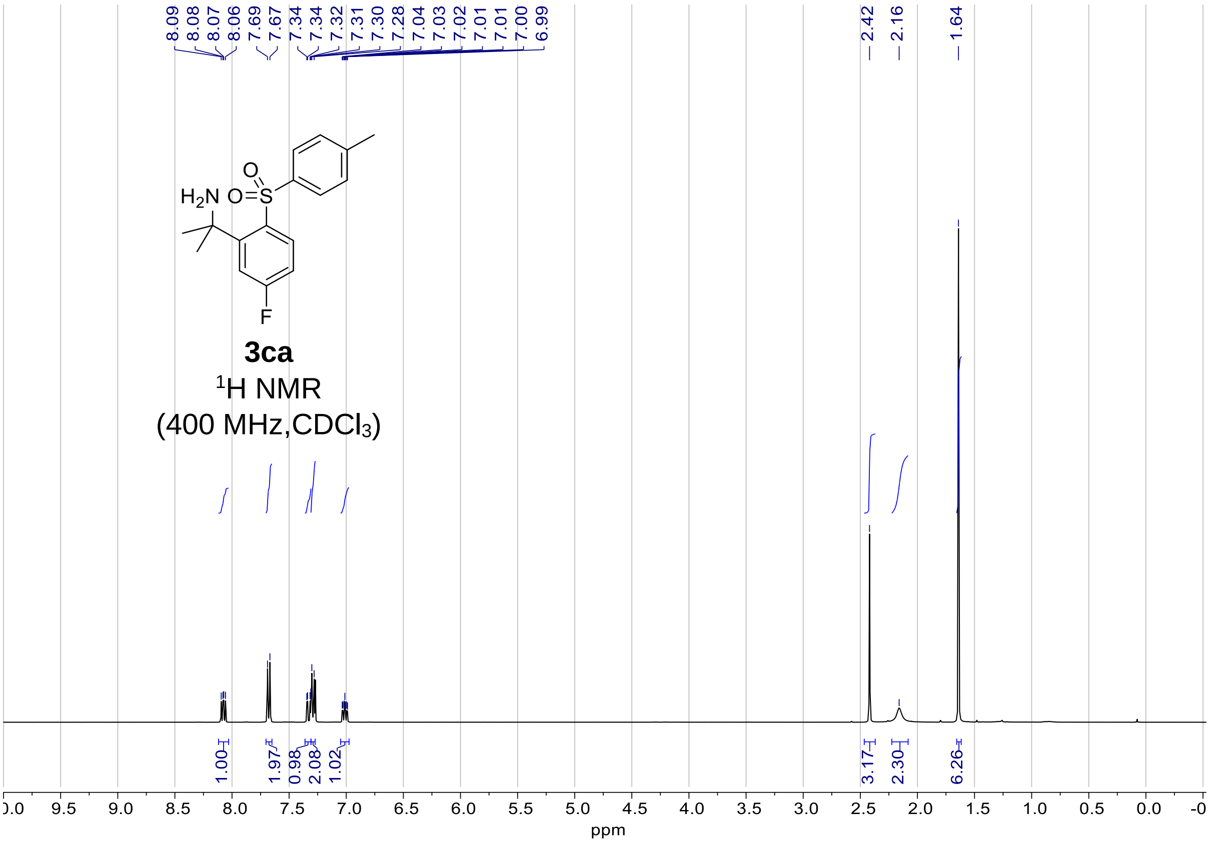
8.09
8.08
8.07
8.06
7.69
7.67
7.34
7.34
7.32
7.31
7.30
7.28
7.04
7.03
7.02
7.01
7.01
7.00
6.99
2.42
2.16
1.64
H2N
O
O=S
F
3ca
1H NMR
(400 MHz,CDCl3)
1.00
1.97
0.98
2.08
1.02
3.17
2.30
6.26
ppm

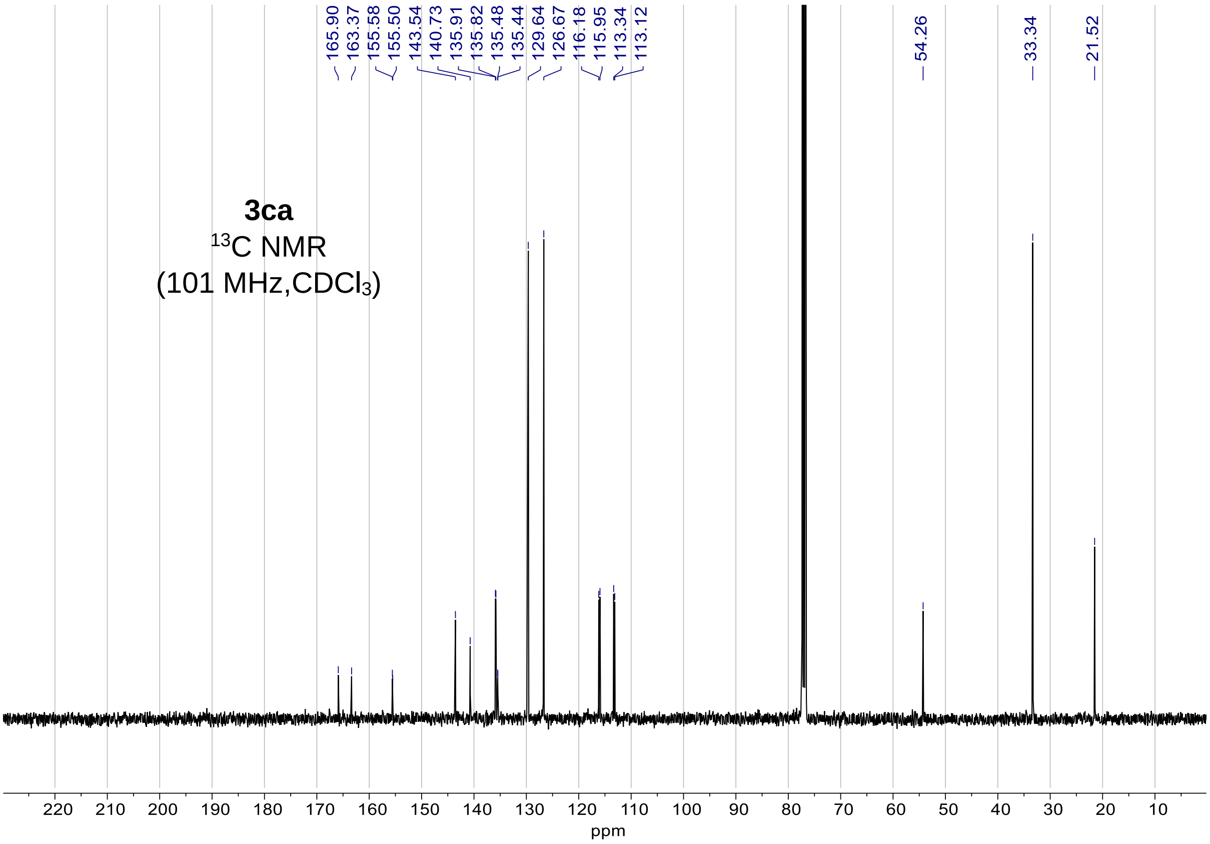
165.90
163.37
155.58
155.50
143.54
140.73
135.91
135.82
135.48
135.44
129.64
126.67
116.18
115.95
113.34
113.12
54.26
33.34
21.52
3ca
13C NMR
(101 MHz,CDCl3)
ppm

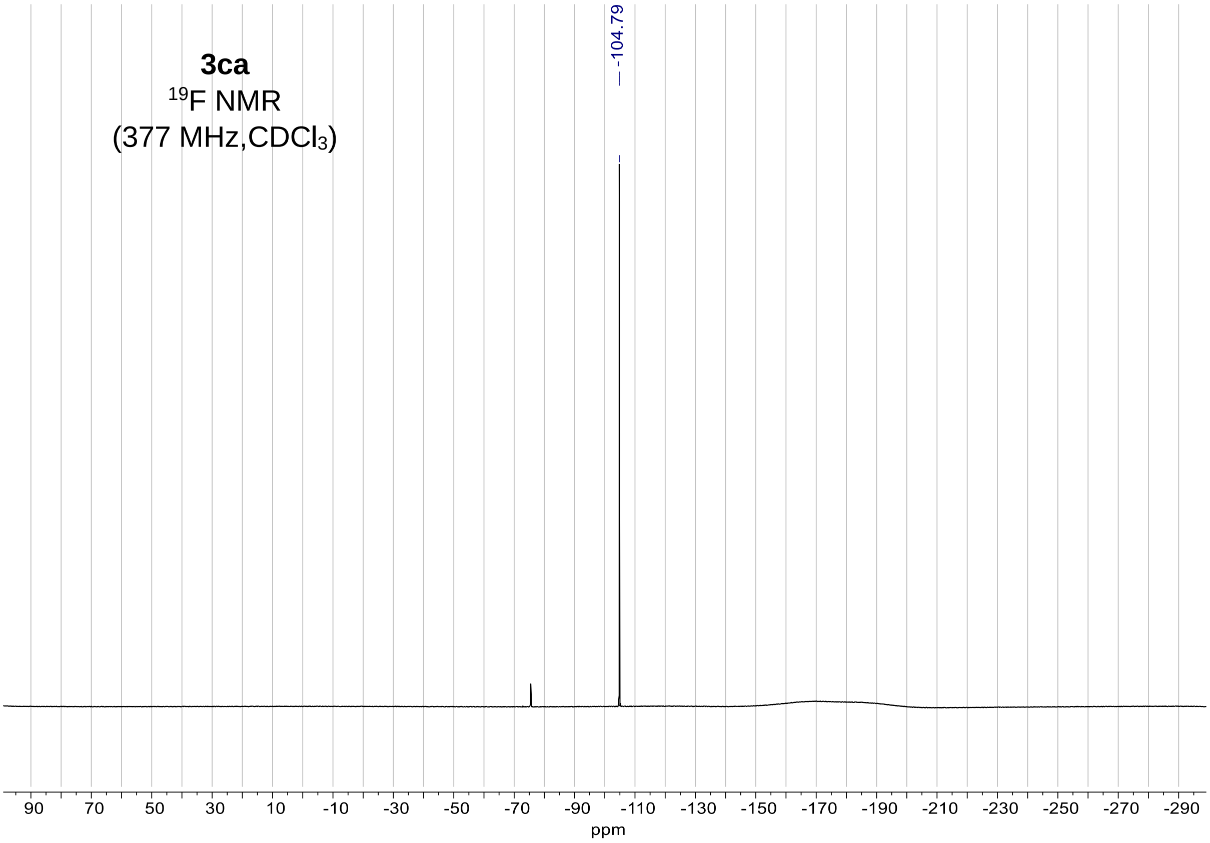
3ca
19F NMR
(377 MHz,CDCl3)
-104.79
90
70
50
30
10
-10
-30
-50
-70
-90
-110
-130
-150
-170
-190
-210
-230
-250
-270
-290
ppm

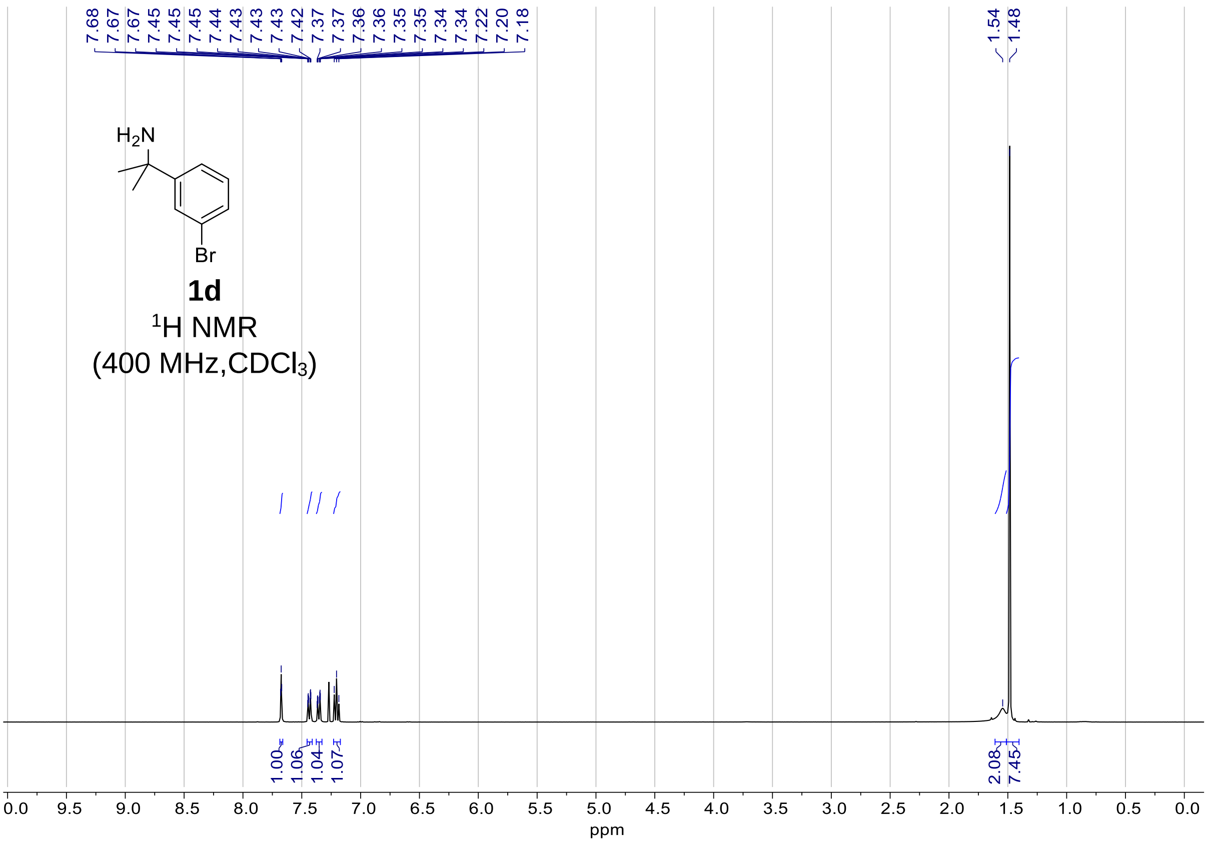
7.68
7.67
7.67
7.45
7.45
7.44
7.43
7.43
7.43
7.42
7.37
7.37
7.36
7.36
7.35
7.35
7.34
7.34
7.22
7.20
7.18
1.54
1.48
H2N
Br
1d
1H NMR
(400 MHz,CDCl3)
1.00
1.06
1.04
1.07
2.08
7.45
ppm

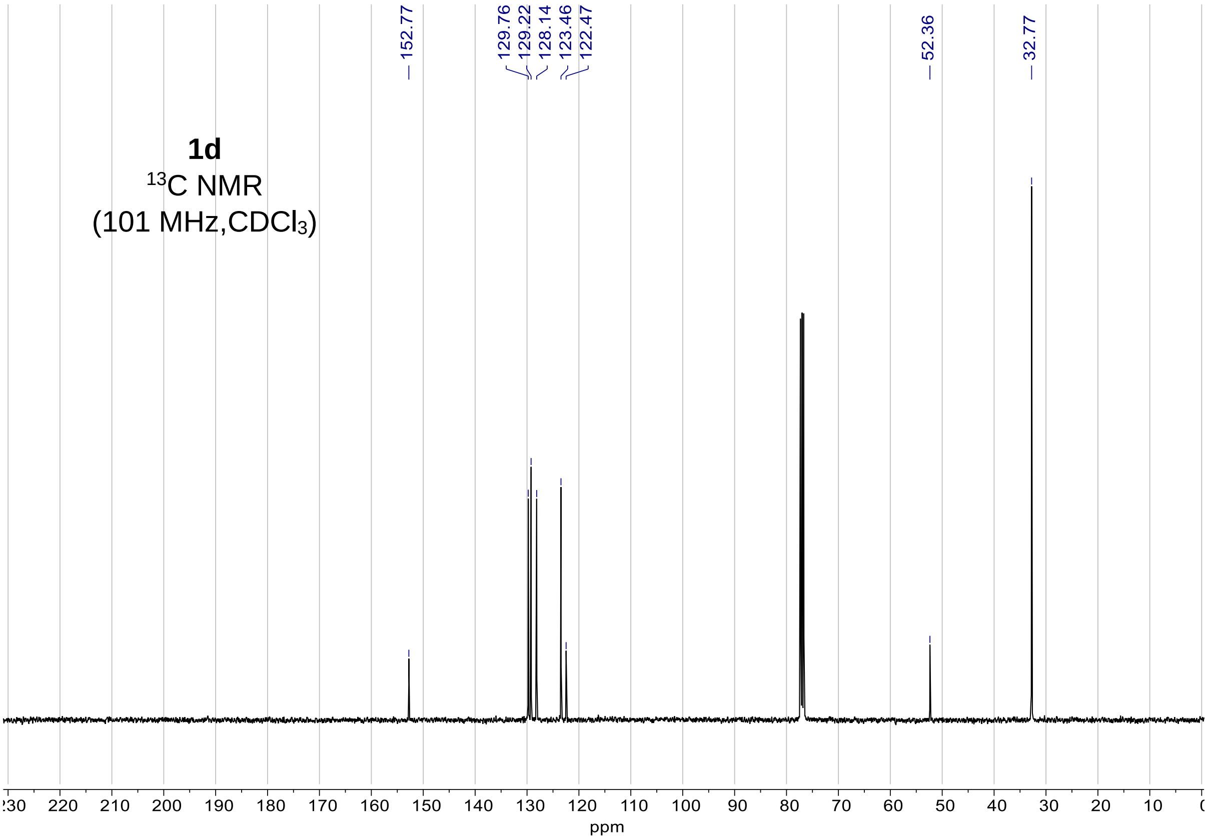
152.77
129.76
129.22
128.14
123.46
122.47
52.36
32.77
1d
13C NMR
(101 MHz,CDCl3)
ppm

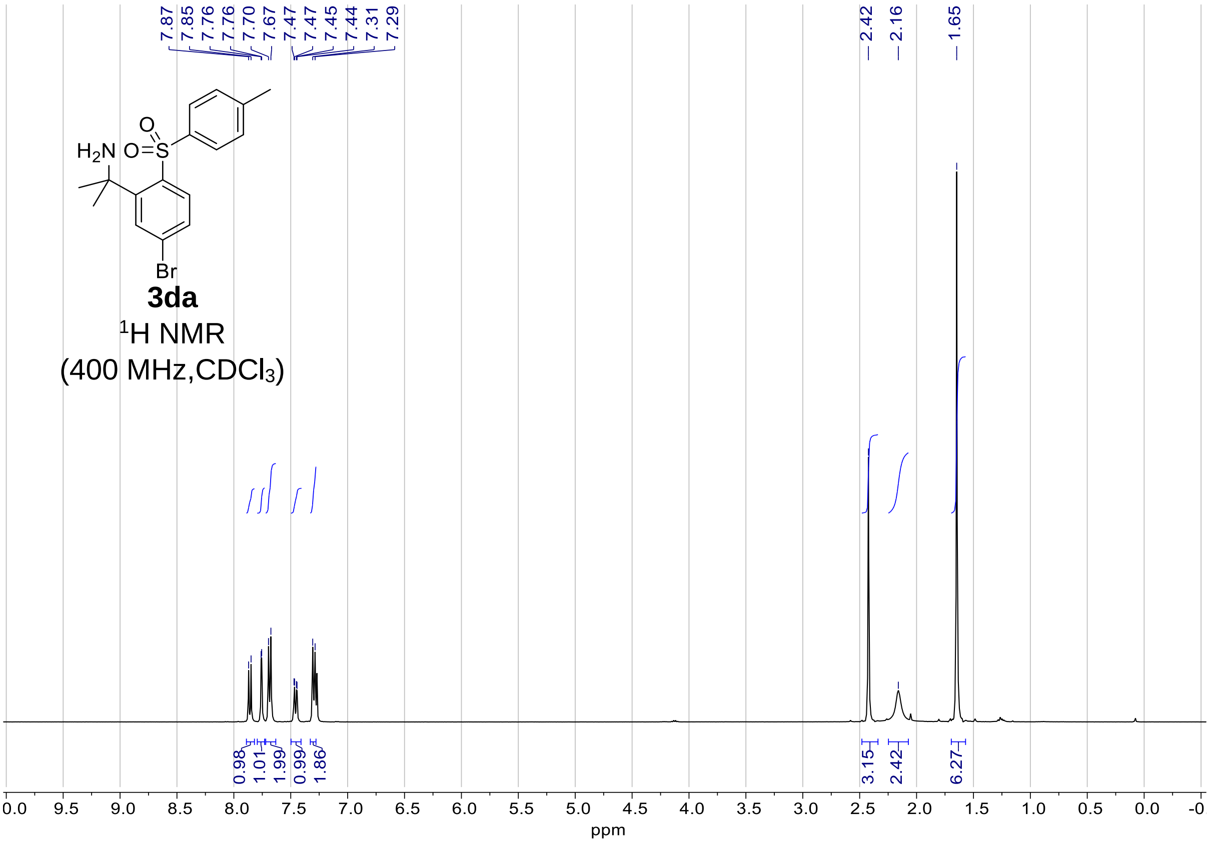
7.87
7.85
7.76
7.76
7.70
7.67
7.47
7.47
7.45
7.44
7.31
7.29
2.42
2.16
1.65
H2N
O
O=S
Br
3da
1H NMR
(400 MHz,CDCl3)
0.98
1.01
1.99
0.99
1.86
3.15
2.42
6.27
ppm

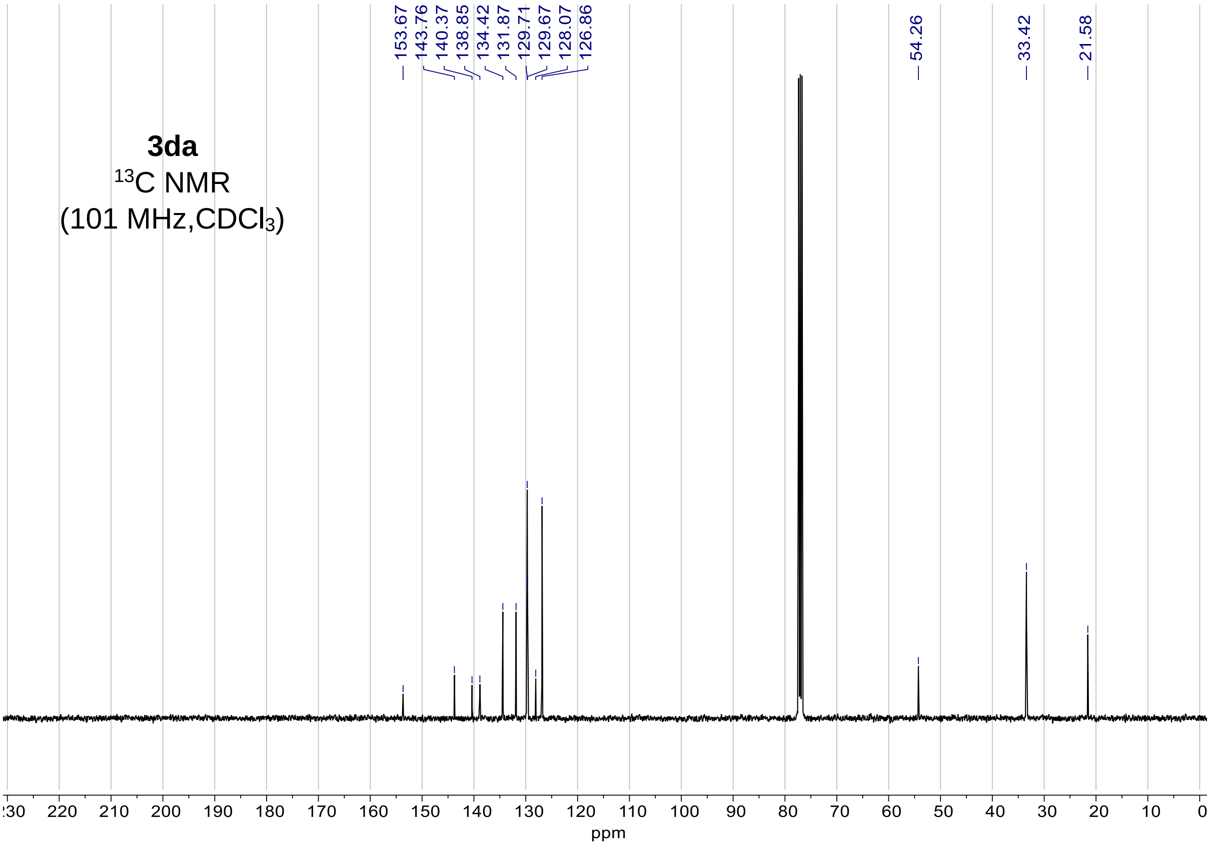
153.67
143.76
140.37
138.85
134.42
131.87
129.71
129.67
128.07
126.86
54.26
33.42
21.58
3da
13C NMR
(101 MHz,CDCl3)
ppm

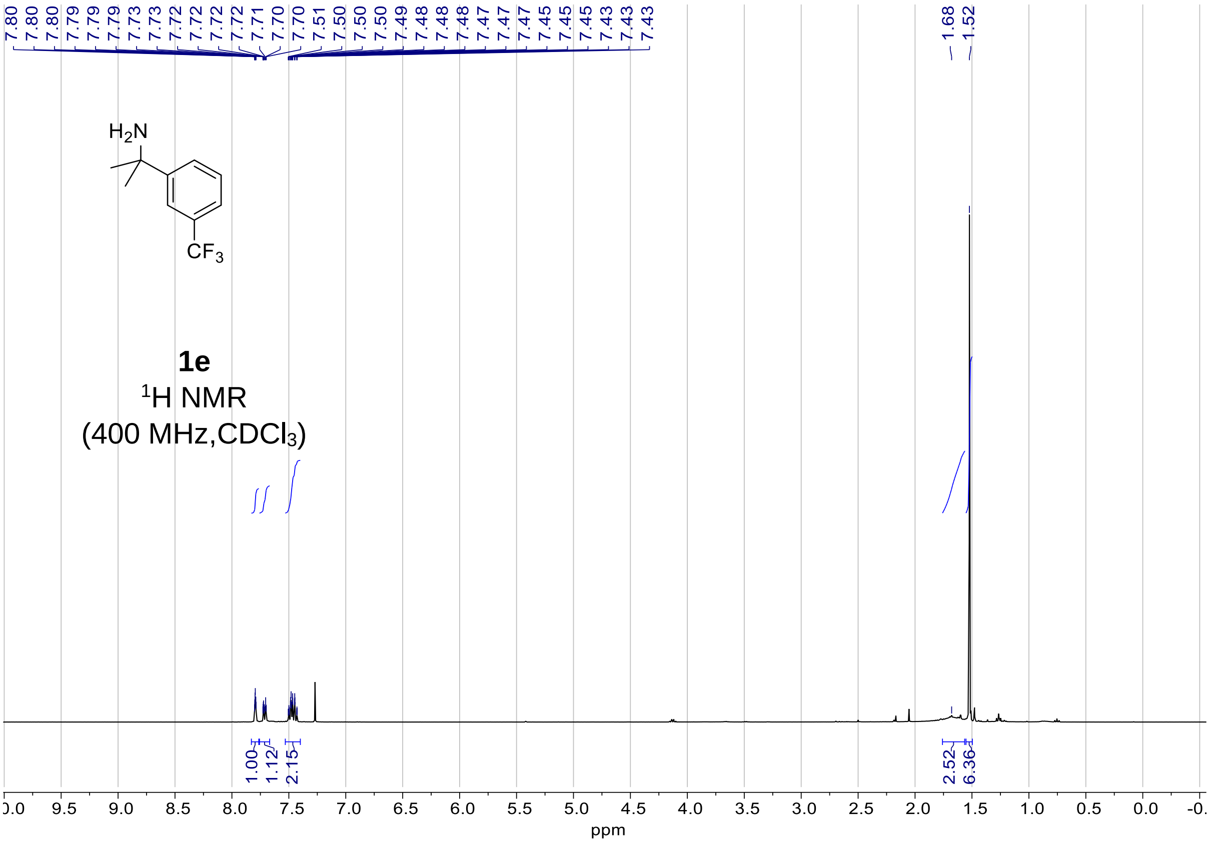
7.80 7.80 7.80 7.79 7.79 7.79 7.73 7.73 7.72 7.72 7.72 7.72 7.71 7.70 7.70 7.51 7.50 7.50 7.49 7.48 7.48 7.48 7.47 7.47 7.47 7.45 7.45 7.45 7.43 7.43 7.43
1.68
1.52
H2N
CF3
1e
1H NMR
(400 MHz,CDCl3)
1.00
1.12
2.15
2.52
6.36
ppm

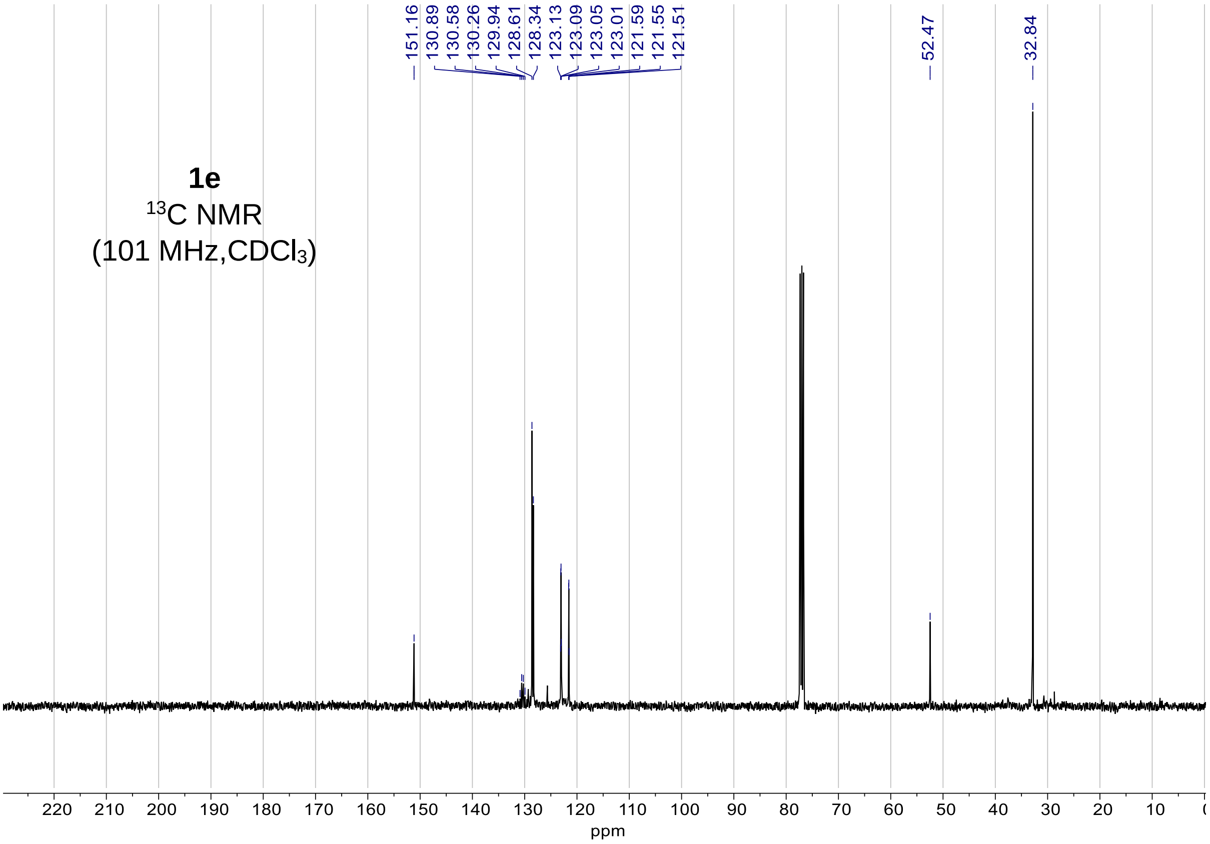
151.16
130.89 130.58 130.26 129.94 128.61 128.34 123.13 123.09 123.05 123.01 121.59 121.55 121.51
52.47
32.84
1e
13C NMR
(101 MHz,CDCl3)
ppm

1e
^{19}F NMR
(377 MHz, $CDCl_3$)

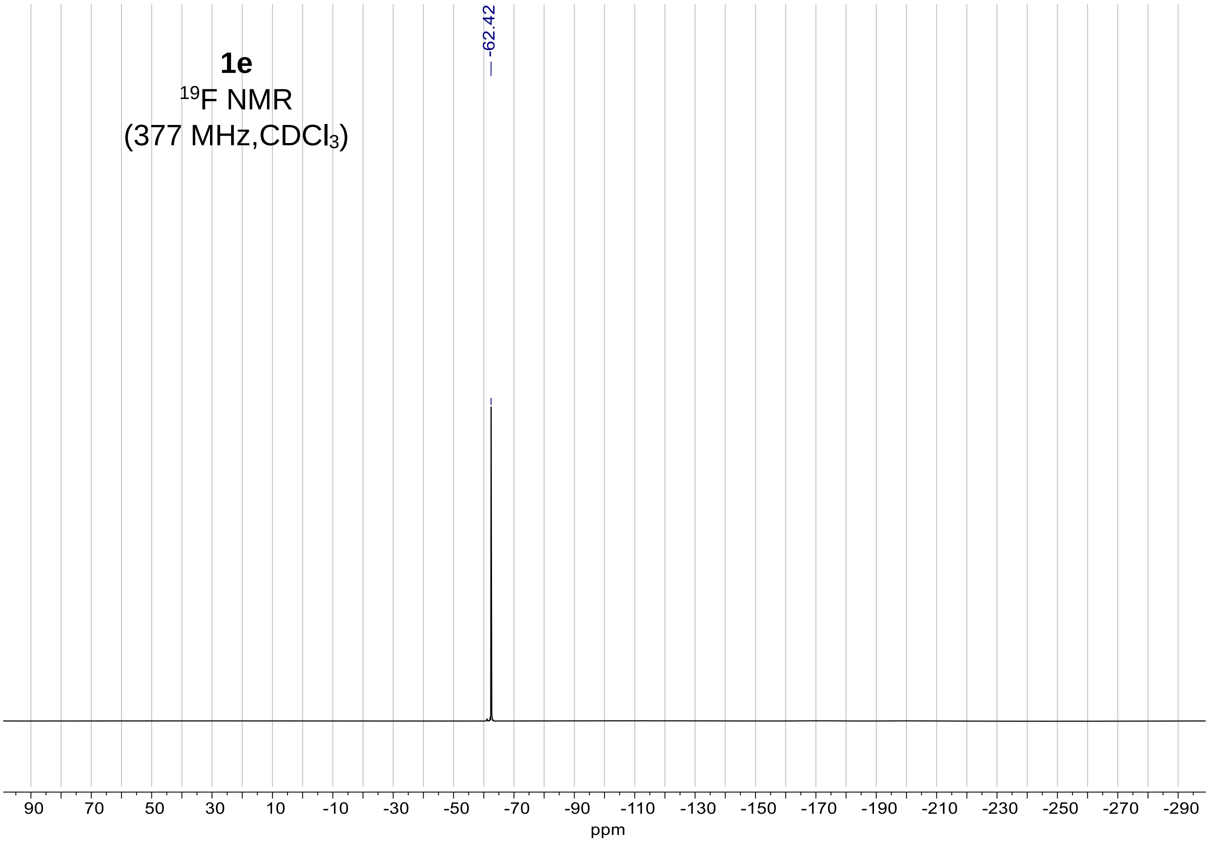

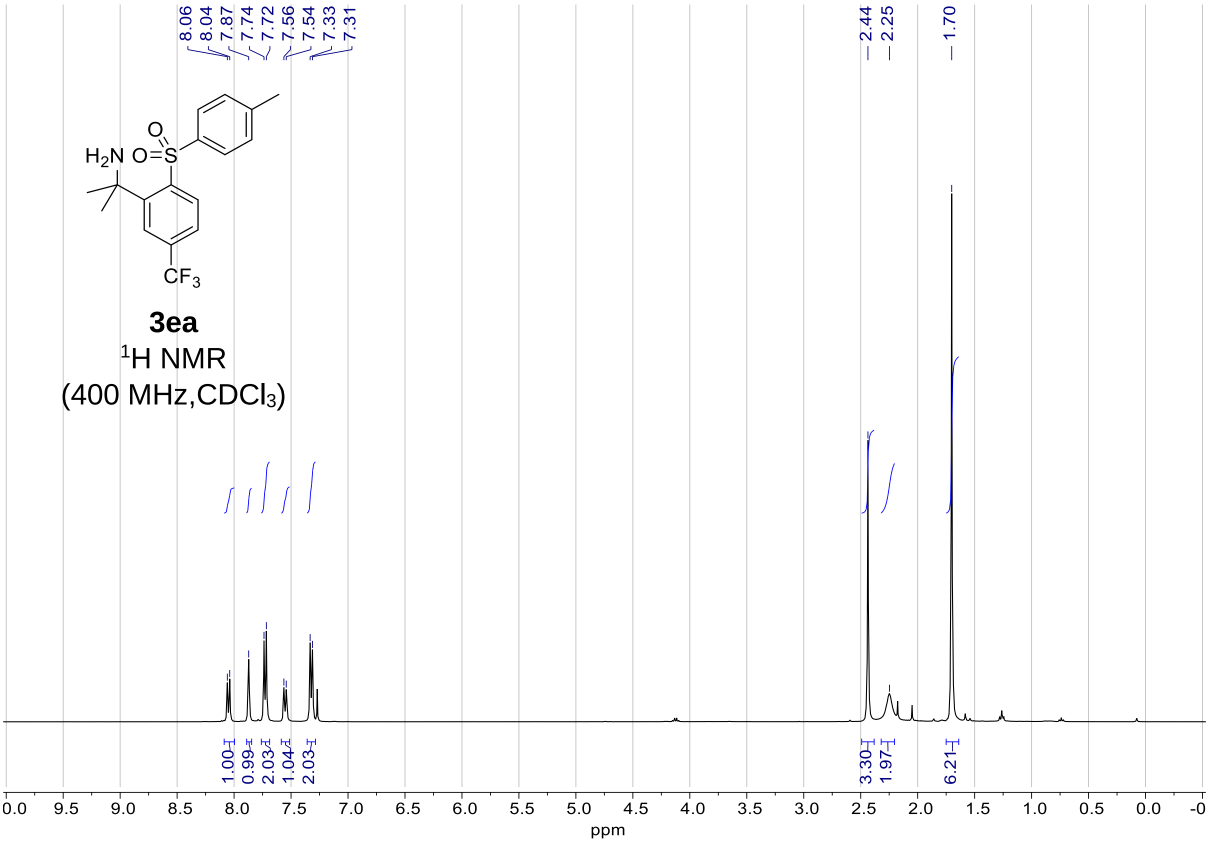
8.06 8.04 7.87 7.74 7.72 7.56 7.54 7.33 7.31
2.44 2.25
1.70
H2N O=S O
CF3
3ea
1H NMR
(400 MHz,CDCl3)
1.00 0.99 2.03 1.04 2.03
3.30 1.97
6.21
0.0 9.5 9.0 8.5 8.0 7.5 7.0 6.5 6.0 5.5 5.0 4.5 4.0 3.5 3.0 2.5 2.0 1.5 1.0 0.5 0.0
ppm

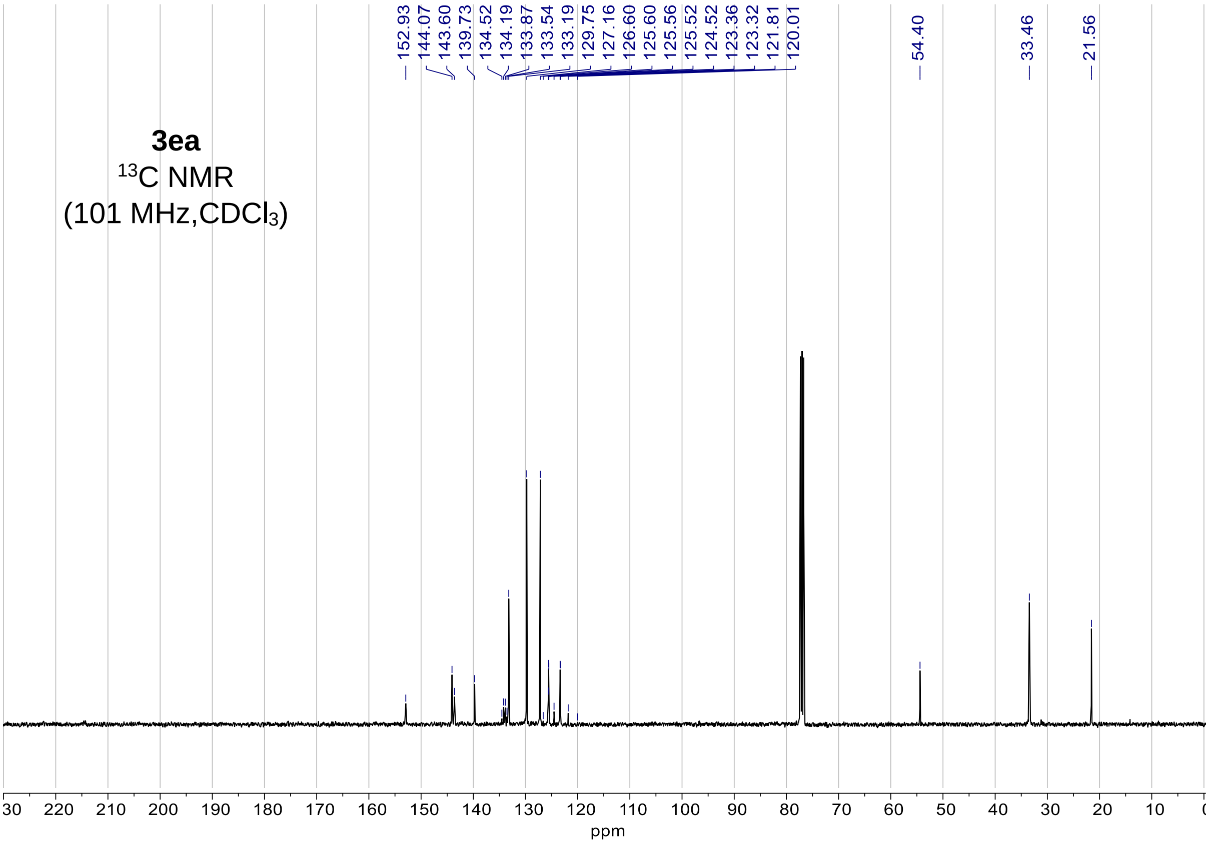
152.93 144.07 143.60 139.73 134.52 134.19 133.87 133.54 133.19 129.75 127.16 126.60 125.60 125.56 125.52 124.52 123.36 123.32 121.81 120.01
54.40
33.46
21.56
3ea
13C NMR
(101 MHz,CDCl3)
230 220 210 200 190 180 170 160 150 140 130 120 110 100 90 80 70 60 50 40 30 20 10 0
ppm

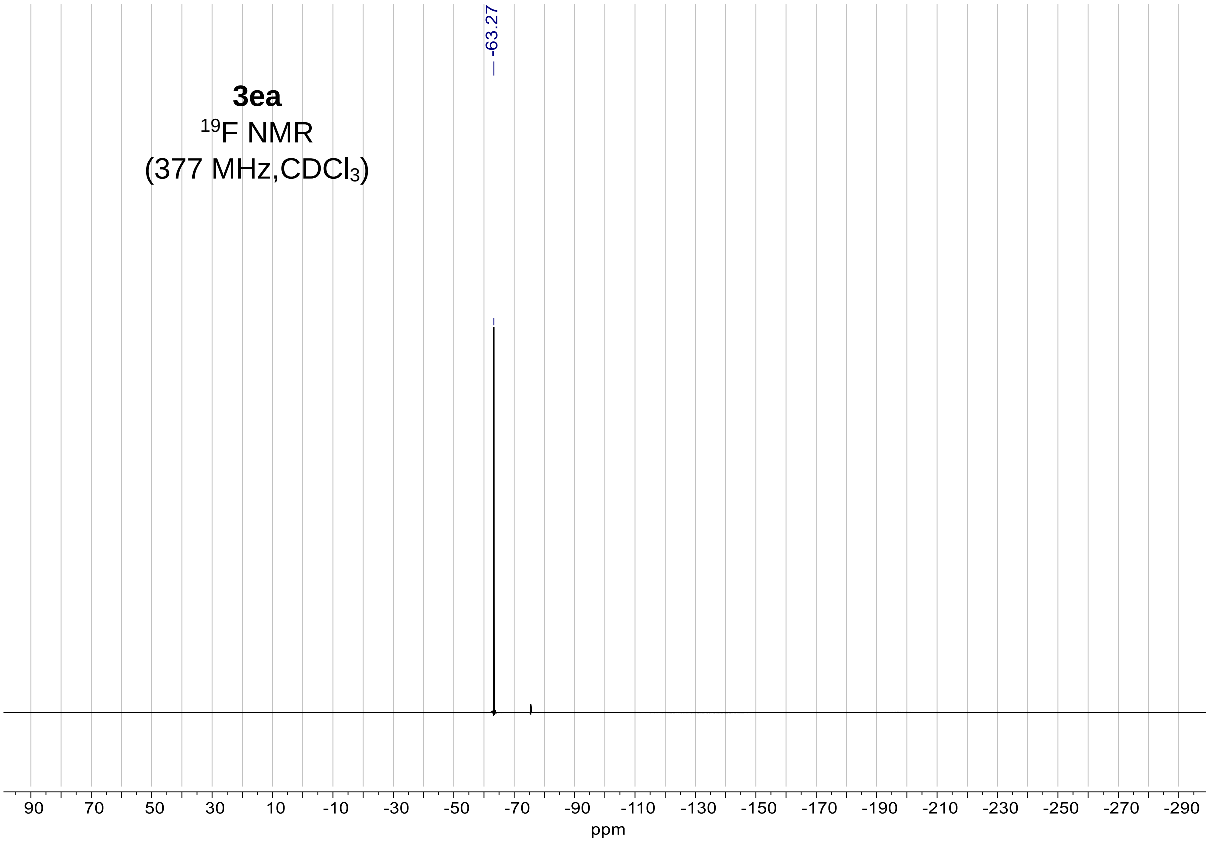
-63.27
3ea
19F NMR
(377 MHz,CDCl3)
90 70 50 30 10 -10 -30 -50 -70 -90 -110 -130 -150 -170 -190 -210 -230 -250 -270 -290
ppm

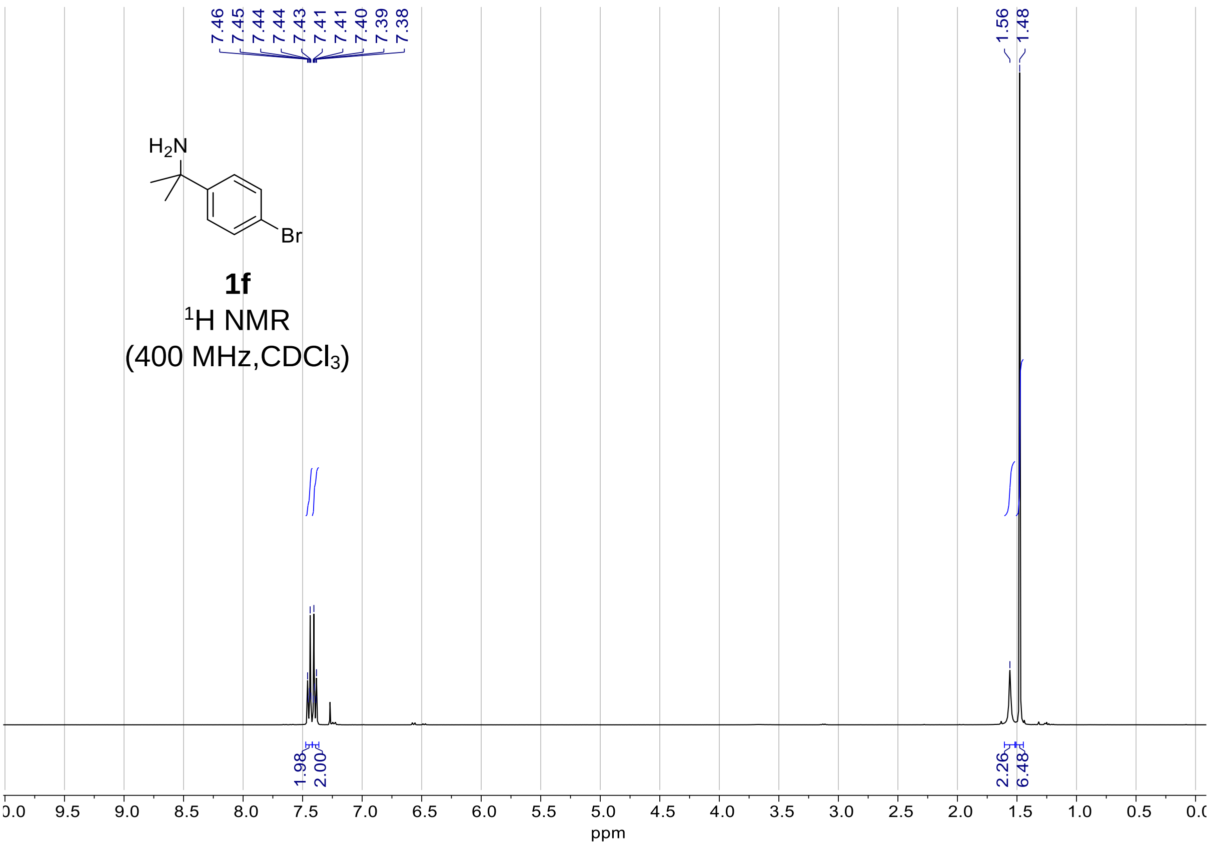
7.46
7.45
7.44
7.44
7.43
7.41
7.41
7.40
7.39
7.38
1.56
1.48
H2N
Br
1f
1H NMR
(400 MHz,CDCl3)
1.98
2.00
2.26
6.48
ppm

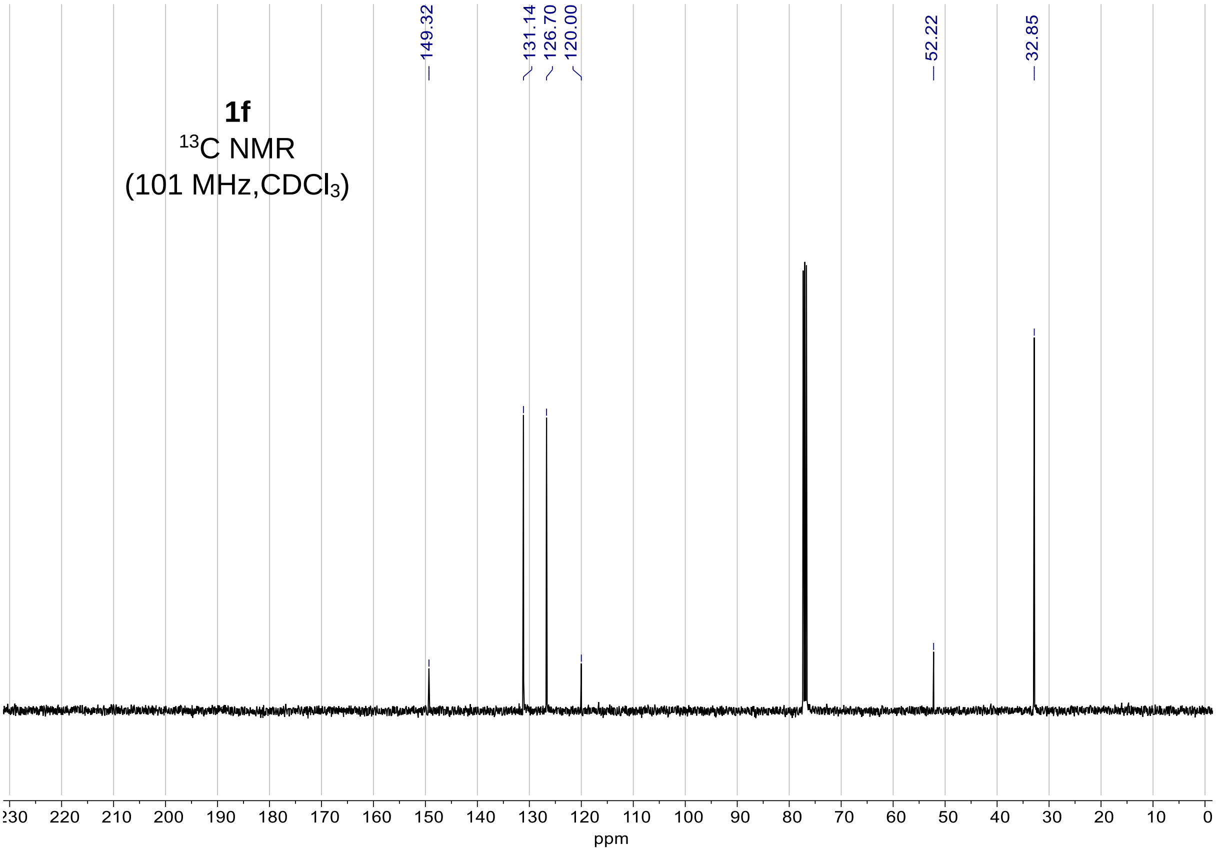
149.32
131.14
126.70
120.00
52.22
32.85
1f
13C NMR
(101 MHz,CDCl3)
ppm

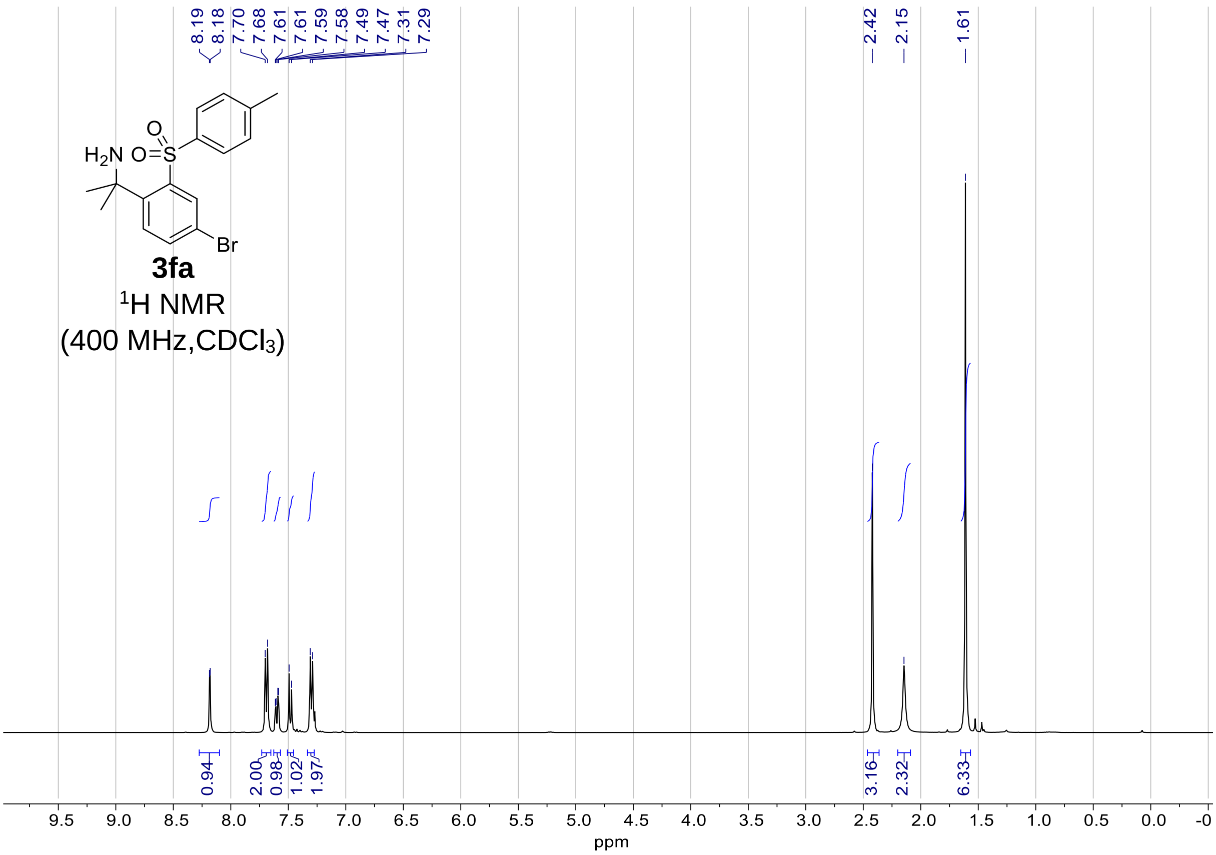
8.19
8.18
7.70
7.68
7.61
7.61
7.59
7.58
7.49
7.47
7.31
7.29
2.42
2.15
1.61
H2N
O=S
O
Br
3fa
1H NMR
(400 MHz,CDCl3)
0.94
2.00
0.98
1.02
1.97
3.16
2.32
6.33
ppm

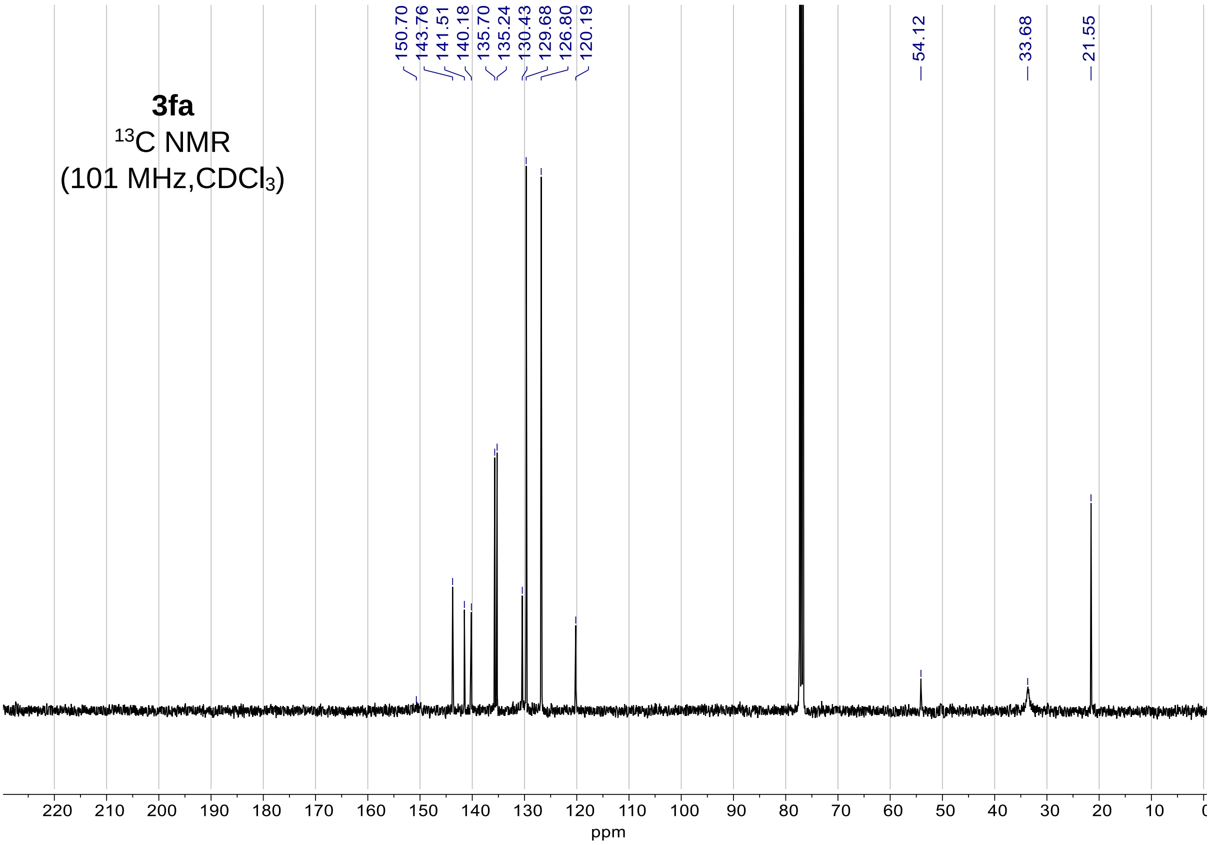
150.70
143.76
141.51
140.18
135.70
135.24
130.43
129.68
126.80
120.19
54.12
33.68
21.55
3fa
13C NMR
(101 MHz,CDCl3)
ppm

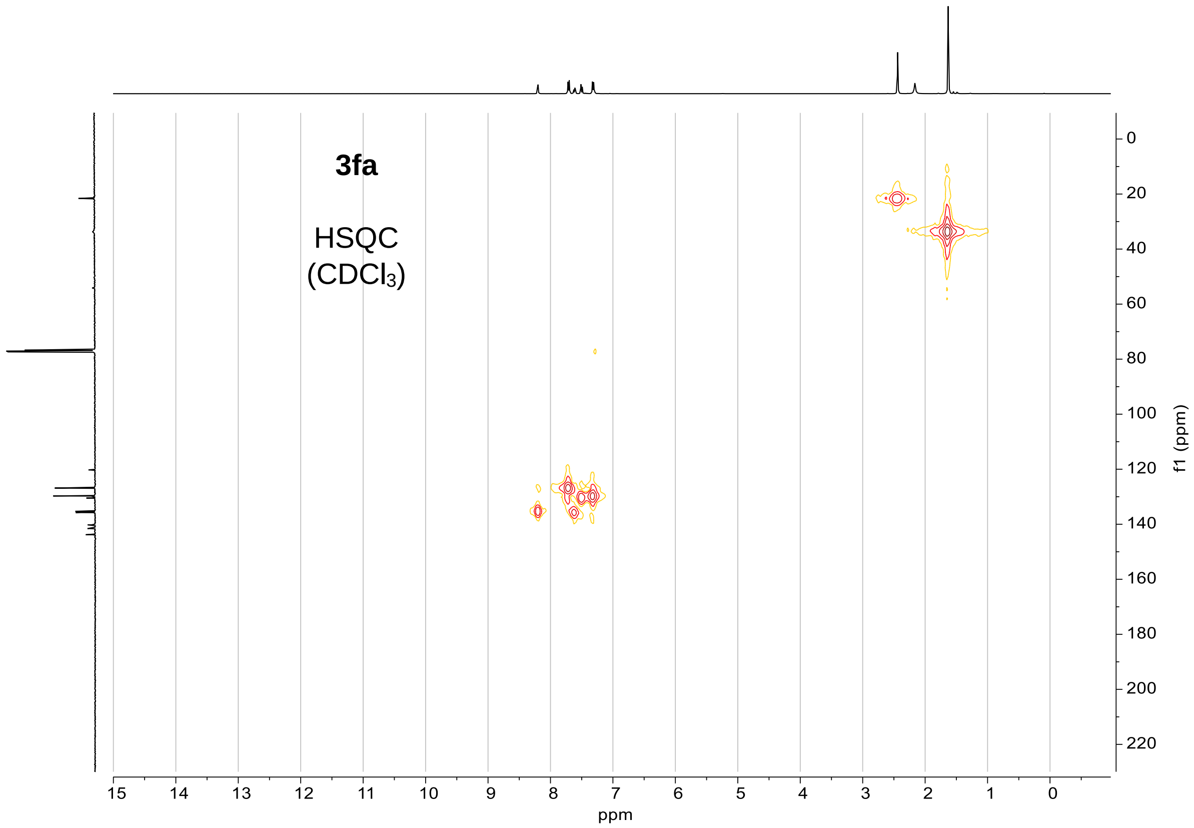
3fa
HSQC
(CDCl3)
ppm
f1 (ppm)

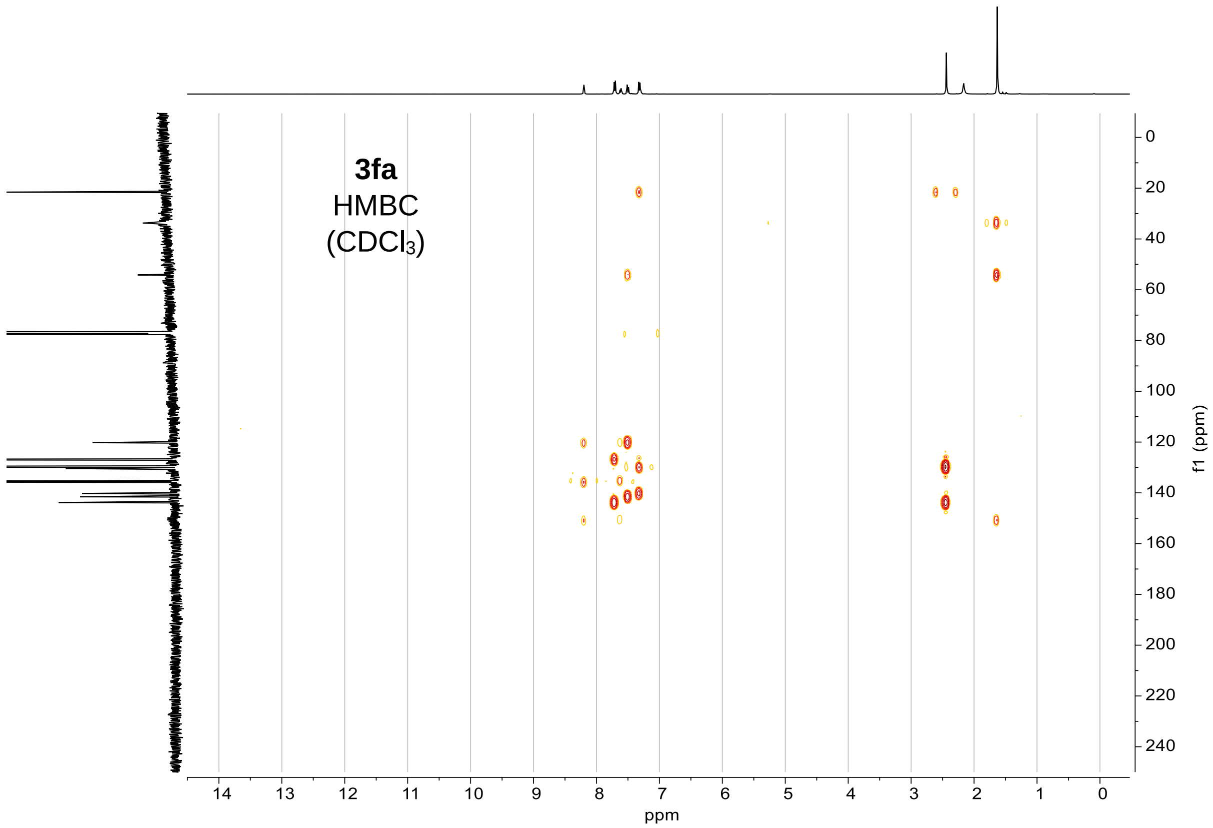
3fa
HMBC
(CDCl3)
ppm
f1 (ppm)

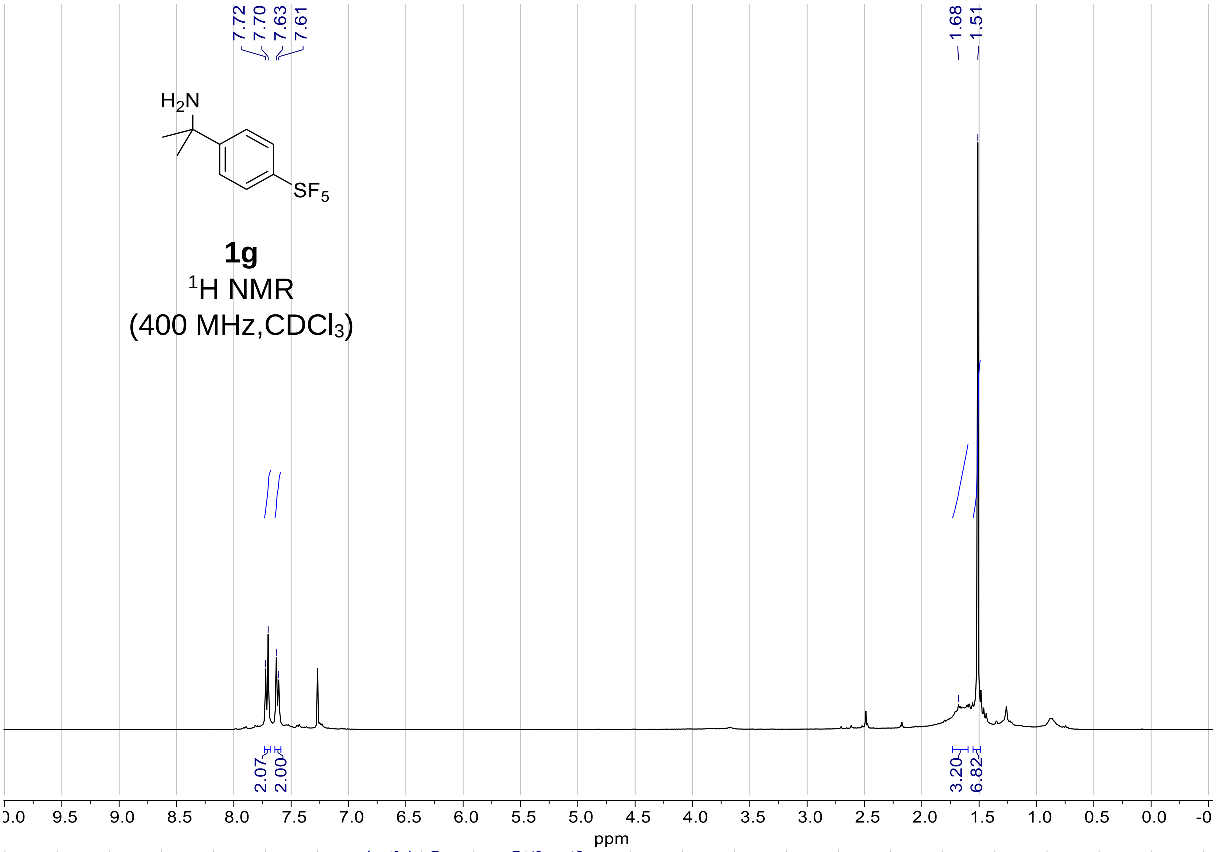
7.72
7.70
7.63
7.61
1.68
1.51
H_2N
SF_5
1g
^1H NMR
(400 MHz,$CDCl_3$)
2.07
2.00
3.20
6.82
ppm

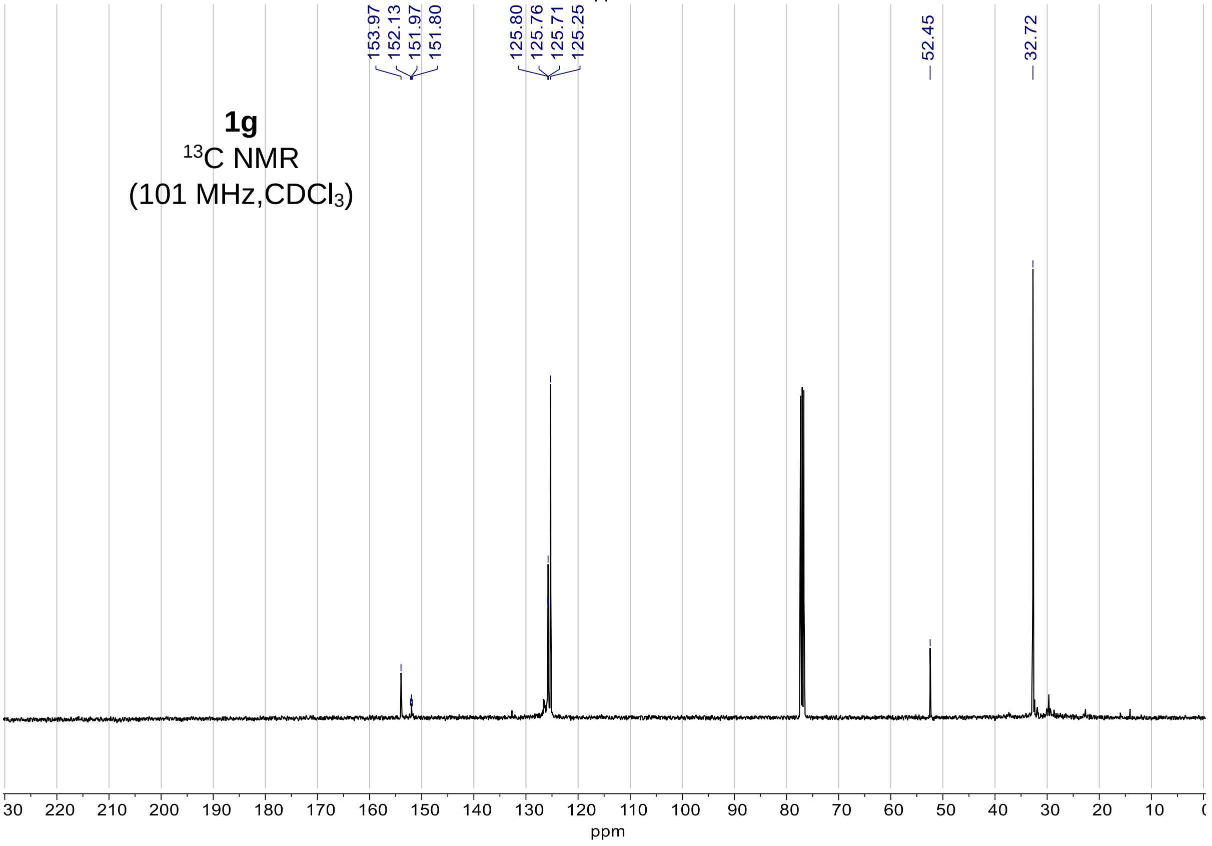
153.97
152.13
151.97
151.80
125.80
125.76
125.71
125.25
52.45
32.72
1g
^{13}C NMR
(101 MHz,$CDCl_3$)
ppm

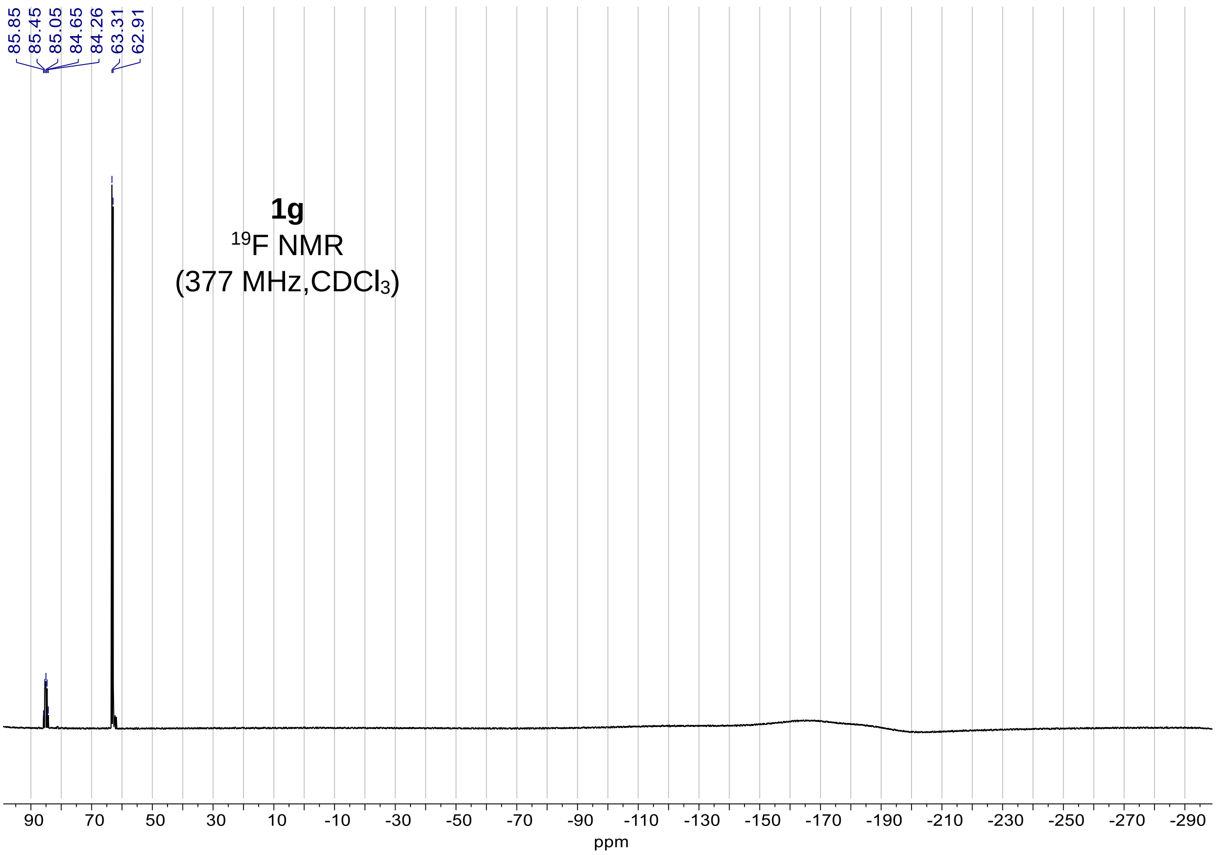
85.85
85.45
85.05
84.65
84.26
63.31
62.91
1g
^{19}F NMR
(377 MHz,$CDCl_3$)
90 70 50 30 10 -10 -30 -50 -70 -90 -110 -130 -150 -170 -190 -210 -230 -250 -270 -290
ppm

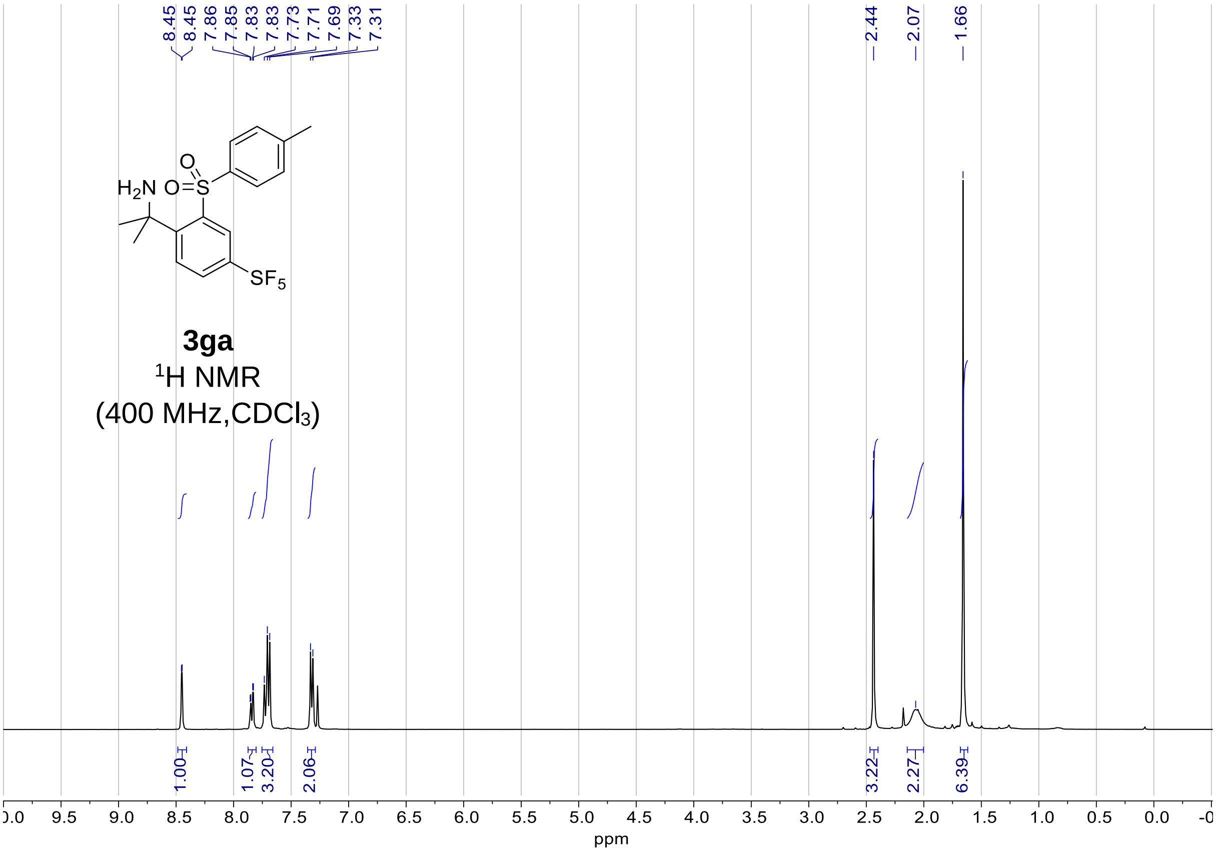
8.45
8.45
7.86
7.85
7.83
7.83
7.73
7.71
7.69
7.33
7.31
2.44
2.07
1.66
H2N
O
O=S
SF5
3ga
1H NMR
(400 MHz,CDCl3)
1.00
1.07
3.20
2.06
3.22
2.27
6.39
ppm

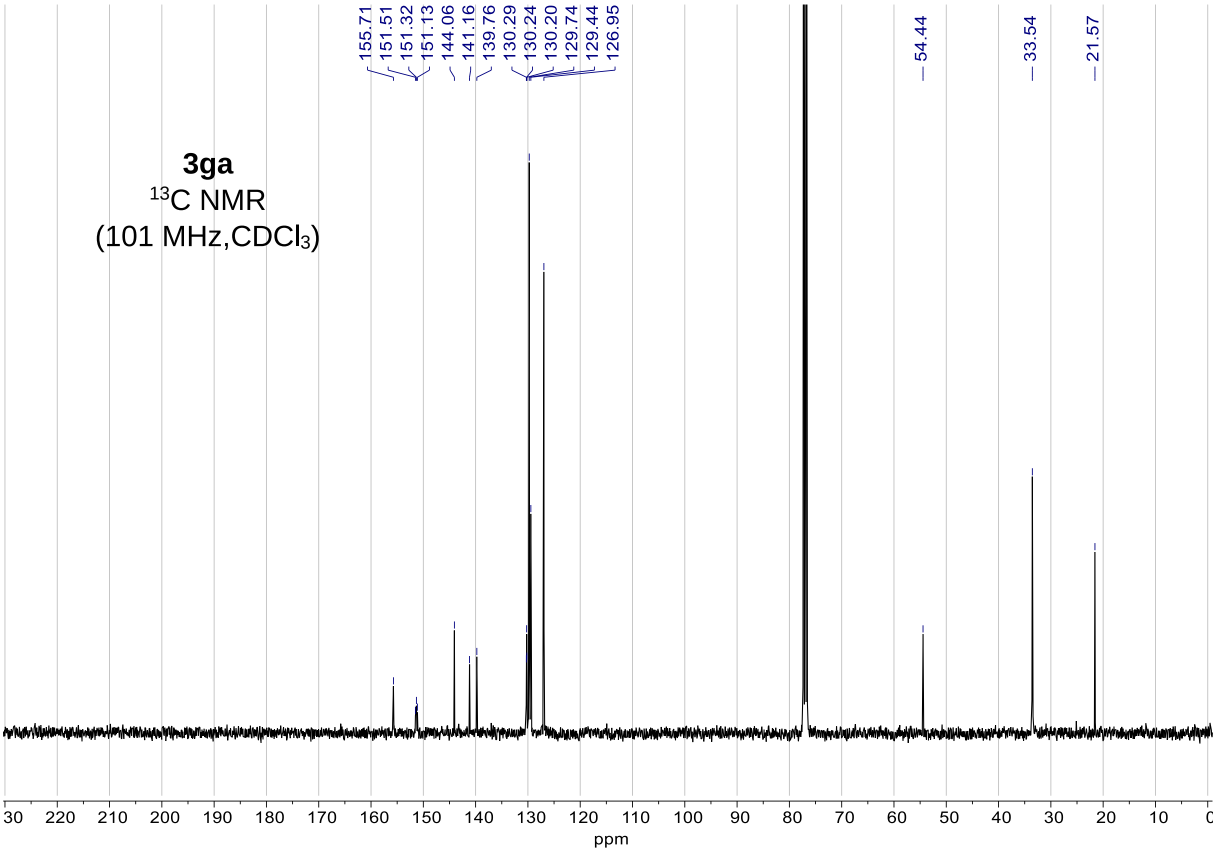
155.71
151.51
151.32
151.13
144.06
141.16
139.76
130.29
130.24
130.20
129.74
129.44
126.95
54.44
33.54
21.57
3ga
13C NMR
(101 MHz,CDCl3)
ppm

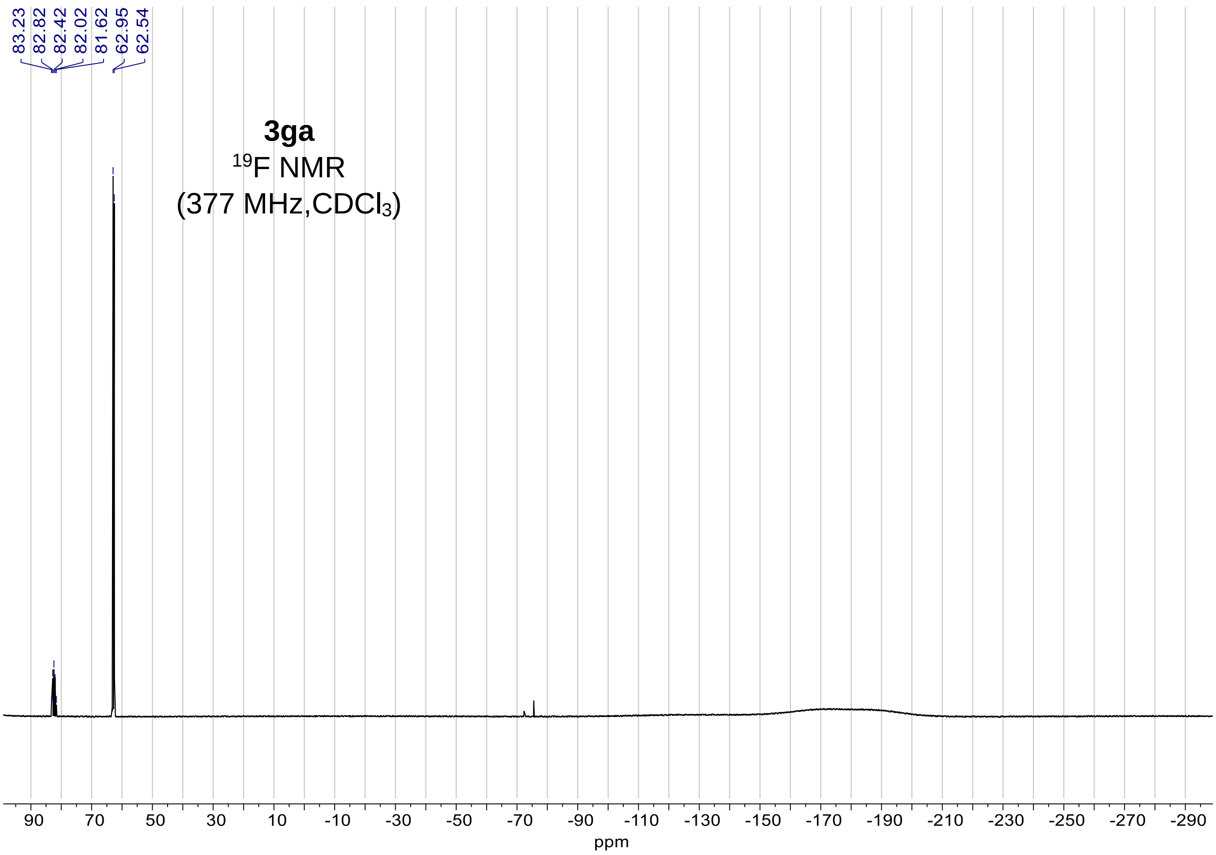
83.23
82.82
82.42
82.02
81.62
62.95
62.54
3ga
19F NMR
(377 MHz,CDCl3)
90 70 50 30 10 -10 -30 -50 -70 -90 -110 -130 -150 -170 -190 -210 -230 -250 -270 -290
ppm

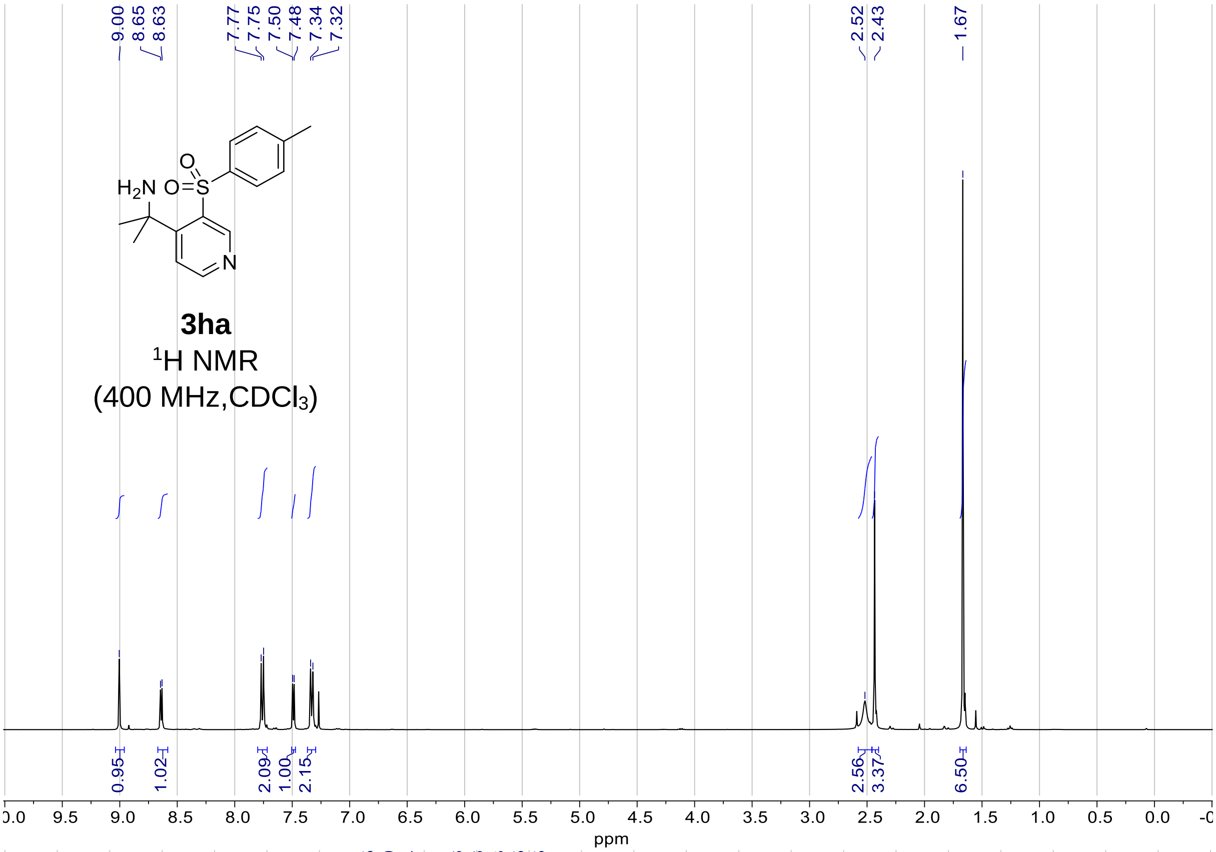
9.00
8.65
8.63
7.77
7.75
7.50
7.48
7.34
7.32
2.52
2.43
1.67
H2N
O
O=S
N
3ha
1H NMR
(400 MHz,CDCl3)
0.95
1.02
2.09
1.00
2.15
2.56
3.37
6.50
ppm

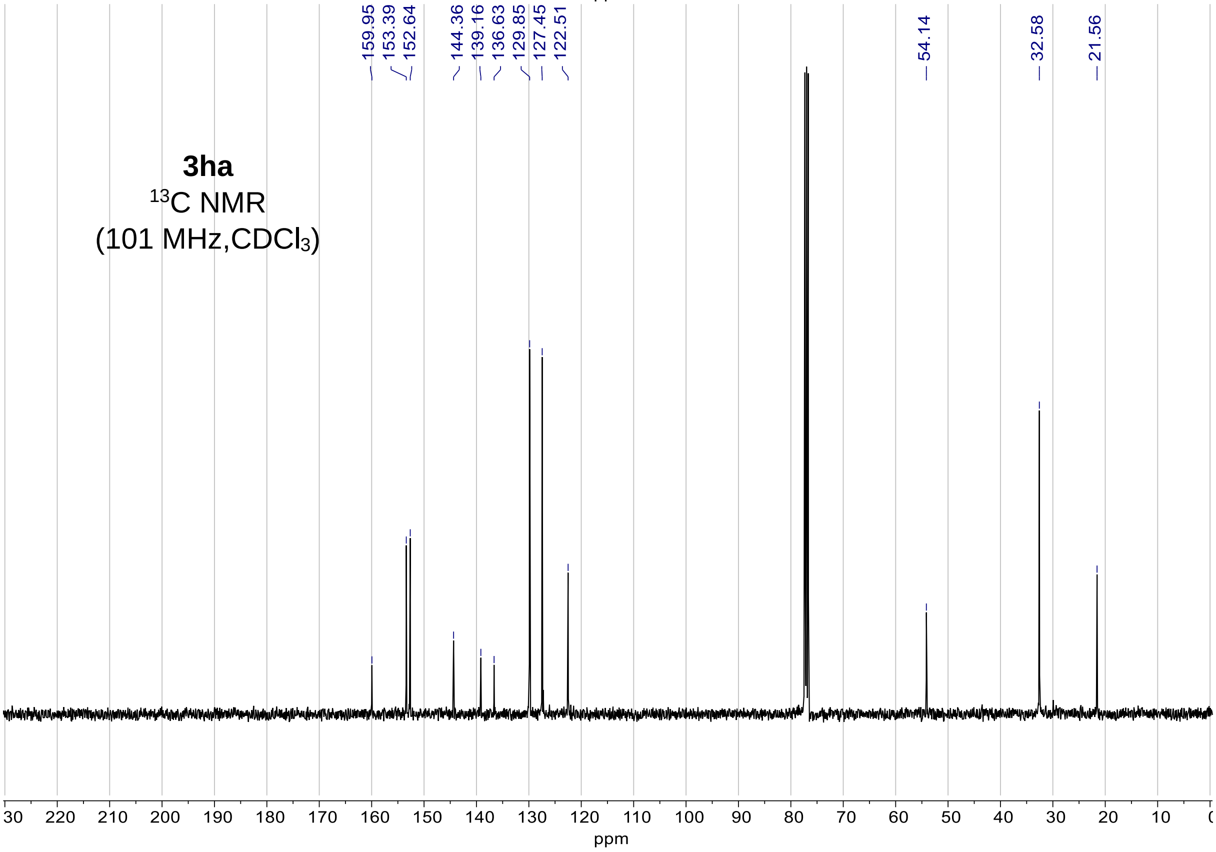
159.95
153.39
152.64
144.36
139.16
136.63
129.85
127.45
122.51
54.14
32.58
21.56
3ha
13C NMR
(101 MHz,CDCl3)
ppm

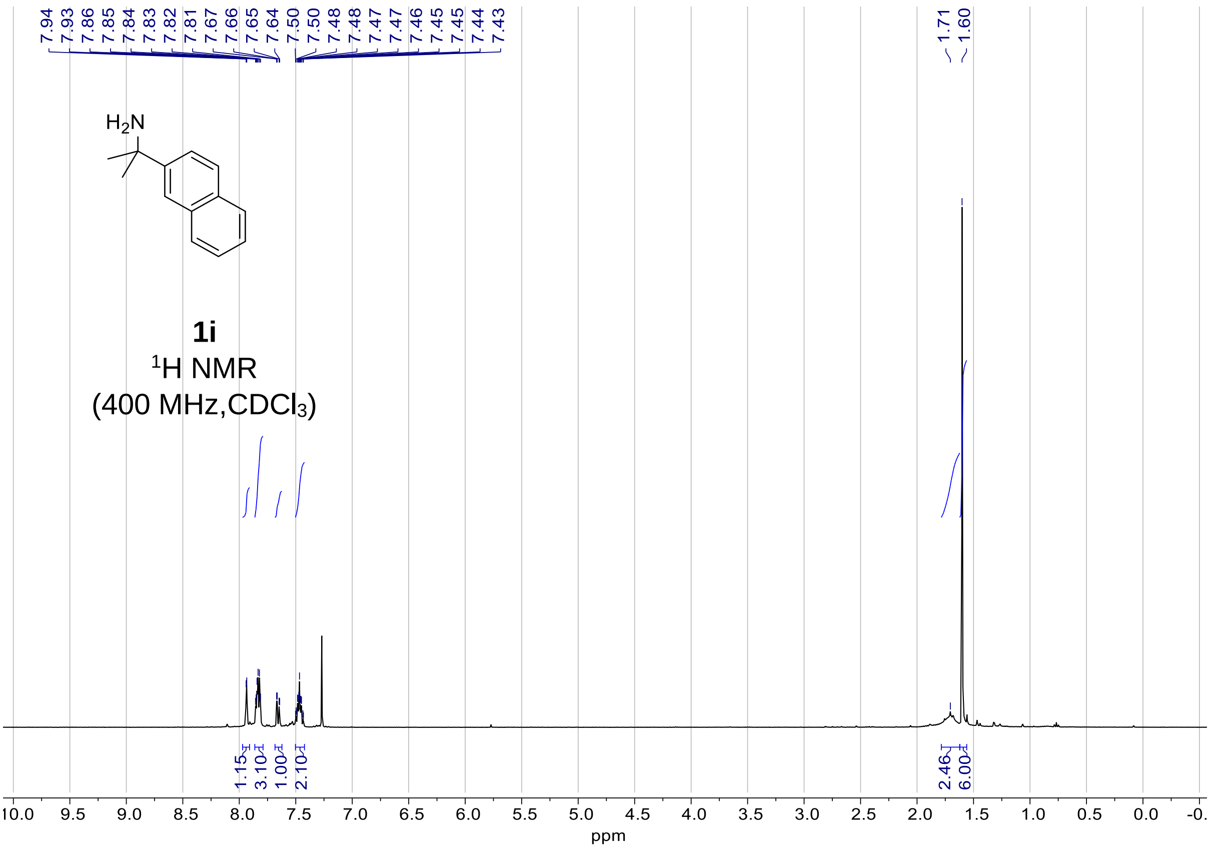
7.94
7.93
7.86
7.85
7.84
7.83
7.82
7.81
7.67
7.66
7.65
7.64
7.50
7.50
7.48
7.47
7.47
7.46
7.45
7.45
7.44
7.43
1.71
1.60
H_2N
1i
^{1}H NMR
(400 MHz,$CDCl_3$)
1.15
3.10
1.00
2.10
2.46
6.00
ppm

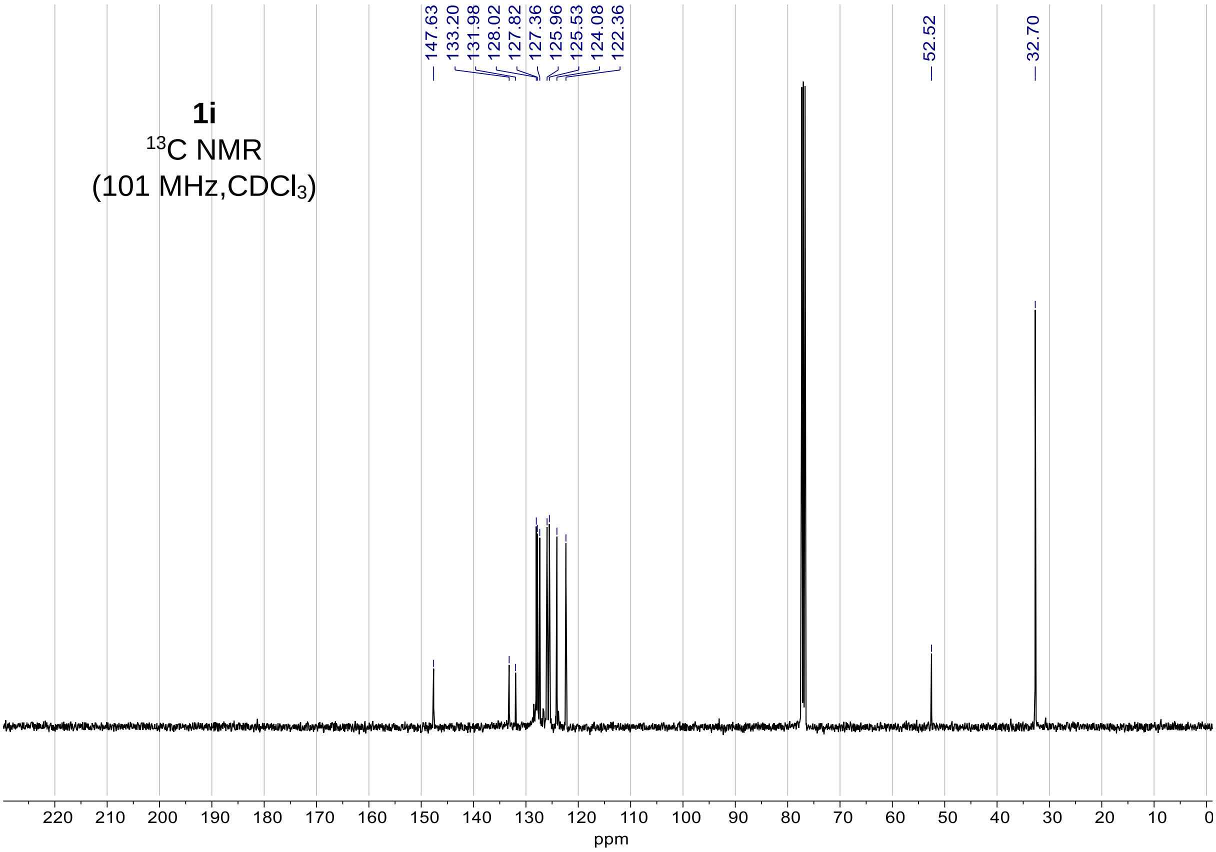
147.63
133.20
131.98
128.02
127.82
127.36
125.96
125.53
124.08
122.36
52.52
32.70
1i
^{13}C NMR
(101 MHz,$CDCl_3$)
ppm

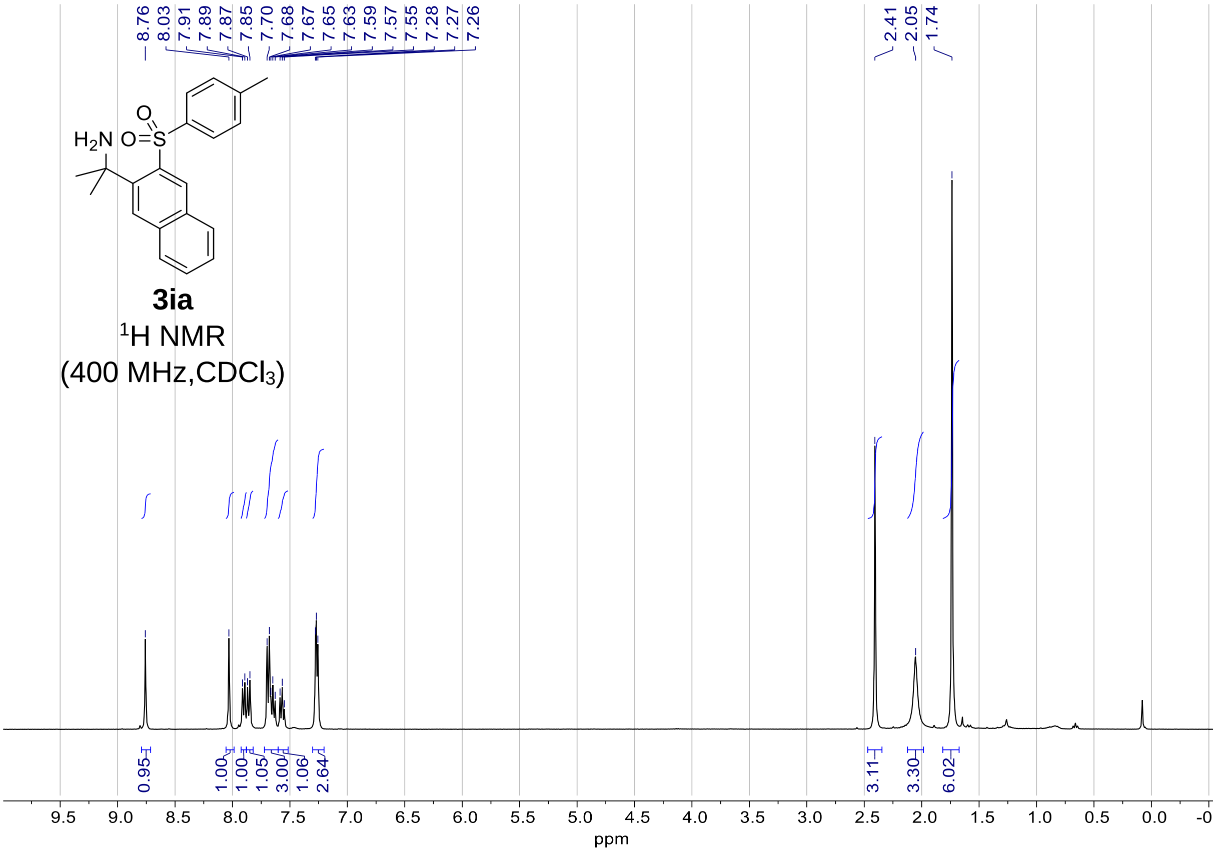
3ia
1H NMR
(400 MHz,CDCl3)
8.76 8.03 7.91 7.89 7.87 7.85 7.70 7.68 7.67 7.65 7.63 7.59 7.57 7.55 7.28 7.27 7.26
2.41 2.05 1.74
0.95 1.00 1.00 1.05 3.00 1.06 2.64 3.11 3.30 6.02
ppm

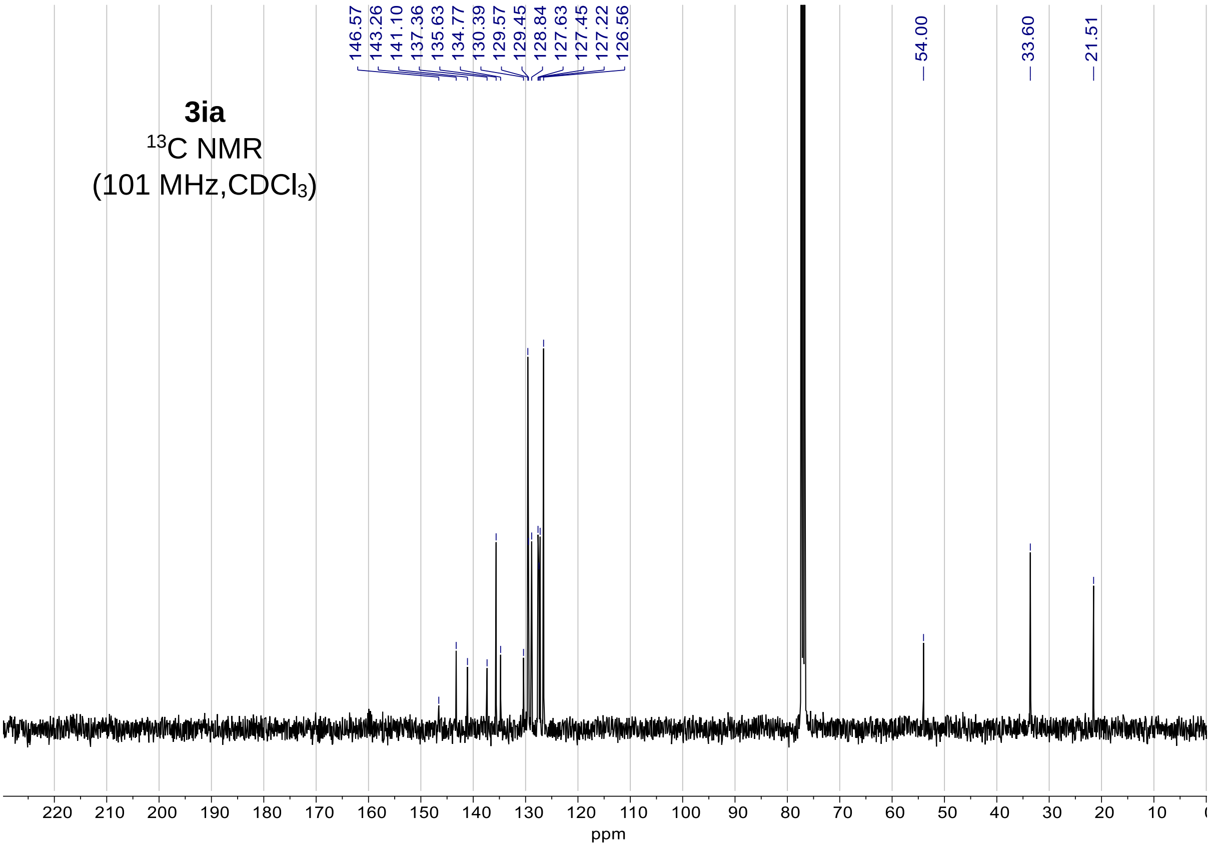
3ia
13C NMR
(101 MHz,CDCl3)
146.57 143.26 141.10 137.36 135.63 134.77 130.39 129.57 129.45 128.84 127.63 127.45 127.22 126.56
54.00 33.60 21.51
ppm

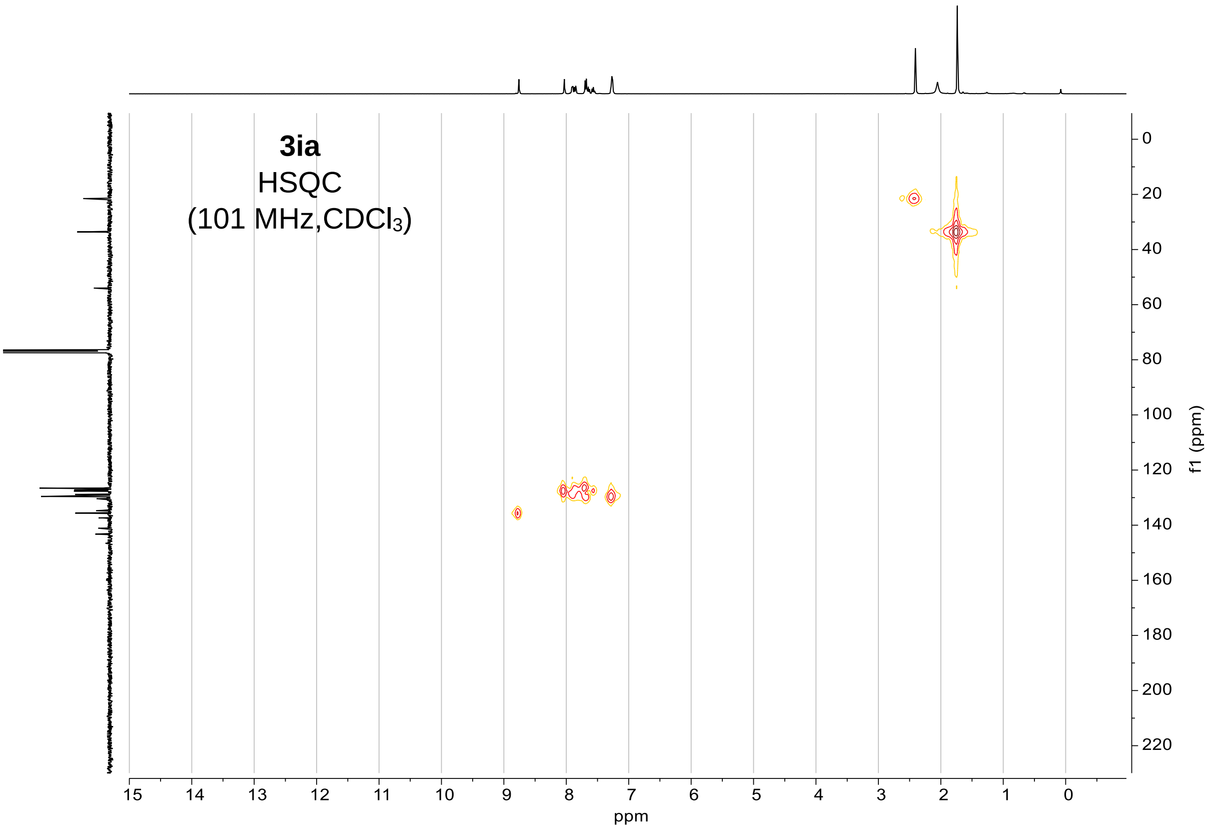
3ia
HSQC
(101 MHz,CDCl3)
ppm
f1 (ppm)

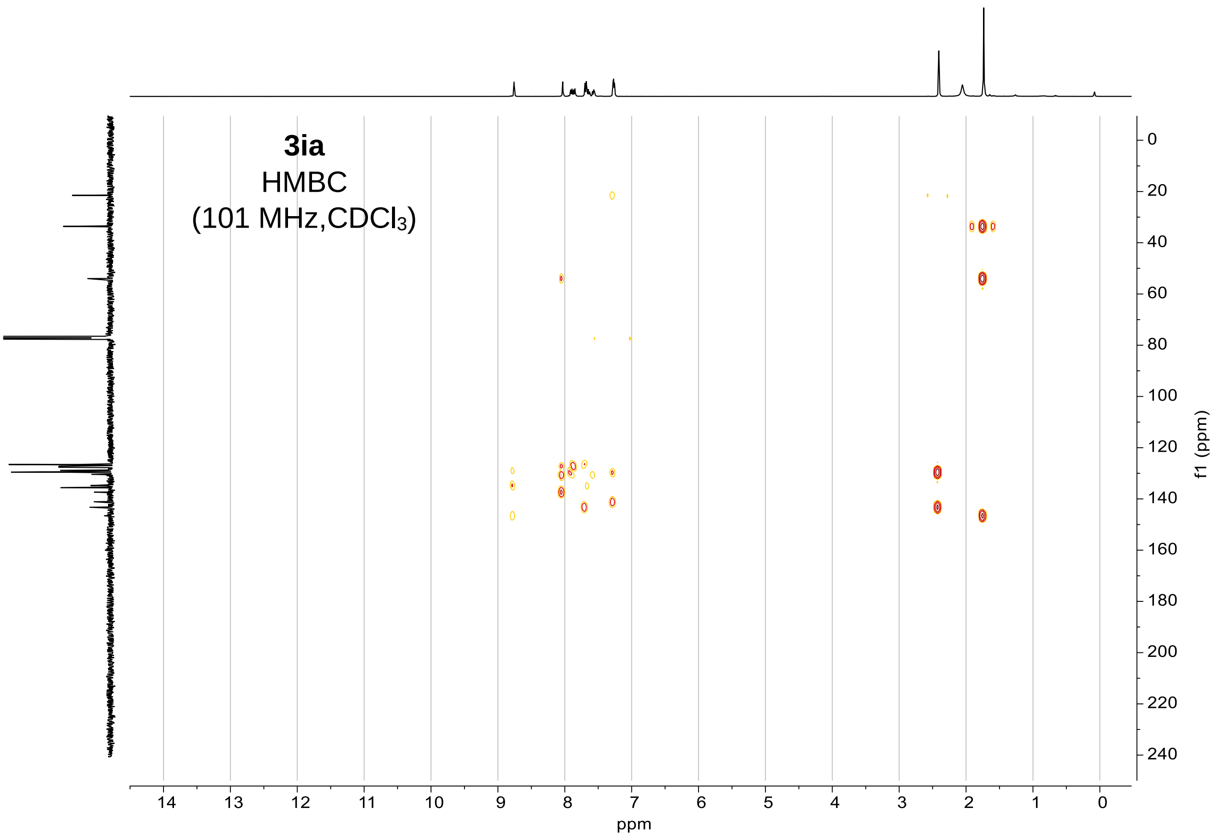
3ia
HMBC
(101 MHz,CDCl3)
ppm
f1 (ppm)

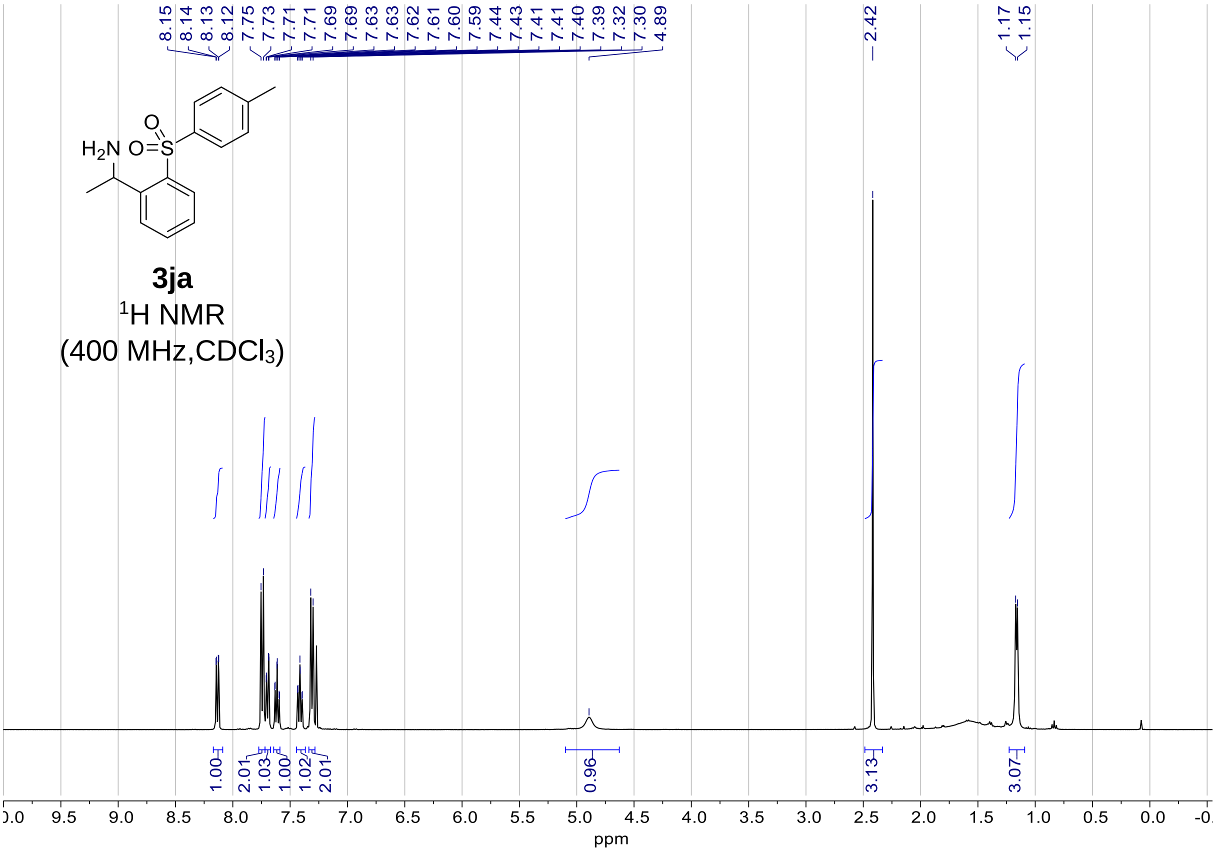
8.15 8.14 8.13 8.12 7.75 7.73 7.71 7.71 7.69 7.63 7.63 7.62 7.61 7.60 7.59 7.44 7.43 7.41 7.41 7.40 7.39 7.32 7.30 4.89
2.42
1.17 1.15
H2N O=S O
3ja
1H NMR
(400 MHz,CDCl3)
1.00 2.01 1.03 1.00 1.02 2.01 0.96 3.13 3.07
ppm

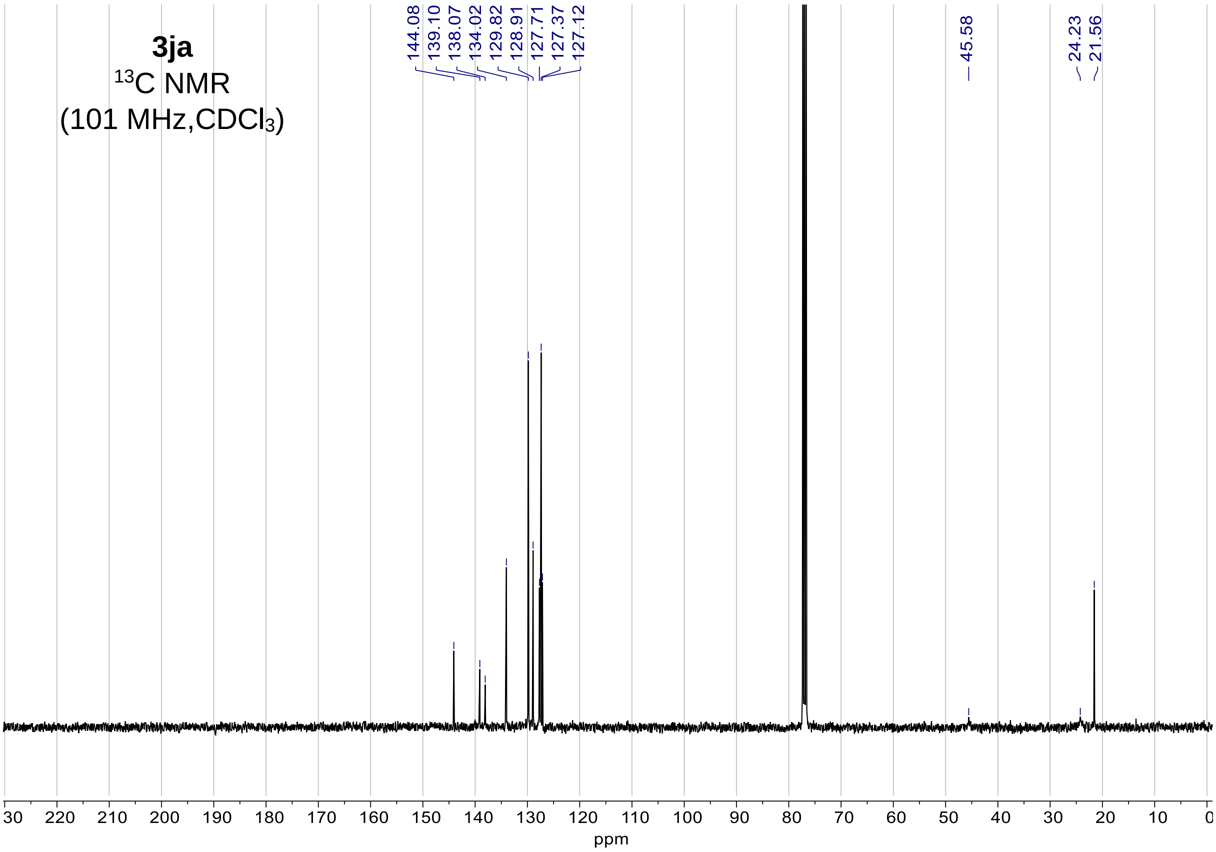
3ja
13C NMR
(101 MHz,CDCl3)
144.08 139.10 138.07 134.02 129.82 128.91 127.71 127.37 127.12
45.58
24.23 21.56
ppm

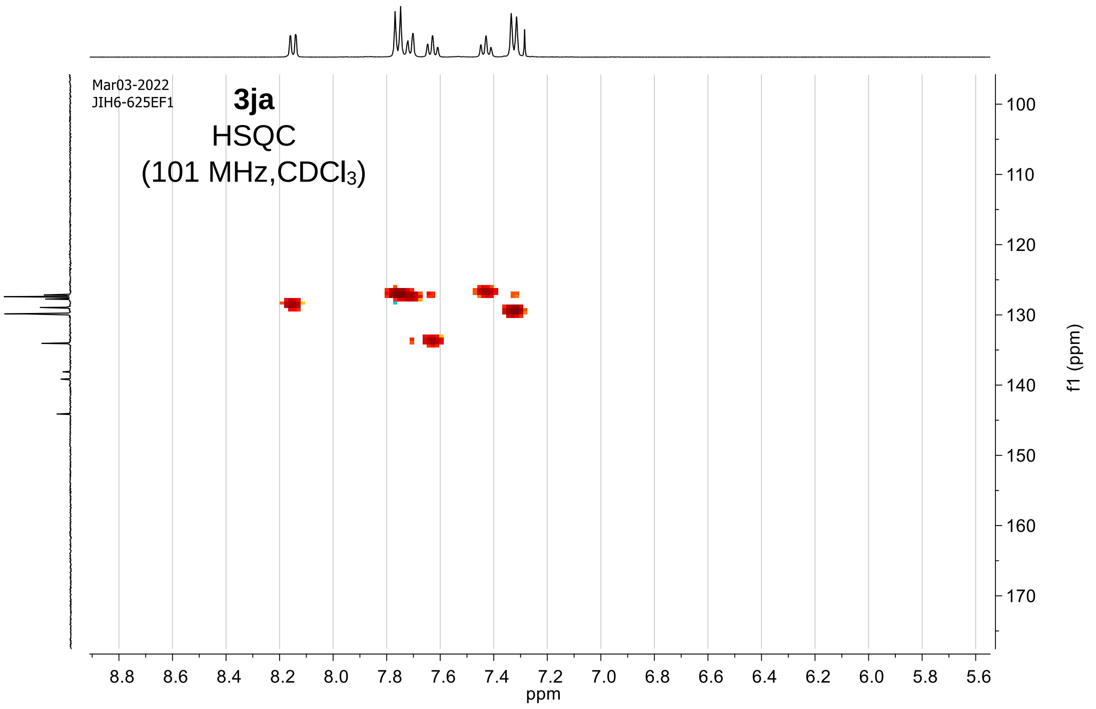
Mar03-2022
JIH6-625EF1
3ja
HSQC
(101 MHz,CDCl3)
f1 (ppm)
ppm

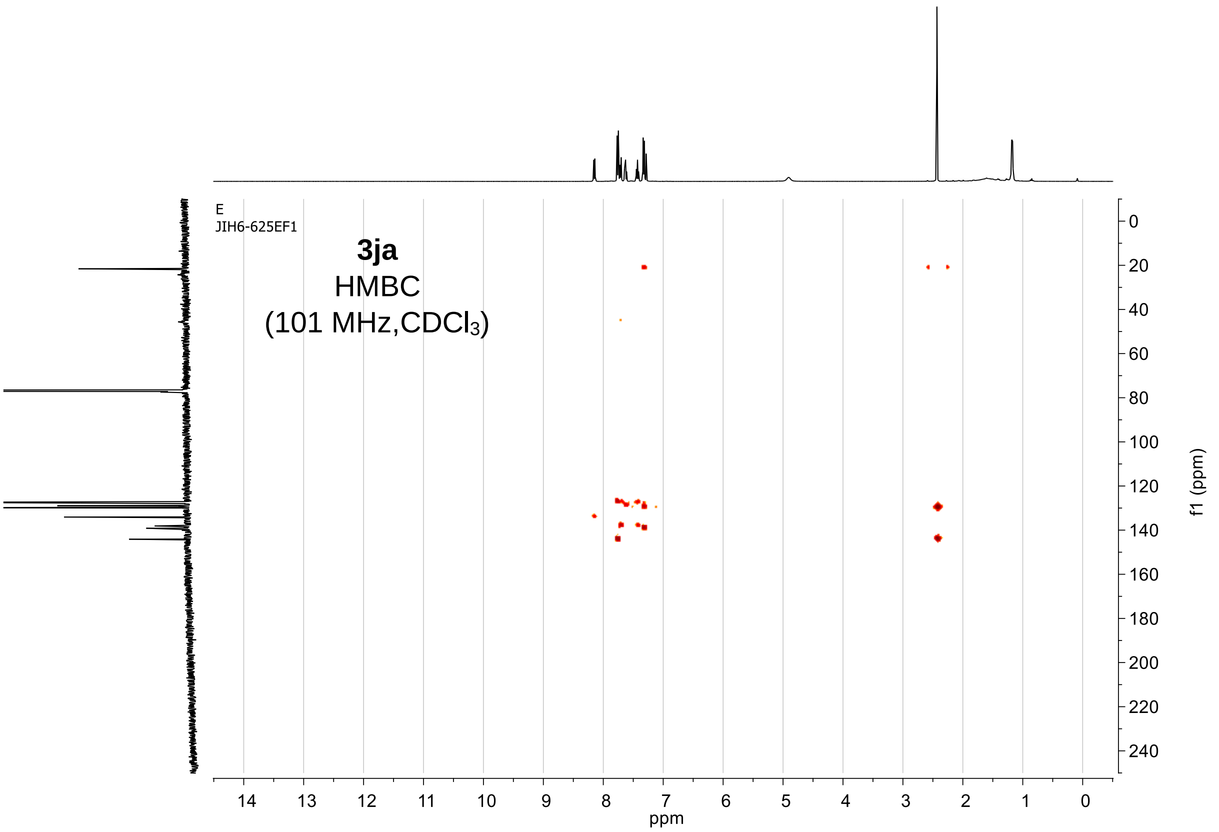
E
JIH6-625EF1
3ja
HMBC
(101 MHz,CDCl3)
f1 (ppm)
ppm

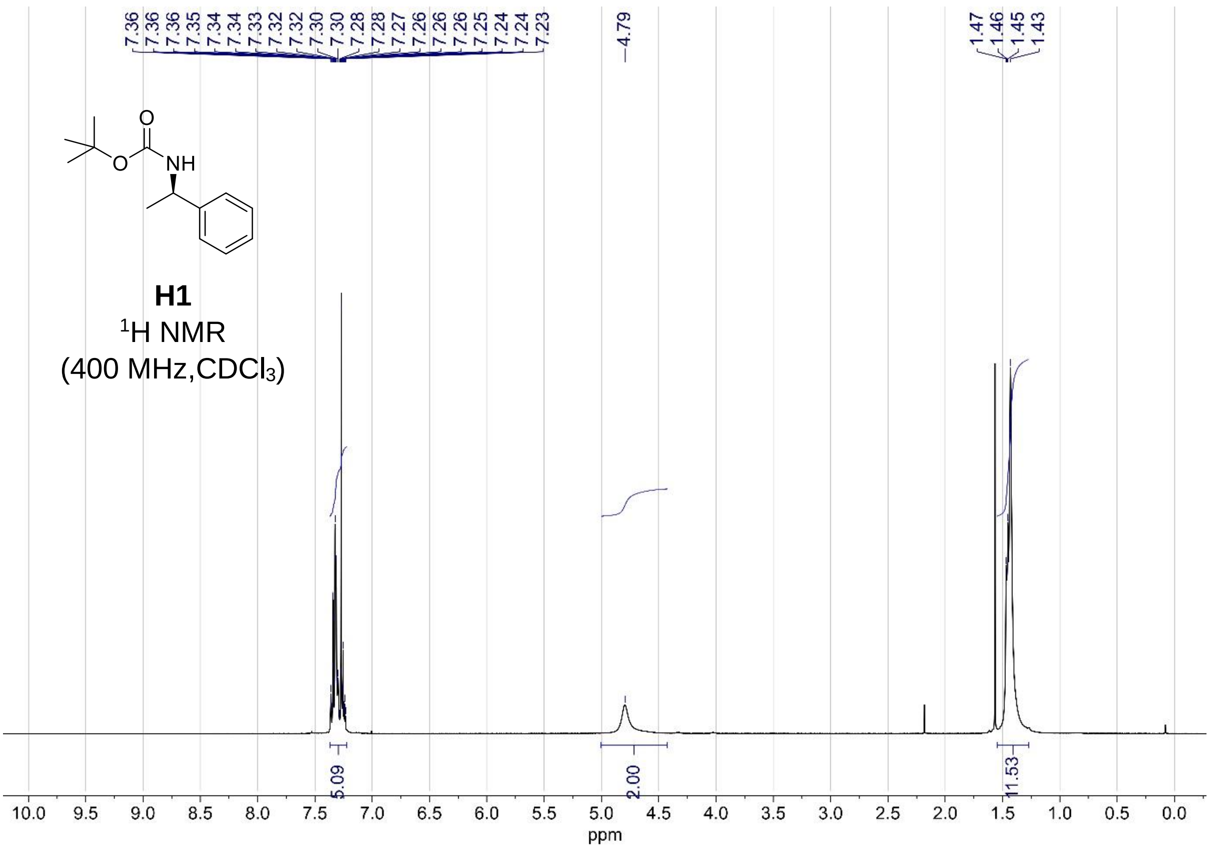
7.36 7.36 7.36 7.35 7.34 7.34 7.33 7.32 7.32 7.30 7.30 7.28 7.28 7.27 7.26 7.26 7.26 7.25 7.24 7.24 7.23
4.79
1.47 1.46 1.45 1.43
O
NH
O
H1
1H NMR
(400 MHz,CDCl3)
5.09
2.00
11.53
10.0 9.5 9.0 8.5 8.0 7.5 7.0 6.5 6.0 5.5 5.0 4.5 4.0 3.5 3.0 2.5 2.0 1.5 1.0 0.5 0.0
ppm

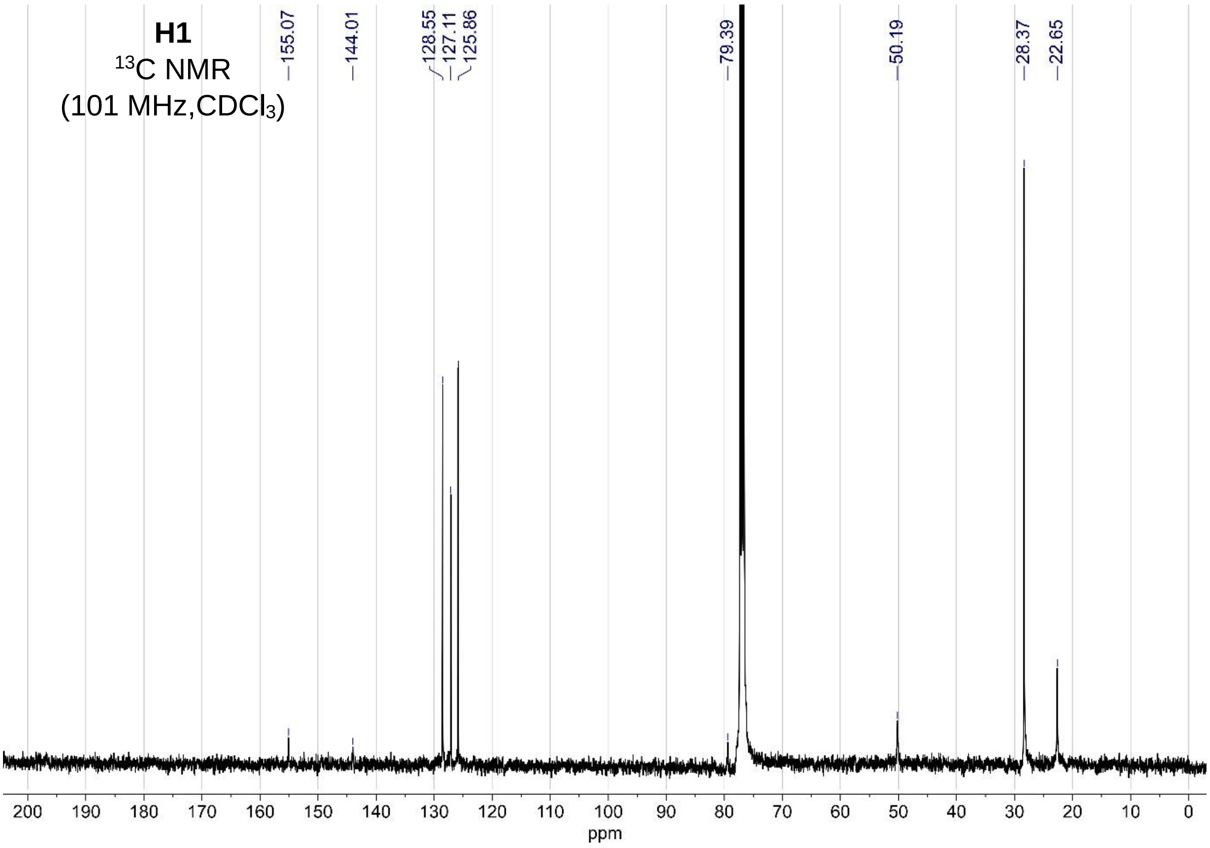
H1
13C NMR
(101 MHz,CDCl3)
155.07
144.01
128.55 127.11 125.86
79.39
50.19
28.37
22.65
200 190 180 170 160 150 140 130 120 110 100 90 80 70 60 50 40 30 20 10 0
ppm

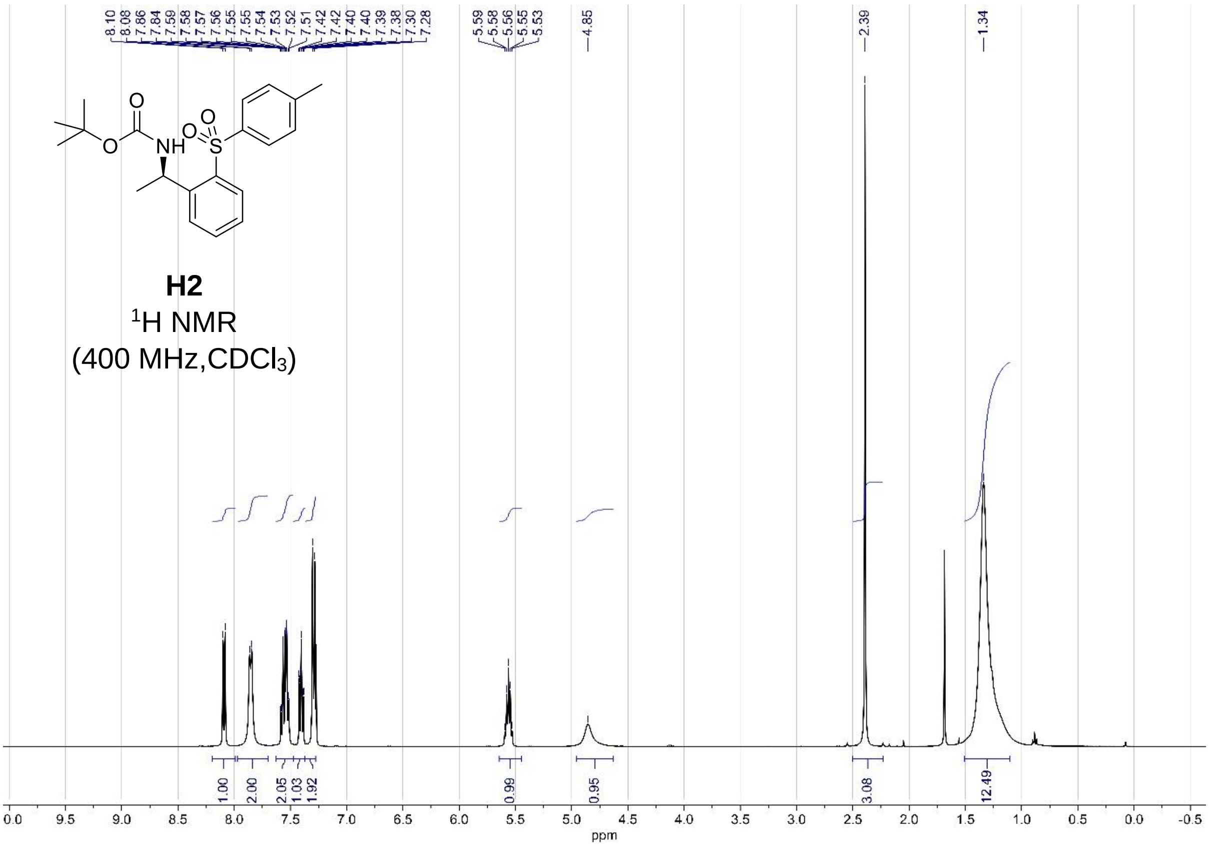
H2
^{1}H NMR
(400 MHz,CDCl$_3$)
ppm

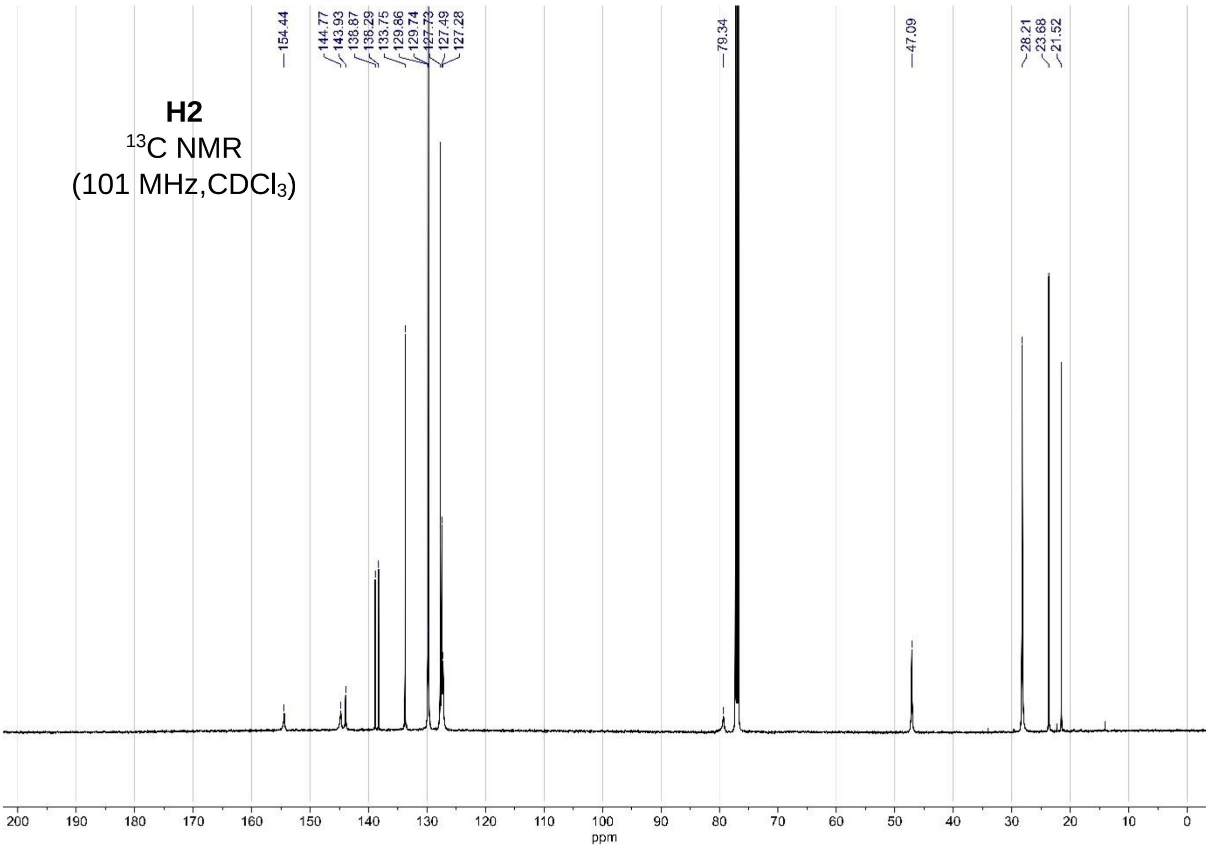
H2
^{13}C NMR
(101 MHz,CDCl$_3$)
ppm

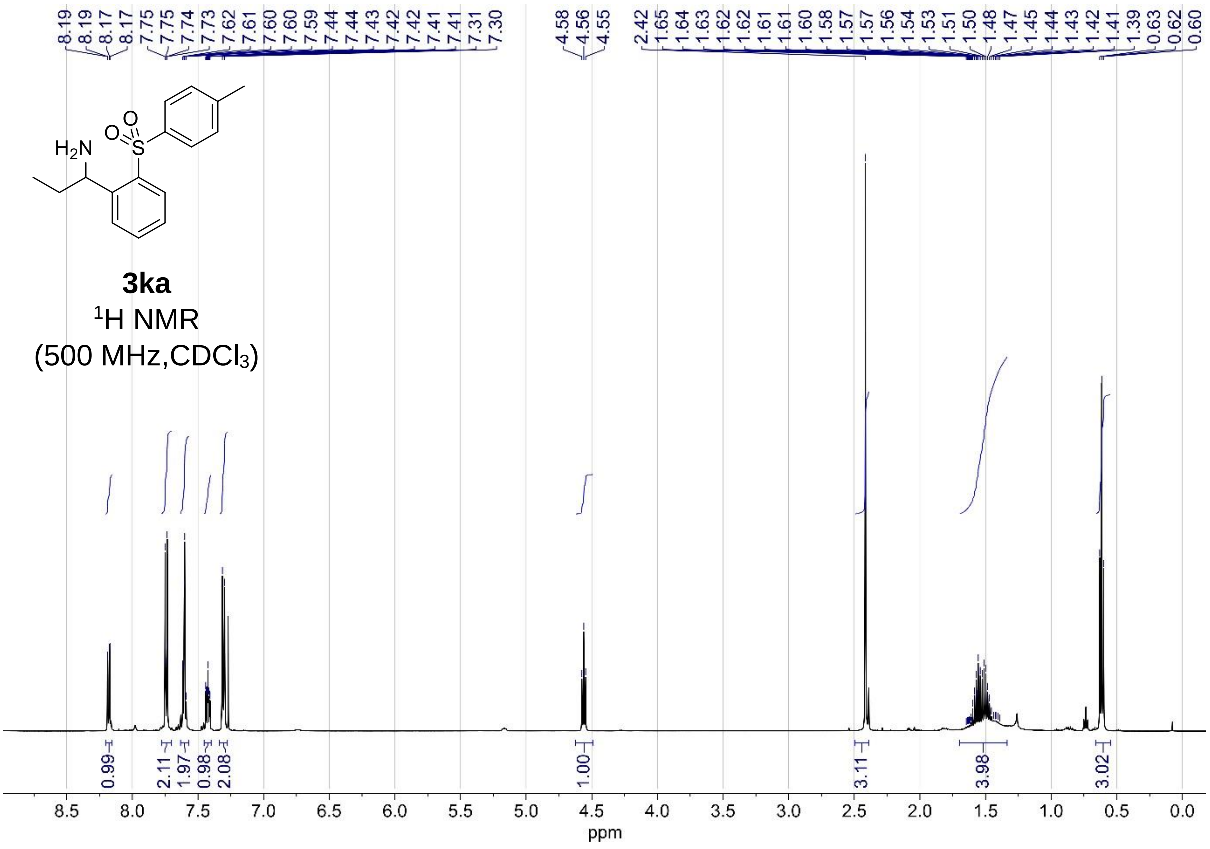

8.19 8.19 8.17 8.17 7.75 7.75 7.74 7.73 7.62 7.61 7.60 7.59 7.44 7.44 7.43 7.42 7.42 7.41 7.41 7.31 7.30
4.58 4.56 4.55
2.42 1.65 1.64 1.63 1.62 1.62 1.61 1.61 1.60 1.58 1.57 1.57 1.56 1.54 1.53 1.51 1.50 1.48 1.47 1.45 1.44 1.43 1.42 1.41 1.39 0.63 0.62 0.60
H2N
O
O
S
3ka
1H NMR
(500 MHz,CDCl3)
0.99 2.11 1.97 0.98 2.08 1.00 3.11 3.98 3.02
8.5 8.0 7.5 7.0 6.5 6.0 5.5 5.0 4.5 4.0 3.5 3.0 2.5 2.0 1.5 1.0 0.5 0.0
ppm

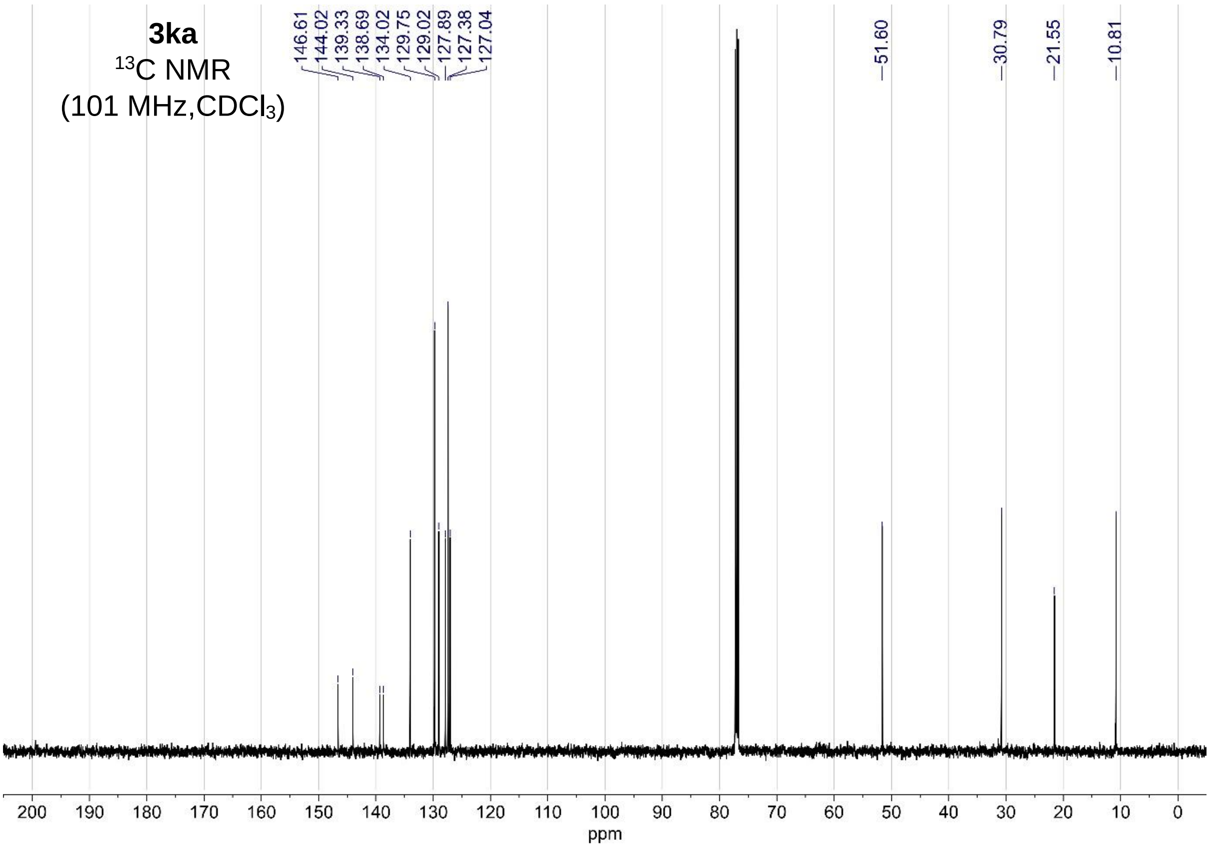

3ka
13C NMR
(101 MHz,CDCl3)
146.61 144.02 139.33 138.69 134.02 129.75 129.02 127.89 127.38 127.04
51.60
30.79
21.55
10.81
200 190 180 170 160 150 140 130 120 110 100 90 80 70 60 50 40 30 20 10 0
ppm

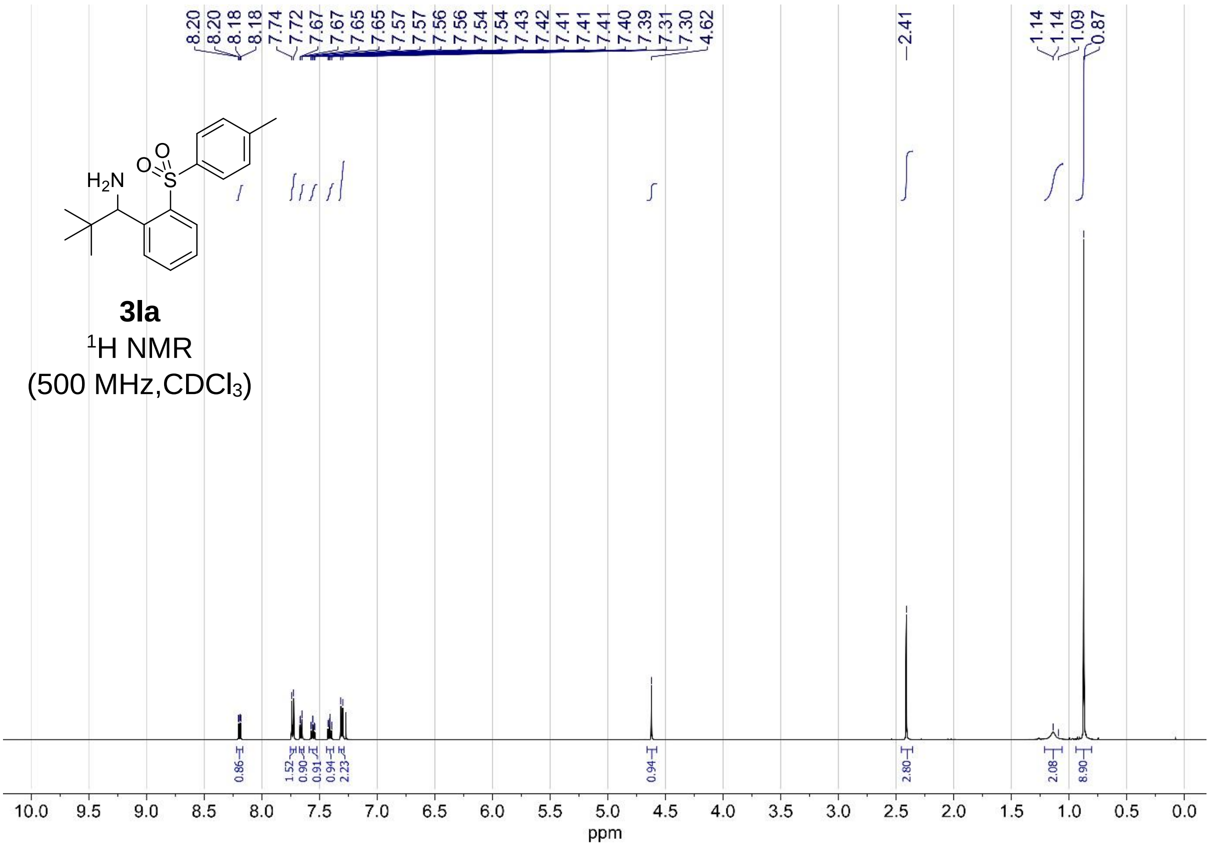
3la
^{1}H NMR
(500 MHz, $CDCl_3$)
H_2N
O
O
S
8.20 8.20 8.18 8.18 7.74 7.72 7.67 7.67 7.65 7.65 7.57 7.57 7.56 7.56 7.54 7.54 7.43 7.42 7.41 7.41 7.41 7.40 7.39 7.31 7.30 4.62
2.41
1.14 1.14 1.09 0.87
0.86 1.52 0.90 0.91 0.94 2.23 0.94 2.80 2.08 8.90
10.0 9.5 9.0 8.5 8.0 7.5 7.0 6.5 6.0 5.5 5.0 4.5 4.0 3.5 3.0 2.5 2.0 1.5 1.0 0.5 0.0
ppm

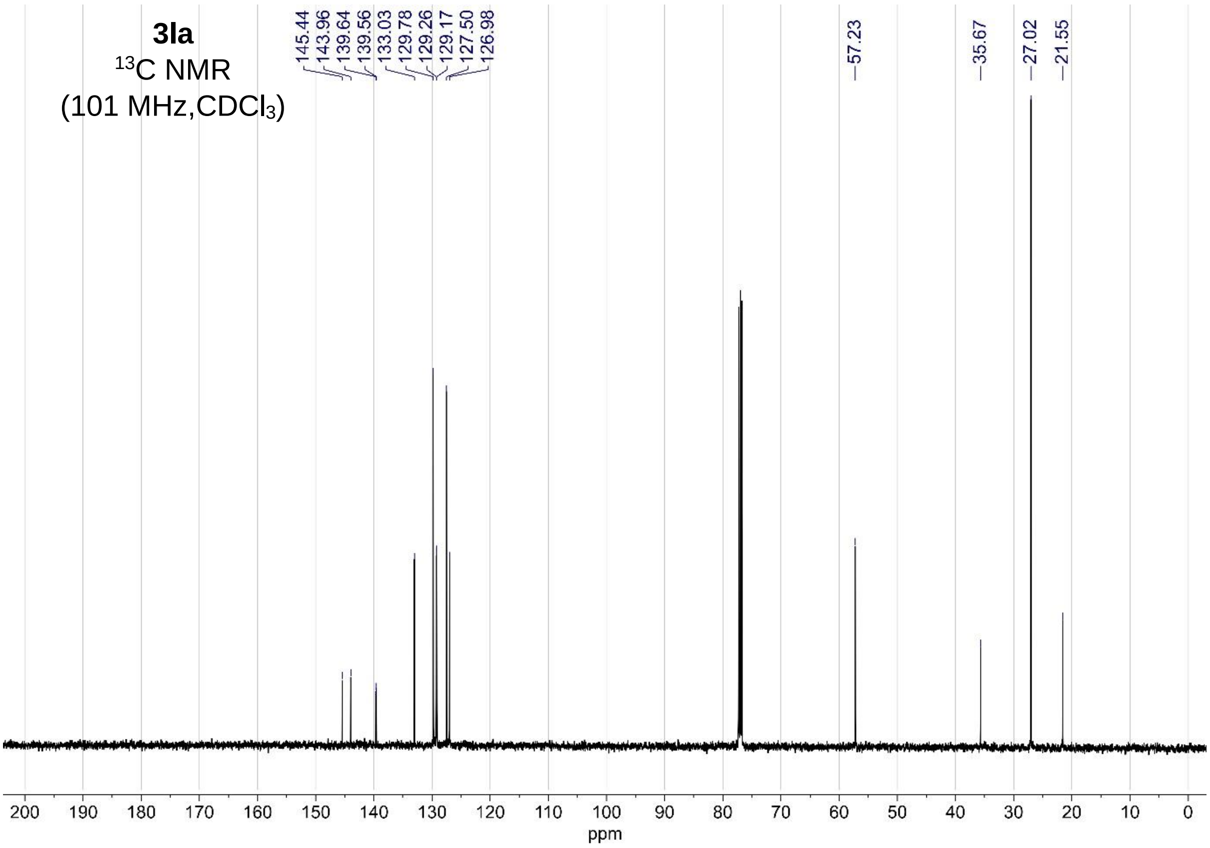
3la
^{13}C NMR
(101 MHz, $CDCl_3$)
145.44 143.96 139.64 139.56 133.03 129.78 129.26 129.17 127.50 126.98
57.23
35.67
27.02
21.55
200 190 180 170 160 150 140 130 120 110 100 90 80 70 60 50 40 30 20 10 0
ppm

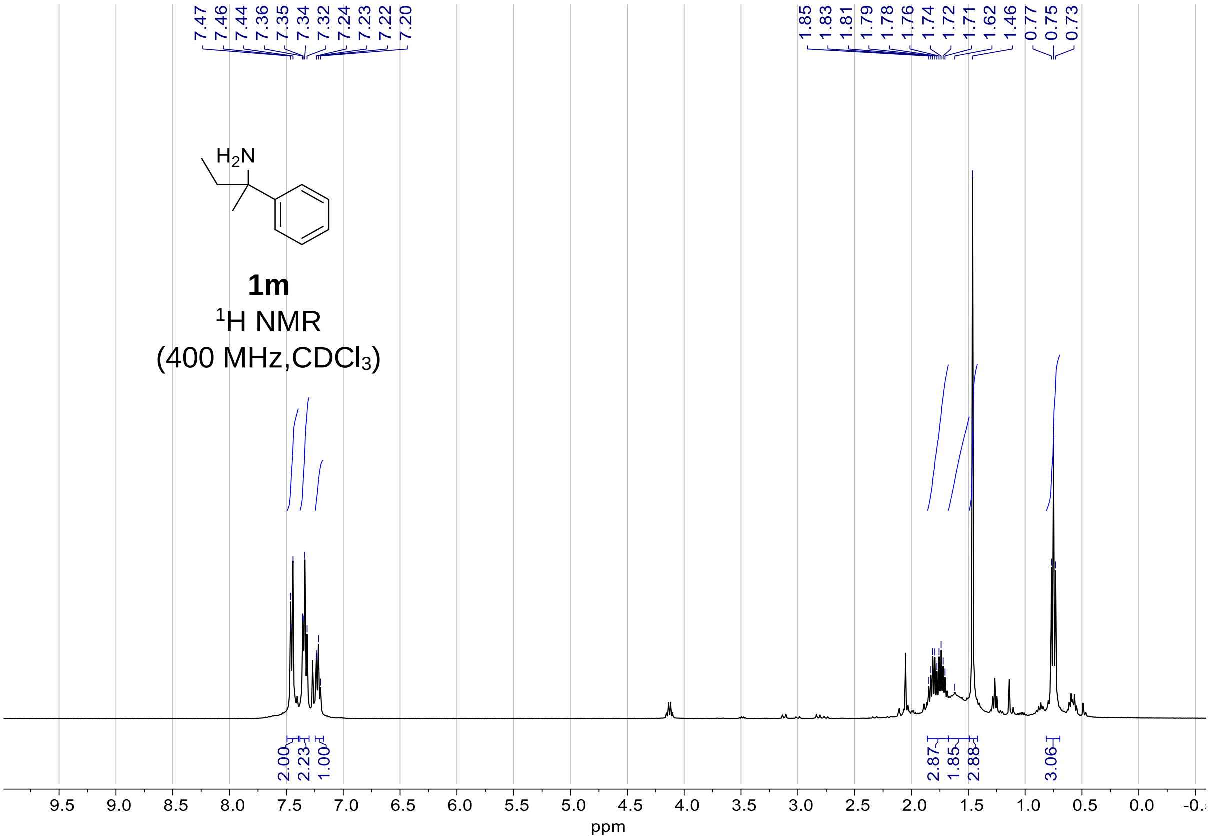
7.47
7.46
7.44
7.36
7.35
7.34
7.32
7.24
7.23
7.22
7.20
1.85
1.83
1.81
1.79
1.78
1.76
1.74
1.72
1.71
1.62
1.46
0.77
0.75
0.73
H2N
1m
1H NMR
(400 MHz,CDCl3)
2.00
2.23
1.00
2.87
1.85
2.88
3.06
9.5 9.0 8.5 8.0 7.5 7.0 6.5 6.0 5.5 5.0 4.5 4.0 3.5 3.0 2.5 2.0 1.5 1.0 0.5 0.0
ppm

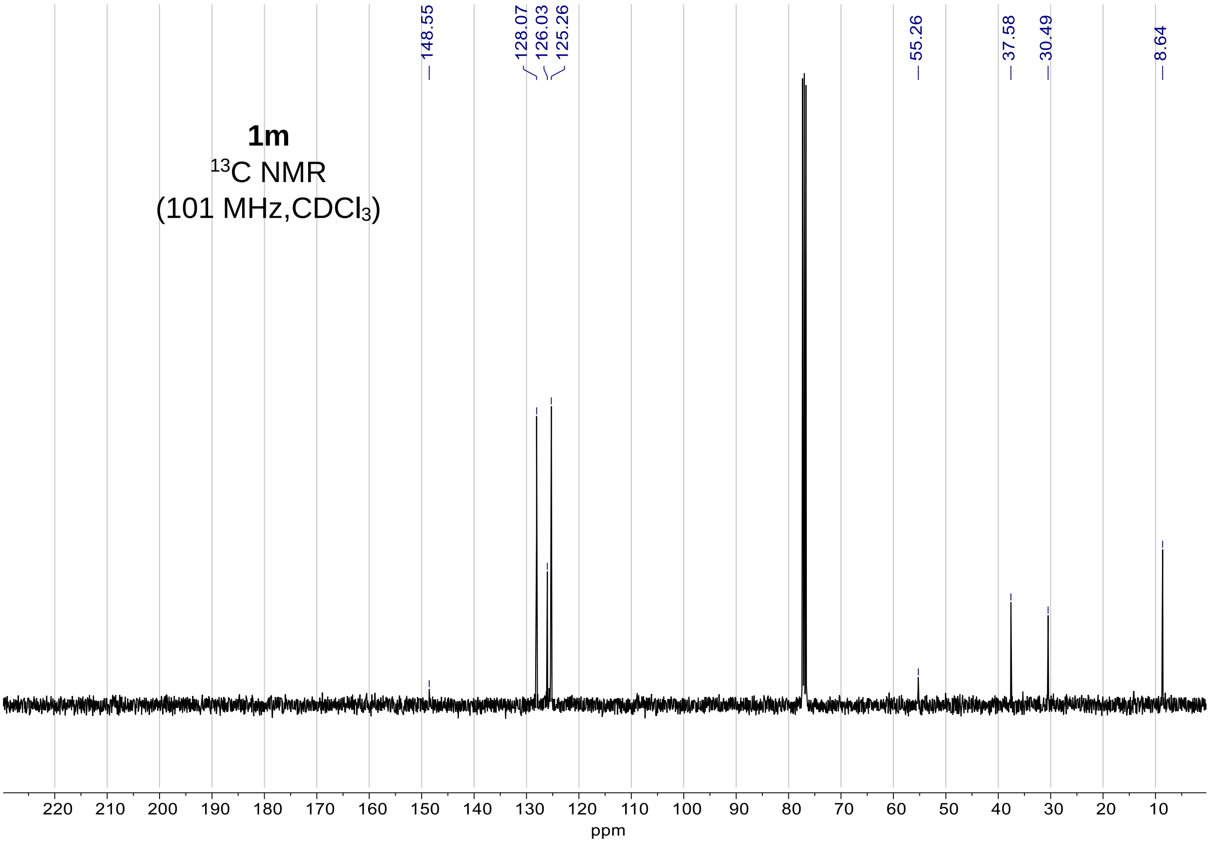
148.55
128.07
126.03
125.26
55.26
37.58
30.49
8.64
1m
13C NMR
(101 MHz,CDCl3)
220 210 200 190 180 170 160 150 140 130 120 110 100 90 80 70 60 50 40 30 20 10
ppm

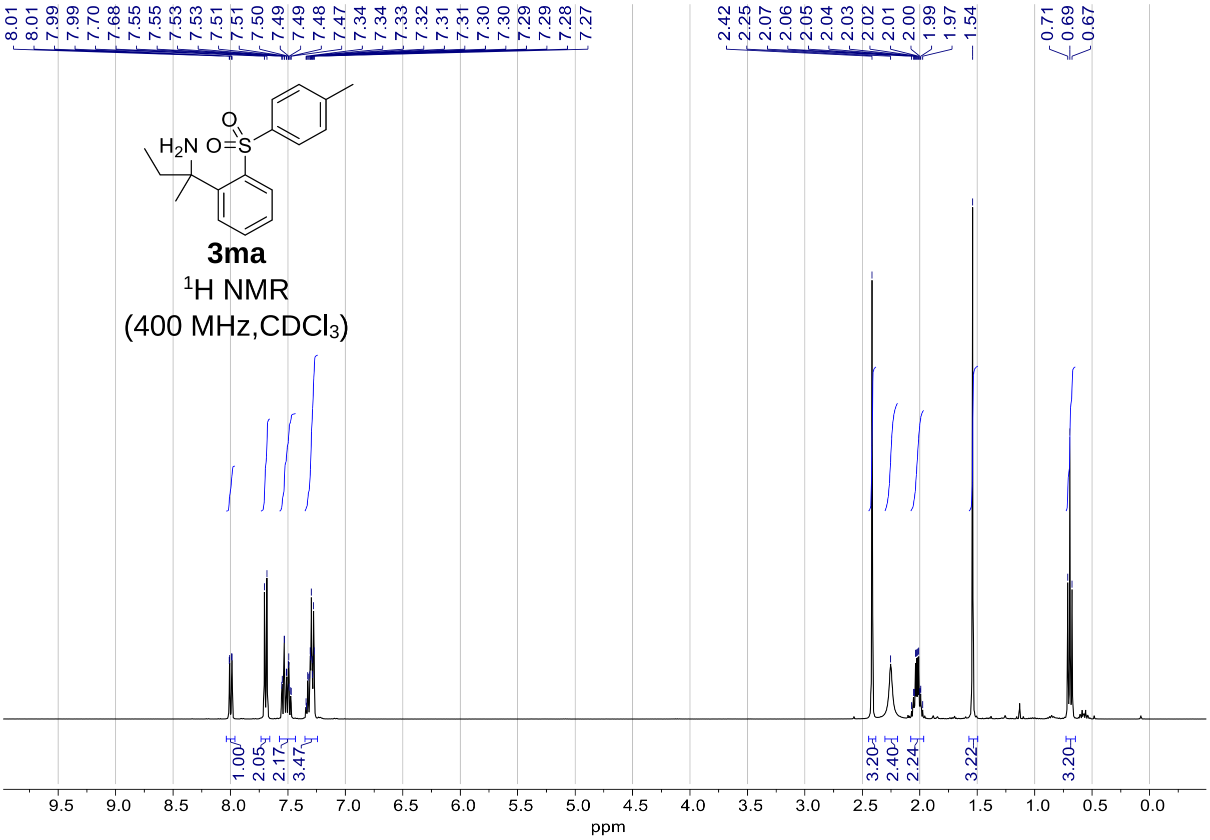
8.01 8.01 7.99 7.70 7.68 7.55 7.55 7.53 7.53 7.51 7.51 7.50 7.49 7.49 7.48 7.47 7.34 7.34 7.33 7.32 7.31 7.31 7.30 7.30 7.29 7.29 7.28 7.27
2.42 2.25 2.07 2.06 2.05 2.04 2.03 2.02 2.01 2.00 1.99 1.97 1.54
0.71 0.69 0.67
H2N O=S O
3ma
1H NMR
(400 MHz,CDCl3)
1.00 2.05 2.17 3.47
3.20 2.40 2.24 3.22 3.20
9.5 9.0 8.5 8.0 7.5 7.0 6.5 6.0 5.5 5.0 4.5 4.0 3.5 3.0 2.5 2.0 1.5 1.0 0.5 0.0
ppm

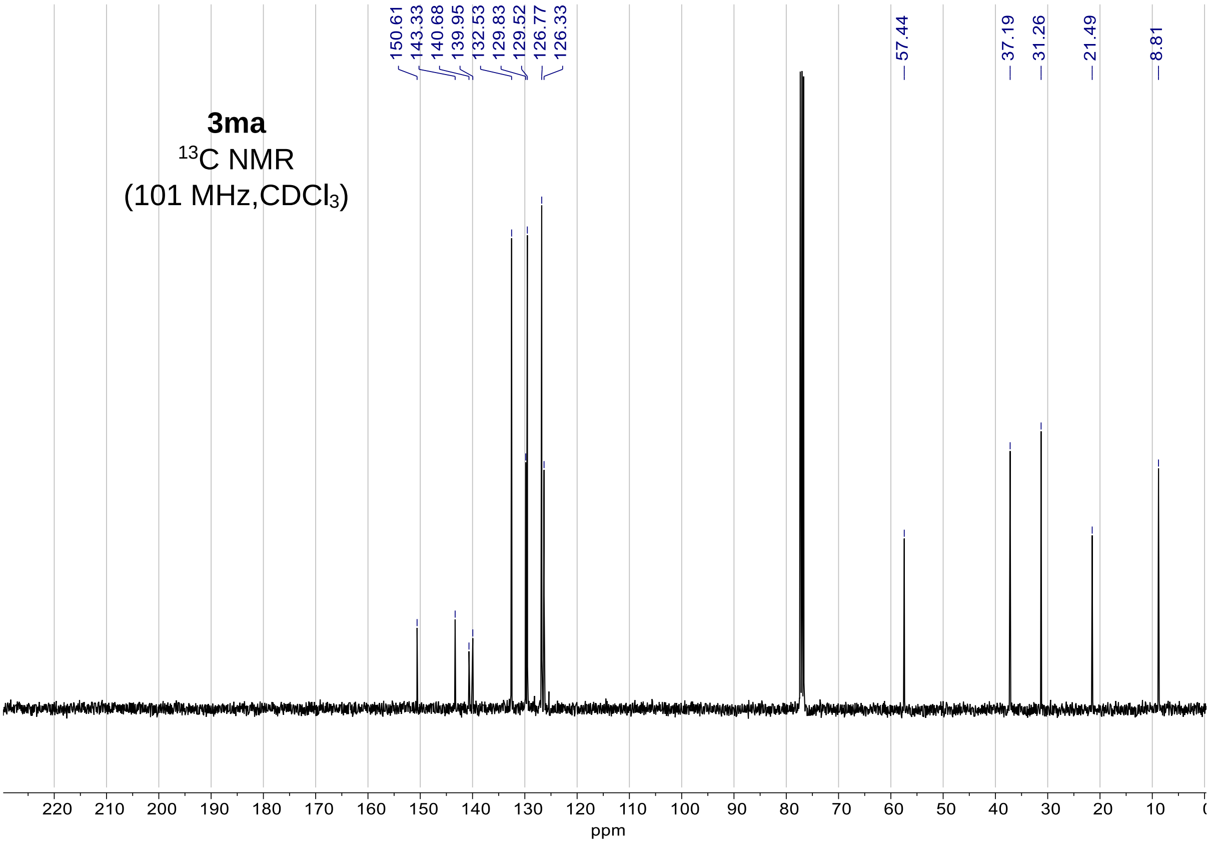
150.61 143.33 140.68 139.95 132.53 129.83 129.52 126.77 126.33
57.44
37.19 31.26 21.49 8.81
3ma
13C NMR
(101 MHz,CDCl3)
220 210 200 190 180 170 160 150 140 130 120 110 100 90 80 70 60 50 40 30 20 10
ppm

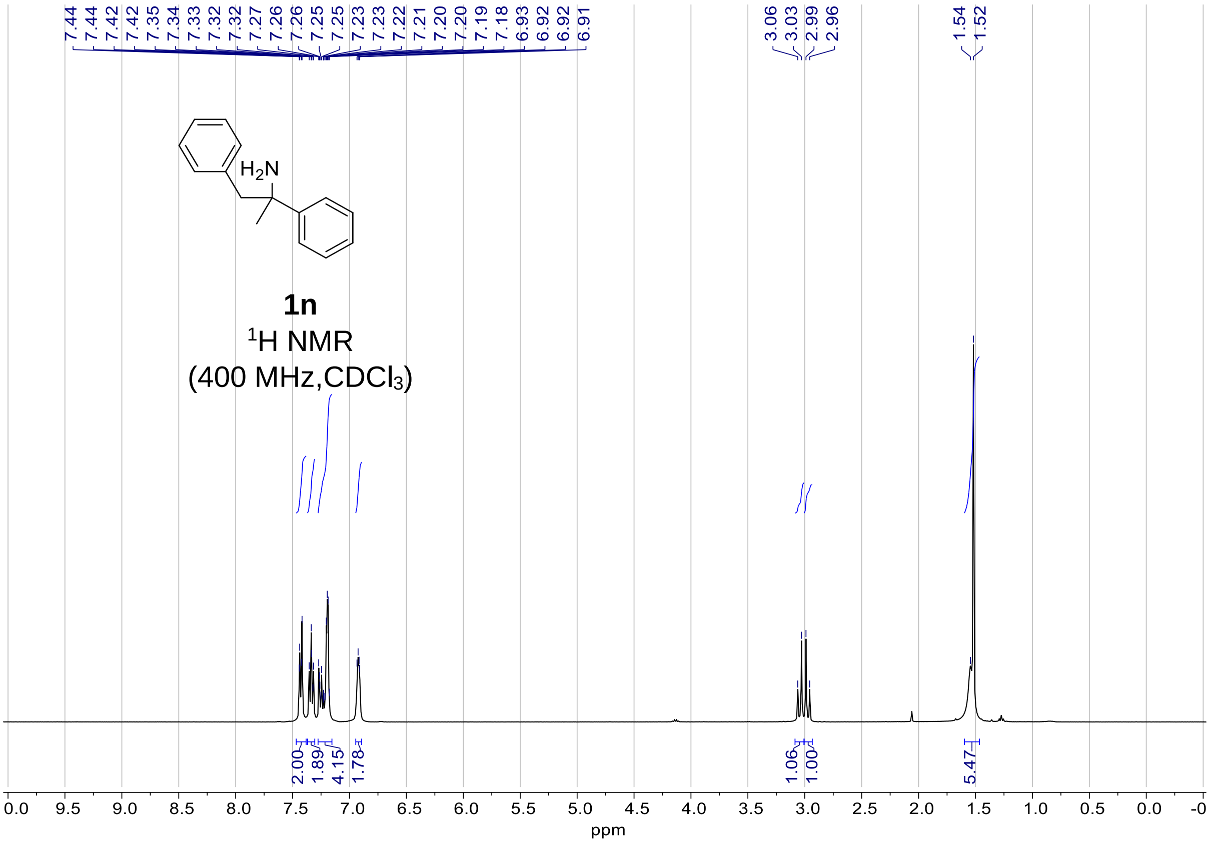
H2N
1n
1H NMR
(400 MHz,CDCl3)
7.44
7.44
7.42
7.35
7.34
7.33
7.32
7.32
7.27
7.26
7.25
7.25
7.23
7.23
7.22
7.21
7.20
7.20
7.19
7.18
6.93
6.92
6.92
6.91
3.06
3.03
2.99
2.96
1.54
1.52
2.00
1.89
4.15
1.78
1.06
1.00
5.47
10.0 9.5 9.0 8.5 8.0 7.5 7.0 6.5 6.0 5.5 5.0 4.5 4.0 3.5 3.0 2.5 2.0 1.5 1.0 0.5 0.0 -0
ppm

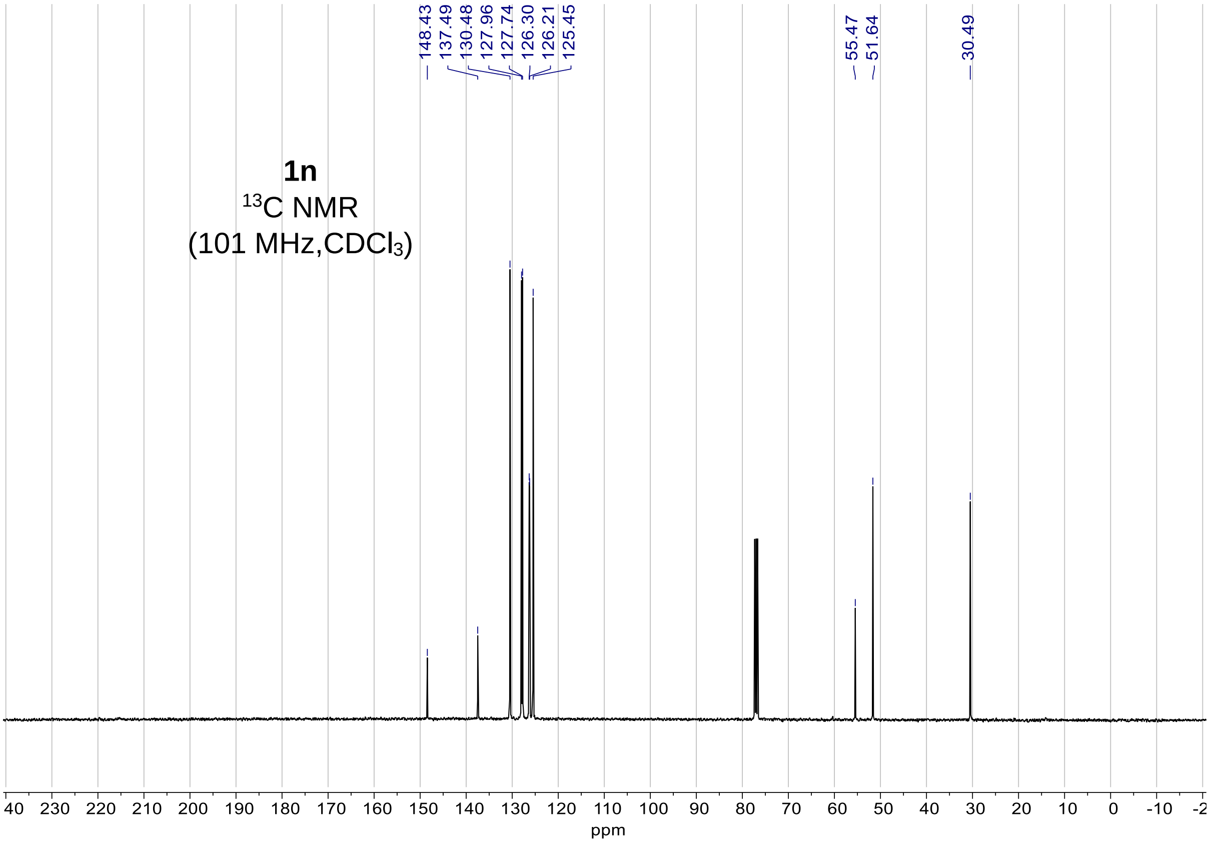
148.43
137.49
130.48
127.96
127.74
126.30
126.21
125.45
55.47
51.64
30.49
1n
13C NMR
(101 MHz,CDCl3)
240 230 220 210 200 190 180 170 160 150 140 130 120 110 100 90 80 70 60 50 40 30 20 10 0 -10 -2
ppm

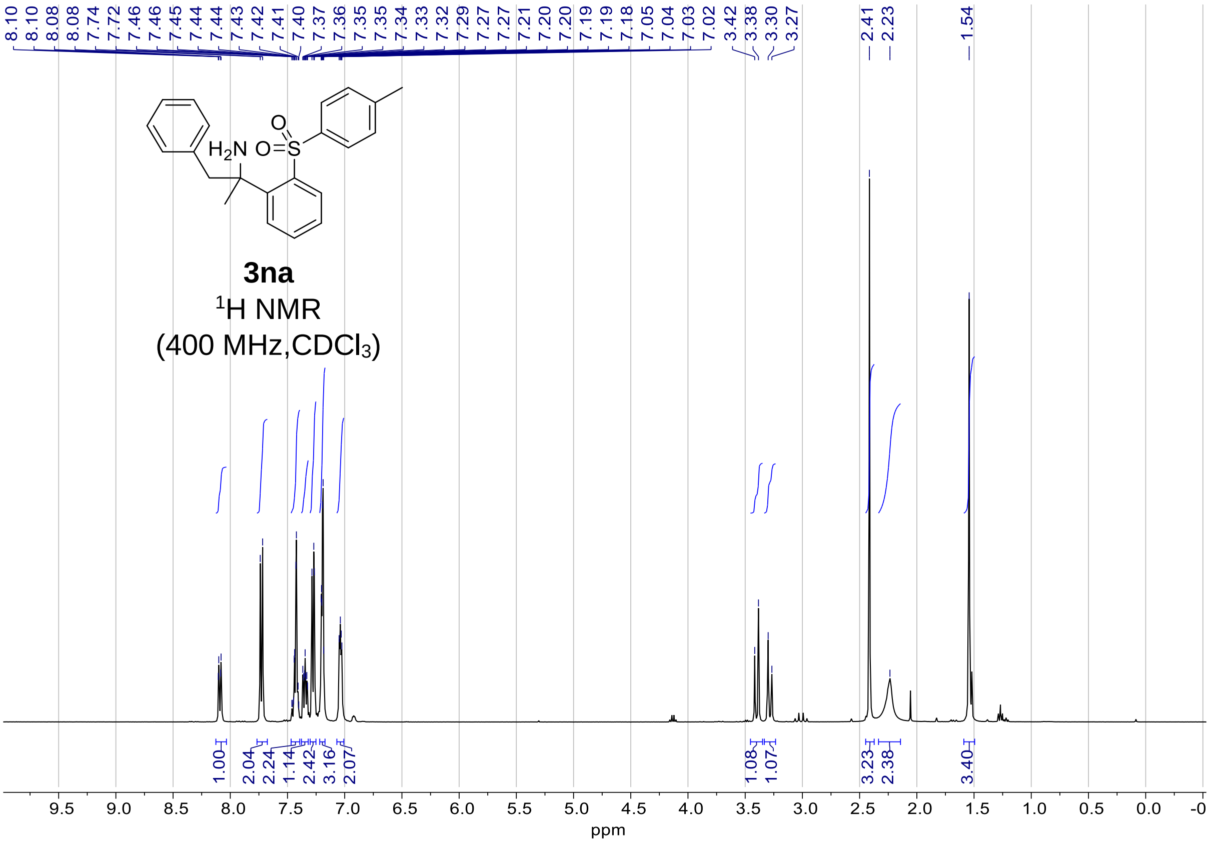
8.10 8.10 8.08 8.08 7.74 7.72 7.46 7.45 7.44 7.44 7.43 7.42 7.41 7.40 7.37 7.36 7.35 7.35 7.34 7.33 7.32 7.29 7.27 7.27 7.21 7.20 7.20 7.19 7.19 7.18 7.05 7.04 7.03 7.02 3.42 3.38 3.30 3.27 2.41 2.23 1.54
H_2N
O
O=S
3na
^{1}H NMR
(400 MHz,$CDCl_3$)
1.00 2.04 2.24 1.14 2.42 3.16 2.07 1.08 1.07 3.23 2.38 3.40
ppm

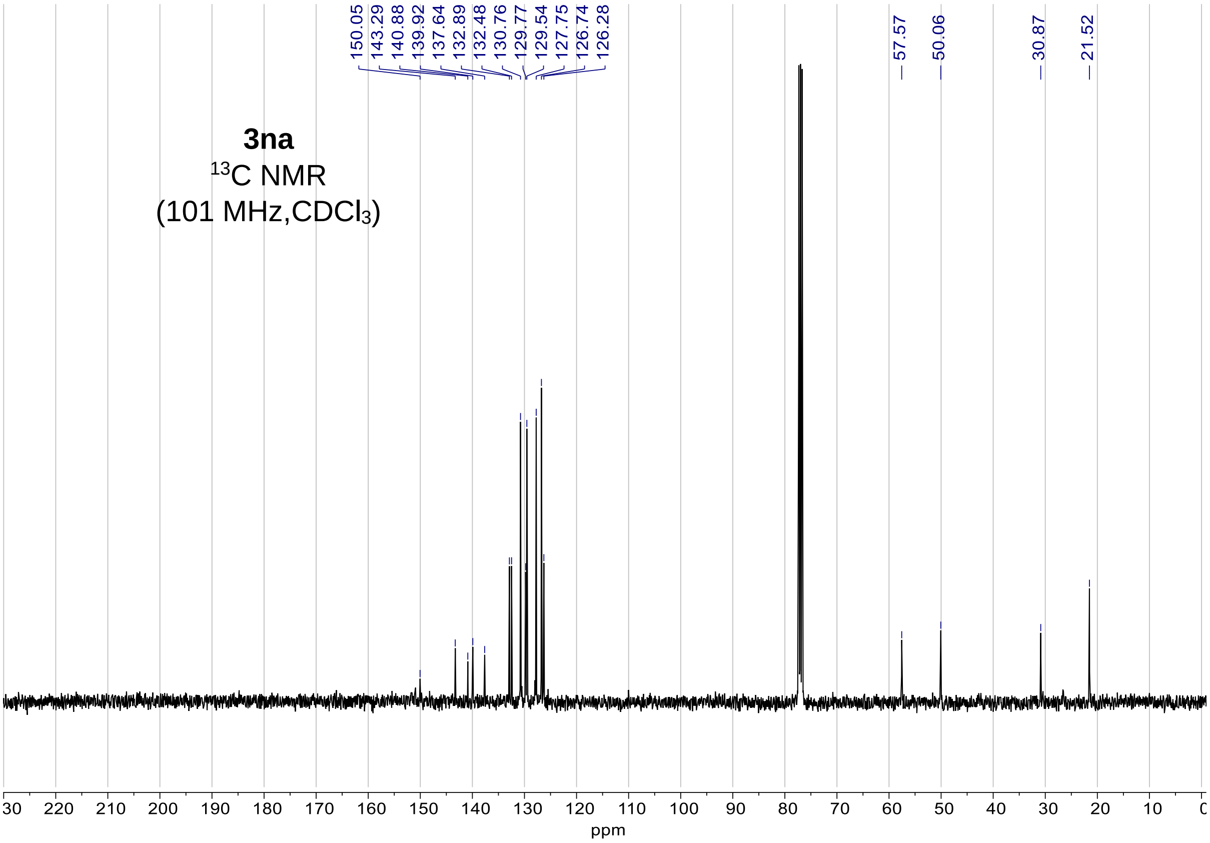
150.05 143.29 140.88 139.92 137.64 132.89 132.48 130.76 129.77 129.54 127.75 126.74 126.28 57.57 50.06 30.87 21.52
3na
^{13}C NMR
(101 MHz,$CDCl_3$)
ppm

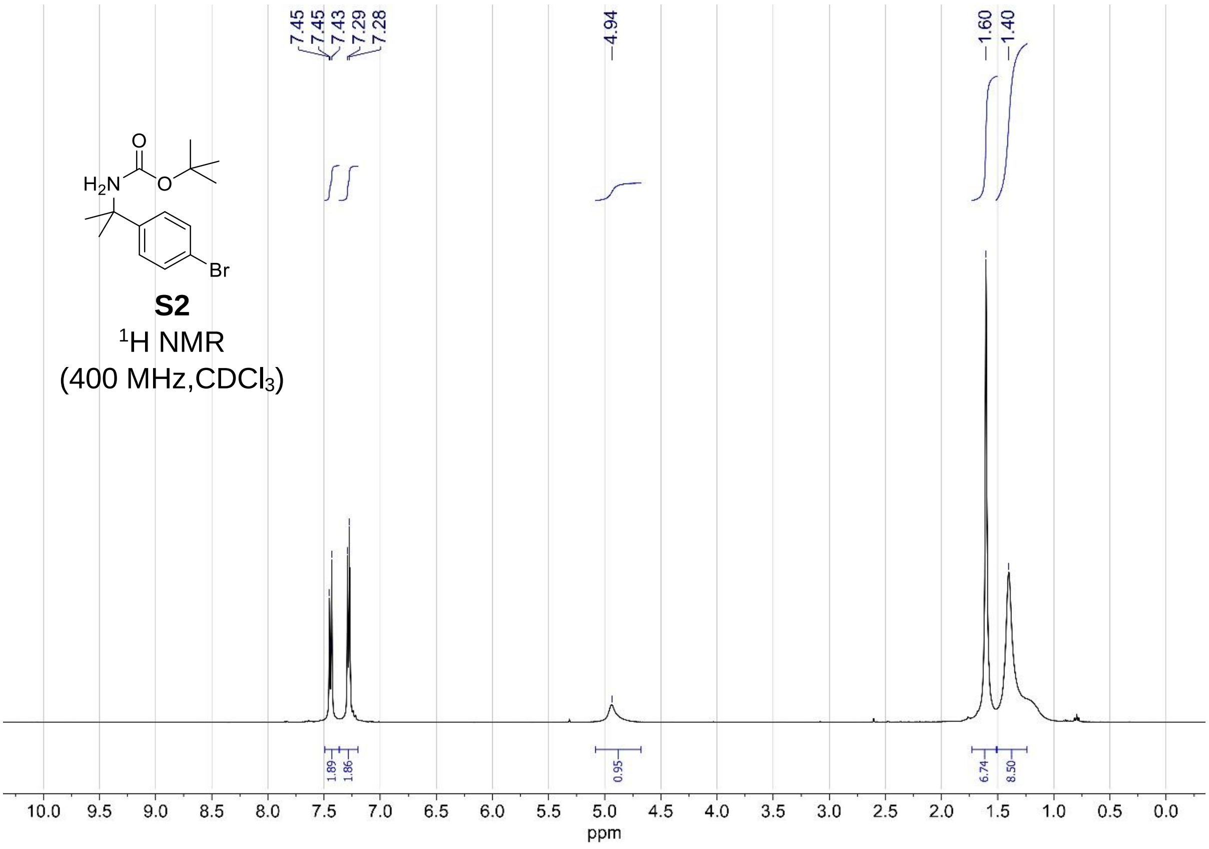
S2
1H NMR
(400 MHz,CDCl3)
H2N
O
O
Br
7.45
7.45
7.43
7.29
7.28
4.94
1.60
1.40
1.89
1.86
0.95
6.74
8.50
10.0 9.5 9.0 8.5 8.0 7.5 7.0 6.5 6.0 5.5 5.0 4.5 4.0 3.5 3.0 2.5 2.0 1.5 1.0 0.5 0.0
ppm

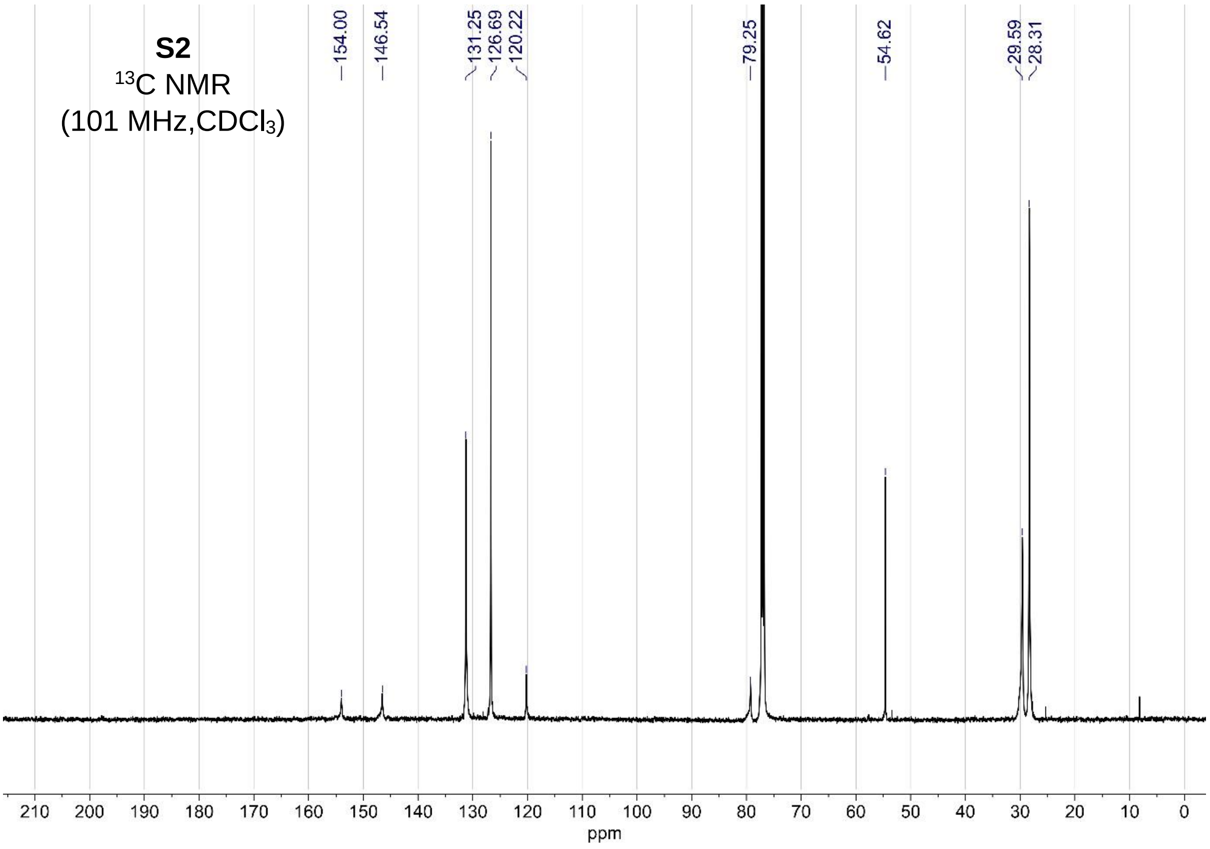
S2
13C NMR
(101 MHz,CDCl3)
154.00
146.54
131.25
126.69
120.22
79.25
54.62
29.59
28.31
210 200 190 180 170 160 150 140 130 120 110 100 90 80 70 60 50 40 30 20 10 0
ppm

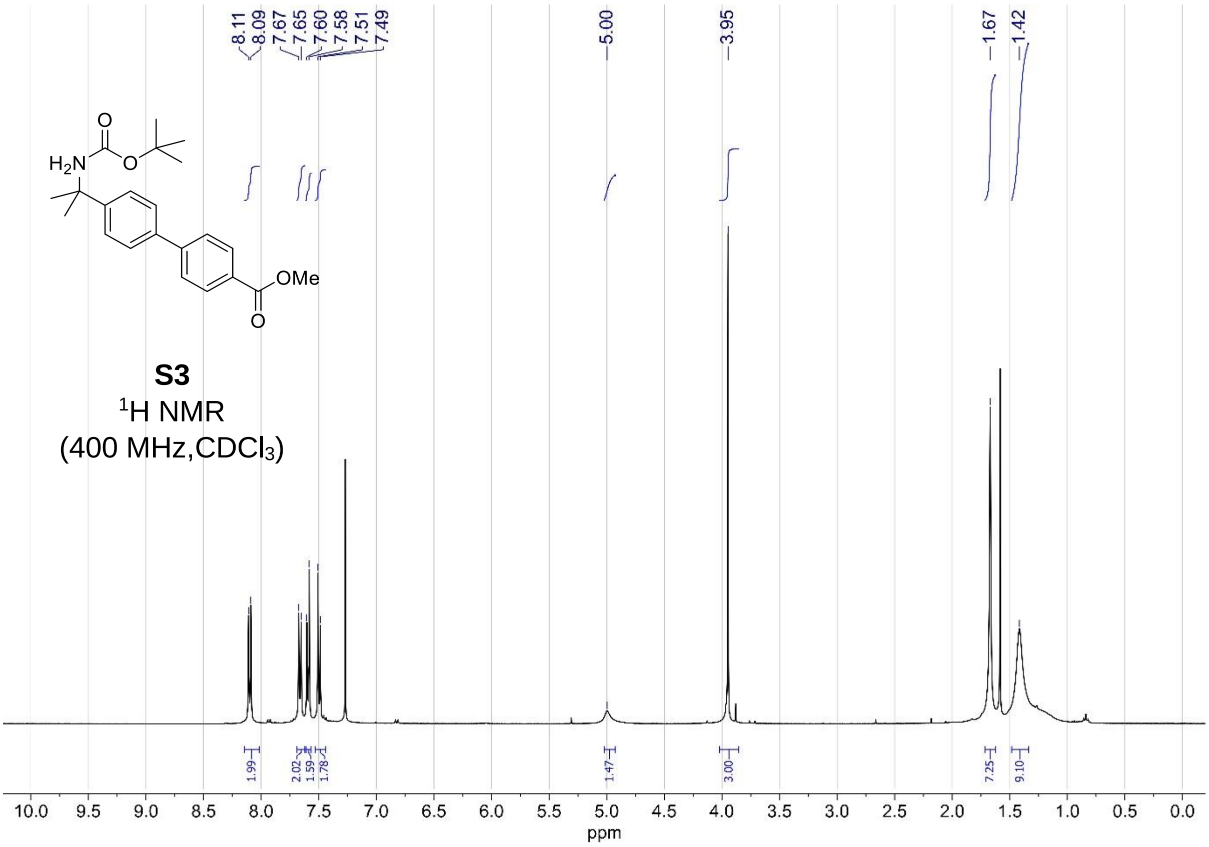
8.11
8.09
7.67
7.65
7.60
7.58
7.51
7.49
5.00
3.95
1.67
1.42
H_2N
O
O
OMe
O
S3
^{1}H NMR
(400 MHz,$CDCl_3$)
1.99
2.02
1.59
1.78
1.47
3.00
7.25
9.10
ppm

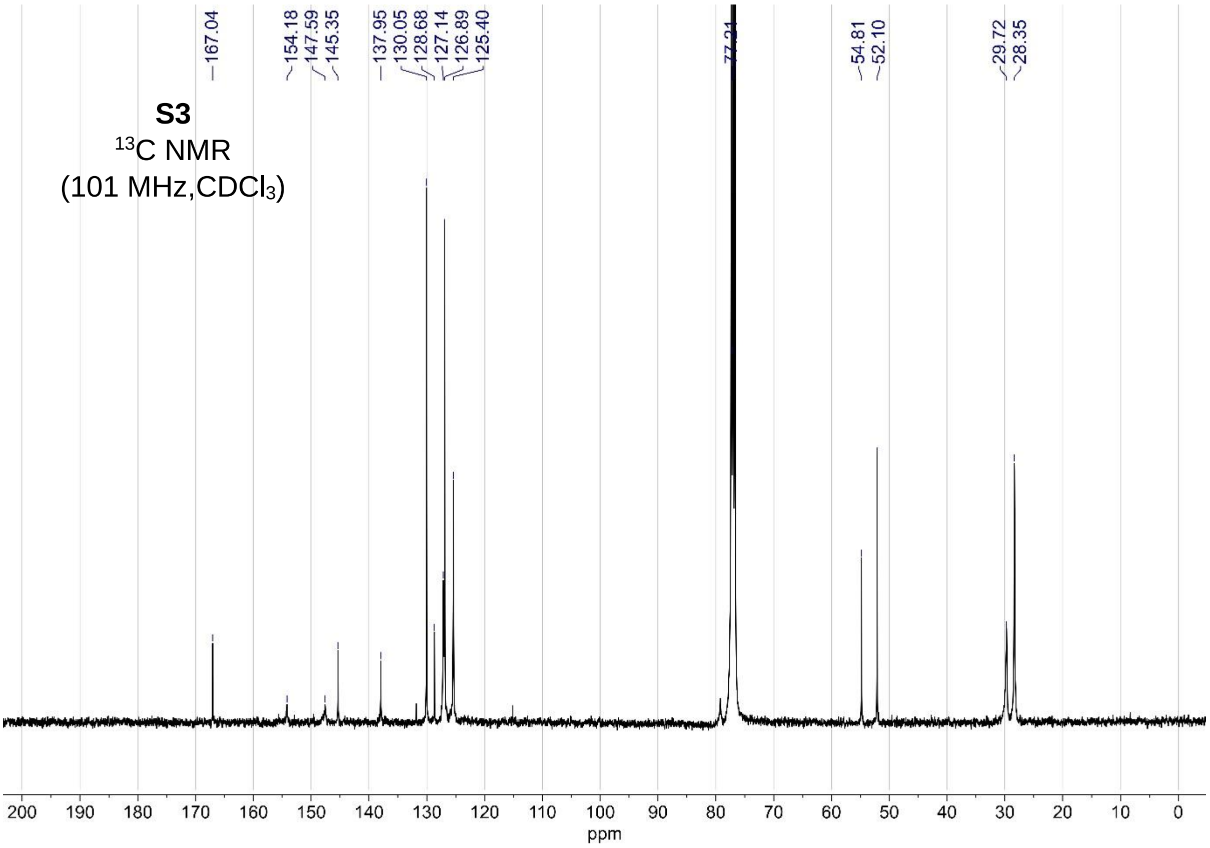
167.04
154.18
147.59
145.35
137.95
130.05
128.68
127.14
126.89
125.40
77.21
54.81
52.10
29.72
28.35
S3
^{13}C NMR
(101 MHz,$CDCl_3$)
ppm

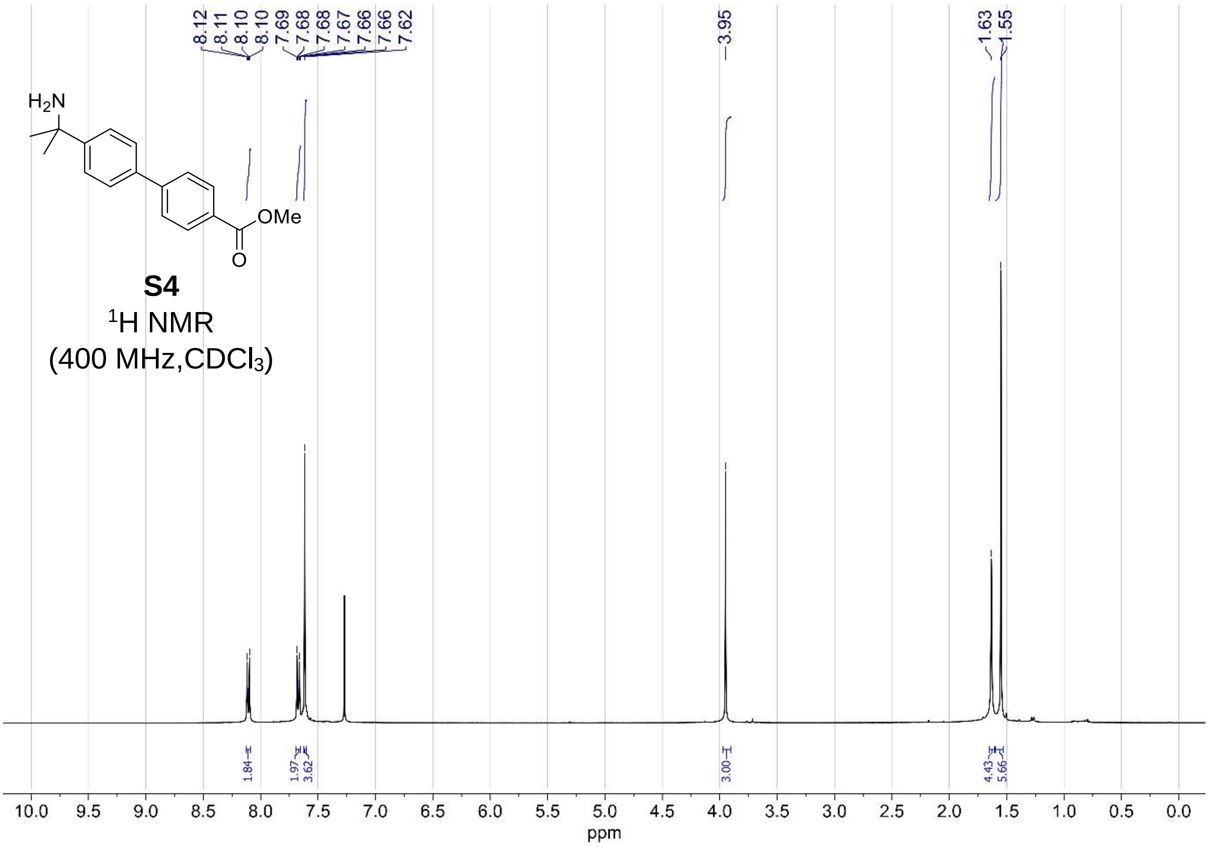
8.12
8.11
8.10
8.10
7.69
7.68
7.68
7.67
7.66
7.66
7.62
3.95
1.63
1.55
H2N
OMe
O
S4
1H NMR
(400 MHz,CDCl3)
1.84
1.97
3.62
3.00
4.43
5.66
10.0 9.5 9.0 8.5 8.0 7.5 7.0 6.5 6.0 5.5 5.0 4.5 4.0 3.5 3.0 2.5 2.0 1.5 1.0 0.5 0.0
ppm

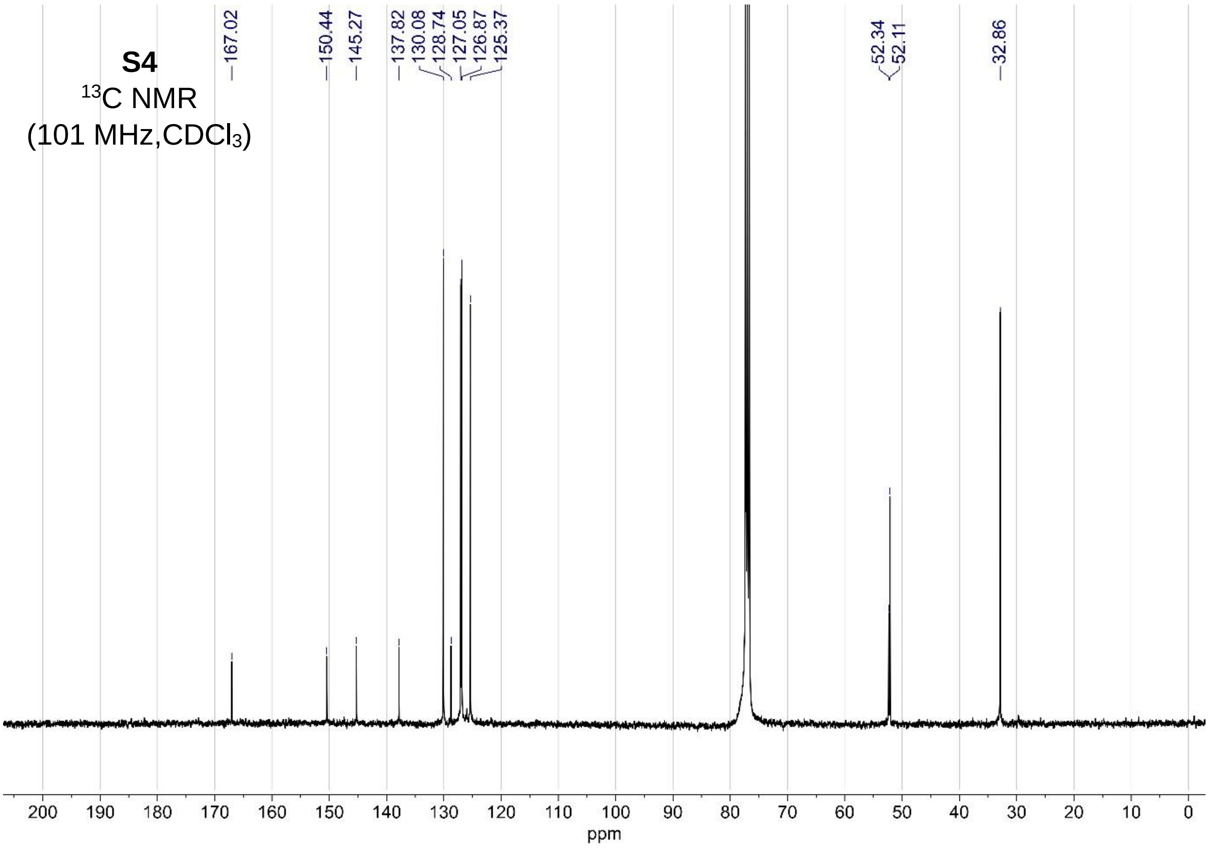
S4
13C NMR
(101 MHz,CDCl3)
167.02
150.44
145.27
137.82
130.08
128.74
127.05
126.87
125.37
52.34
52.11
32.86
200 190 180 170 160 150 140 130 120 110 100 90 80 70 60 50 40 30 20 10 0
ppm

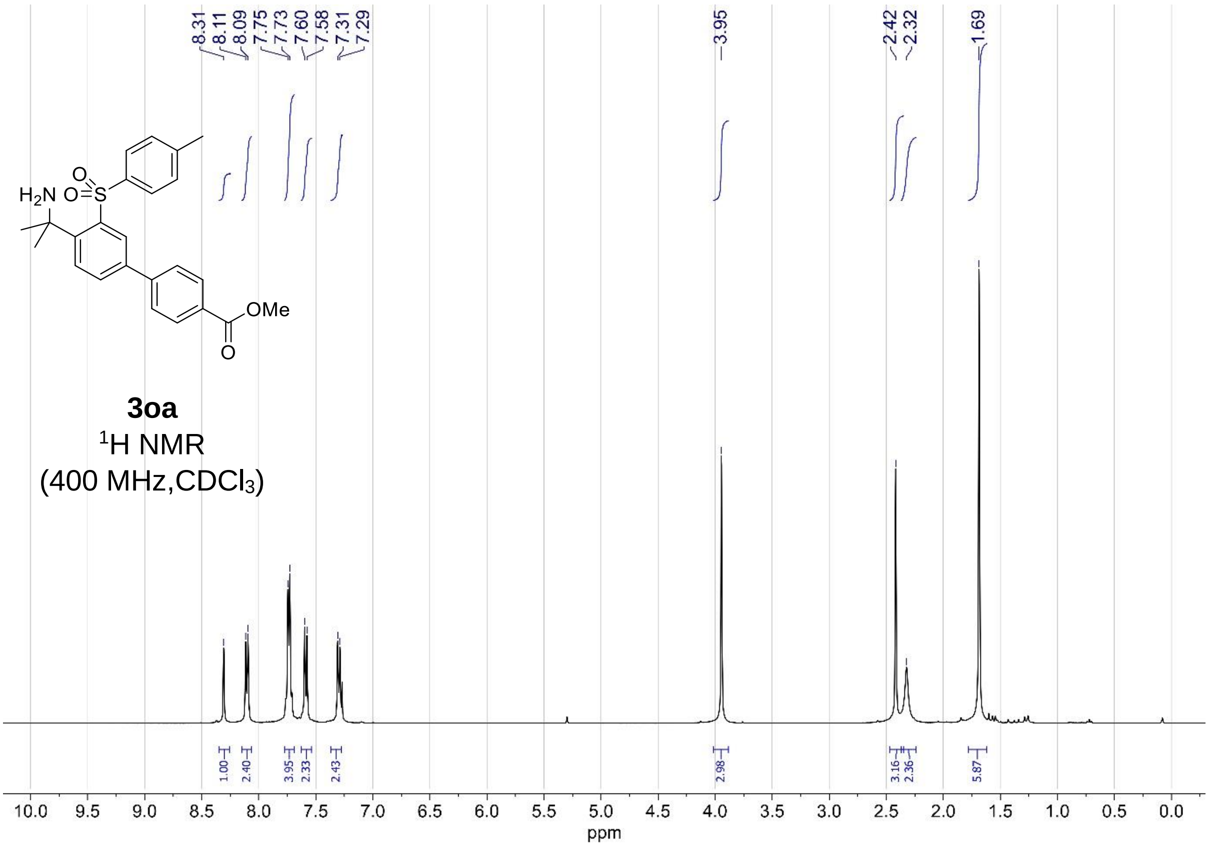
8.31
8.11
8.09
7.75
7.73
7.60
7.58
7.31
7.29
3.95
2.42
2.32
1.69
H2N
O
O=S
OMe
O
3oa
1H NMR
(400 MHz,CDCl3)
1.00
2.40
3.95
2.33
2.43
2.98
3.16
2.36
5.87
10.0 9.5 9.0 8.5 8.0 7.5 7.0 6.5 6.0 5.5 5.0 4.5 4.0 3.5 3.0 2.5 2.0 1.5 1.0 0.5 0.0
ppm

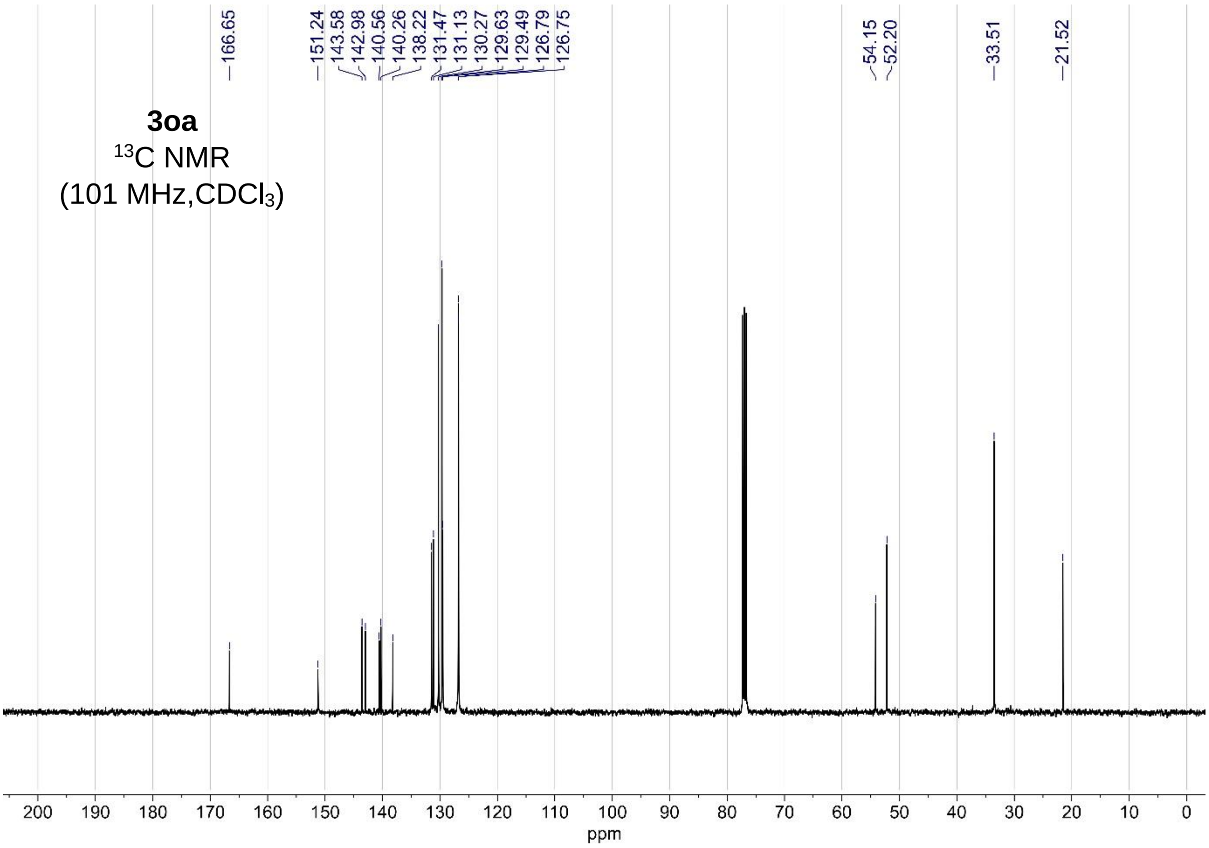
166.65
151.24
143.58
142.98
140.56
140.26
138.22
131.47
131.13
130.27
129.63
129.49
126.79
126.75
54.15
52.20
33.51
21.52
3oa
13C NMR
(101 MHz,CDCl3)
200 190 180 170 160 150 140 130 120 110 100 90 80 70 60 50 40 30 20 10 0
ppm

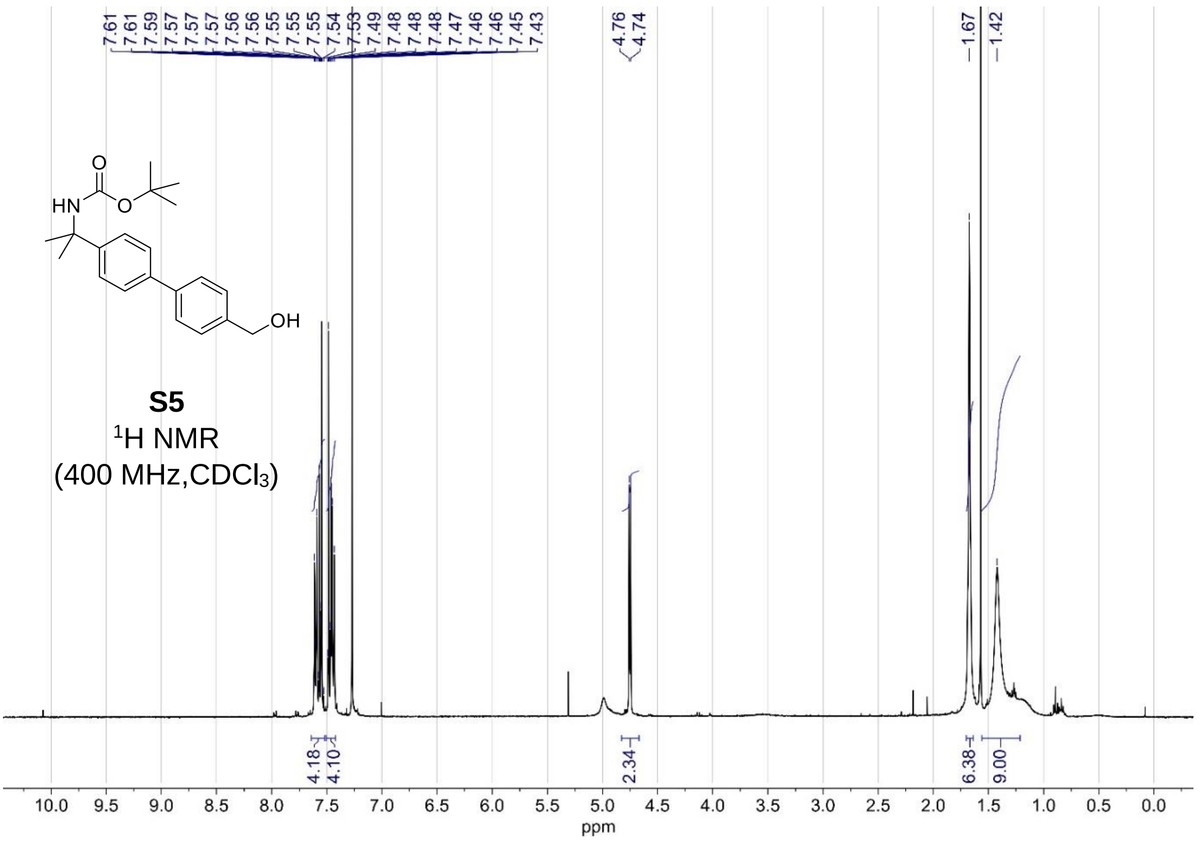
7.61 7.61 7.59 7.57 7.57 7.57 7.56 7.56 7.55 7.55 7.55 7.54 7.53 7.49 7.48 7.48 7.48 7.47 7.46 7.46 7.45 7.43
4.76 4.74
1.67
1.42
HN
O
O
OH
S5
^{1}H NMR
(400 MHz,CDCl$_3$)
4.18
4.10
2.34
6.38
9.00
10.0 9.5 9.0 8.5 8.0 7.5 7.0 6.5 6.0 5.5 5.0 4.5 4.0 3.5 3.0 2.5 2.0 1.5 1.0 0.5 0.0
ppm

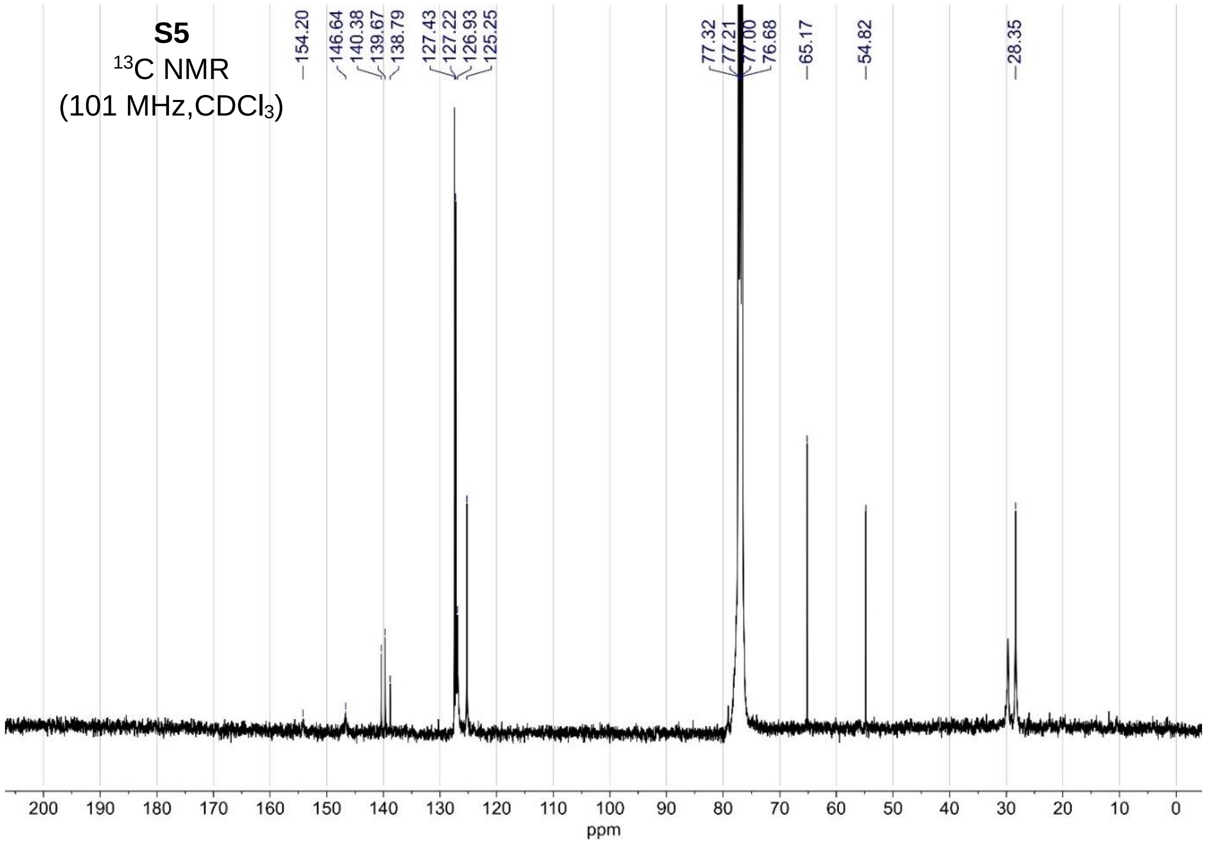
S5
^{13}C NMR
(101 MHz,CDCl$_3$)
154.20
146.64 140.38 139.67 138.79
127.43 127.22 126.93 125.25
77.32 77.21 77.00 76.68
65.17
54.82
28.35
200 190 180 170 160 150 140 130 120 110 100 90 80 70 60 50 40 30 20 10 0
ppm

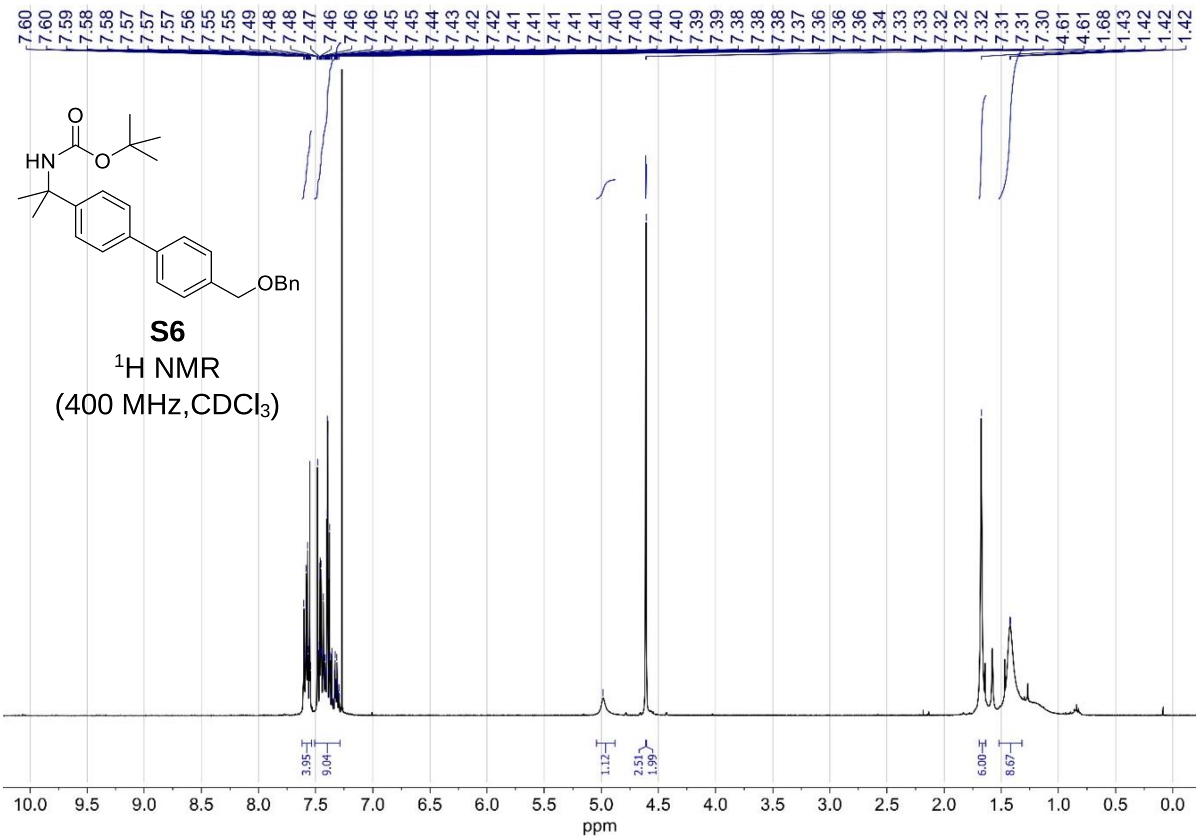
7.60 7.60 7.59 7.58 7.58 7.57 7.57 7.57 7.56 7.55 7.55 7.49 7.48 7.48 7.47 7.46 7.46 7.46 7.45 7.45 7.44 7.43 7.42 7.42 7.42 7.41 7.41 7.41 7.41 7.41 7.40 7.40 7.40 7.40 7.39 7.39 7.38 7.38 7.38 7.37 7.36 7.36 7.36 7.34 7.33 7.32 7.32 7.32 7.31 7.31 7.30 4.61 4.61 1.68 1.43 1.42 1.42 1.42
HN
O
O
OBn
S6
^{1}H NMR
(400 MHz,CDCl$_3$)
3.95
9.04
1.12
2.51
1.99
6.00
8.67
10.0 9.5 9.0 8.5 8.0 7.5 7.0 6.5 6.0 5.5 5.0 4.5 4.0 3.5 3.0 2.5 2.0 1.5 1.0 0.5 0.0
ppm

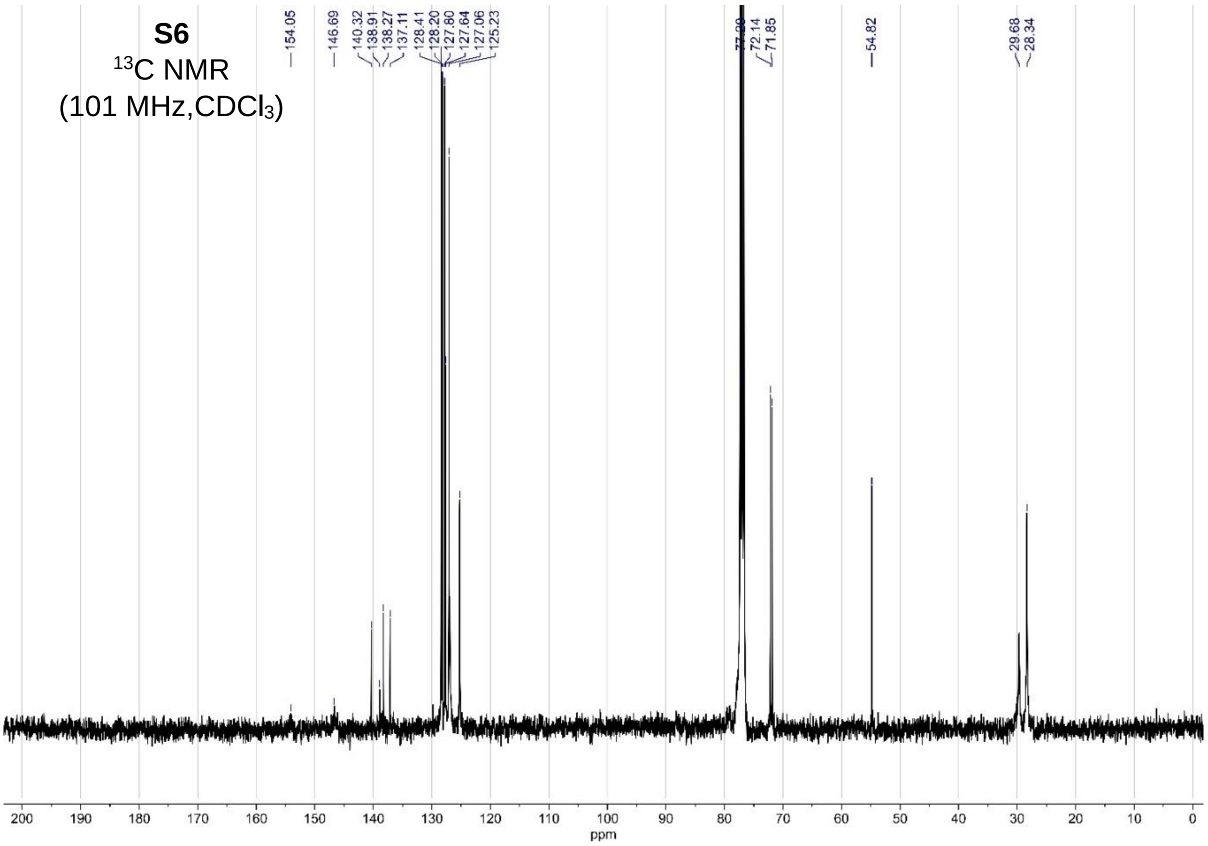
S6
^{13}C NMR
(101 MHz,CDCl$_3$)
154.05
146.69
140.32 138.91 138.27 137.11
128.41 128.20 127.80 127.64 127.06 125.23
77.20 72.14 71.85
54.82
29.68 28.34
200 190 180 170 160 150 140 130 120 110 100 90 80 70 60 50 40 30 20 10 0
ppm

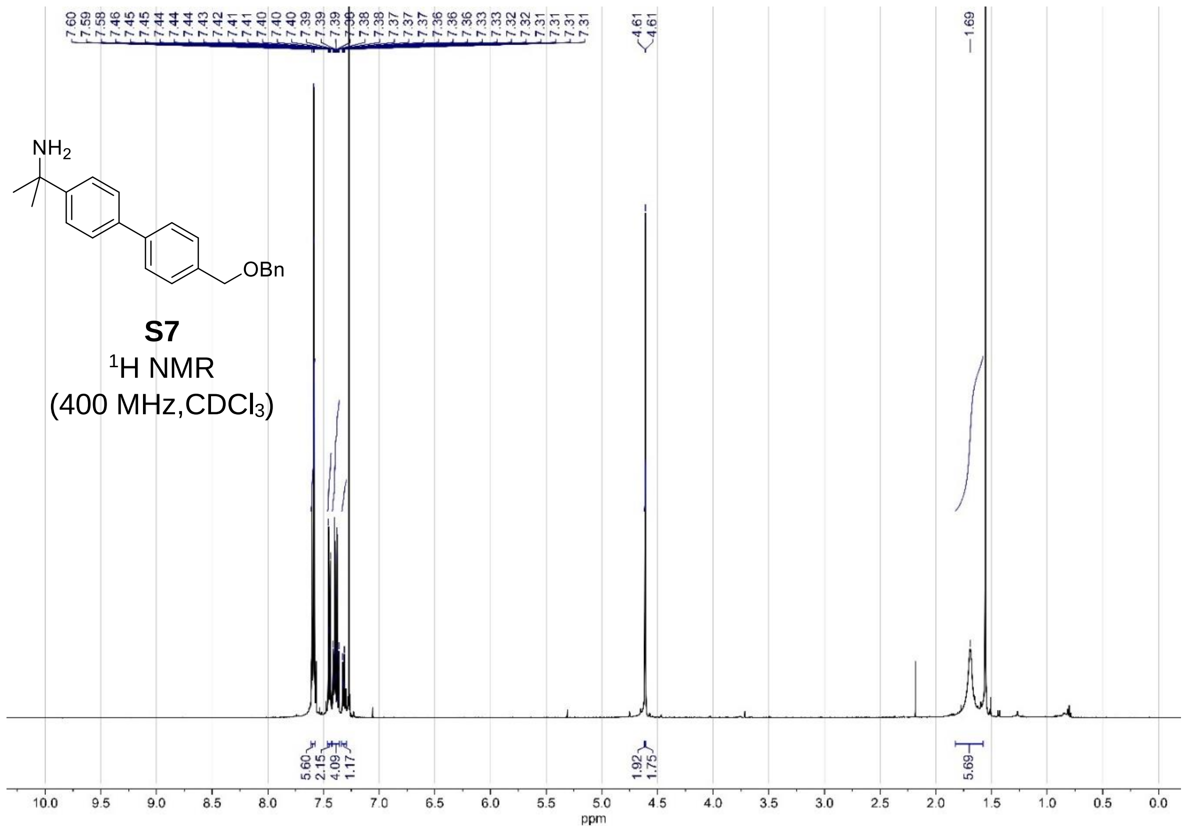
NH2
OBn
S7
1H NMR
(400 MHz,CDCl3)
7.60 7.59 7.58 7.46 7.45 7.45 7.44 7.44 7.44 7.43 7.42 7.41 7.41 7.40 7.40 7.40 7.39 7.39 7.39 7.39 7.38 7.38 7.38 7.37 7.37 7.37 7.36 7.36 7.36 7.33 7.33 7.32 7.32 7.31 7.31 7.31 7.31
4.61 4.61
1.69
5.60 2.15 4.09 1.17
1.92 1.75
5.69
10.0 9.5 9.0 8.5 8.0 7.5 7.0 6.5 6.0 5.5 5.0 4.5 4.0 3.5 3.0 2.5 2.0 1.5 1.0 0.5 0.0
ppm

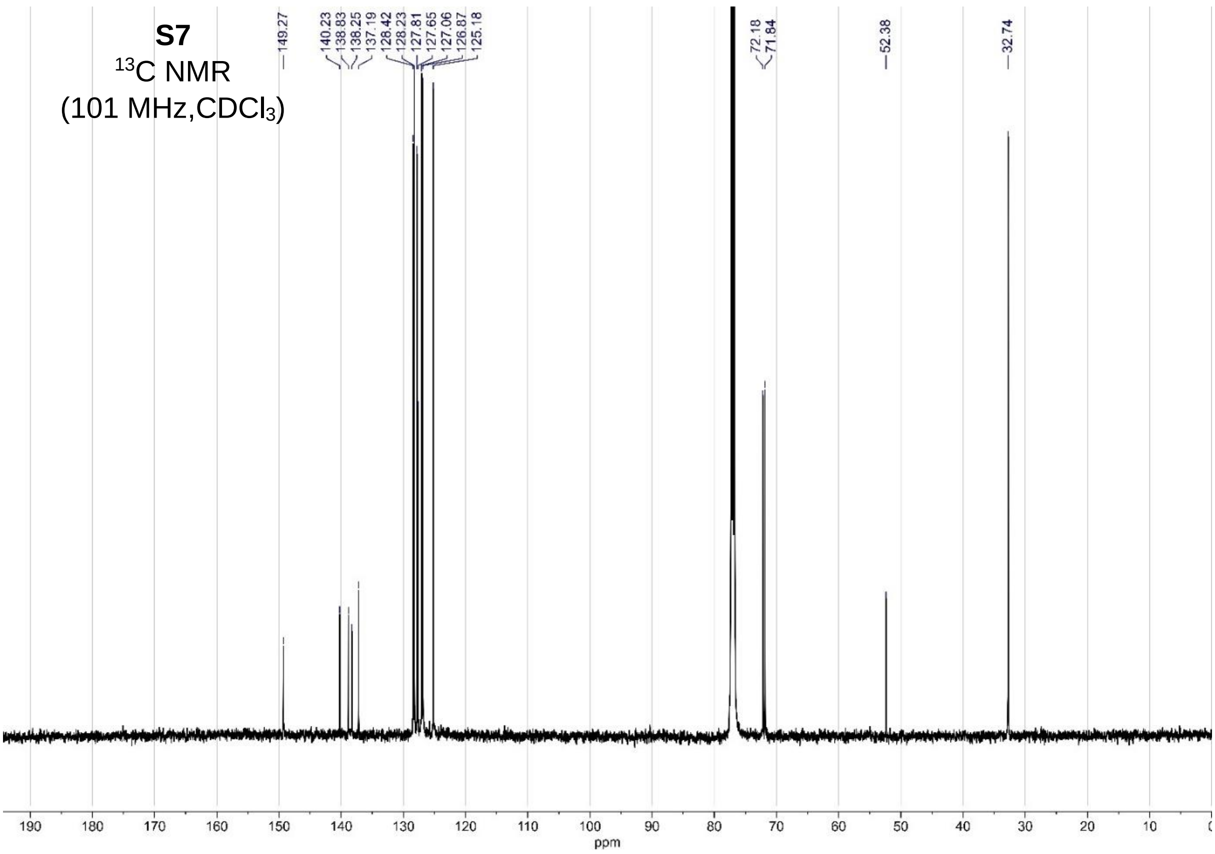
S7
13C NMR
(101 MHz,CDCl3)
149.27
140.23 138.83 138.25 137.19 128.42 128.23 127.81 127.65 127.06 126.87 125.18
72.18 71.84
52.38
32.74
190 180 170 160 150 140 130 120 110 100 90 80 70 60 50 40 30 20 10 0
ppm

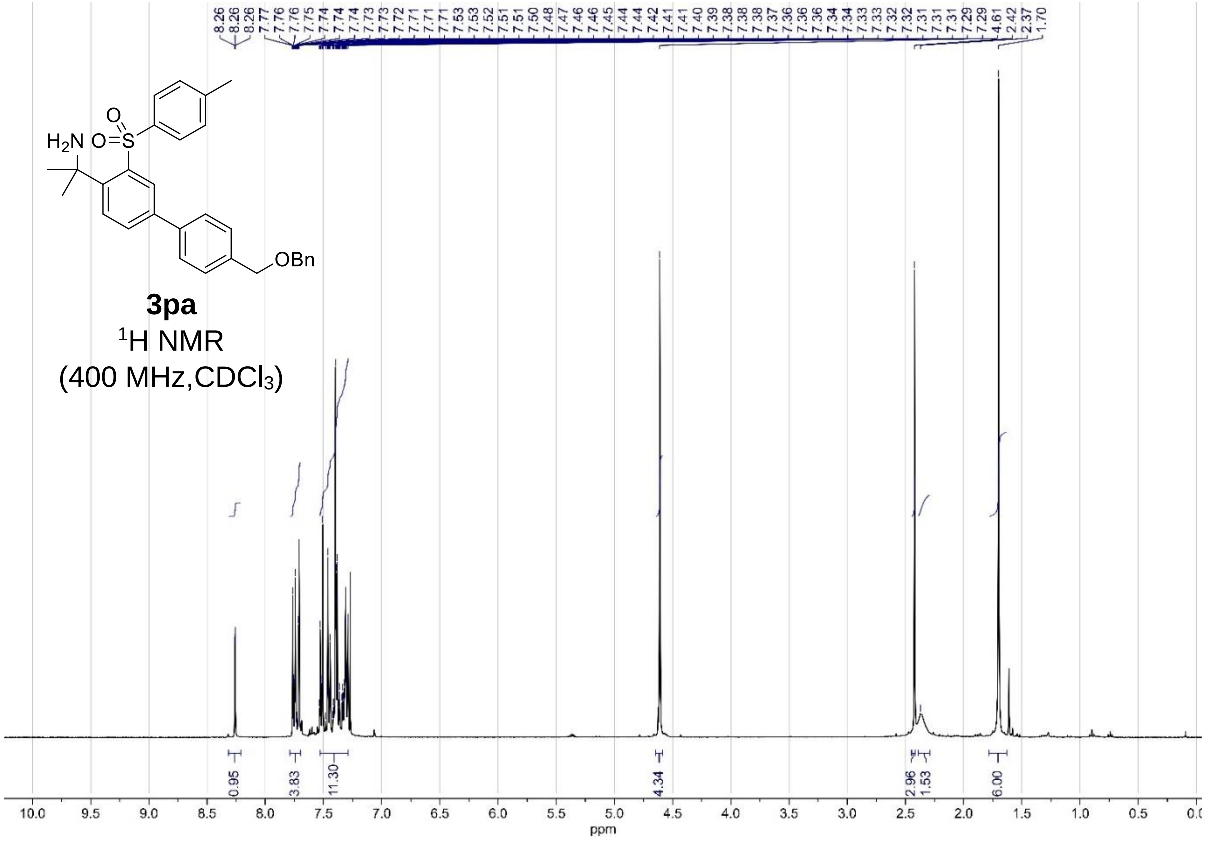
3pa
^{1}H NMR
(400 MHz,CDCl$_3$)
ppm

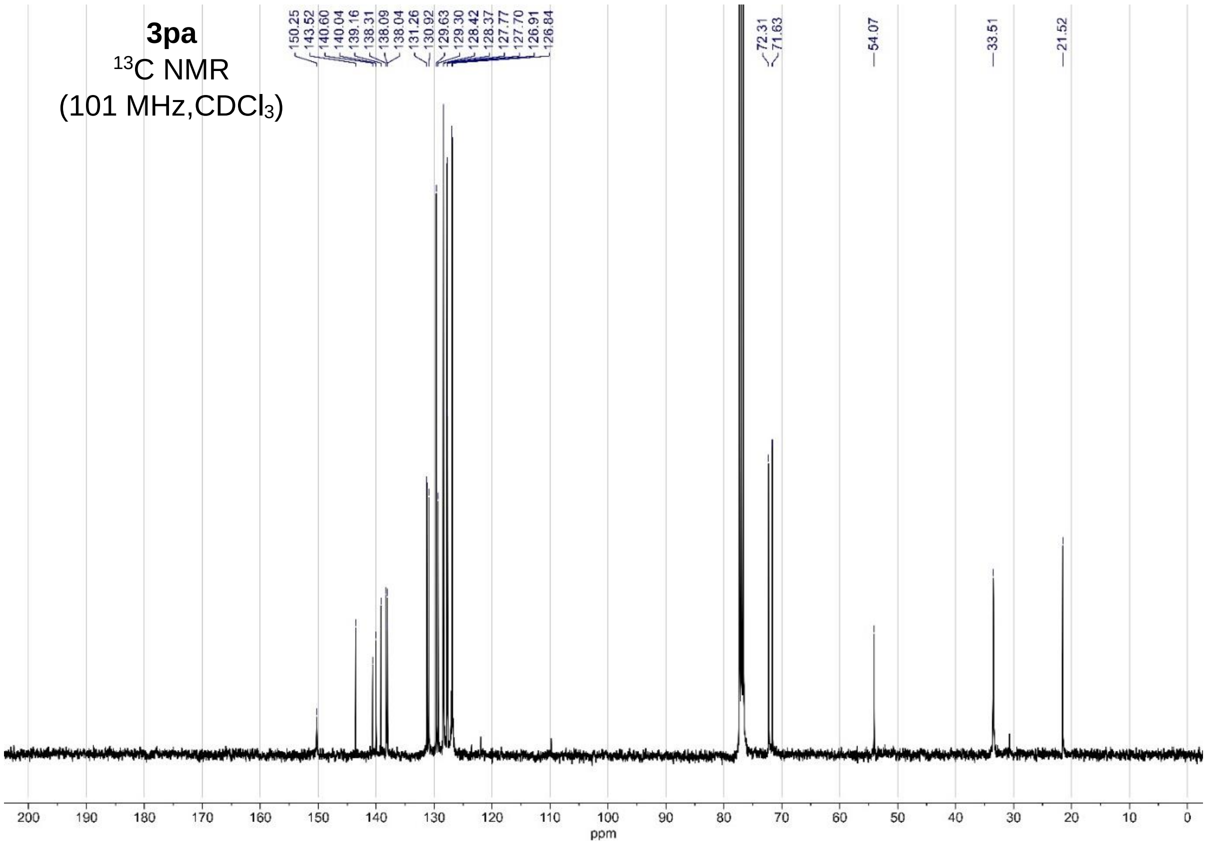
3pa
^{13}C NMR
(101 MHz,CDCl$_3$)
150.25
143.52
140.60
140.04
139.16
138.31
138.09
138.04
131.26
130.92
129.63
129.30
128.42
128.37
127.77
127.70
126.91
126.84
72.31
71.63
54.07
33.51
21.52
ppm

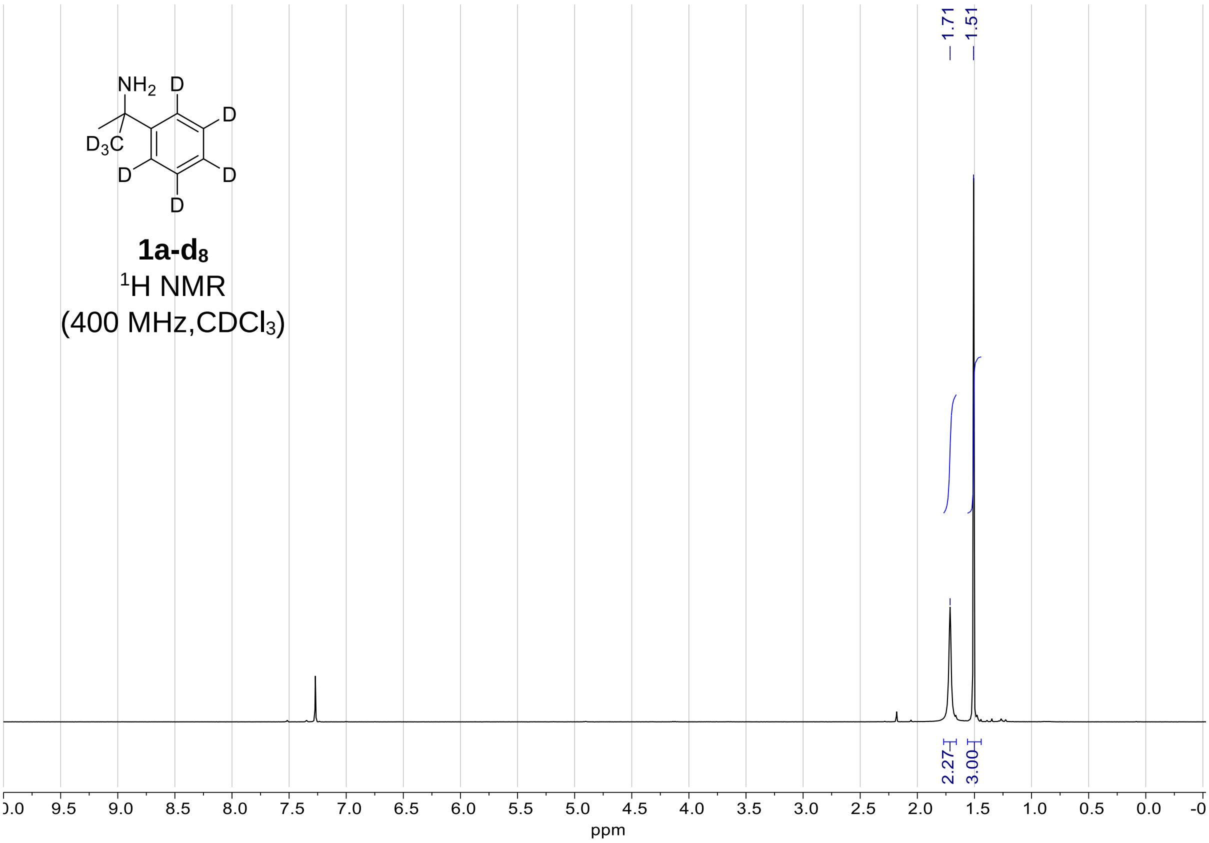
NH2 D D D3C D D D
1a-d8
1H NMR
(400 MHz,CDCl3)
1.71
1.51
2.27
3.00
ppm

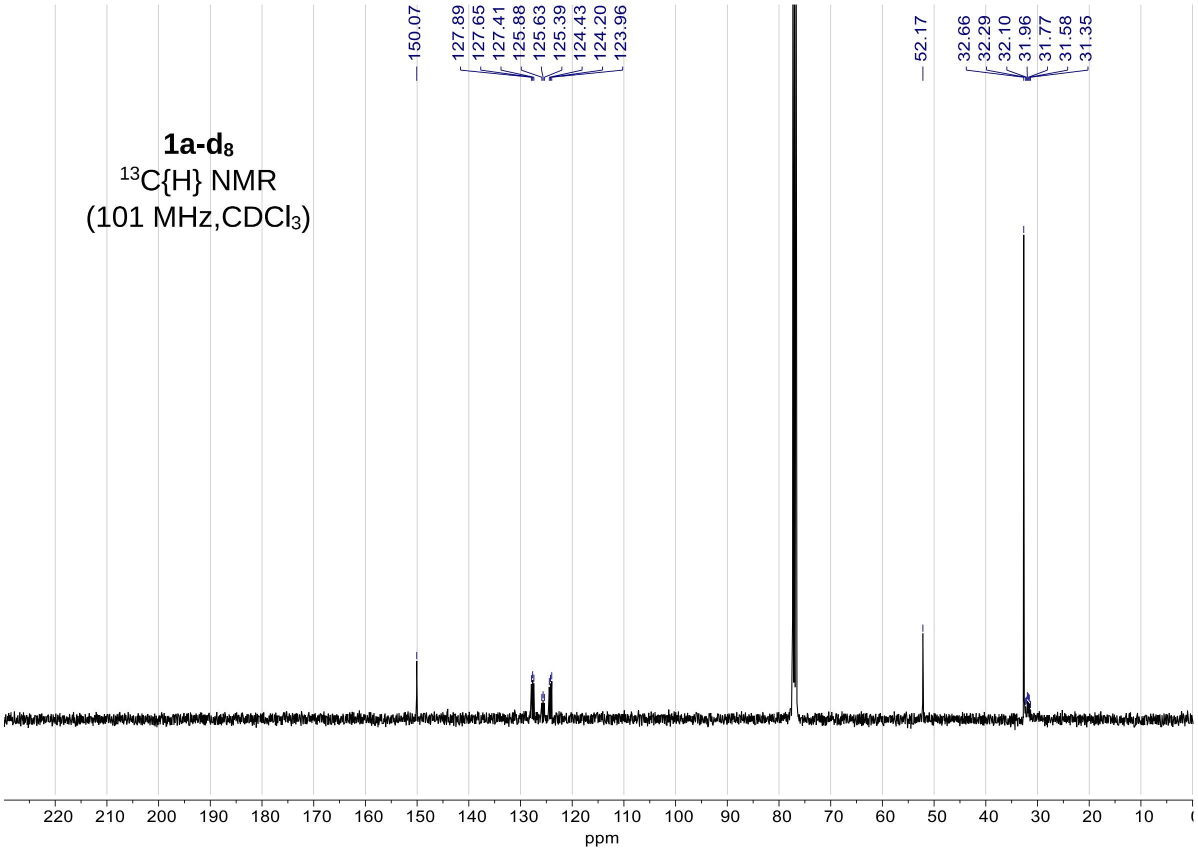
1a-d8
13C{H} NMR
(101 MHz,CDCl3)
150.07
127.89 127.65 127.41 125.88 125.63 125.39 124.43 124.20 123.96
52.17
32.66 32.29 32.10 31.96 31.77 31.58 31.35
ppm

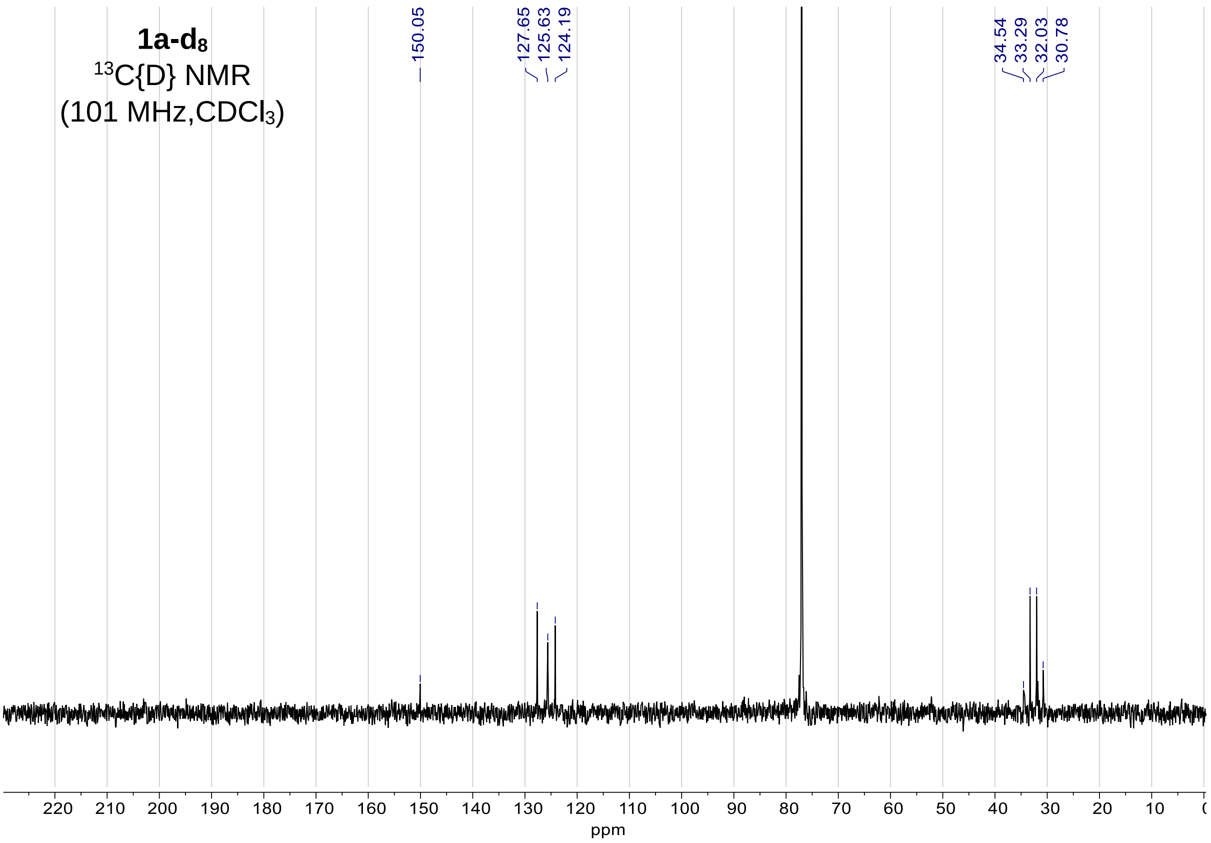
1a-d8
13C{D} NMR
(101 MHz,CDCl3)
150.05
127.65
125.63
124.19
34.54
33.29
32.03
30.78
220 210 200 190 180 170 160 150 140 130 120 110 100 90 80 70 60 50 40 30 20 10
ppm

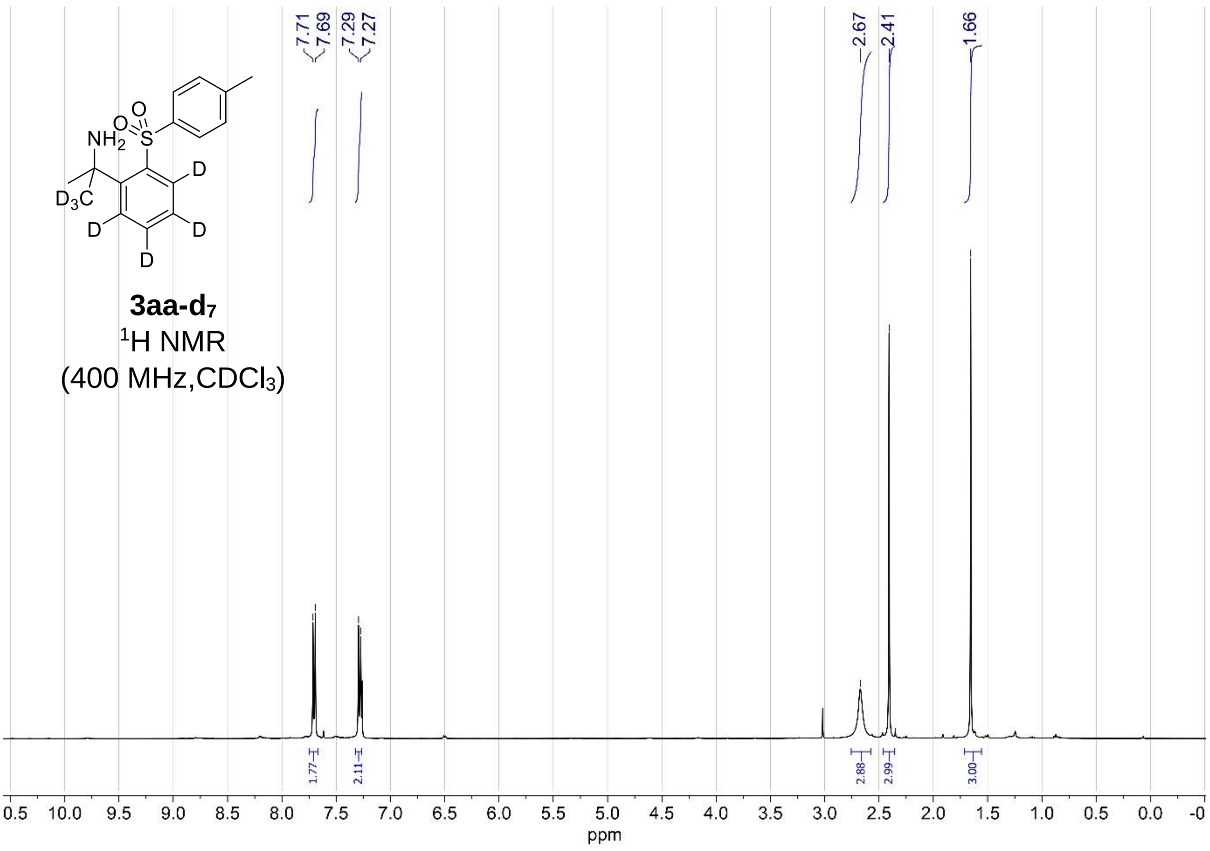
7.71
7.69
7.29
7.27
2.67
2.41
1.66
NH2
O
O
S
D
D3C
D
D
D
3aa-d7
1H NMR
(400 MHz,CDCl3)
1.77
2.11
2.88
2.99
3.00
ppm

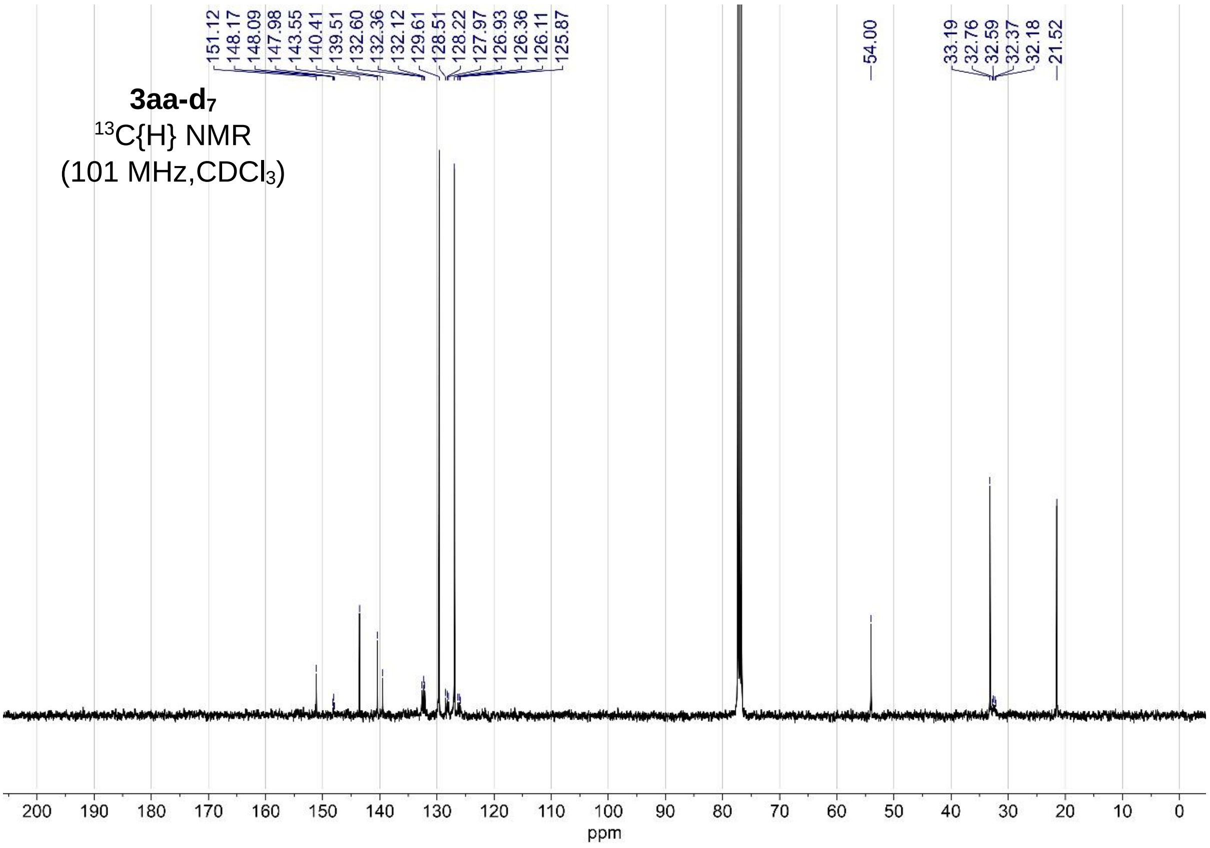
151.12
148.17
148.09
147.98
143.55
140.41
139.51
132.60
132.36
132.12
129.61
128.51
128.22
127.97
126.93
126.36
126.11
125.87
54.00
33.19
32.76
32.59
32.37
32.18
21.52
3aa-d7
13C{H} NMR
(101 MHz,CDCl3)
ppm

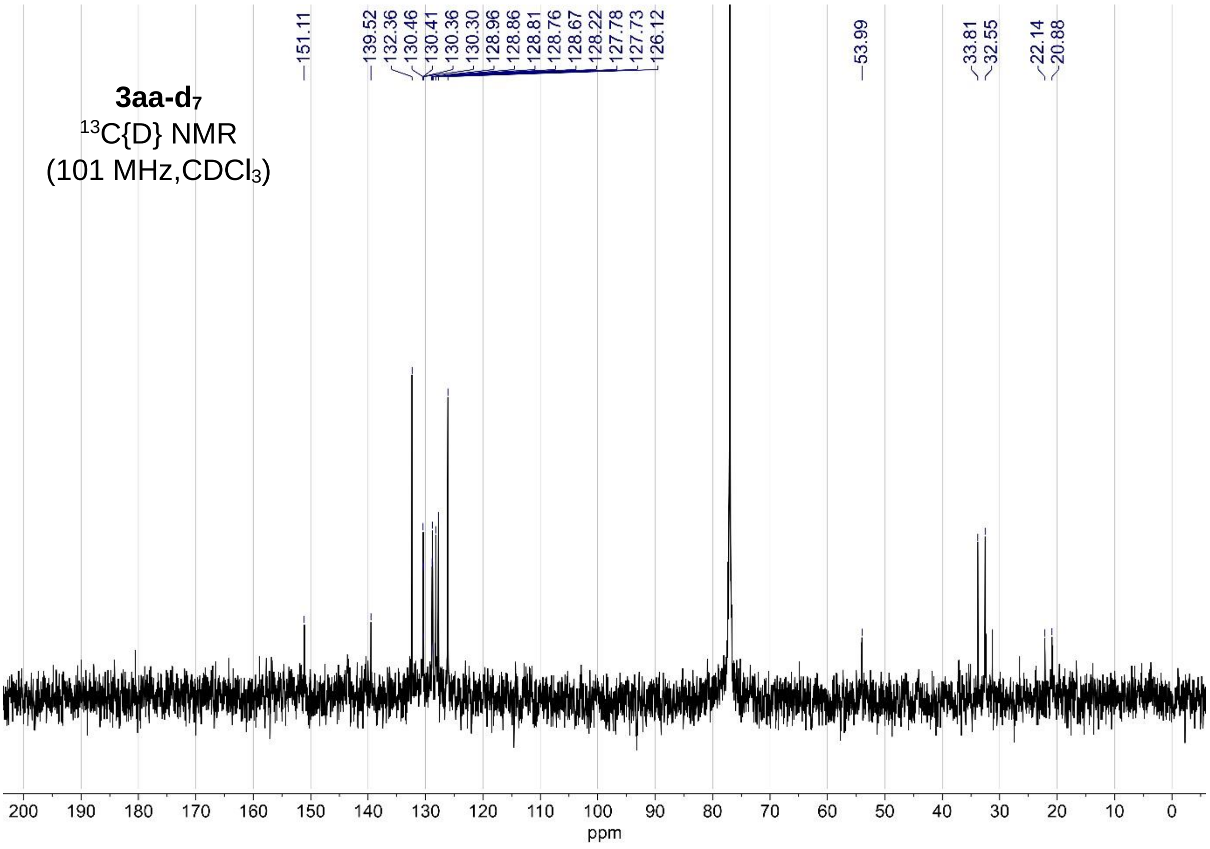
3aa-d7
13C{D} NMR
(101 MHz,CDCl3)
151.11
139.52
132.36
130.46
130.41
130.36
130.30
128.96
128.86
128.81
128.76
128.67
128.22
127.78
127.73
126.12
53.99
33.81
32.55
22.14
20.88
200 190 180 170 160 150 140 130 120 110 100 90 80 70 60 50 40 30 20 10 0
ppm

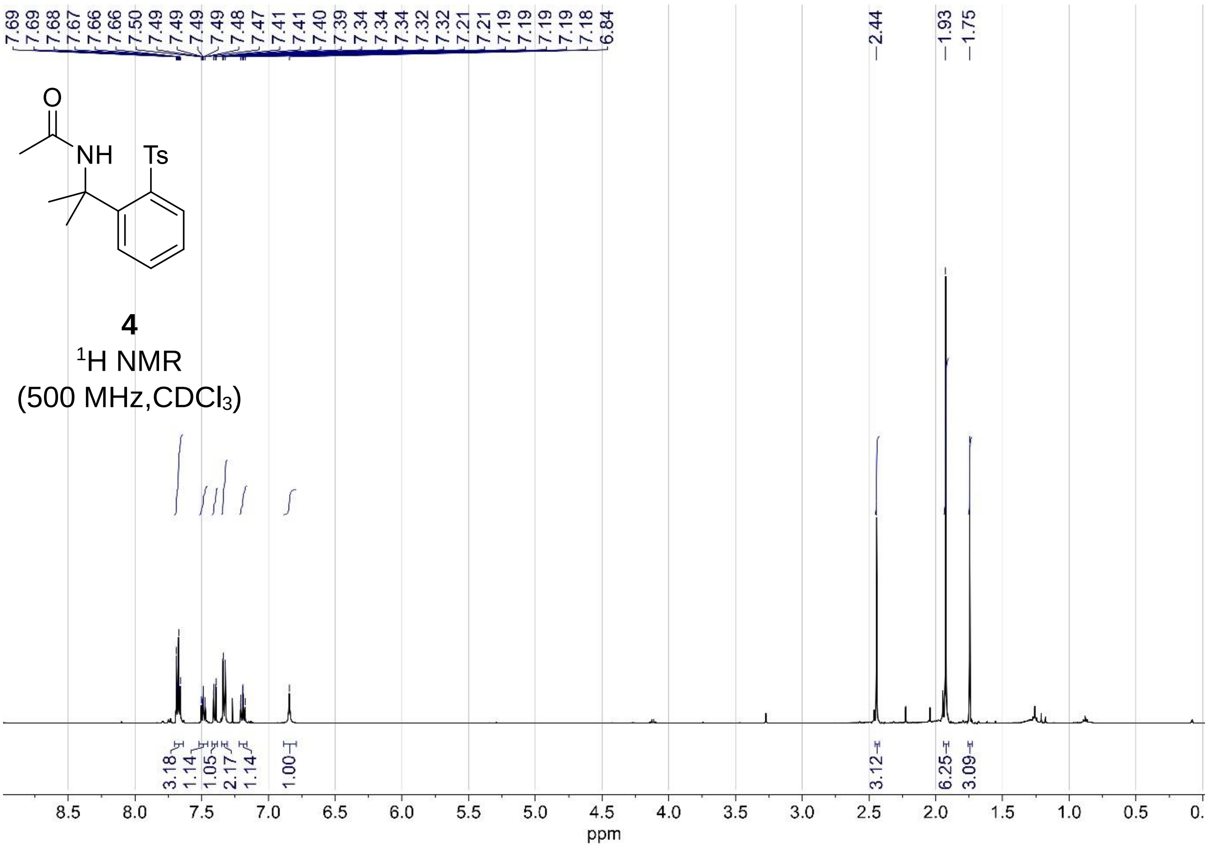
7.69 7.69 7.68 7.67 7.66 7.66 7.50 7.49 7.49 7.49 7.49 7.48 7.47 7.41 7.41 7.40 7.39 7.34 7.34 7.34 7.32 7.32 7.21 7.21 7.19 7.19 7.19 7.19 7.18 6.84
2.44
1.93
1.75
O
NH
Ts
4
^{1}H NMR
(500 MHz,CDCl$_3$)
3.18 1.14 1.05 2.17 1.14 1.00
3.12
6.25 3.09
8.5 8.0 7.5 7.0 6.5 6.0 5.5 5.0 4.5 4.0 3.5 3.0 2.5 2.0 1.5 1.0 0.5 0.
ppm

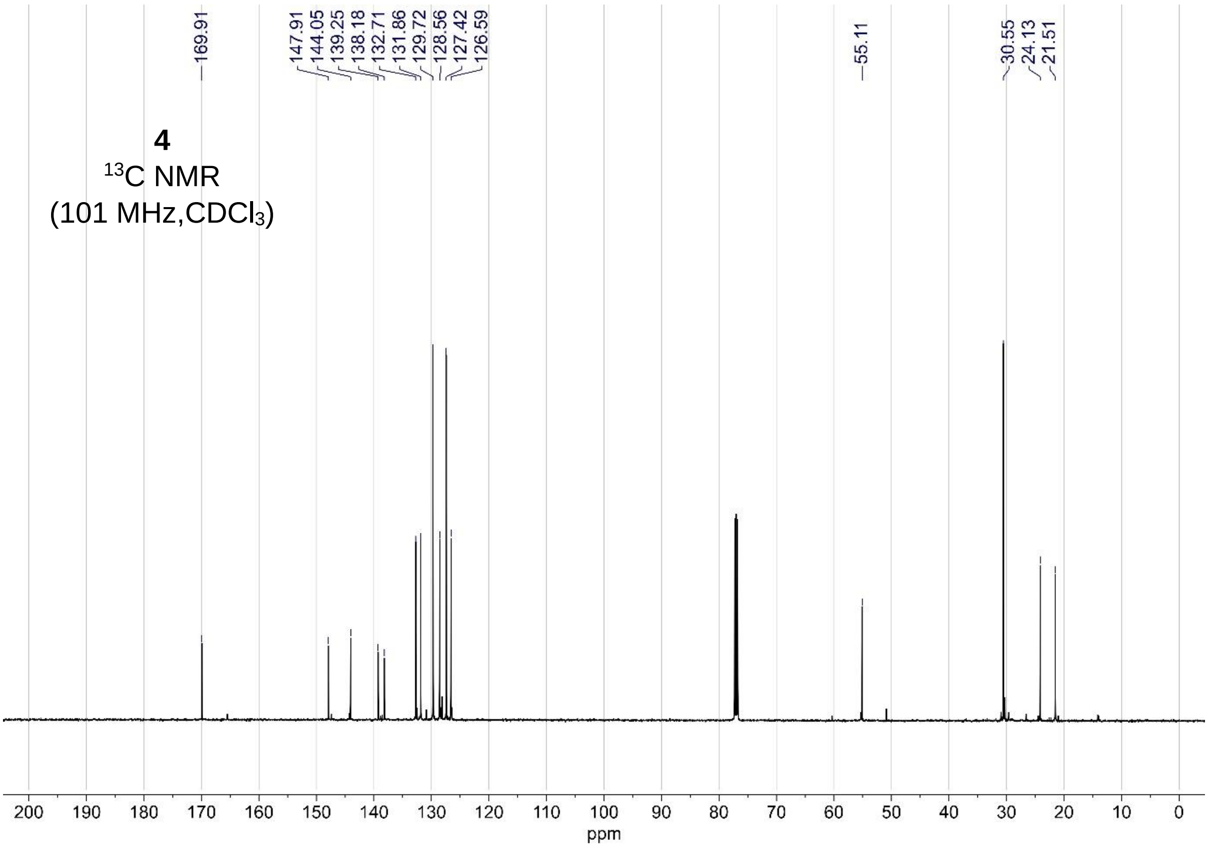
169.91
147.91 144.05 139.25 138.18 132.71 131.86 129.72 128.56 127.42 126.59
55.11
30.55 24.13 21.51
4
^{13}C NMR
(101 MHz,CDCl$_3$)
200 190 180 170 160 150 140 130 120 110 100 90 80 70 60 50 40 30 20 10 0
ppm

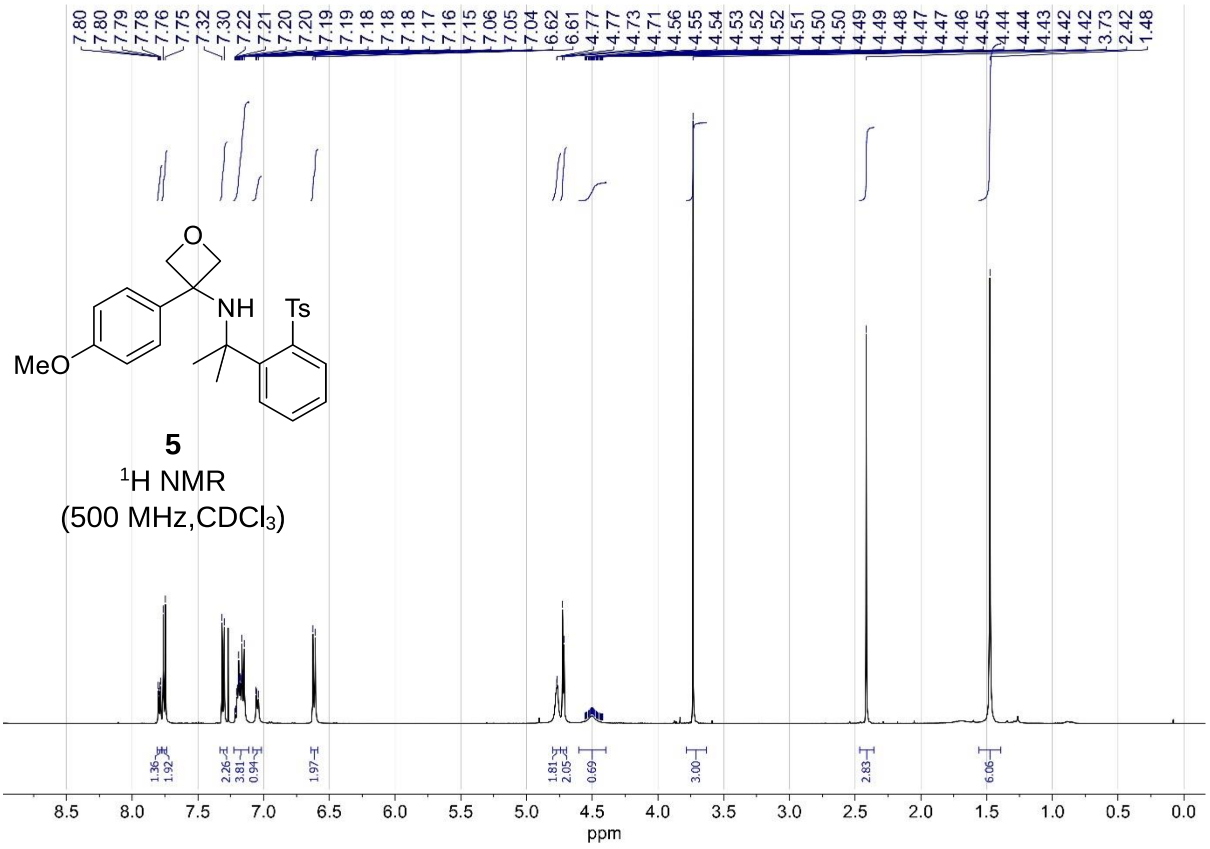
7.80 7.80 7.79 7.78 7.76 7.75 7.32 7.30 7.22 7.21 7.20 7.20 7.19 7.19 7.18 7.18 7.18 7.17 7.16 7.15 7.06 7.05 7.04 6.62 6.61 4.77 4.77 4.73 4.71 4.56 4.55 4.54 4.53 4.52 4.52 4.51 4.50 4.50 4.49 4.49 4.48 4.47 4.47 4.46 4.45 4.44 4.44 4.43 4.42 4.42 3.73 2.42 1.48
O
NH
Ts
MeO
5
1H NMR
(500 MHz,CDCl3)
1.36 1.92 2.26 3.81 0.94 1.97 1.81 2.05 0.69 3.00 2.83 6.06
8.5 8.0 7.5 7.0 6.5 6.0 5.5 5.0 4.5 4.0 3.5 3.0 2.5 2.0 1.5 1.0 0.5 0.0
ppm

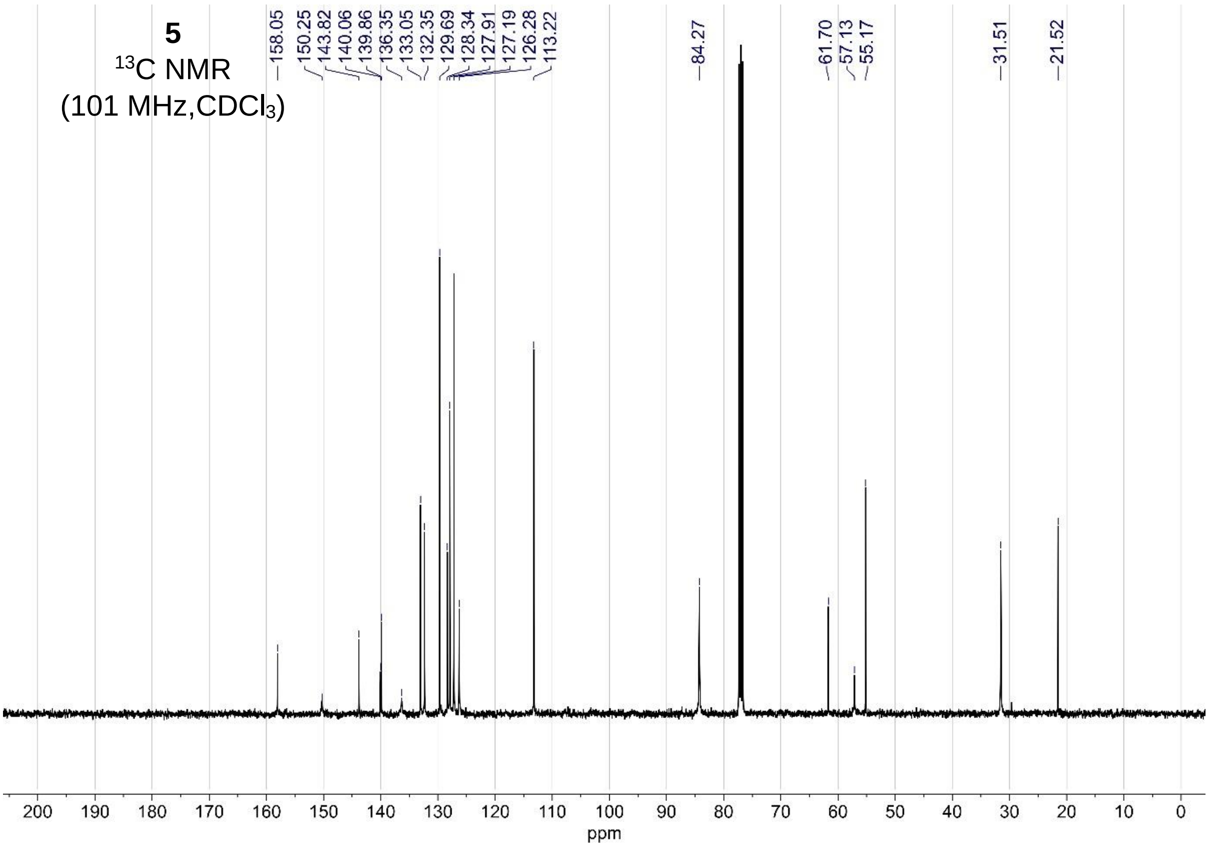
5
13C NMR
(101 MHz,CDCl3)
158.05 150.25 143.82 140.06 139.86 136.35 133.05 132.35 129.69 128.34 127.91 127.19 126.28 113.22
84.27
61.70 57.13 55.17
31.51
21.52
200 190 180 170 160 150 140 130 120 110 100 90 80 70 60 50 40 30 20 10 0
ppm

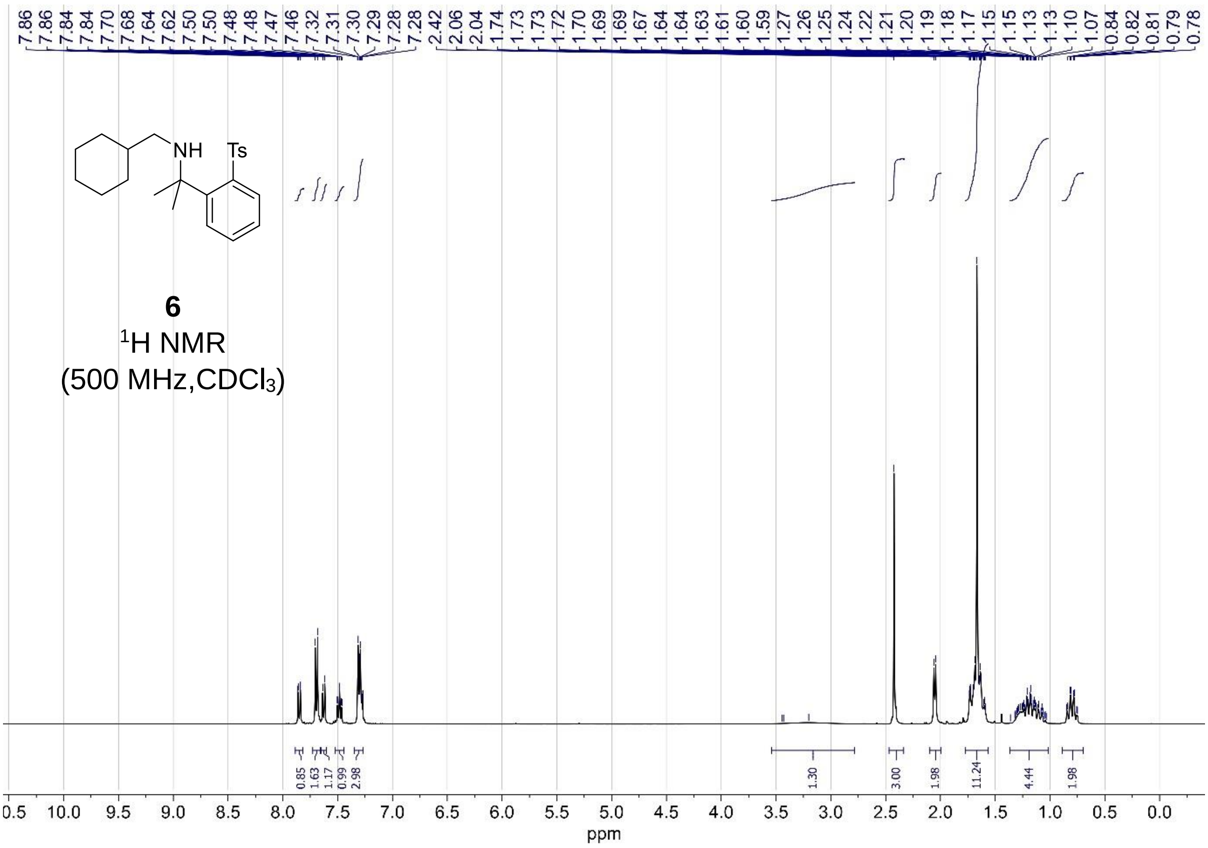
7.86 7.86 7.84 7.84 7.70 7.68 7.64 7.62 7.50 7.50 7.48 7.48 7.47 7.46 7.32 7.31 7.30 7.29 7.28 7.28 2.42 2.06 2.04 1.74 1.73 1.73 1.72 1.70 1.69 1.69 1.67 1.64 1.64 1.63 1.61 1.60 1.59 1.27 1.26 1.25 1.24 1.22 1.21 1.20 1.19 1.18 1.17 1.15 1.15 1.13 1.13 1.10 1.07 0.84 0.82 0.81 0.79 0.78
NH
Ts
6
^{1}H NMR
(500 MHz,CDCl$_3$)
0.85 1.63 1.17 0.99 2.98 1.30 3.00 1.98 11.24 4.44 1.98
0.5 10.0 9.5 9.0 8.5 8.0 7.5 7.0 6.5 6.0 5.5 5.0 4.5 4.0 3.5 3.0 2.5 2.0 1.5 1.0 0.5 0.0
ppm

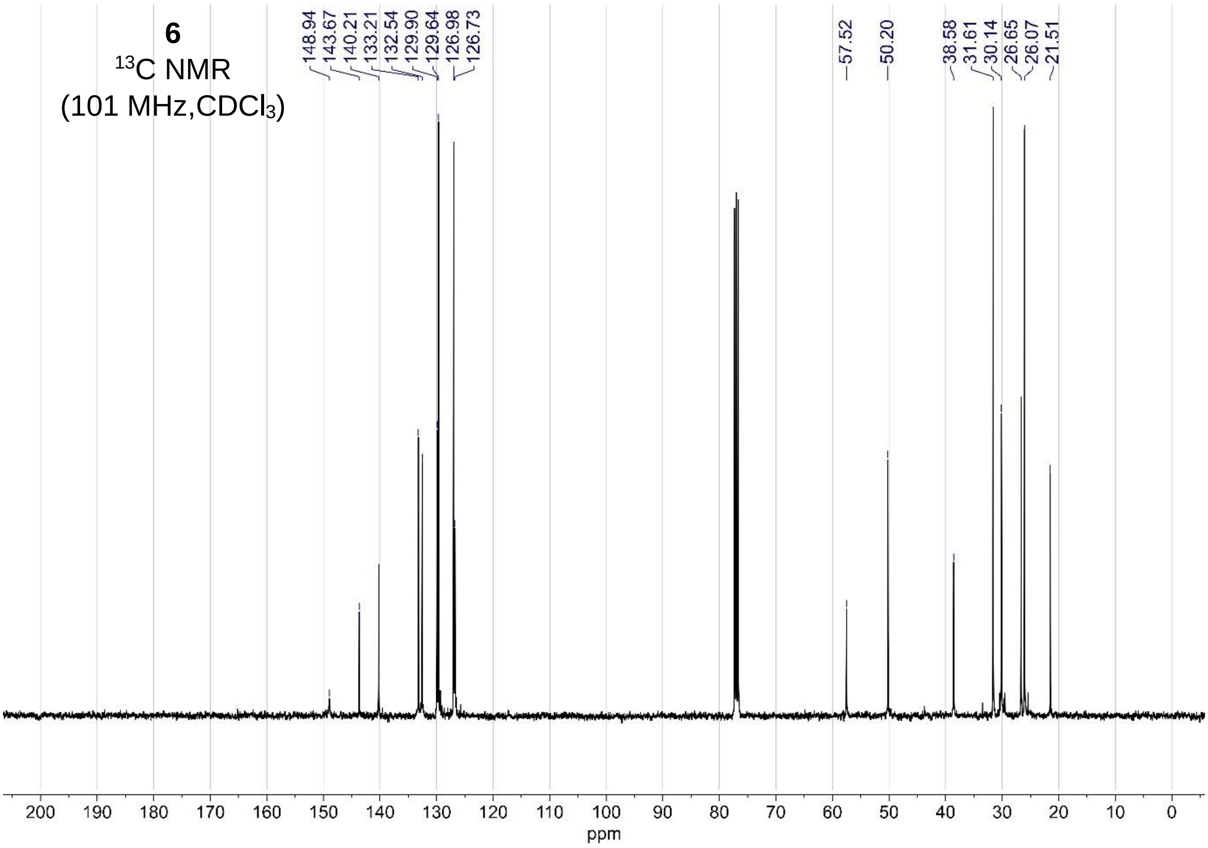
6
^{13}C NMR
(101 MHz,CDCl$_3$)
148.94 143.67 140.21 133.21 132.54 129.90 129.64 126.98 126.73
57.52
50.20
38.58 31.61 30.14 26.65 26.07 21.51
200 190 180 170 160 150 140 130 120 110 100 90 80 70 60 50 40 30 20 10 0
ppm

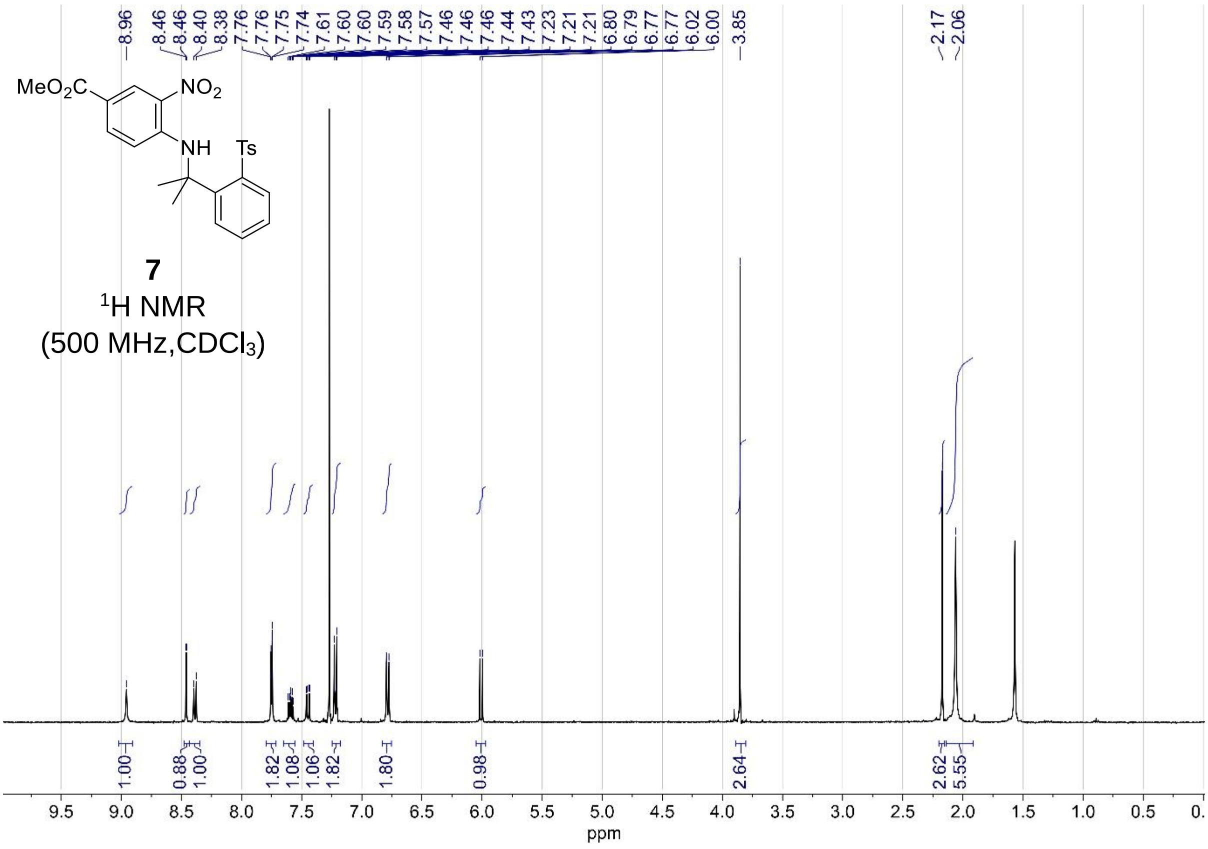
MeO2C
NO2
NH
Ts
7
1H NMR
(500 MHz,CDCl3)
8.96
8.46
8.46
8.40
8.38
7.76
7.76
7.75
7.74
7.61
7.60
7.60
7.59
7.58
7.57
7.46
7.46
7.46
7.44
7.43
7.23
7.21
7.21
6.80
6.79
6.77
6.77
6.02
6.00
3.85
2.17
2.06
1.00
0.88
1.00
1.82
1.08
1.06
1.82
1.80
0.98
2.64
2.62
5.55
ppm

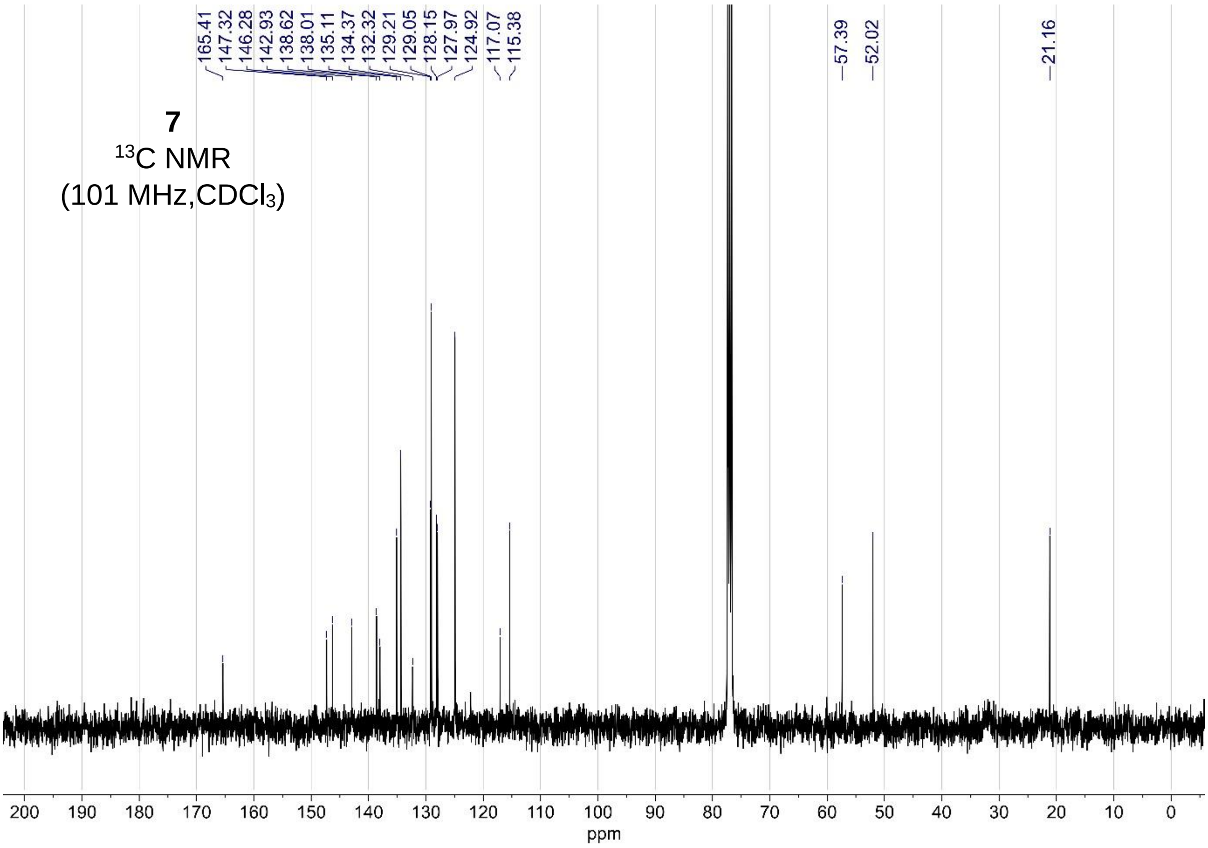
7
13C NMR
(101 MHz,CDCl3)
165.41
147.32
146.28
142.93
138.62
138.01
135.11
134.37
132.32
129.21
129.05
128.15
127.97
124.92
117.07
115.38
57.39
52.02
21.16
ppm

Energies, Lowest vibration and coordinates of calculations

Cu(OAc)$_2$

SCF (wB97x) = -654.333640
E(SCF)+ZPE(0 K)= -654.230202
H(298 K)= -654.218165
G(298 K)= -654.270988
Lowest Frequency = 16.6284cm^{-1}

C	2.596322	-1.566855	-1.604177
O	1.777463	-1.693756	-2.567155
O	2.190404	-0.920218	-0.589227
Cu	0.444667	-0.661441	-1.509133
C	3.959473	-2.175696	-1.654227
H	4.632002	-1.664817	-0.965521
H	4.349220	-2.143057	-2.672301
H	3.876914	-3.224512	-1.352740
C	-1.710239	0.235670	-1.409954
O	-0.889201	0.369300	-0.450141
O	-1.301602	-0.405309	-2.427772
C	-3.099977	0.777929	-1.333917
H	-3.751019	-0.006742	-0.936029
H	-3.455917	1.048731	-2.328287
H	-3.138139	1.634979	-0.661527

TDG

SCF (wB97x) = -436.780886
E(SCF)+ZPE(0 K)= -436.676986
H(298 K)= -436.668848
G(298 K)= -436.708914
Lowest Frequency = 85.3669cm^{-1}

C	-2.188190	5.566127	-1.623005
C	-1.412766	6.791613	-1.888495
C	-1.558820	7.865662	-1.007114
C	-0.522585	6.974433	-2.971969
C	-0.852148	9.044645	-1.208873
H	-2.233319	7.767027	-0.160560
C	-0.006583	9.104433	-2.310938
H	-0.949686	9.889698	-0.538831
H	0.570909	10.000889	-2.518651
H	-2.824608	5.640135	-0.718431
N	0.156073	8.096412	-3.172373
O	-2.181832	4.548124	-2.278918
O	-0.333491	5.991407	-3.853124
H	0.301989	6.313280	-4.504131

H_2O

SCF (wB97x) = -76.438259
E(SCF)+ZPE(0 K)= -76.416618
H(298 K)= -76.412838
G(298 K)= -76.434904
Lowest Frequency = 1609.4468cm^{-1}

O	0.003253	1.319690	-0.948040
H	0.961339	1.353387	-0.948040
H	-0.284802	2.234074	-0.948040

HOAc

SCF (wB97x) = -229.081527
E(SCF)+ZPE(0 K)= -229.019309
H(298 K)= -229.013778
G(298 K)= -229.046716
Lowest Frequency = 53.9106cm^{-1}

O	1.561782	-1.136432	-2.011297
C	1.324924	-2.256462	-2.400146
O	0.150128	-2.858132	-2.166436
C	2.264745	-3.131200	-3.178049
H	2.456586	-4.051189	-2.619861
H	1.805852	-3.410295	-4.129914
H	3.199734	-2.602755	-3.355878
H	-0.407478	-2.250462	-1.664538

$MeSO_2^-$

SCF (wB97x) = -588.644031
E(SCF)+ZPE(0 K)= -588.599488
H(298 K)= -588.593911
G(298 K)= -588.626866
Lowest Frequency = 218.1447cm^{-1}

S	-2.444364	4.255675	-3.972156
O	-2.619308	5.770217	-3.848152
O	-3.785077	3.536662	-3.814513
C	-2.138999	4.068699	-5.762075
H	-2.988141	4.518338	-6.286686
H	-1.212148	4.584501	-6.024953
H	-2.060380	3.005976	-6.004164

Int-1

SCF (wB97x) = -405.455828
E(SCF)+ZPE(0 K)= -405.251830
H(298 K)= -405.241356
G(298 K)= -405.285989
Lowest Frequency = 54.4921cm^{-1}

C	-0.201239	0.113289	0.477651
C	0.981675	0.591294	1.034082
C	-0.990348	2.384595	0.101206
C	-1.174952	1.001941	0.015698
H	-0.373041	-0.956078	0.397982
H	1.739729	-0.098625	1.391765

N	-1.398508	4.248717	-1.408844
C	-2.036485	3.396696	-0.385140
C	1.180312	1.969135	1.129182
H	2.095712	2.360078	1.563257
C	0.203768	2.848931	0.671725
H	0.383062	3.915321	0.770841
C	-2.539835	4.219762	0.820062
H	-1.721365	4.770765	1.293598
H	-3.290875	4.945276	0.488248
H	-2.996510	3.571479	1.575389
C	-3.239032	2.727971	-1.057493
H	-2.930123	2.124451	-1.915630
H	-3.791215	2.095864	-0.356185
H	-3.927689	3.499693	-1.417222
H	-2.083440	0.595091	-0.414184
H	-0.616152	4.761080	-1.016265
H	-2.063183	4.947632	-1.726364

Int-2

SCF (wB97x) = -765.797489
E(SCF)+ZPE(0 K)= -765.514270
H(298 K)= -765.497576
G(298 K)= -765.559197
Lowest Frequency = 17.7057cm^{-1}

C	-0.168359	0.195716	0.398389
C	1.014598	0.680869	0.949700
C	-1.004457	2.459540	0.114360
C	-1.168568	1.078496	-0.015141
H	-0.318380	-0.873799	0.284589
H	1.793407	-0.004953	1.268739
N	-1.432905	4.502567	-1.082694
C	-1.490324	5.722433	-0.743631
C	-2.097296	3.465111	-0.278126
C	1.189515	2.058783	1.085833
H	2.107373	2.452592	1.512211
C	0.189485	2.933936	0.672281
H	0.342959	4.003035	0.778169
C	-2.787623	3.947892	1.011548
H	-2.084876	4.433610	1.695826
H	-3.606757	4.641480	0.793417
H	-3.210537	3.083330	1.530312
C	-3.156188	2.853678	-1.208079
H	-2.694880	2.426123	-2.102468
H	-3.733476	2.077152	-0.698651
H	-3.852151	3.638096	-1.519298
H	-2.078329	0.671084	-0.442700
C	-0.874092	6.836367	-1.487879
C	-1.111974	8.133134	-1.029336
C	-0.047263	6.726566	-2.630265
C	-0.568398	9.237552	-1.678986
H	-1.735395	8.272550	-0.150138
C	0.221656	8.999509	-2.794255
H	-0.750653	10.247120	-1.331283
H	0.674451	9.818858	-3.345574
H	-2.021635	6.057238	0.156098
O	0.253613	5.524201	-3.136044
N	0.477348	7.771958	-3.256525
H	0.828804	5.682692	-3.894280

Int-3

SCF (wB97x) = -1420.187935
E(SCF)+ZPE(0 K)= -1419.798712
H(298 K)= -1419.769976
G(298 K)= -1419.861594
Lowest Frequency = 5.3172cm^{-1}

C	-2.802740	0.795945	-2.102643
C	-2.961933	1.944160	-2.875218
C	-1.850682	2.581730	-3.425345
C	-0.581288	2.058000	-3.191449
C	-0.427222	0.904171	-2.423774
C	-1.534038	0.258127	-1.867095
H	-3.957814	2.343125	-3.042649
H	-1.973086	3.479817	-4.022675
H	0.295855	2.546494	-3.604901
H	-3.683372	0.322935	-1.684108
Cu	0.198776	0.142419	1.190556
C	-1.374120	-1.074192	-1.122514
N	-0.061959	-1.073946	-0.408637
O	0.715936	2.417993	0.558823
C	-0.528087	2.463139	0.556736
O	-1.222720	1.427783	0.859611
C	-1.288460	3.708917	0.184358
H	-0.610214	4.550520	0.043274
H	-1.831917	3.517213	-0.745695
H	-2.023430	3.945127	0.957897
H	0.572118	0.517338	-2.253274
C	-2.441568	-1.305716	-0.040233
H	-3.424162	-1.463078	-0.490579
H	-2.187507	-2.209033	0.522030
H	-2.504457	-0.458409	0.643848
C	-1.509335	-2.193863	-2.172508
H	-1.392628	-3.185463	-1.722830
H	-2.514336	-2.133428	-2.596163
H	-0.805490	-2.082404	-3.002220
O	-0.042495	0.596428	3.228618
C	0.744043	0.575715	4.178334
O	1.965299	0.125618	4.086464
C	0.358068	1.066184	5.539447
H	0.397364	0.231947	6.245040
H	1.076863	1.817807	5.874338
H	-0.645431	1.487141	5.517835
C	0.879536	-1.848874	-0.812099
H	0.709002	-2.466547	-1.692697
C	2.198565	-2.031403	-0.238067
C	3.087845	-2.879929	-0.909845
C	2.636689	-1.444949	0.980927

C	4.349197	-3.120926	-0.390234
H	2.778145	-3.347430	-1.840889
C	4.671717	-2.502223	0.817749
H	5.059230	-3.768513	-0.889537
H	5.646099	-2.668051	1.271677
O	1.872751	-0.650117	1.669575
H	2.114475	-0.221399	3.152230
N	3.852914	-1.695896	1.488514

Int-4

SCF (wB97x) = -1420.165542
E(SCF)+ZPE(0 K)= -1419.777912
H(298 K)= -1419.748515
G(298 K)= -1419.842396
Lowest Frequency = 9.9625cm^{-1}

C	-0.131111	1.610556	-1.215102
C	-0.310471	3.000451	-1.280336
C	-0.973057	3.599764	-2.352410
C	-1.470239	2.802961	-3.384245
C	-1.300456	1.420496	-3.340847
C	-0.633022	0.822993	-2.265226
H	0.070241	3.620971	-0.472377
H	-1.104065	4.677919	-2.384155
H	-1.991377	3.255892	-4.222625
H	-1.835898	1.496887	0.032302
Cu	0.766105	0.580849	0.163366
C	-0.457780	-0.697288	-2.211318
N	0.325778	-0.999194	-0.983904
O	-2.622326	1.327603	0.589443
C	-2.275871	0.548560	1.610172
O	-1.138545	0.155421	1.777968
C	-3.434243	0.212571	2.502819
H	-3.912694	1.130734	2.852058
H	-4.176778	-0.352942	1.933199
H	-3.090498	-0.377845	3.350449
H	-1.697422	0.816682	-4.153480
C	-1.835323	-1.376710	-2.118505
H	-2.444058	-1.112815	-2.986901
H	-1.743278	-2.466761	-2.094723
H	-2.360735	-1.048863	-1.216842
C	0.318664	-1.186842	-3.445151
H	0.440526	-2.274118	-3.433671
H	-0.217506	-0.924482	-4.360353
H	1.308419	-0.722467	-3.478436
O	1.289478	2.240930	1.364878
C	1.853885	2.302525	2.459214
O	2.393172	1.271344	3.047371
C	1.963228	3.587755	3.223044
H	1.394125	3.498486	4.152601
H	3.006392	3.772380	3.489572
H	1.572182	4.412983	2.630314
C	0.614899	-2.211241	-0.692387
H	0.275728	-3.016817	-1.351410
C	1.371117	-2.669574	0.458813
C	1.531767	-4.050750	0.621323
C	1.972155	-1.807582	1.427905
C	2.245413	-4.553004	1.697773
H	1.088325	-4.725897	-0.106278
C	2.789000	-3.627050	2.588223
H	2.381951	-5.616806	1.848627
H	3.358237	-3.970411	3.449603
O	1.889332	-0.517806	1.350986
H	2.270006	0.447977	2.461520
N	2.666631	-2.308427	2.467137

Int-5

SCF (wB97x) = -1191.070022
E(SCF)+ZPE(0 K)= -1190.745219
H(298 K)= -1190.722306
G(298 K)= -1190.798459
Lowest Frequency = 23.4853cm^{-1}

C	1.937403	2.419008	-0.003546
Cu	0.699674	0.087881	-0.933769
C	2.172889	1.327158	-0.851355
C	3.399883	1.250123	-1.522586
H	3.592895	0.407722	-2.182606
C	4.376929	2.232905	-1.359794
H	5.323893	2.158666	-1.887966
C	4.131329	3.314937	-0.512467
H	4.883936	4.086384	-0.377055
C	2.916206	3.407052	0.162692
N	-0.200436	1.329359	0.344407
C	0.601760	2.520325	0.736963
C	-1.376601	1.165172	0.823859
H	-1.778238	1.908687	1.519502
H	2.741850	4.257070	0.818551
C	-2.281296	0.065964	0.545853
C	-1.982234	-1.023734	-0.327337
C	-3.531201	0.075024	1.177379
C	-4.437809	-0.947893	0.951962
H	-3.781850	0.894031	1.846676
C	-4.046834	-1.965257	0.080978
H	-5.410947	-0.965808	1.427043
H	-4.722994	-2.791327	-0.128871
O	-0.850814	-1.115136	-0.954234
C	0.836302	2.496232	2.256836
H	1.473317	3.333389	2.552650
H	-0.104612	2.583814	2.808729
H	1.330560	1.565211	2.548958
C	-0.142122	3.798000	0.313047
H	0.462392	4.678992	0.542301
H	-0.337709	3.782982	-0.763023
H	-1.093924	3.902310	0.842756
N	-2.870945	-2.011554	-0.538293
O	1.609986	-1.255035	-2.278577
C	1.226598	-2.313679	-2.783394

O	0.058256	-2.839658	-2.545348
C	2.086009	-3.095286	-3.730275
H	2.229239	-4.106582	-3.341532
H	1.576930	-3.179996	-4.693909
H	3.048522	-2.603420	-3.859460
H	-0.449890	-2.242343	-1.898221

Int-7

SCF (wB97x) = -1779.729765
E(SCF)+ZPE(0 K)= -1779.188200
H(298 K)= -1779.159627
G(298 K)= -1779.247711
Lowest Frequency = 17.5992cm^{-1}

C	1.814616	2.534531	-0.044642
Cu	0.149464	0.797037	-1.398674
C	1.739573	1.847840	-1.257417
C	2.791029	1.842740	-2.164356
H	2.747420	1.257382	-3.076638
C	3.929335	2.598630	-1.885696
H	4.754072	2.600465	-2.591385
C	4.002530	3.344711	-0.710541
H	4.881822	3.944180	-0.498157
C	2.956929	3.304498	0.204634
N	-0.208582	1.366261	0.401952
C	0.703424	2.401519	0.971555
C	-1.251238	1.006199	1.059987
H	-1.420731	1.455822	2.041157
H	3.035050	3.867059	1.130758
C	-2.256659	0.055935	0.653723
C	-2.244991	-0.619786	-0.600496
C	-3.300235	-0.226116	1.547410
C	-4.271733	-1.149829	1.207570
H	-3.333599	0.282765	2.506914
C	-4.155299	-1.772492	-0.037489
H	-5.089746	-1.392208	1.874451
H	-4.889188	-2.514698	-0.343526
O	-1.330356	-0.413362	-1.499108
C	1.260143	1.918461	2.319533
H	1.972599	2.645951	2.714555
H	0.465513	1.806180	3.061804
H	1.770903	0.959475	2.199089
C	-0.054362	3.730379	1.117360
H	0.632654	4.511280	1.453256
H	-0.482064	4.031585	0.157213
H	-0.858120	3.650116	1.854955
N	-3.188130	-1.524114	-0.914855
O	1.519911	-1.246841	-1.335797
C	1.258443	-2.217289	-2.027374
O	0.028321	-2.499367	-2.426049
C	2.283858	-3.192094	-2.524386
H	1.947140	-4.217732	-2.360168
H	2.405630	-3.048177	-3.602206
H	3.236638	-3.018408	-2.026630
H	-0.586547	-1.799016	-2.089910
S	0.177704	1.027634	-3.653867
O	1.187514	0.155756	-4.295607
O	0.208872	2.455377	-4.034692
C	-1.424466	0.421054	-4.214357
H	-2.209006	0.968083	-3.693253
H	-1.435863	0.635043	-5.285450
H	-1.497793	-0.646669	-4.021582

Int-8

SCF (wB97x) = -1779.594273
E(SCF)+ZPE(0 K)= -1779.219101
H(298 K)= -1779.191123
G(298 K)= -1779.280052
Lowest Frequency = 3.1892cm^{-1}

Cu	0.826759	1.737586	0.610317
C	-0.550198	-0.360408	2.556022
C	-0.662007	-1.454808	1.659435
C	0.054857	-2.637164	1.873254
C	0.893074	-2.786264	2.970554
C	1.002939	-1.738201	3.871420
C	0.291904	-0.561260	3.657024
H	1.439938	-3.711542	3.114046
H	1.641908	-1.823151	4.744286
C	-1.290306	0.994306	2.453474
N	-1.020537	1.539859	1.096563
C	-1.992670	1.978125	0.376033
H	-0.056669	-3.454996	1.171600
C	-2.768294	0.799704	2.839465
H	-2.795774	0.345451	3.833375
H	-3.285860	1.761556	2.907551
H	-3.298627	0.147691	2.147126
C	-0.718800	2.060266	3.410498
H	-0.927949	1.821053	4.455834
H	0.359093	2.196764	3.280897
H	-1.210620	3.009131	3.181667
H	0.409295	0.230031	4.384381
O	2.759402	1.857054	0.398282
H	-3.003795	1.892721	0.772766
C	-1.952893	2.584515	-0.945281
C	-3.185685	3.014362	-1.456288
C	-0.784801	2.782978	-1.761995
C	-3.265482	3.628872	-2.694785
H	-4.085079	2.863785	-0.863928
C	-2.071095	3.797547	-3.396893
H	-4.207507	3.970422	-3.106114
H	-2.079190	4.285359	-4.370038
O	0.371245	2.372391	-1.385234
N	-0.881166	3.396343	-2.963987
C	3.387995	2.271328	-0.585405
O	2.833868	2.676966	-1.687566
H	1.810981	2.616411	-1.669884
C	4.884751	2.335789	-0.576138

H	5.204179	3.364378	-0.761459
H	5.272595	1.989974	0.380277
H	5.278078	1.716453	-1.386270
S	-1.657507	-1.568538	0.140282
O	-2.853744	-0.733554	0.210763
O	-1.867041	-2.995410	-0.111747
C	-0.601843	-0.968498	-1.163868
H	0.307526	-1.570496	-1.167975
H	-1.162771	-1.109786	-2.089946
H	-0.372922	0.085049	-1.006841

Int-9

SCF (wB97x) = -1353.688634
E(SCF)+ZPE(0 K)= -1353.366411
H(298 K)= -1353.345264
G(298 K)= -1353.414867
Lowest Frequency = 26.3851cm^{-1}

C	-0.513541	-0.364685	2.532886
C	-0.548368	-1.440494	1.611679
C	0.178008	-2.614857	1.836206
C	0.950401	-2.774562	2.979265
C	0.989074	-1.742749	3.905852
C	0.270196	-0.573583	3.675535
H	1.505441	-3.692841	3.136663
H	1.578584	-1.835938	4.812238
C	-1.305189	0.960728	2.430133
N	-1.136723	1.490594	1.071810
C	-2.116387	2.033992	0.479814
H	0.125911	-3.413066	1.105597
C	-2.750619	0.699196	2.902669
H	-2.717174	0.223621	3.886777
H	-3.306097	1.635855	3.013179
H	-3.282556	0.042261	2.212799
C	-0.706129	2.062927	3.331115
H	-0.852288	1.849234	4.392647
H	0.358821	2.211392	3.133768
H	-1.229906	2.996616	3.110745
H	0.329381	0.203958	4.425110
H	-3.119749	2.066815	0.918988
C	-2.056513	2.630366	-0.865743
C	-3.264101	2.939105	-1.494555
C	-0.884101	2.932259	-1.595264
C	-3.286983	3.489962	-2.772112
H	-4.194866	2.736848	-0.971465
C	-2.067414	3.730523	-3.389054
H	-4.218633	3.726658	-3.271332
H	-2.019178	4.159605	-4.385894
O	0.322084	2.695015	-1.061012
N	-0.892896	3.461735	-2.810827
S	-1.460521	-1.502497	0.045910
O	-2.749939	-0.824761	0.162455
O	-1.498657	-2.907015	-0.371777
C	-0.415978	-0.642566	-1.113674
H	0.486442	-1.240685	-1.245253
H	-0.976476	-0.575915	-2.048393
H	-0.185296	0.343723	-0.710765
H	0.973956	2.984850	-1.710481

Int-10

SCF (wB97x) = -993.345022
E(SCF)+ZPE(0 K)= -993.102184
H(298 K)= -993.087204
G(298 K)= -993.141784
Lowest Frequency = 52.3836cm^{-1}

C	-0.503580	-0.436506	2.406730
C	-0.420881	-1.702993	1.776659
C	0.520909	-2.659555	2.173110
C	1.404022	-2.409300	3.214473
C	1.331282	-1.186585	3.866288
C	0.396826	-0.237461	3.463823
H	2.126978	-3.162267	3.508318
H	2.000254	-0.959113	4.690083
C	-1.517317	0.696119	2.096815
H	0.548974	-3.614053	1.661335
C	-2.821602	0.397276	2.865997
H	-2.630830	0.324946	3.941629
H	-3.537365	1.208409	2.693874
H	-3.265366	-0.540484	2.524321
C	-0.986632	2.065427	2.558186
H	-0.911118	2.143529	3.643961
H	-0.010928	2.288749	2.116066
H	-1.696057	2.835838	2.240430
H	0.378361	0.699615	4.003245
S	-1.446126	-2.304067	0.411073
O	-2.817855	-1.803999	0.534529
O	-1.288845	-3.759065	0.358779
C	-0.696018	-1.639335	-1.062804
H	0.315556	-2.040854	-1.131051
H	-1.306391	-1.981245	-1.900999
H	-0.697637	-0.552764	-0.976183
N	-1.740146	0.815558	0.647101
H	-2.538466	0.260397	0.362444
H	-1.942972	1.777512	0.407325

Int-S1

SCF (wB97x) = -1191.085207
E(SCF)+ZPE(0 K)= -1190.759685
H(298 K)= -1190.736689
G(298 K)= -1190.813733
Lowest Frequency = 19.6974cm^{-1}

C	-0.291415	0.148888	0.525647
C	0.990854	0.584000	0.857467
C	-0.983875	2.430378	0.082307
C	-1.268913	1.064745	0.141306

H	-0.531990	-0.909480	0.556293
H	1.754055	-0.131102	1.148152
N	-1.372081	4.506049	-1.121184
C	-1.557917	5.755348	-0.876948
C	-2.052388	3.462692	-0.298096
C	1.282426	1.945977	0.814849
H	2.274400	2.301620	1.076072
Cu	-0.192023	3.915968	-2.578921
C	0.299759	2.859188	0.437405
H	0.541885	3.916855	0.415172
C	-2.654711	4.005736	1.009546
H	-1.907240	4.525340	1.616712
H	-3.505593	4.670246	0.830472
H	-3.021708	3.155146	1.588687
C	-3.181791	2.885968	-1.165729
H	-2.786654	2.379958	-2.047943
H	-3.787190	2.179503	-0.592428
H	-3.836251	3.702572	-1.483324
H	-2.253128	0.698220	-0.128981
C	-0.954273	6.879942	-1.551268
C	-1.357107	8.164716	-1.158784
C	0.057415	6.763001	-2.556501
C	-0.789019	9.281905	-1.743131
H	-2.119803	8.271581	-0.391519
C	0.195201	9.062082	-2.711286
H	-1.081255	10.287077	-1.465362
H	0.676424	9.910419	-3.194214
H	-2.229778	6.033120	-0.065236
O	0.496891	5.627374	-2.978563
N	0.611337	7.865295	-3.106475
O	1.074957	2.898930	-3.816833
C	0.359652	1.867160	-3.659292
O	-0.670101	1.975730	-2.927070
C	0.725823	0.554255	-4.279803
H	-0.173165	0.001119	-4.555180
H	1.366962	0.704511	-5.148430
H	1.273632	-0.034054	-3.536949

Int-S2

SCF (wB97x) = -1779.729765
E(SCF)+ZPE(0 K)= -1779.358712
H(298 K)= -1779.329394
G(298 K)= -1779.421006
Lowest Frequency = 17.4310cm^{-1}

C	1.882248	2.604065	0.052691
Cu	0.270686	0.814222	-1.403635
C	1.834730	1.945217	-1.185445
C	2.898810	2.133783	-2.078611
H	2.880991	1.629714	-3.043116
C	3.981933	2.953903	-1.761373
H	4.797398	3.083237	-2.468510
C	4.011188	3.611688	-0.530448
H	4.845396	4.258001	-0.272323
C	2.964050	3.436611	0.370936
N	-0.202834	1.433692	0.463923
C	0.741497	2.415844	1.057277
C	-1.260616	1.106088	1.104394
H	-1.463601	1.562037	2.079520
H	3.000692	3.957045	1.325588
C	-2.266630	0.147353	0.683255
C	-2.195946	-0.618061	-0.524145
C	-3.354906	-0.066351	1.536331
C	-4.326610	-1.001815	1.216448
H	-3.426563	0.506217	2.457962
C	-4.155054	-1.713480	0.029104
H	-5.179090	-1.186426	1.858754
H	-4.882005	-2.470148	-0.260056
O	-1.240551	-0.480612	-1.382100
C	1.286116	1.854738	2.382256
H	2.024278	2.537316	2.810344
H	0.487617	1.725918	3.119467
H	1.767115	0.886898	2.213050
C	0.018650	3.755514	1.281631
H	0.720998	4.503244	1.658929
H	-0.400816	4.119914	0.339249
H	-0.789880	3.659486	2.013100
N	-3.140243	-1.539098	-0.811890
O	1.622738	-1.505012	-1.425763
C	1.207816	-2.396216	-2.145303
O	-0.072987	-2.545428	-2.440104
C	2.088500	-3.421447	-2.803841
H	1.675706	-4.423549	-2.668910
H	2.120966	-3.215491	-3.878045
H	3.096950	-3.367682	-2.395789
H	-0.600987	-1.801492	-2.023815
S	0.125572	0.892363	-3.812588
O	1.265947	0.238902	-4.551752
O	-0.116897	2.315345	-4.252063
C	-1.337554	0.017887	-4.425700
H	-2.220858	0.411705	-3.920828
H	-1.383314	0.208050	-5.501509
H	-1.228250	-1.047942	-4.222984

$MeSO_2^{\bullet}$

SCF (wB97x) = -588.465201
E(SCF)+ZPE(0 K)= -588.419619
H(298 K)= -588.413955
G(298 K)= -588.447793
Lowest Frequency = 180.3936cm^{-1}

S	-2.548991	4.307542	-4.044352
O	-2.560726	5.757091	-3.779146
O	-3.749613	3.505971	-3.748663
C	-2.147600	4.072078	-5.800814
H	-2.973487	4.502179	-6.370321
H	-1.212000	4.594195	-5.994982
H	-2.055999	3.001010	-5.974422

$Cu(OAc)_2^-$

SCF (wB97x) = -654.504709
E(SCF)+ZPE(0 K)= -654.402773
H(298 K)= -654.389928
G(298 K)= -654.446232
Lowest Frequency = 6.7890cm^{-1}

C	2.947509	-1.763059	-1.135611
O	2.021100	-1.570143	-2.009189
O	2.834402	-1.552193	0.075654
Cu	0.334956	-0.919364	-1.549662
C	4.250151	-2.289759	-1.717152
H	4.640127	-1.576621	-2.449267
H	4.065947	-3.230414	-2.244016
H	4.991731	-2.448979	-0.933639
C	-2.018565	0.525986	-1.820025
O	-1.379535	-0.334729	-1.106367
O	-1.588069	1.050581	-2.851483
C	-3.400482	0.871618	-1.288027
H	-4.009328	-0.035068	-1.227229
H	-3.894887	1.600627	-1.930902
H	-3.314688	1.275723	-0.275194

amineCuOAc

SCF (wB97x) = -831.355703
E(SCF)+ZPE(0 K)= -831.097628
H(298 K)= -831.079888
G(298 K)= -831.144910
Lowest Frequency = 28.2286cm^{-1}

Cu	0.705689	2.101367	0.682265
C	-0.422579	-0.184442	2.527617
C	-0.652921	-1.155274	1.543019
C	0.118741	-2.311393	1.477107
C	1.136490	-2.527256	2.406239
C	1.369705	-1.576345	3.395154
C	0.597968	-0.415230	3.453112
H	1.740249	-3.427922	2.357961
H	2.157906	-1.730634	4.125506
C	-1.281840	1.084063	2.554057
H	-0.076517	-3.044119	0.700362
C	-2.762386	0.724862	2.755108
H	-2.896749	0.221653	3.716327
H	-3.378434	1.629914	2.750613
H	-3.126874	0.056558	1.969722
C	-0.854100	2.084049	3.629202
H	-0.988651	1.659046	4.626696
H	0.191288	2.385281	3.513029
H	-1.476133	2.982221	3.565681
H	0.808807	0.308052	4.230796
O	2.450046	2.495762	0.154335
C	3.311395	1.544129	0.026099
O	3.070279	0.349107	0.217444
C	4.695970	2.006846	-0.393722
H	4.635202	2.522317	-1.356528
H	5.082535	2.723444	0.336517
H	5.380948	1.162402	-0.474982
H	-1.443443	-1.020617	0.809268
N	-1.108492	1.770005	1.226077
H	-1.548280	1.211654	0.496957
H	-1.638604	2.639756	1.240934

TS-3/4

SCF (wB97x) = -1420.145646
E(SCF)+ZPE(0 K)= -1419.762363
H(298 K)= -1419.734023
G(298 K)= -1419.823110
Lowest Frequency = -1309.7437cm^{-1}

C	-0.718174	1.288504	-0.165234
C	-0.732285	2.690464	-0.225951
C	-0.751396	3.363428	-1.444020
C	-0.752014	2.625323	-2.626760
C	-0.720206	1.230864	-2.592561
C	-0.695362	0.556274	-1.370985
H	-0.741841	3.254901	0.702373
H	-0.767580	4.448551	-1.475569
H	-0.767727	3.134777	-3.585710
H	-1.517376	0.879539	0.850112
Cu	0.459952	0.231692	1.178837
C	-0.656155	-0.971616	-1.283228
N	0.137836	-1.278402	-0.060381
O	-2.503228	0.671996	1.648920
C	-2.132263	-0.022687	2.660499
O	-0.960046	-0.399336	2.833865
C	-3.214092	-0.388708	3.649860
H	-3.777322	0.503116	3.935060
H	-3.912915	-1.081947	3.172078
H	-2.785010	-0.860219	4.534083
H	-0.705942	0.688233	-3.531588
C	-2.089773	-1.504527	-1.104573
H	-2.698435	-1.215489	-1.965747
H	-2.090268	-2.597022	-1.034391
H	-2.544534	-1.090873	-0.200994
C	0.003584	-1.608229	-2.513091
H	0.064875	-2.693925	-2.408397
H	-0.590460	-1.414620	-3.408689
H	1.011722	-1.212967	-2.665180
O	0.950886	1.913480	2.255406
C	1.657919	2.037691	3.261166
O	2.404823	1.084178	3.735270
C	1.703072	3.320347	4.032420
H	1.252685	3.155448	5.015385
H	2.740445	3.623678	4.188789
H	1.153979	4.097502	3.503730
C	0.442145	-2.486416	0.235820

H	0.090662	-3.299100	-0.405876
C	1.241861	-2.916382	1.366918
C	1.363066	-4.290692	1.601233
C	1.956486	-2.025101	2.224178
C	2.142259	-4.754823	2.649175
H	0.838398	-4.989003	0.954333
C	2.799891	-3.801147	3.425681
H	2.248100	-5.811987	2.859371
H	3.428605	-4.115404	4.255991
O	1.920270	-0.736994	2.063068
H	2.311367	0.255858	3.152113
N	2.722691	-2.487689	3.226530

TS-7/8

SCF (wB97x) = -1779.546625
E(SCF)+ZPE(0 K)= -1779.174337
H(298 K)= -1779.146303
G(298 K)= -1779.232691
Lowest Frequency = -167.3463cm^{-1}

Cu	0.523960	0.989807	0.080855
C	-0.380842	-0.222433	2.353473
C	0.504455	-0.512239	1.299063
C	1.628575	-1.313758	1.455386
C	1.898010	-1.864793	2.706907
C	1.016848	-1.637077	3.760003
C	-0.100671	-0.821560	3.582575
H	2.778574	-2.483285	2.845103
H	1.202346	-2.084130	4.731145
S	-0.294073	-1.210813	-0.700199
C	-1.149441	-0.334127	-2.029208
H	-0.437013	0.293180	-2.562007
H	-1.546744	-1.127002	-2.667574
H	-1.958167	0.253912	-1.596446
O	-1.332133	-2.057887	-0.092135
O	0.860356	-1.884549	-1.315755
C	-1.542116	0.758668	2.188369
N	-1.263016	1.490117	0.931675
C	-2.119504	2.290164	0.422283
H	2.268361	-1.524004	0.604977
C	-2.873201	-0.008895	2.119217
H	-3.002897	-0.599011	3.030434
H	-3.720314	0.679369	2.046793
H	-2.879169	-0.686484	1.262659
C	-1.559726	1.777283	3.341188
H	-1.757063	1.289021	4.297970
H	-0.603139	2.303159	3.404097
H	-2.353463	2.511194	3.174895
H	-0.753438	-0.635051	4.429726
O	2.563657	1.503615	0.223633
H	-3.092647	2.430035	0.905740
C	-1.935985	3.077179	-0.789741
C	-2.998891	3.906329	-1.170115
C	-0.755957	3.086656	-1.605603
C	-2.900210	4.716128	-2.289748
H	-3.905080	3.912026	-0.569472
C	-1.703044	4.667358	-3.002595
H	-3.709204	5.365709	-2.600676
H	-1.567754	5.292257	-3.883055
O	0.275232	2.339759	-1.363340
N	-0.673028	3.888939	-2.685038
C	3.240935	2.202474	-0.532656
O	2.747700	2.842037	-1.558300
H	1.742647	2.712787	-1.605892
C	4.716286	2.384091	-0.339286
H	4.930527	3.442771	-0.170972
H	5.060817	1.795165	0.508911
H	5.242885	2.080077	-1.247340

TS-S1/5

SCF (wB97x) = -1191.040244
E(SCF)+ZPE(0 K)= -1190.719836
H(298 K)= -1190.697519
G(298 K)= -1190.771599
Lowest Frequency = -1350.6939cm^{-1}

C	1.904449	2.403878	0.011565
Cu	0.873478	-0.204495	-0.273897
H	2.780922	0.114121	0.519641
O	3.352958	-0.823985	1.083019
C	2.970613	-1.878678	0.473584
O	1.985343	-1.883254	-0.303970
C	2.442494	1.152894	-0.362256
C	3.435365	1.115082	-1.350403
H	3.854293	0.155762	-1.646891
C	3.920555	2.282639	-1.938667
H	4.694045	2.236243	-2.698929
C	3.403475	3.509586	-1.533982
H	3.772548	4.428856	-1.978772
C	2.394453	3.571575	-0.568583
N	-0.134079	1.292145	0.498898
C	0.739911	2.384903	1.008296
C	-1.414979	1.343241	0.556897
H	-1.891823	2.224477	0.989104
C	3.758493	-3.138007	0.694917
H	3.231944	-3.999015	0.285322
H	4.727993	-3.033788	0.198968
H	3.943672	-3.275544	1.762093
H	1.993561	4.542031	-0.297606
C	-2.329837	0.328179	0.088707
C	-1.917881	-0.903115	-0.523759
C	-3.701951	0.573304	0.240056
C	-4.630302	-0.356238	-0.192298
H	-4.026902	1.502955	0.700976
C	-4.132463	-1.524429	-0.777364
H	-5.696409	-0.196040	-0.088165
H	-4.824117	-2.285303	-1.134784
O	-0.687218	-1.225796	-0.706688

C	1.243172	1.960892	2.401330
H	1.962195	2.696064	2.773299
H	0.402412	1.904191	3.099378
H	1.733331	0.984868	2.370083
C	0.022444	3.729864	1.121201
H	0.728608	4.491510	1.458901
H	-0.404160	4.048018	0.165341
H	-0.771468	3.694235	1.871382
N	-2.844760	-1.797910	-0.941486

Int-S3

SCF (wB97x) = -1465.323586
E(SCF)+ZPE(0 K)= -1464.804799
H(298 K)= -1464.772077
G(298 K)= -1464.869636
Lowest Frequency = 15.9595cm^{-1}

C	-1.144678	-0.101146	0.630588
C	0.026623	0.078610	1.359437
C	-1.419422	2.305304	0.410740
C	-1.858316	1.003015	0.159214
H	-1.510448	-1.102055	0.422547
H	0.583793	-0.778459	1.724737
N	-1.311357	4.259212	-1.068633
C	-2.181436	3.540148	-0.087405
C	0.480884	1.373375	1.611507
H	1.398267	1.530994	2.170385
C	-0.236210	2.468717	1.143129
H	0.148288	3.462513	1.347361
C	-2.494803	4.482044	1.088513
H	-1.585996	4.824047	1.591392
H	-3.037194	5.362463	0.729081
H	-3.118618	3.968909	1.826052
C	-3.494457	3.188712	-0.790370
H	-3.336548	2.554819	-1.665003
H	-4.171547	2.683052	-0.097137
H	-3.984662	4.107018	-1.127632
H	-2.767098	0.828140	-0.405208
H	-0.495969	4.635458	-0.591154
H	-1.824445	5.043280	-1.480300
Cu	-0.528368	3.323923	-2.689962
O	1.134508	3.220034	-1.659808
C	1.834583	2.151137	-1.535836
C	3.068028	2.294342	-0.666121
H	3.393864	1.320642	-0.297630
H	3.870566	2.723357	-1.276546
H	2.879990	2.971639	0.168970
O	1.585860	1.075622	-2.098583
O	-2.191277	3.428088	-3.720238
C	-2.891232	4.497069	-3.844060
C	-4.124494	4.354190	-4.714114
H	-3.936315	3.677054	-5.549305
H	-4.927198	3.925088	-4.103962
H	-4.450172	5.328002	-5.082447
O	-2.642589	5.572408	-3.280932
N	0.254521	2.388525	-4.311257
H	-0.560860	2.012289	-4.788754
H	0.767546	1.604457	-3.899506
C	1.124708	3.107415	-5.292527
C	2.437907	3.458458	-4.589710
H	3.115002	3.964043	-5.282997
H	2.280281	4.092302	-3.714990
H	2.927955	2.539999	-4.252631
C	1.437705	2.165493	-6.468532
H	0.528768	1.823814	-6.971397
H	2.061653	2.678475	-7.206065
H	1.979821	1.284870	-6.109181
C	0.362965	4.342495	-5.790496
C	-0.820300	4.179438	-6.522872
C	0.802148	5.644658	-5.538803
C	-1.537182	5.274998	-6.991071
H	-1.205017	3.185754	-6.727223
C	0.088724	6.749033	-6.009996
H	1.710993	5.819274	-4.974402
C	-1.082639	6.569629	-6.738837
H	-2.454615	5.117641	-7.549939
H	0.454711	7.749835	-5.801826
H	-1.639637	7.426867	-7.103998

Int-S4

SCF (wB97x) = -1059.830053
E(SCF)+ZPE(0 K)= -1059.519617
H(298 K)= -1059.496719
G(298 K)= -1059.575315
Lowest Frequency = 16.8794cm^{-1}

C	-0.905796	0.013835	0.353133
C	0.241124	0.176015	1.124725
C	-1.239322	2.420809	0.282918
C	-1.639295	1.126762	-0.061648
H	-1.237646	-0.980413	0.070132
H	0.811824	-0.688850	1.448279
N	-1.115606	4.333033	-1.228180
C	-1.989750	3.665681	-0.208368
C	0.649007	1.461646	1.479597
H	1.542147	1.605008	2.079877
C	-0.083237	2.567332	1.060156
H	0.266718	3.554578	1.342953
C	-2.257233	4.646136	0.944523
H	-1.334827	4.983275	1.424028
H	-2.785752	5.528189	0.570102
H	-2.880081	4.166848	1.704837
C	-3.323591	3.338419	-0.885792
H	-3.202447	2.706859	-1.769294
H	-3.989327	2.832601	-0.182162
H	-3.807565	4.265124	-1.206414
H	-2.528816	0.965592	-0.659990
H	-0.284874	4.709969	-0.777869

H	-1.615070	5.112040	-1.672216
Cu	-0.527197	3.220790	-2.769325
O	1.242756	2.577649	-2.047742
C	1.341754	1.777834	-3.028465
C	2.493491	0.825947	-3.128124
H	2.623711	0.484542	-4.154990
H	3.406225	1.296540	-2.759712
H	2.277468	-0.039298	-2.493511
O	0.418138	1.796422	-3.893902
O	-1.963356	3.575617	-3.983887
C	-2.670741	4.652212	-4.050534
C	-3.780445	4.605539	-5.080994
H	-3.409010	4.203473	-6.026175
H	-4.563667	3.931398	-4.719467
H	-4.206708	5.597259	-5.233860
O	-2.508370	5.648994	-3.338097

Int-S5

SCF (wB97x) = -1059.797799
E(SCF)+ZPE(0 K)= -1059.488247
H(298 K)= -1059.465135
G(298 K)= -1059.543359
Lowest Frequency = 19.1308cm^{-1}

C	1.847923	2.580237	-0.154178
Cu	0.340734	0.429957	-1.181485
H	3.001740	-0.041908	-1.199005
O	3.350202	-0.917372	-1.460124
C	2.432004	-1.556837	-2.160191
O	1.296734	-1.126615	-2.318913
C	1.773167	1.754081	-1.293747
C	2.633220	2.027480	-2.365529
H	2.591022	1.412971	-3.263395
C	3.555072	3.077311	-2.311890
H	4.210501	3.273878	-3.155681
C	3.628048	3.869207	-1.168388
H	4.343841	4.684146	-1.112972
C	2.774959	3.621938	-0.090934
N	-0.267012	1.567962	0.394540
C	0.901680	2.252828	1.003392
C	2.910272	-2.850319	-2.739276
H	2.094979	-3.349452	-3.259216
H	3.733116	-2.653589	-3.431330
H	3.294137	-3.487213	-1.938606
H	2.842023	4.253756	0.790367
O	-1.254525	-0.689522	-1.267845
C	1.577374	1.258858	1.958631
H	2.487314	1.699969	2.373585
H	0.907504	1.002325	2.786531
H	1.851865	0.338377	1.433584
C	0.433420	3.490799	1.775658
H	1.260626	3.960923	2.313986
H	-0.001591	4.233946	1.099544
H	-0.322110	3.202657	2.513105
H	-0.941540	2.244126	0.046889
H	-0.758144	0.937194	1.031140
C	-1.982558	-1.082965	-0.288153
O	-1.760418	-0.840793	0.907756
C	-3.190274	-1.912720	-0.691248
H	-3.796108	-1.358496	-1.413501
H	-2.853386	-2.829955	-1.183308
H	-3.796683	-2.169334	0.177947

Int-S7

SCF (wB97x) = -1648.307816
E(SCF)+ZPE(0 K)= -1647.948726
H(298 K)= -1647.920448
G(298 K)= -1648.009437
Lowest Frequency = 8.7889cm^{-1}

C	2.093845	2.433012	-0.029901
Cu	0.040909	1.061383	-1.282647
H	0.611122	-1.847767	-0.714241
O	1.596335	-1.939449	-0.900573
C	1.894023	-1.332229	-2.032795
O	1.057565	-0.720554	-2.681397
C	1.686912	2.073716	-1.322609
C	2.493847	2.294366	-2.424580
H	2.181551	1.983645	-3.414124
C	3.713734	2.955214	-2.248026
H	4.350416	3.135912	-3.108498
C	4.107398	3.376411	-0.981101
H	5.051761	3.894426	-0.848044
C	3.304443	3.107638	0.126407
N	0.567609	0.760163	0.590568
C	1.199602	2.010769	1.115999
C	3.344314	-1.398131	-2.406207
H	3.458644	-1.248231	-3.479255
H	3.855912	-0.579996	-1.886564
H	3.795266	-2.340149	-2.092667
H	3.635963	3.402798	1.117380
O	-1.674671	0.192523	-1.084923
C	1.950794	1.705762	2.412396
H	2.365064	2.620764	2.843245
H	1.265163	1.273566	3.147214
H	2.771105	1.002912	2.238956
C	0.093917	3.044424	1.360313
H	0.542846	3.986756	1.682890
H	-0.480542	3.236613	0.448848
H	-0.590127	2.702127	2.143521
H	-0.234993	0.480634	1.150461
H	1.231487	-0.012024	0.632423
C	-1.837867	-1.002244	-0.660005
O	-0.917992	-1.764471	-0.309894
C	-3.272095	-1.475823	-0.608835
H	-3.358181	-2.385932	-0.015491
H	-3.917847	-0.693640	-0.205199
H	-3.603079	-1.685619	-1.631179

S	-0.714363	1.989933	-3.184130
O	0.252115	2.373637	-4.230883
C	-1.715707	0.698943	-3.942417
H	-2.072264	1.125745	-4.882058
H	-1.066847	-0.159297	-4.117767
H	-2.532541	0.449520	-3.270216
O	-1.623026	3.055887	-2.709351

Int-S8

SCF (wB97x) = -1648.346129
E(SCF)+ZPE(0 K)= -1647.984586
H(298 K)= -1647.956788
G(298 K)= -1648.044615
Lowest Frequency = 14.1856cm^{-1}

Cu	-1.301723	2.352730	0.152935
C	-0.405161	0.199763	2.220687
C	0.051959	-0.923989	1.486508
C	1.407609	-1.260302	1.450927
C	2.362687	-0.484308	2.092298
C	1.948013	0.643367	2.783241
C	0.594569	0.958204	2.845426
H	3.409311	-0.763112	2.043272
H	2.667580	1.280165	3.286598
C	-1.877678	0.642740	2.410737
H	1.742445	-2.138143	0.913412
C	-2.731083	-0.492237	3.005024
H	-2.321472	-0.788848	3.974484
H	-3.753852	-0.136058	3.161331
H	-2.781988	-1.375008	2.370183
C	-2.020497	1.847104	3.352763
H	-1.655544	1.616386	4.356069
H	-1.507202	2.736310	2.976548
H	-3.082853	2.093031	3.438841
H	0.317857	1.836729	3.410573
O	0.726030	0.516877	-1.412388
O	-0.095393	3.525066	-0.680735
C	1.858752	0.951404	-1.304210
O	2.123762	2.221320	-1.056682
H	1.274957	2.746011	-0.961346
C	3.099606	0.112348	-1.451354
H	3.647865	0.433415	-2.341725
H	3.754178	0.256723	-0.588752
H	2.836203	-0.940217	-1.548552
N	-2.410838	1.095700	1.085911
H	-2.536601	0.272743	0.491433
H	-3.340563	1.483341	1.232893
C	-0.334917	4.796383	-0.823854
O	-1.377913	5.335134	-0.470531
C	0.794625	5.572328	-1.470410
H	1.692853	5.497798	-0.850291
H	1.032260	5.138016	-2.445593
H	0.522302	6.620551	-1.591491
S	-0.973700	-2.101576	0.566728
O	-1.220325	-3.263171	1.419704
O	-2.148566	-1.424474	0.002133
C	-0.001667	-2.603939	-0.841962
H	0.376508	-1.701759	-1.328748
H	0.791574	-3.288049	-0.546551
H	-0.719465	-3.120219	-1.482390

TS-S4/S5

SCF (wB97x) = -1059.782744
E(SCF)+ZPE(0 K)= -1059.477948
H(298 K)= -1059.455503
G(298 K)= -1059.532101
Lowest Frequency = -1354.9353cm^{-1}

C	1.937116	2.461698	0.012340
Cu	0.779007	-0.128536	-0.281273
H	2.705279	0.147305	0.478497
O	3.256688	-0.801350	1.058258
C	2.832249	-1.854625	0.477039
O	1.837230	-1.842575	-0.289095
C	2.397501	1.190434	-0.398455
C	3.377491	1.123512	-1.397824
H	3.742083	0.151047	-1.721977
C	3.920684	2.278371	-1.961364
H	4.681077	2.208638	-2.732978
C	3.483427	3.522242	-1.515375
H	3.903210	4.429855	-1.938607
C	2.490260	3.615460	-0.535794
N	-0.178494	1.466641	0.483527
C	0.779667	2.479176	1.011675
C	3.580180	-3.135383	0.714185
H	3.011264	-3.988054	0.345796
H	4.536899	-3.081402	0.186246
H	3.792959	-3.248566	1.778963
H	2.147634	4.597427	-0.226287
O	-0.847043	-1.095186	-0.719909
C	1.256812	2.008955	2.392580
H	2.023421	2.690454	2.769533
H	0.422472	2.001534	3.101594
H	1.684295	1.003567	2.350678
C	0.084504	3.836185	1.137472
H	0.750573	4.578233	1.586085
H	-0.244573	4.210883	0.163064
H	-0.791918	3.739390	1.784855
H	-0.751252	1.870124	-0.254064
H	-0.816636	1.104487	1.193175
C	-1.768843	-1.289926	0.155093
O	-1.726324	-0.876706	1.321333
C	-2.961414	-2.082404	-0.348491
H	-3.432415	-1.546857	-1.177842
H	-2.626386	-3.050038	-0.731912
H	-3.690392	-2.235007	0.447804

TS-S7/S8

SCF (wB97x) = -1648.291330
E(SCF)+ZPE(0 K)= -1647.933336
H(298 K)= -1647.905687
G(298 K)= -1647.991994
Lowest Frequency = -141.2382cm^{-1}

Cu	0.198699	0.955876	-0.145252
C	-0.227616	-0.406887	2.167214
C	0.361109	-0.664214	0.919944
C	1.469162	-1.490592	0.764388
C	2.014451	-2.111662	1.884460
C	1.413809	-1.928600	3.128263
C	0.313012	-1.086156	3.263159
H	2.884040	-2.751345	1.777659
H	1.810867	-2.433281	4.003079
S	-0.913692	-1.075787	-0.872142
C	-1.434841	-0.158378	-2.339284
H	-0.552556	0.082655	-2.930542
H	-2.090969	-0.854453	-2.868351
H	-1.963822	0.743963	-2.030480
O	-2.155767	-1.354282	-0.131085
O	-0.145980	-2.212258	-1.398279
C	-1.302209	0.664410	2.369291
H	1.878863	-1.677533	-0.221622
C	-2.588856	0.060744	2.947737
H	-2.405524	-0.399003	3.922873
H	-3.338186	0.847778	3.077741
H	-2.985542	-0.702241	2.272489
C	-0.743305	1.751381	3.300947
H	-0.496818	1.341906	4.282660
H	0.160613	2.194215	2.872040
H	-1.493443	2.536896	3.442039
H	-0.113623	-0.929121	4.249597
O	1.924218	1.850490	0.781525
O	0.328975	2.302426	-1.606514
C	2.779362	2.569792	0.268208
O	2.674646	3.050005	-0.944673
H	1.775541	2.791913	-1.330893
C	4.034297	2.965777	0.985968
H	4.901727	2.635593	0.408629
H	4.082072	4.055101	1.061544
H	4.056072	2.520847	1.979264
N	-1.558886	1.305331	1.060583
H	-2.347380	0.865584	0.596114
H	-1.796572	2.285007	1.167578
C	-0.627084	3.116792	-1.941880
O	-1.799526	2.963627	-1.610010
C	-0.186783	4.286956	-2.798879
H	0.553885	4.883150	-2.257114
H	0.292921	3.917167	-3.709478
H	-1.037323	4.915996	-3.061017

benzene

SCF (wB97x) = -232.558020
E(SCF)+ZPE(0 K)= -232.079312
H(298 K)= -232.074005
G(298 K)= -232.106766
Lowest Frequency = 413.7684cm^{-1}

C	-0.866256	-0.466765	0.000066
C	0.529427	-0.467301	0.000485
C	1.227158	0.741466	-0.000080
C	0.529220	1.950102	-0.001061
C	-0.866433	1.950384	-0.001477
C	-1.564430	0.741771	-0.000912
H	1.071824	-1.407985	0.001248
H	2.313084	0.742228	0.000242
H	1.072272	2.890437	-0.001501
H	-1.408728	2.891220	-0.002240
H	-2.650275	0.741170	-0.001234
H	-1.409401	-1.407257	0.000506

arenium

SCF (wB97x) = -232.180810
E(SCF)+ZPE(0 K)= -232.446465
H(298 K)= -232.440477
G(298 K)= -232.474566
Lowest Frequency = 192.6988cm^{-1}

C	-0.887165	-0.503113	0.000083
C	0.576797	-0.465496	0.000495
C	1.251343	0.724420	-0.000058
C	0.514637	1.924908	-0.001037
C	-0.893285	1.962853	-0.001492
C	-1.586451	0.783663	-0.000939
H	-1.240349	-1.115347	-0.850159
H	1.110339	-1.410296	0.001259
H	2.333804	0.756547	0.000239
H	1.057592	2.865588	-0.001495
H	-1.406527	2.916479	-0.002267
H	-2.671403	0.773647	-0.001255
H	-1.240843	-1.114283	0.850885

1) Marenich, A. V.; Bloino, J.; Janesko, B. G.; Gomperts, R.; Mennucci, B.; Hratchian, H. P.; Ortiz, J. V.; Izmaylov, A. F.; Sonnenberg, J. L.; Williams-Young, D.; Ding, F.; Lipparini, F.; Egidi, F.; Goings, J.; Peng, B.; Petrone, A.; Henderson, T.; Ranasinghe, D.; Zakrzewski, V. G.; Gao, J.; Rega, N.; Zheng, G.; Liang, W.; Hada, M.; Ehara, M.; Toyota, K.; Fukuda, R.; Hasegawa, J.; Ishida, M.; Nakajima, T.; Honda, Y.; Kitao, O.; Nakai, H.; Vreven, T.; Throssell, K.; Montgomery, J. A., Jr.; Peralta, J. E.; Ogliaro, F.; Bearpark, M. J.; Heyd, J. J.; Brothers, E. N.; Kudin, K. N.; Staroverov, V. N.; Keith, T. A.; Kobayashi, R.; Normand, J.; Raghavachari, K.; Rendell, A. P.; Burant, J. C.; Iyengar, S. S.; Tomasi, J.; Cossi, M.; Millam, J. M.; Klene, M.; Adamo, C.; Cammi, R.; Ochterski, J. W.; Martin, R. L.; Morokuma, K.; Farkas, O.; Foresman, J. B.; Fox, D. J. Gaussian, Inc., Wallingford CT, 2016.
2) Chai, J.-D.; Head-Gordon, M. Systematic Optimization of Long-Range Corrected Hybrid Density Functionals. *J. Chem. Phys.* **2007**, *128*, 084106.
3) Alipour, M.; Fallahzadeh, P. First Principles Optimally Tuned Range-Separated Density Functional Theory for Prediction of Phosphorus–Hydrogen Spin–Spin Coupling Constants. *Phys. Chem. Chem. Phys.* **2016**, *18*, 18431–18440
4) Becke, A. D. Density-Functional Thermochemistry. III. The Role of Exact Exchange. *J. Chem. Phys.* **1993**, *98*, 5648–5652
5) Lee, C.; Yang, W.; Parr, R. G. Development of the Colle-Salvetti Correlation-Energy Formula into a Functional of the Electron Density. *Phys. Rev. B* **1988**, *37*, 785–789
6) Grimme, S.; Ehrlich, S.; Goerigk, L. Effect of the Damping Function in Dispersion Corrected Density Functional Theory. *J. Comp. Chem*. **2011**, *32*, 1456–1465
7) Becke, A. D.; Johnson, E. R. A Density-Functional Model of the Dispersion Interaction. *J. Chem. Phys.* **2005**, 123, 154101
8) Zhao, Y.; Truhlar, D. G. The M06 Suite of Density Functionals for Main Group Thermochemistry, Thermochemical Kinetics, Noncovalent Interactions, Excited States, and Transition Elements: Two New Functionals and Systematic Testing of Four M06-Class Functionals and 12 Other Functionals. *Theor. Chem. Account.* **2008**, *120*, 215–241
9) Grimme, S.; Antony, J.; Ehrilich, S.; Krieg, H. A Consistent and Accurate Ab Initio Parametrization of Density Functional Dispersion Correction (DFT-D) for the 94 Elements H-Pu. *J. Chem. Phys.* **2010**, *132*, 154104.
10) Perdew, J. P.; Burke, K.; Ernzerhof, M. Generalized Gradient Approximation Made Simple. *Phys. Rev. Lett.* **1996**, *77*, 3865–3868
11) Adamo, C.; Barone, V. Toward Reliable Density Functional Methods Without Adjustable Parameters: The PBE0 Model*. J. Chem. Phys.* **1999**, *110*, 6158–6170
12) NBO 6.0. Glendening, E. D.; Badenhoop, J. K.; Reed, A. E.; Carpenter, J. E.; Bohmann, J. A.; Morales, C. M.; Landis, C. R.; Weinhold, F. Theoretical Chemistry Institute, University of Misconsin, Madison (2013)
13) J. I. Higham, J. A. Bull, *Angew. Chem. Int. Ed.* **2022**, *61*, e202202933
14) F. Xu, X. Y. Qian, Y. J. Li, H. C. Xu, *Org. Lett.* **2017**, *19*, 6332–6335.
15) M. Kapoor, P. Chand-Thakuri, M. C. Young, *J. Am. Chem. Soc.* **2019**, *141*, 7980–7989.
